# Supplementary material for: TDP1 phosphorylation by CDK1 in mitosis promotes MUS81-dependent repair of trapped Top1-DNA covalent complexes
Source: EMBO J. 2024 Jul 16;43(17):3710–32. doi: 10.1038/s44318-024-00169-3 (PMC11377750; doi:10.1038/s44318-024-00169-3)
Supplement: Supplementary file 1 — Appendix [file 44318_2024_169_MOESM1_ESM.pdf]

Appendix

TDP1 phosphorylation by CDK1 in mitosis promotes MUS81-dependent repair of trapped Top1-DNA covalent complexes

Srijita Paul Chowdhuri[1], and Benu Brata Das[1*],
[1]Laboratory of Molecular Biology, School of Biological Sciences, Indian Association for the Cultivation of Science, 2A & B, Raja S. C. Mullick Road, Jadavpur, Kolkata-700032, INDIA.

Table of Contents	Page Number
**Appendix Fig S1**	**2**
**Appendix Fig S2**	**4**
**Appendix Fig S3**	**5**
**Appendix Fig S4**	**6**
**Appendix Fig S5**	**7**
**Appendix Fig S6**	**8**
**Appendix Fig S7**	**10**

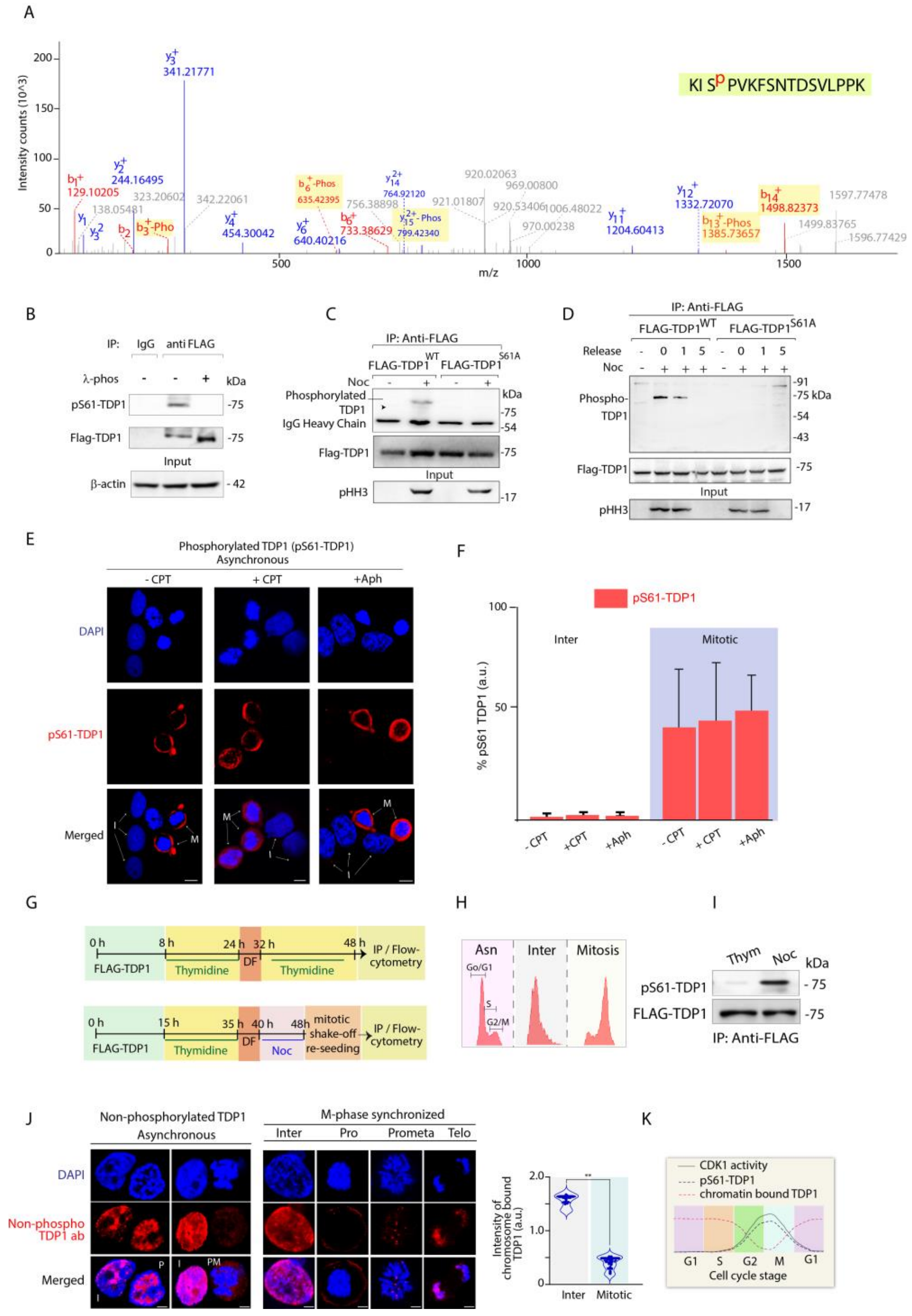

Appendix Figure S1: **Mitotic phosphorylation of TDP1 at S61.** (**A**) MS/MS spectrum of the TDP1 phosphopeptide harbouring the S61 residue (**B**) MCF7 cells ectopically expressing FLAG-TDP1 was co-immunoprecipitated using an anti-FLAG antibody following pretreatment of cell lysates with λ-phosphatase. Immune complexes were blotted with an anti-pS61-TDP1 antibody.

The same blot was stripped and re-probed with anti-FLAG antibody to show the expression of FLAG-TDP1. Aliquots (10%) of the input show the level of β-actin prior to immunoprecipitation as loading control. Note: λ-phosphatase treatment completely ablates the phosphorylation mark at S61 residue of TDP1. (**C**) MCF7 cells complemented with FLAG-TDP1^{WT} or FLAG-TDP1^{S61A} were left asynchronous or synchronized to the mitosis. Following immunoprecipitation with an anti-FLAG antibody, the immune complexes were subjected to western blotting. The anti-MPM2 antibody recognizes a single band (phosphorylated TDP1) with a molecular weight corresponding to TDP1 in the MCF7 cells, which have been synchronized at the mitotic phase. This blot was stripped and re-probed with an anti-FLAG antibody (FLAG-TDP1) to confirm equal levels of immunoprecipitation for each condition. Aliquots (10%) of the input show the level of phospho-histone H3 at Ser10 (anti-pHH3) as a marker of mitotic state prior to immunoprecipitation. (**D**) Detection of the temporal kinetics of phosphorylation on TDP1 using the anti-MPM2 antibody. Following mitotic synchronization, FLAG-TDP1 variants were immunoprecipitated at the indicated time points (0, 1, and 5 h post noc release) using the anti-FLAG antibody, and immune complexes were blotted with the anti-MPM2 antibody. The same blot was stripped and re-probed with anti-FLAG antibody. Phospho-histone H3 (anti-pHH3) indicates mitotic state prior to immunoprecipitation in the aliquots (10%) of the input. (**E**) Field images showing immunolocalization of endogenous pS61-TDP1 (red) in asynchronously growing MCF7 cells treated with or without CPT (15 nM, 24 h) or aphidicolin (0.4 μM, 24 h) detected with anti-pS61-TDP1 antibody (red). Cells at interphase and mitosis were tallied on the basis of their chromatin morphology, as indicated by DAPI staining (blue). (**F**) Bar graph showing the intensity of pS61-TDP1 for interphase and mitotic cells following RS with CPT or Aph. Intensities from 50 nuclei per stage were expressed as mean ± s.d. a.u: arbitrary unit. (**G**) Schematic representation for the protocol followed for synchronization of MCF7 cells expressing ectopic FLAG-TDP1^{WT} to interphase and mitotic phase respectively for immunoprecipitation or PI-RNase based flow cytometry analysis (**H**) Flow cytometry profile of the MCF7 cells expressing ectopic FLAG-TDP1^{WT} left asynchronous or synchronized to interphase or mitosis. (**I**) Ectopic FLAG-TDP1^{WT} in MCF7 cells were synchronized to interphase or mitosis, following immunoprecipitation with anti-FLAG antibody, the immune complexes were analyzed by western blotting with TDP1-S61 phospho-specific antibody (pS61-TDP1) which recognizes a single band with a molecular weight corresponding to TDP1 in the mitotic phase only. The same

blot was stripped and re-probed with anti-FLAG antibody (FLAG-TDP1). Please note: pS61-TDP1 antibody picks up signal only in FLAG-TDP1 expressing mitotic cells and not interphase cells. (**J**) MCF7 cells were left asynchronous or fixed at different time intervals after release from nocodazole and stained with a control immune-peptide TDP1 antibody to detect the endogenous TDP1-S61 epitope (red). Cells at interphase and the indicated distinct stages of mitotic propagation were tallied on the basis of their chromatin morphology, as indicated by DAPI staining. The fluorescence intensity of chromosome-bound TDP1 in interphase and mitotic phases was quantified. Staining intensities from 20 nuclei per stage were expressed as mean ± s.d. a.u., arbitrary unit. Interphase (I), Prophase (P), and Prometaphase (PM). Asterisks denote statistically significant differences (**P < 0.001; t-test). (**K**) A schematic representation shows the correlation between the relative distributions of TDP1 and pS61-TDP1 and the activity of CDK1 across the different cell cycle stages, as indicated. Scale bars 10 μm.

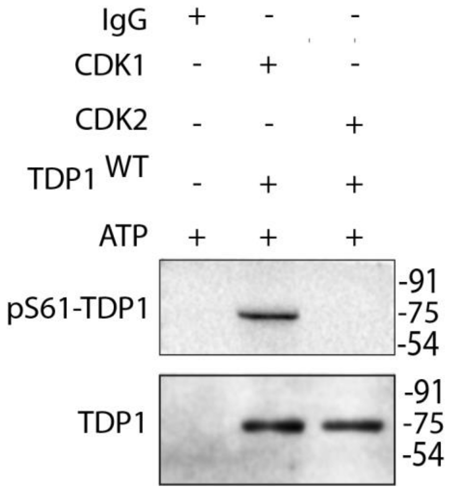

Appendix Figure S2: TDP1 is phosphorylated at S61 by CDK1 not CDK2. *In vitro* kinase assays with immunoprecipitated endogenous CDK2 or CDK1 from MCF7 cells in the presence of ATP. The substrates were recombinant 6xHis-tagged $TDP1^{WT}$. Western blotting of the kinase assay products was done with anti-TDP1-pS61 antibody. Western blotting against the anti-TDP1 antibody shows the amount of substrate in each reaction. Immunoprecipitated IgG was used as a negative control. Protein molecular weight markers (kDa) are indicated on the right.

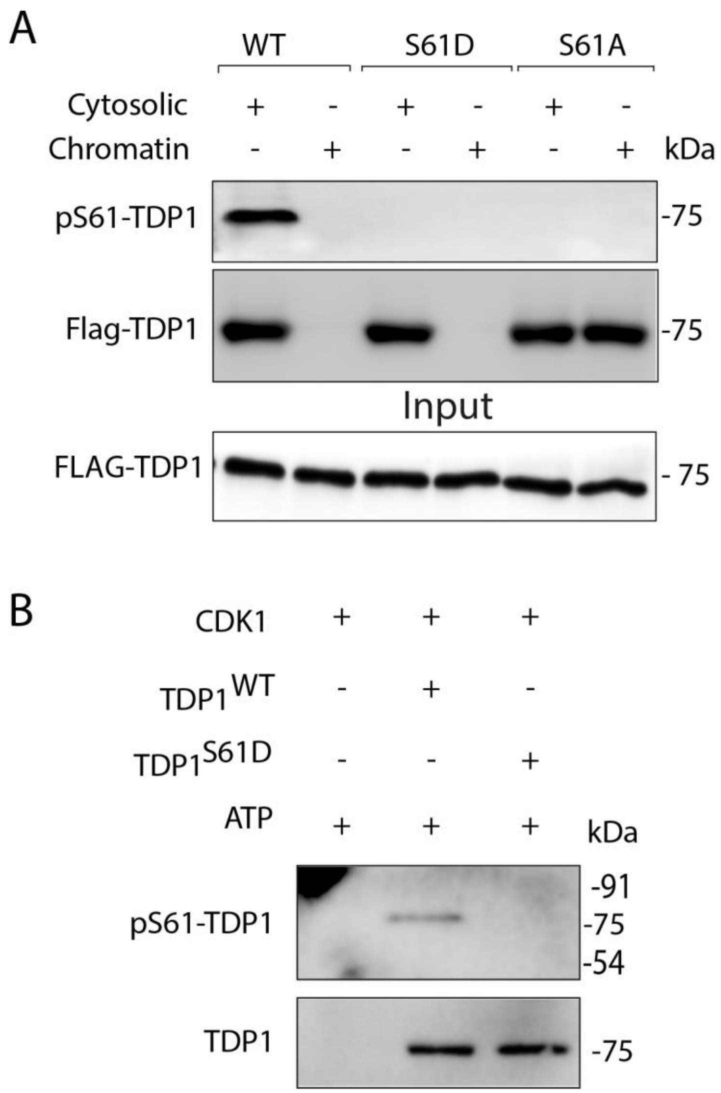

Appendix Figure S3: TDP1-pS61 is key for chromosomal dissociation of TDP1. (**A**) Chromatin fractions were prepared from TDP1$^{-/-/WT}$, TDP1$^{-/-/S61D}$ and TDP1$^{-/-/S61A}$ MEFs and analyzed by western blotting to detect FLAG-TDP1 variants in the chromatin fractions using pS61-TDP1 and anti-FLAG antibodies. Protein levels of FLAG-TDP1^{WT}, FLAG-TDP1^{S61D} and FLAG-TDP1^{S61A} were analyzed in whole cell lysates (WCE) to ensure equal levels of protein before chromatin fractionation. **(B)** *In vitro* kinase assays with HA-CDK1 immunoprecipitated from MCF7 cells in the presence of ATP. The substrates were recombinant 6xHis-tagged TDP1 variants (WT or S61D). Western blotting against the anti-TDP1 antibody shows the amount of substrate in each reaction. Protein molecular weight markers (kDa) are indicated on the right.

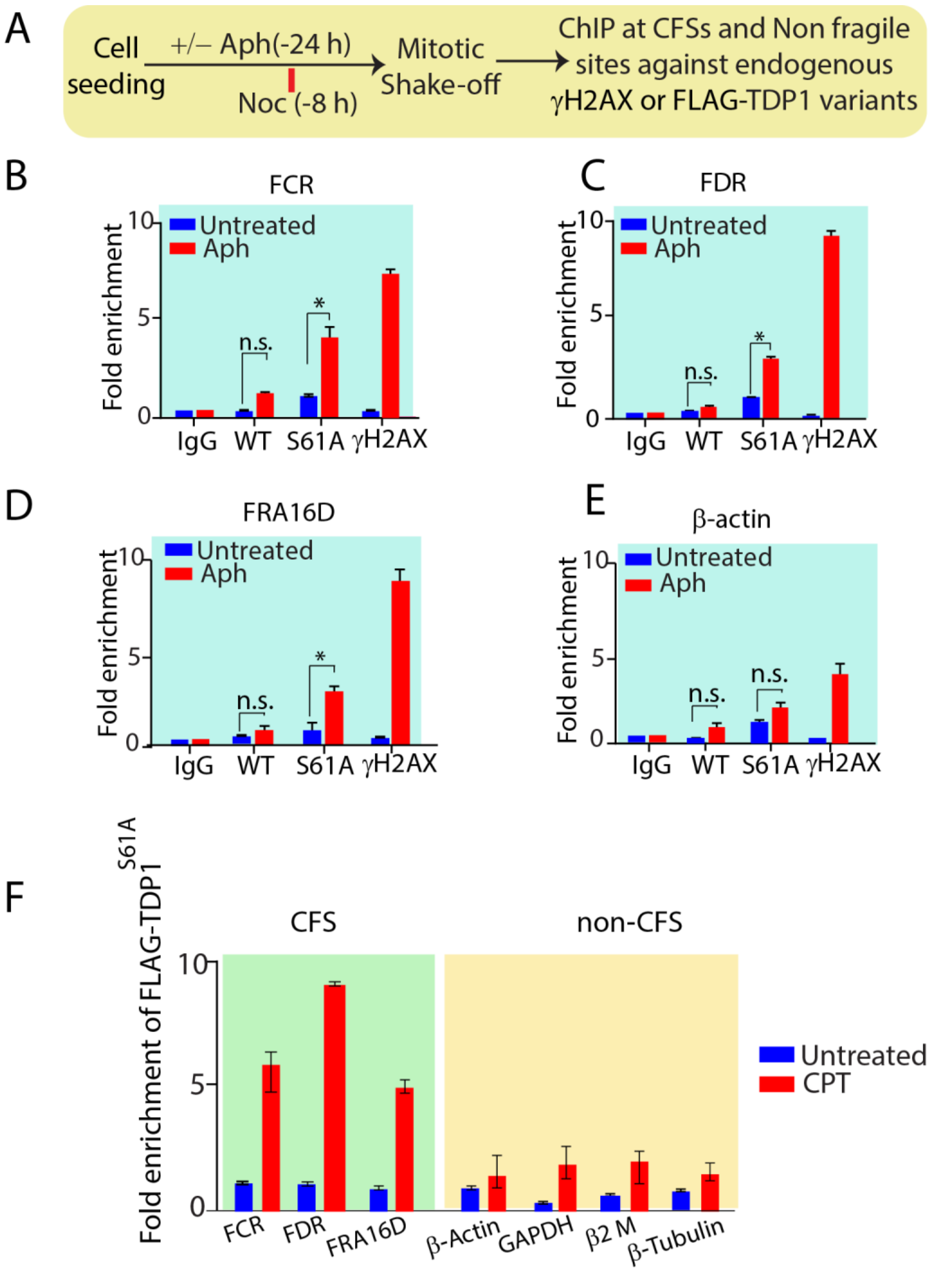

Appendix Figure S4: APH-induced TDP1^{S61A} enrichment at the CFS loci **during mitosis.** (**A**) Schematic representation of the protocol followed for the chromatin immunoprecipitation (ChIP) at the CFSs or non-CFSs. (**B-E**) Quantification of cross-linked FRA3B-FCR, FRA3B-FDR, FRA16D and β–actin loci chromatin-immunoprecipitated from MCF7 cells transfected with FLAG-TDP1 (WT or S61A) and treated with or without aphidicolin (0.4 μM, 24 h) using

the anti-FLAG antibody. The γH2AX antibody was used as a positive control for FRA3B and FRA16D enrichment post APH treatment. Fold enrichment over goat IgG was determined and is shown for each primer pair for the ChIP. All the results are expressed as mean ± SD for at least three independent experiments. n.s., non-significant (P>0.05); *P<0.05 (ANOVA). (**F**) Comparative analysis for the fold enrichment of FLAG-TDP1^{S61A} in CFSs and non-CFSs in presence of CPT. All the results are expressed as mean ± SD for at least three independent experiments.

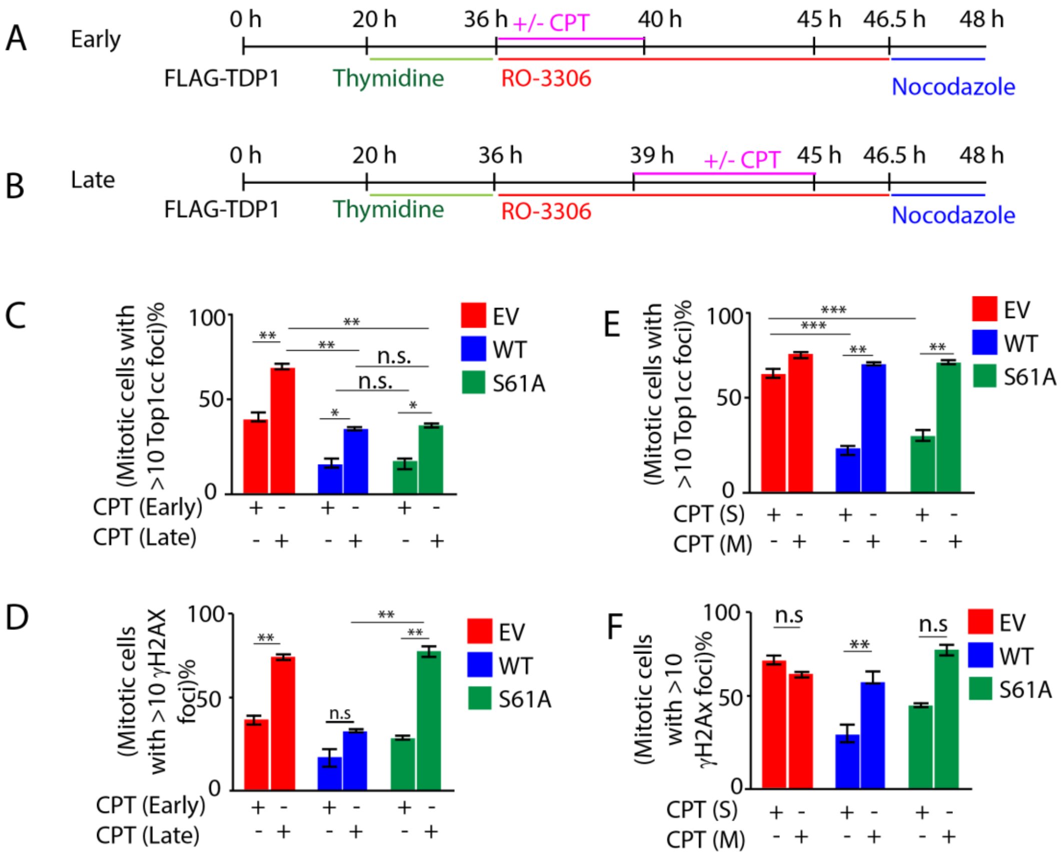

Appendix Figure S5: TDP1^{S61A} trapping generates mitotic DNA breaks independent of Top1ccs. (**A- B**) Schematic for the protocol followed to study the repair of Top1ccs induced by CPT treatment in early and late S-phases (**C-D**) Bar diagram showing the quantifications of percentages of mitotic nuclei with >10 Top1ccs and >10 γH2AX upon treatment with 15 nM CPT following protocols detailed in schemes: early CPT (A) and late CPT (B), respectively

calculated for 20–25 cells (mean ± S.E.M.). Asterisks denote statistically significant differences (*P < 0.01, **P < 0.001, ***P < 0.0001; one-way ANOVA). (**E-F**) Bar diagram showing the quantifications of percentages of mitotic nuclei with >10 Top1ccs and >10 γH2AX upon CPT treatment during S or M phases calculated for 20–25 cells (mean ± S.E.M.). n.s.; non-significant (P>0.05); *P < 0.05; **P < 0.001; ***P < 0.0001 (one-way ANOVA).

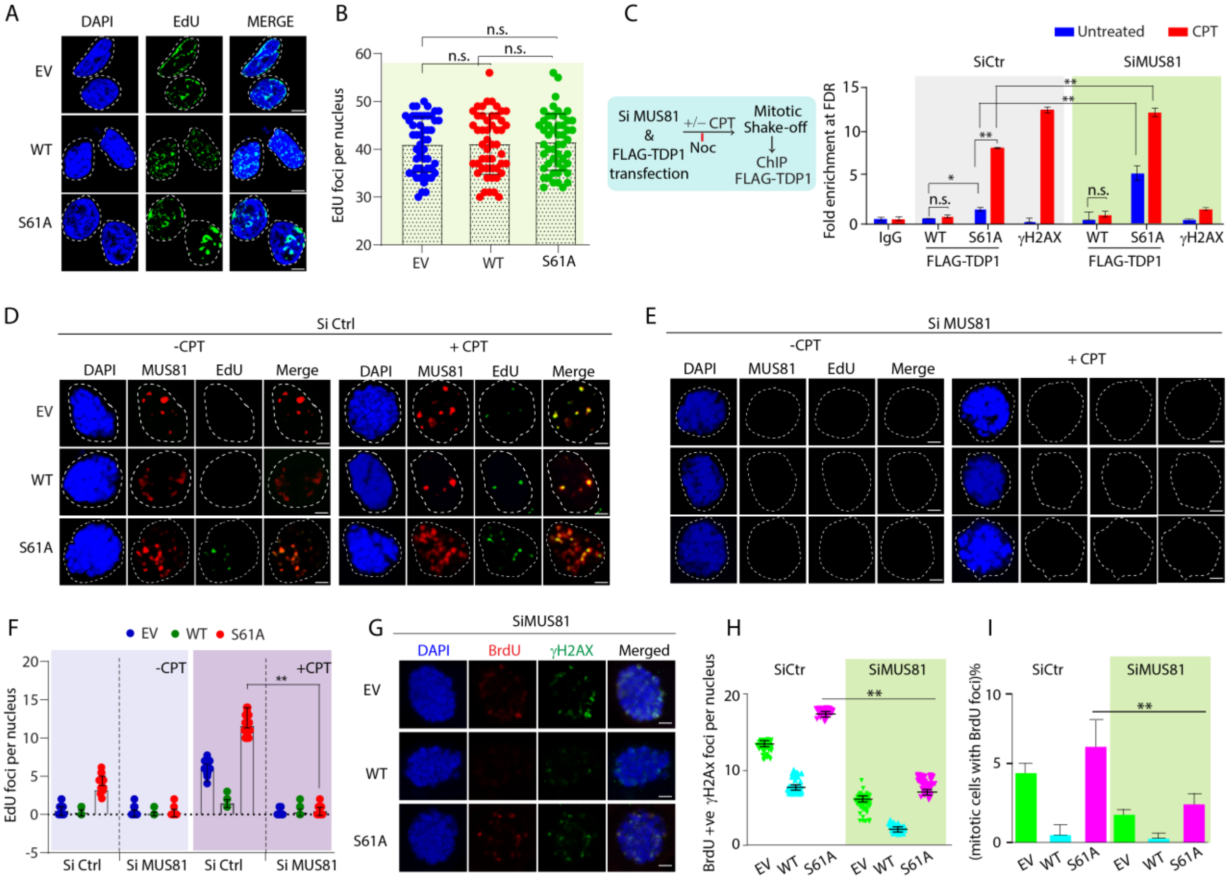

Appendix Figure S6: MUS81 knockdown results in elevated TDP1^{S61} trapping and reduces MiDAS related mitotic DNA breaks. (A) TDP1-/- MEFs complemented with EV or FLAG-TDP1 variants (TDP1$^{-/-/WT}$ and TDP1$^{-/-/S61A}$) pulsed with EdU to study replication. Representative images show newly synthesized DNA marked by EdU foci (green). Cells were counterstained with DAPI to visualize nuclei (blue). (**B**) Quantification of EdU foci per nucleus obtained from immunofluorescence were calculated for 20–25 cells. Data are mean ± SD for 3 independent experiments. Data are mean ± SD, n = 3 biological replicates. ns, non-significant (P > 0.05) (one-way ANOVA). (**C**) Scheme of the protocol followed for ChIP of FLAG-TDP1

variants (WT and S61A) at FRA3B-FDR under MUS81 proficient and knock down conditions (left). Quantification of cross-linked FRA3B-FDR loci chromatin-immunoprecipitated from MUS81 proficient (left) and MUS81 depleted (right) MCF7 cells ectopically expressing the FLAG-TDP1 variants (WT and S61A) using the specified antibodies with or without CPT treatment (15 nM; 24 h). The DSB marker γH2AX antibody was used as control for FRA3B-FDR, showed decreased enrichment post CPT treatment upon MUS81 depletion. Fold enrichment over IgG was determined and is shown for each primer pair for the ChIP. Data are mean ± SD, n = 3 biological replicates. ns, non-significant ($P > 0.05$); $*P \leq 0.05$; $**P \leq 0.01$ (one-way ANOVA). (**D-E**) Representative images of immunofluorescence microscopy showing induction of CPT (15 nM, 24 h)-induced EdU (green) and MUS81 (red) foci during mitosis in $TDP1^{-/-/EV}$, $TDP1^{-/-/WT}$, and $TDP1^{-/-/S61A}$ MEFs, co-transfected either with Si Ctrl or Si MUS81 to knockdown MUS81. Cells were counterstained with DAPI to visualize mitotic nuclei (blue). Note: Colocalization of CPT-induced EdU and MUS81 foci in merged images indicates MUS81 overloading amplifies MiDAS. (**F**) Quantifications of EdU foci on the mitotic chromosomes scored for 50 nuclei (each category) as depicted by the corresponding bar diagram. . Data are mean ± SD, n = 3. $**P \leq 0.01$ (one-way ANOVA). (**G**) Representative images showing the BrdU (red) and γH2AX (green) levels observed in the TDP1-/- MEFs co-transfected with Si MUS81 and FLAG-TDP1 variants (WT and S61A) or EV following replication stress with 15 nM CPT for 24 h. (**H and I**) Quantifications for the percentage of BrdU positive γH2AX per nucleus calculated for 50 cells per condition. Quantifications showing percentage of mitotic nuclei with BrdU foci calculated for 50 cells per condition. $**P \leq 0.001$ (one-way ANOVA). Scale bars 10 μm.

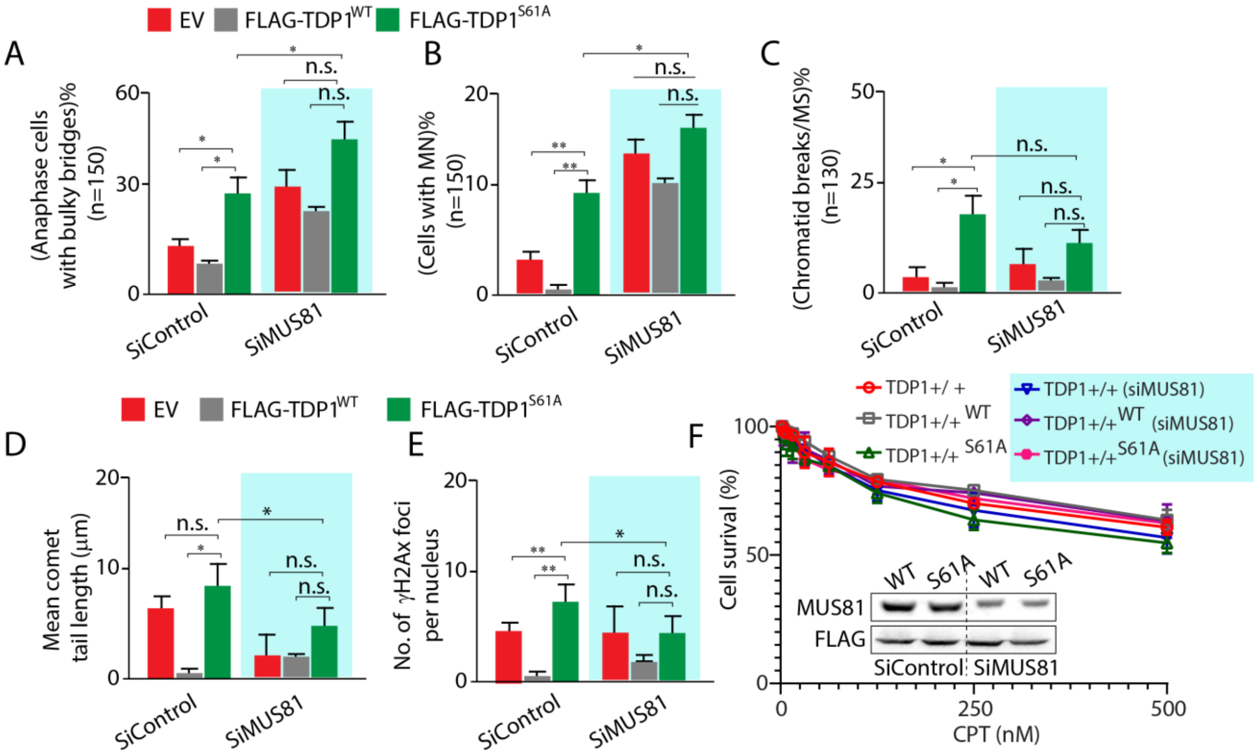

Appendix Figure S7: MUS81 knockdown in TDP1$^{+/+}$ MEFs are not sensitive to CPT. (**A-C**) Quantifications for the mitotic defects observed for TDP1$^{+/+}$ MEFs co-transfected with Si Ctrl or Si MUS81 and FLAG-TDP1 variants (WT and S61) or empty vector (EV) following replication stress with 15 nM CPT for 24 h. The percentage of such defects was scored for as indicated by the corresponding bar diagram quantification. MN, micronuclei (A); AB, anaphase bridges (B); CB, chromatid breaks (C). (**D-E**) Quantification of CPT-induced DNA strand breaks measured by neutral comet assays (D) or γH2AX staining (E) in TDP1$^{+/+}$ MEF cells transfected with Si Ctrl or Si MUS81 expressing EV or FLAG-TDP1 variants (WT or S61A) arrested in mitosis with 200 ng/mL nocodazole added during the last 8 h of CPT treatment. CPT-induced DNA strand breaks were calculated for 50 cells (mean ± S.E.M.). (**F**) Survival of TDP1$^{+/+}$ MEF cells expressing Si Ctrl or Si MUS81 and ectopically expressing empty vector (EV) FLAG-TDP1 variants (WT or S61A) in the presence of CPT. Percent survival was normalized for the CPT-untreated cells ± S.E.M. Asterisks denote statistically significant differences (*P < 0.05, **P < 0.01, ***P < 0.001; one-way ANOVA); n.s. is non-significant (P > 0.05).
