# Supplementary figures and images for: TDP1 phosphorylation by CDK1 in mitosis promotes MUS81-dependent repair of trapped Top1-DNA covalent complexes (part 1 of 3)
Source: EMBO J. 2024 Jul 16;43(17):3710–32. doi: 10.1038/s44318-024-00169-3 (PMC11377750; doi:10.1038/s44318-024-00169-3)

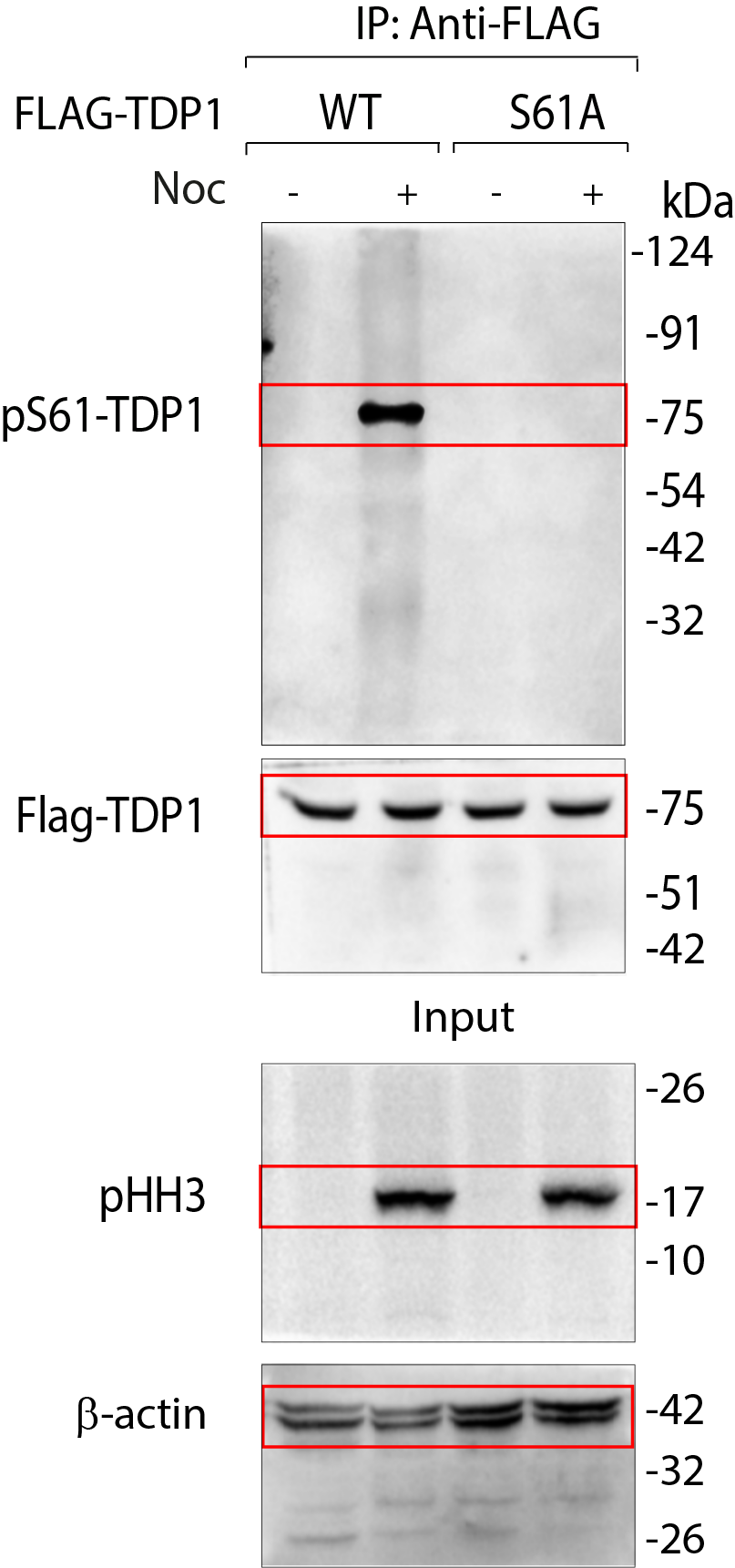

Supplement: Supplementary file 3 — Source data Fig. 1 [file 44318_2024_169_MOESM3_ESM.zip › SD_Figure_1.zip/Figure 1/1C/1C.tif]

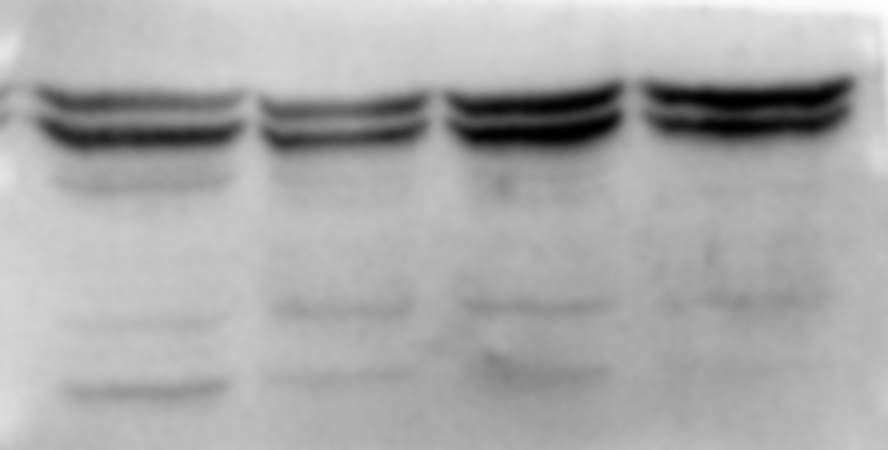

Supplement: Supplementary file 3 — Source data Fig. 1 [file 44318_2024_169_MOESM3_ESM.zip › SD_Figure_1.zip/Figure 1/1C/Fig 1C_beta-actin.tif]

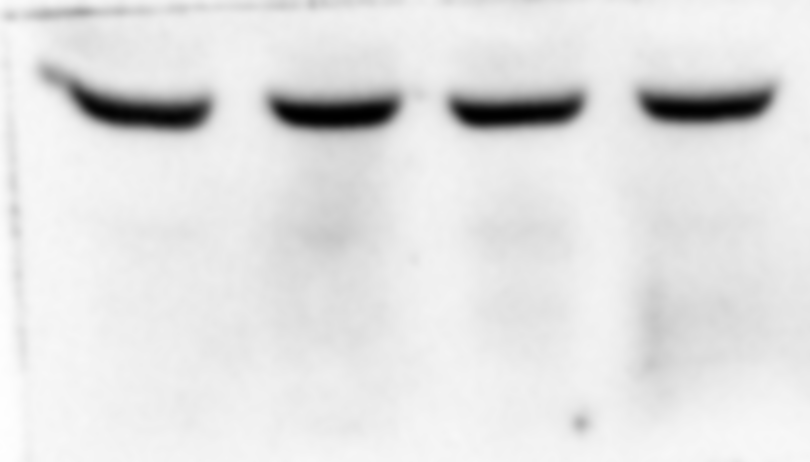

Supplement: Supplementary file 3 — Source data Fig. 1 [file 44318_2024_169_MOESM3_ESM.zip › SD_Figure_1.zip/Figure 1/1C/Fig 1C_FLAG-TDP1.tif]

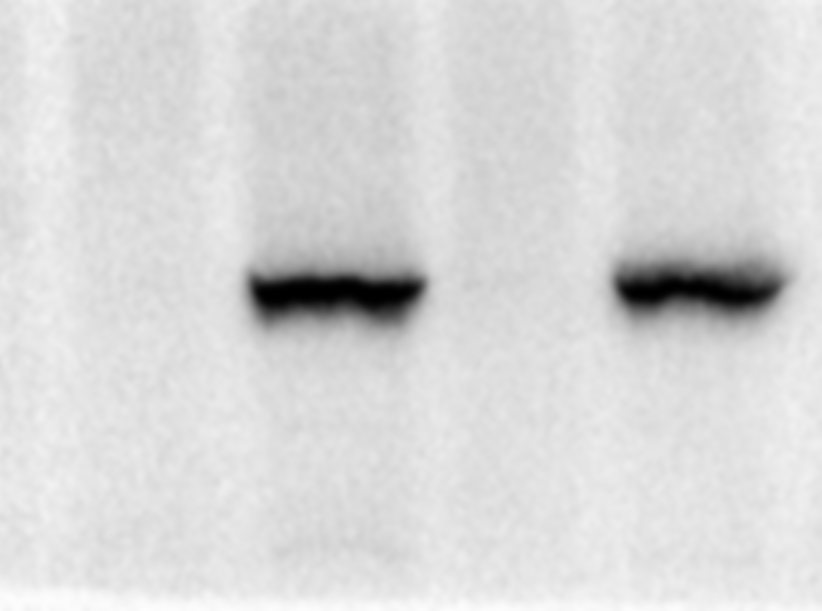

Supplement: Supplementary file 3 — Source data Fig. 1 [file 44318_2024_169_MOESM3_ESM.zip › SD_Figure_1.zip/Figure 1/1C/Fig 1C_pHH3.tif]

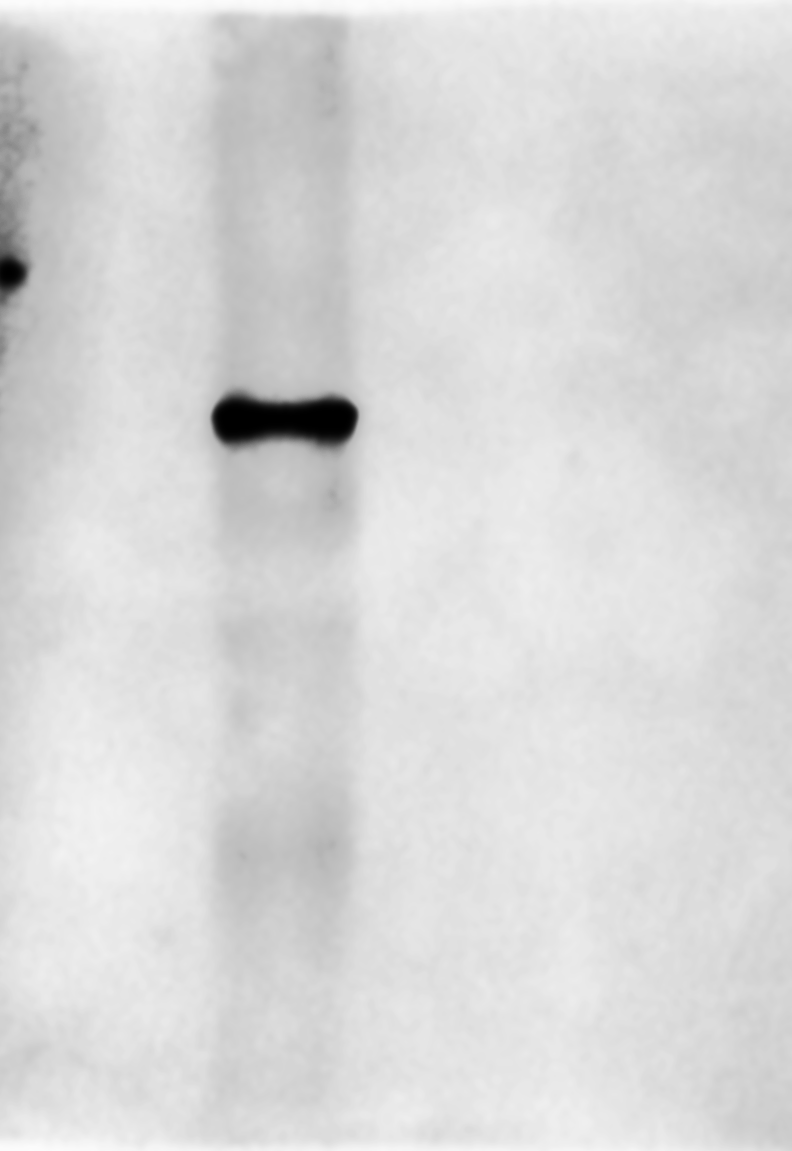

Supplement: Supplementary file 3 — Source data Fig. 1 [file 44318_2024_169_MOESM3_ESM.zip › SD_Figure_1.zip/Figure 1/1C/Fig 1C_pS61-TDP1.tif]

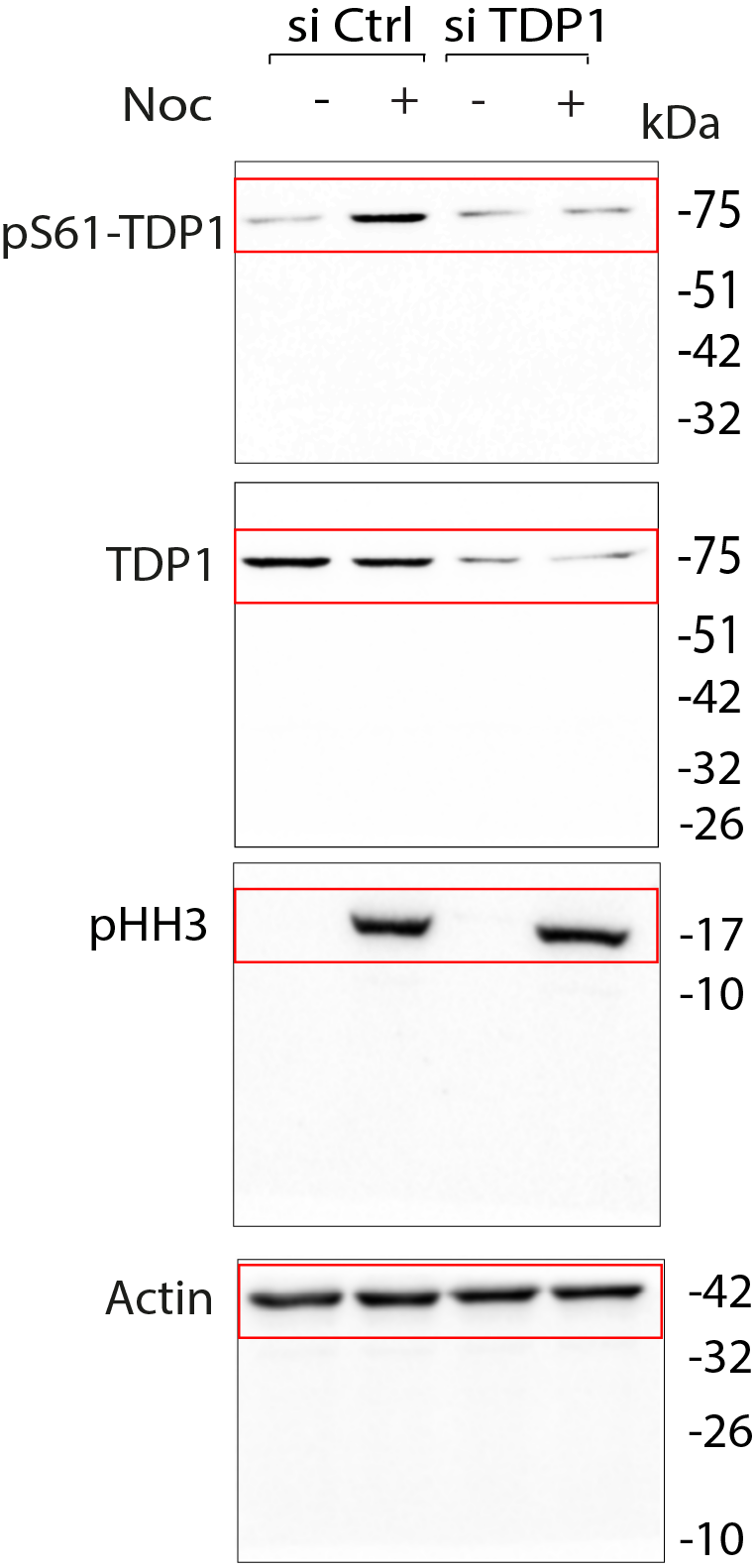

Supplement: Supplementary file 3 — Source data Fig. 1 [file 44318_2024_169_MOESM3_ESM.zip › SD_Figure_1.zip/Figure 1/1D/1D.tif]

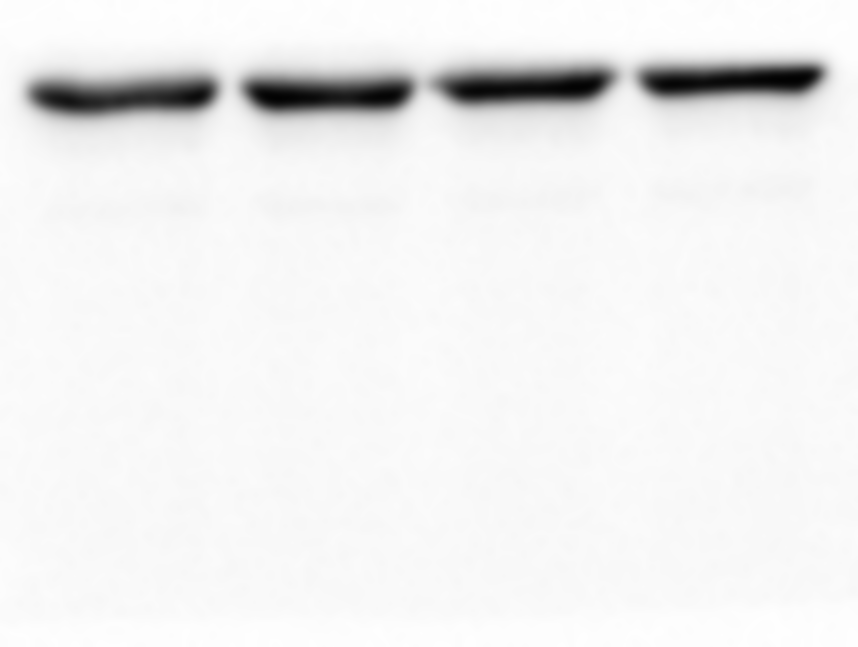

Supplement: Supplementary file 3 — Source data Fig. 1 [file 44318_2024_169_MOESM3_ESM.zip › SD_Figure_1.zip/Figure 1/1D/Fig 1D_actin.tif]

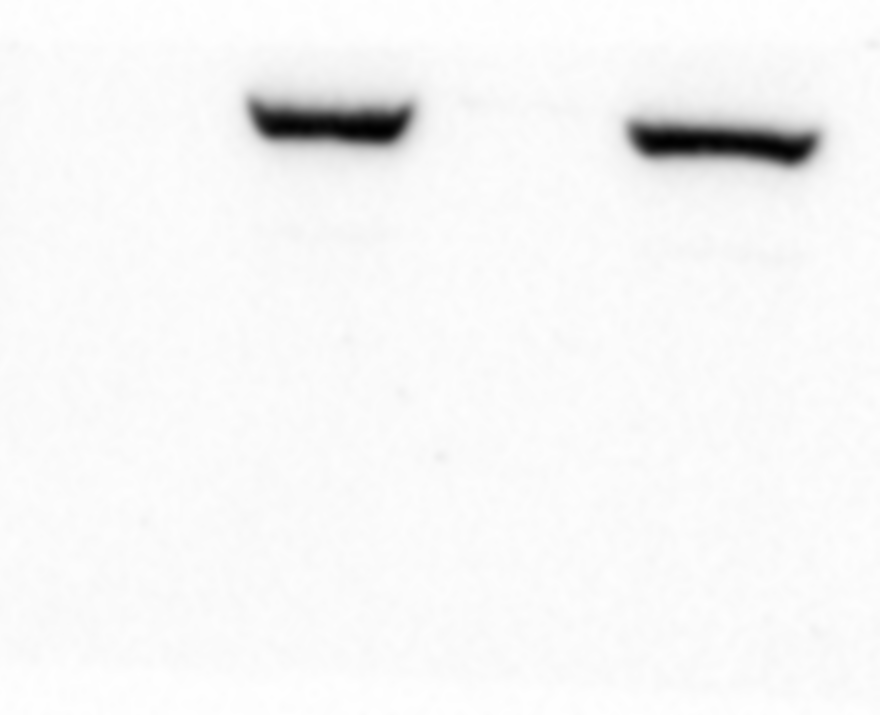

Supplement: Supplementary file 3 — Source data Fig. 1 [file 44318_2024_169_MOESM3_ESM.zip › SD_Figure_1.zip/Figure 1/1D/Fig 1D_pHH3.tif]

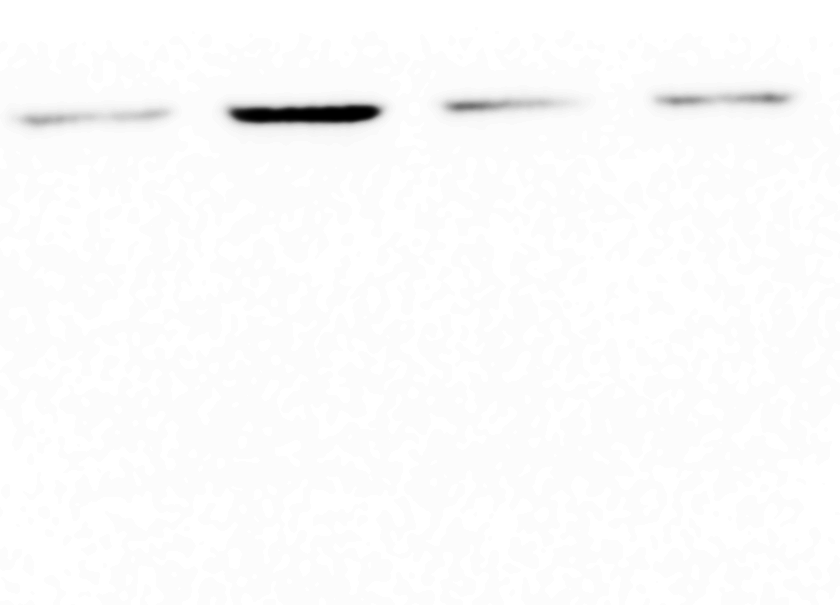

Supplement: Supplementary file 3 — Source data Fig. 1 [file 44318_2024_169_MOESM3_ESM.zip › SD_Figure_1.zip/Figure 1/1D/Fig 1D_ps61-TDP1.tif]

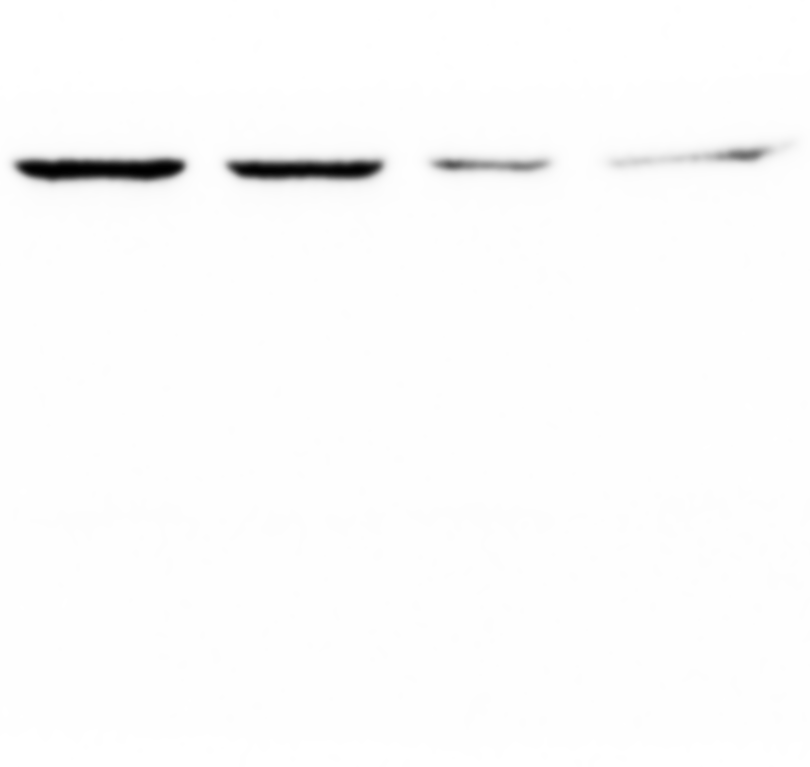

Supplement: Supplementary file 3 — Source data Fig. 1 [file 44318_2024_169_MOESM3_ESM.zip › SD_Figure_1.zip/Figure 1/1D/Fig 1D_TDP1.tif]

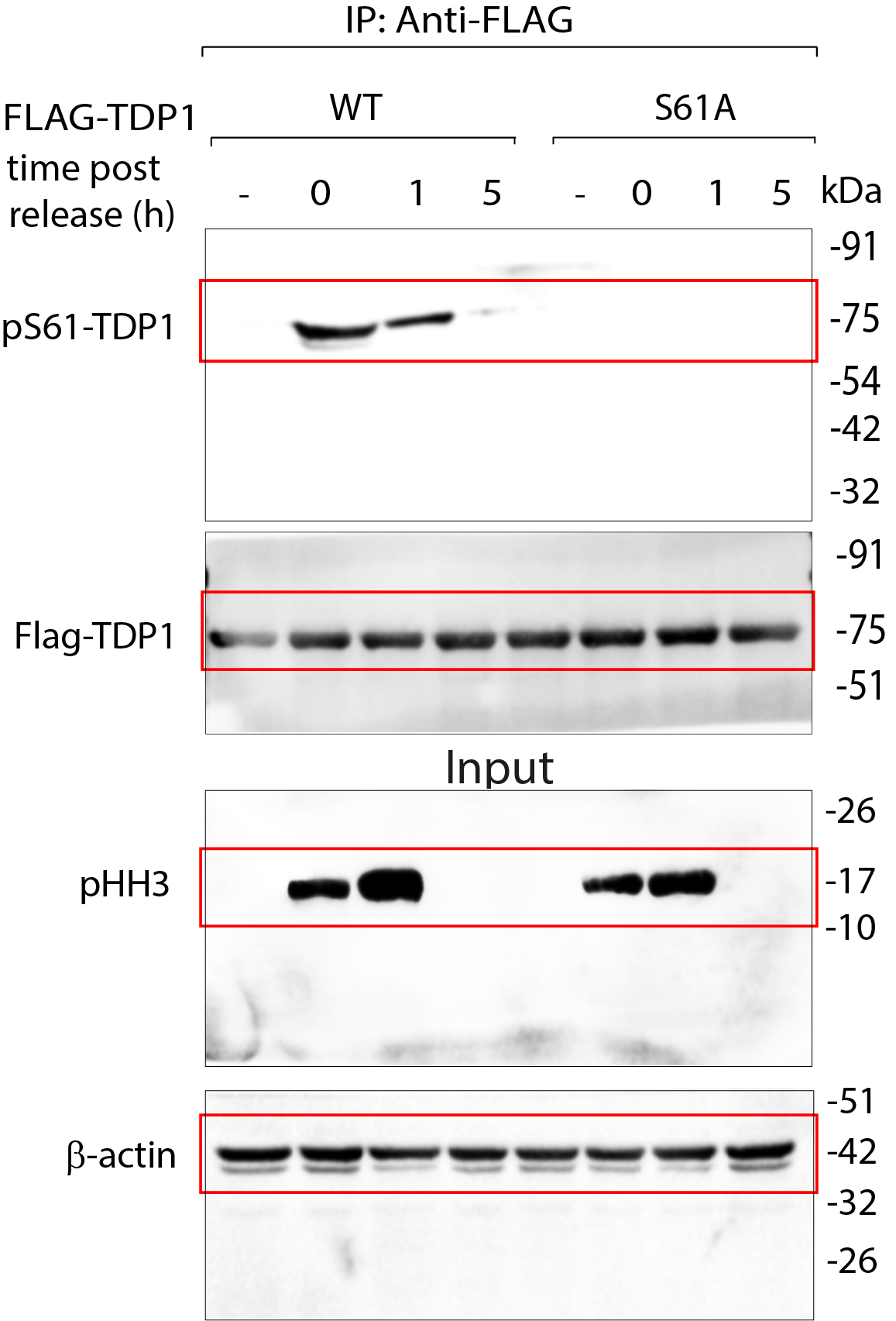

Supplement: Supplementary file 3 — Source data Fig. 1 [file 44318_2024_169_MOESM3_ESM.zip › SD_Figure_1.zip/Figure 1/1F/1F.tif]

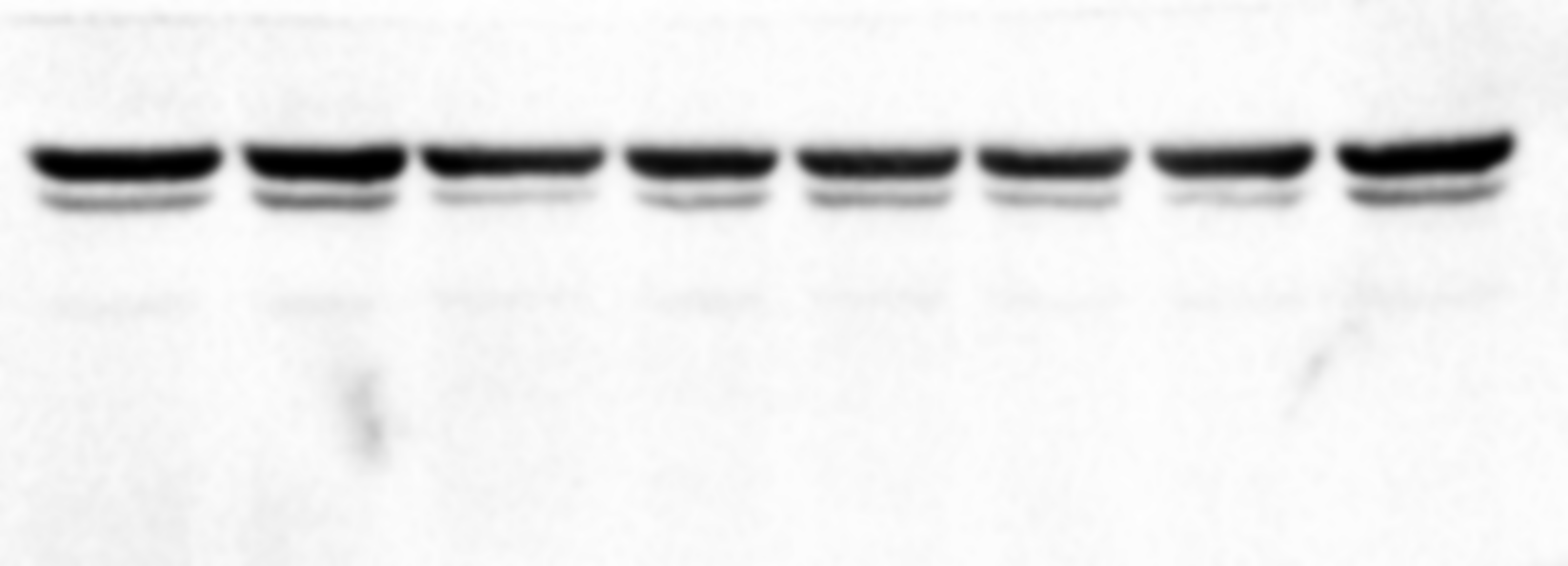

Supplement: Supplementary file 3 — Source data Fig. 1 [file 44318_2024_169_MOESM3_ESM.zip › SD_Figure_1.zip/Figure 1/1F/Fig 1F_beta actin.tif]

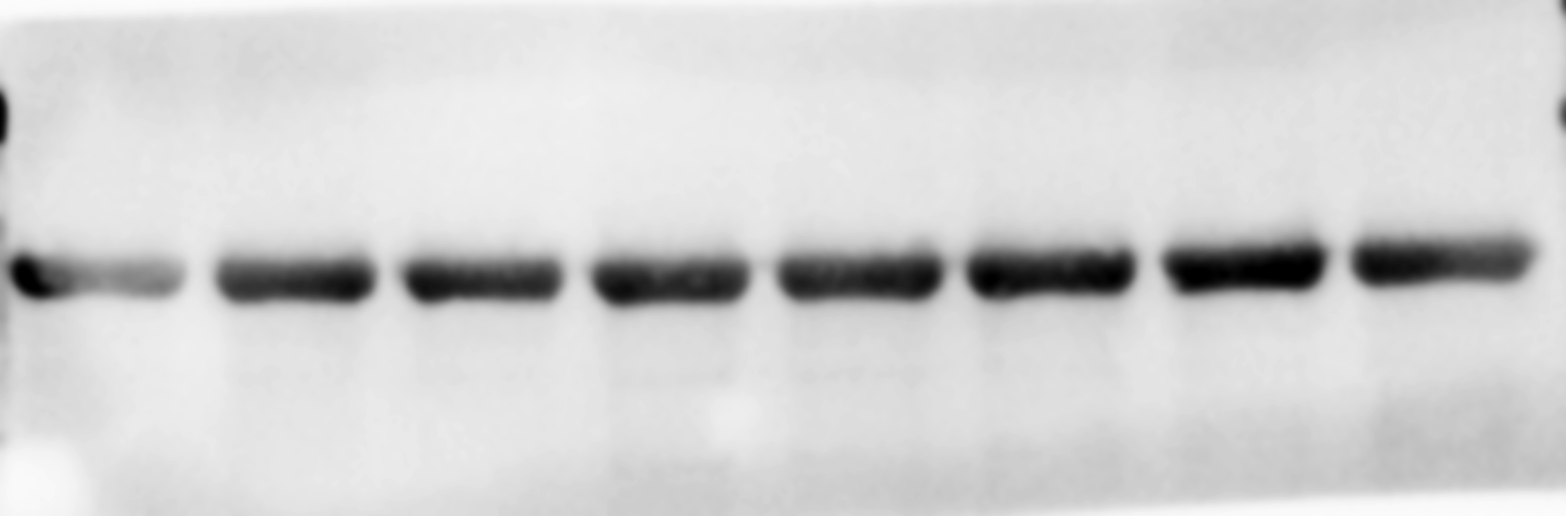

Supplement: Supplementary file 3 — Source data Fig. 1 [file 44318_2024_169_MOESM3_ESM.zip › SD_Figure_1.zip/Figure 1/1F/Fig 1F_FLAG-TDP1.tif]

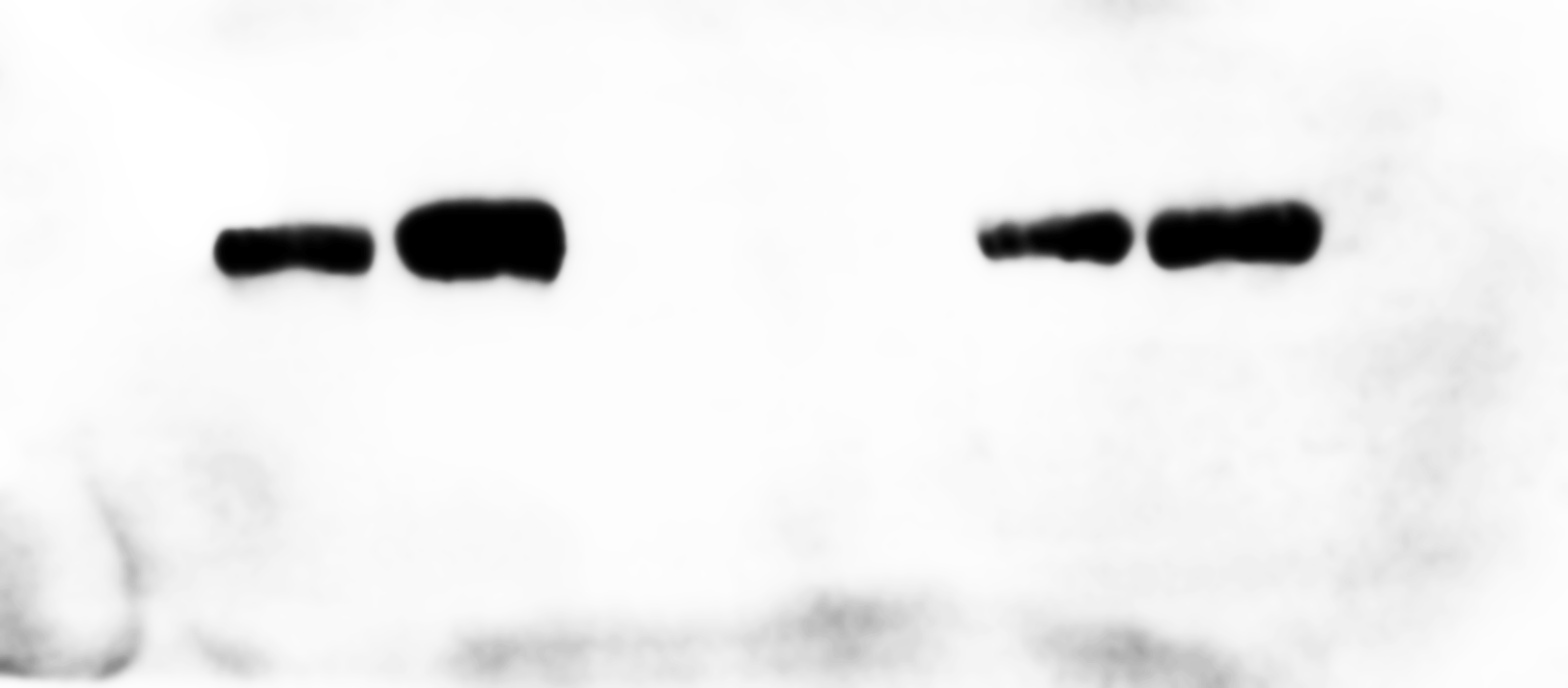

Supplement: Supplementary file 3 — Source data Fig. 1 [file 44318_2024_169_MOESM3_ESM.zip › SD_Figure_1.zip/Figure 1/1F/Fig 1F_pHH3.tif]

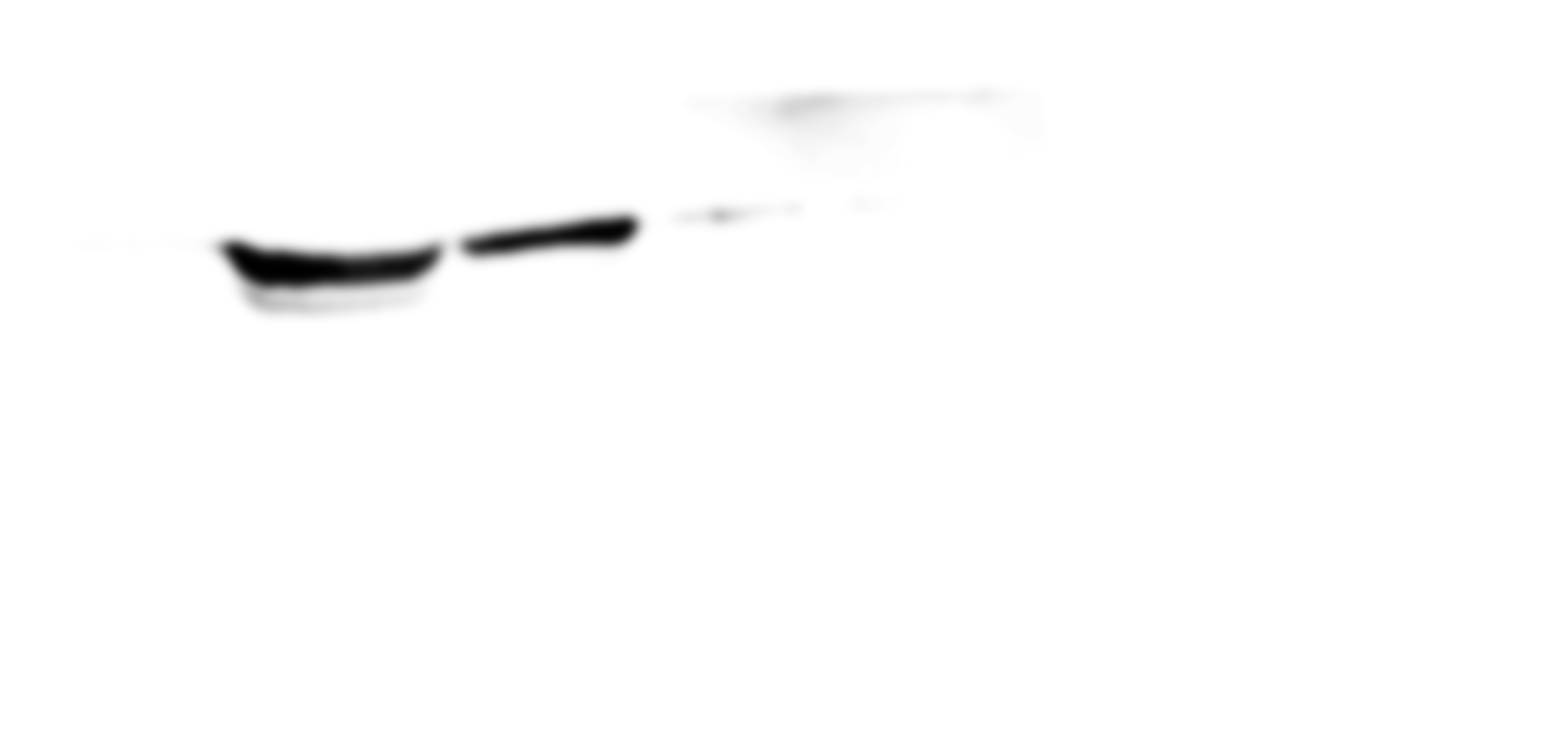

Supplement: Supplementary file 3 — Source data Fig. 1 [file 44318_2024_169_MOESM3_ESM.zip › SD_Figure_1.zip/Figure 1/1F/Fig 1F_pS61-TDP1.tif]

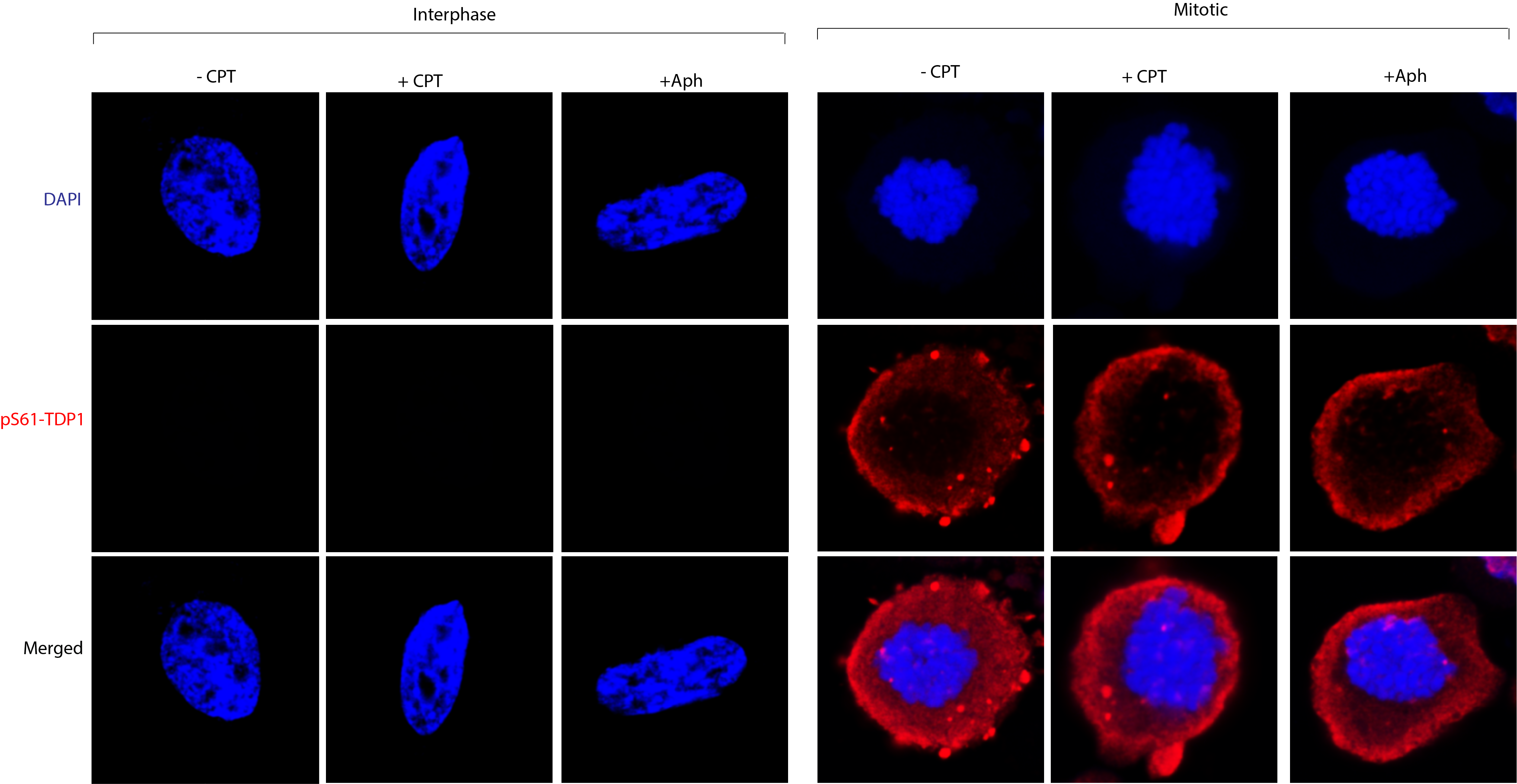

Supplement: Supplementary file 3 — Source data Fig. 1 [file 44318_2024_169_MOESM3_ESM.zip › SD_Figure_1.zip/Figure 1/1G/1G.tif]

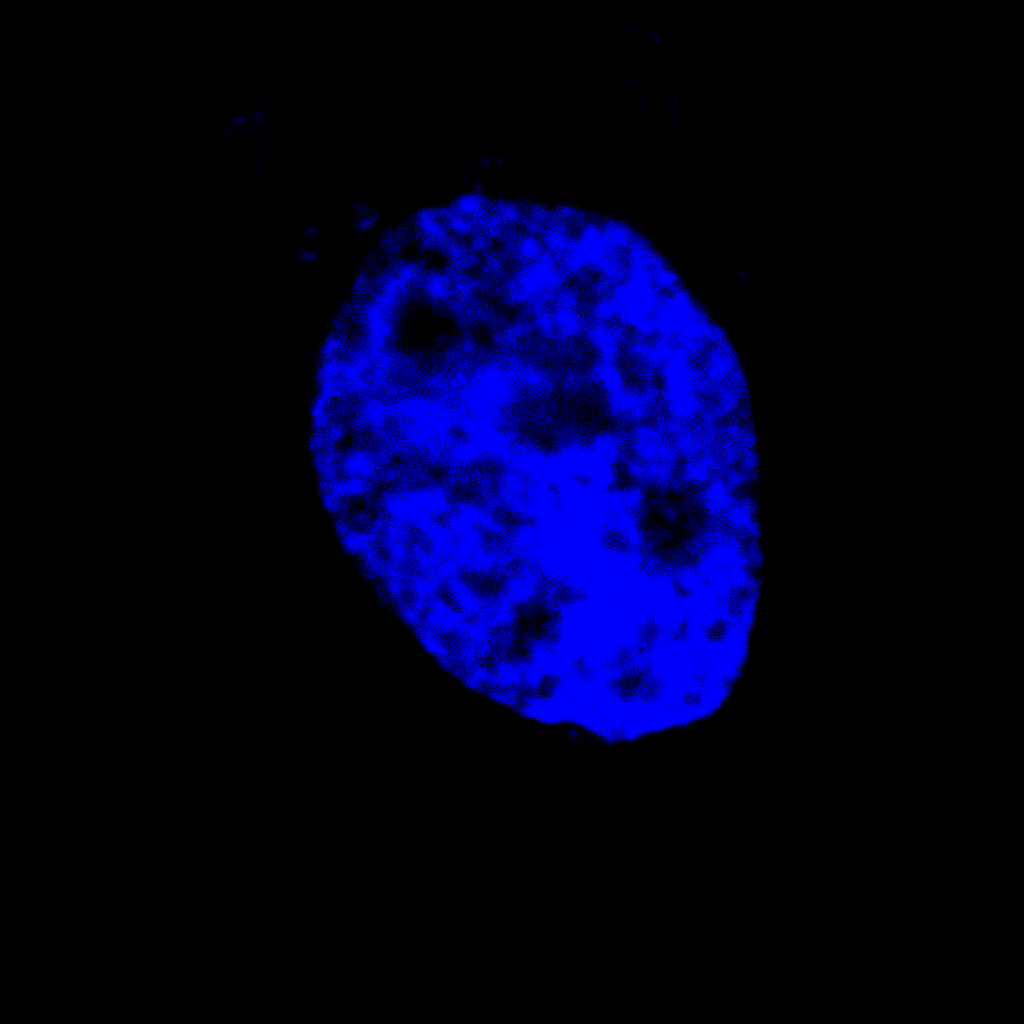

Supplement: Supplementary file 3 — Source data Fig. 1 [file 44318_2024_169_MOESM3_ESM.zip › SD_Figure_1.zip/Figure 1/1G/Control_Series005.tif]

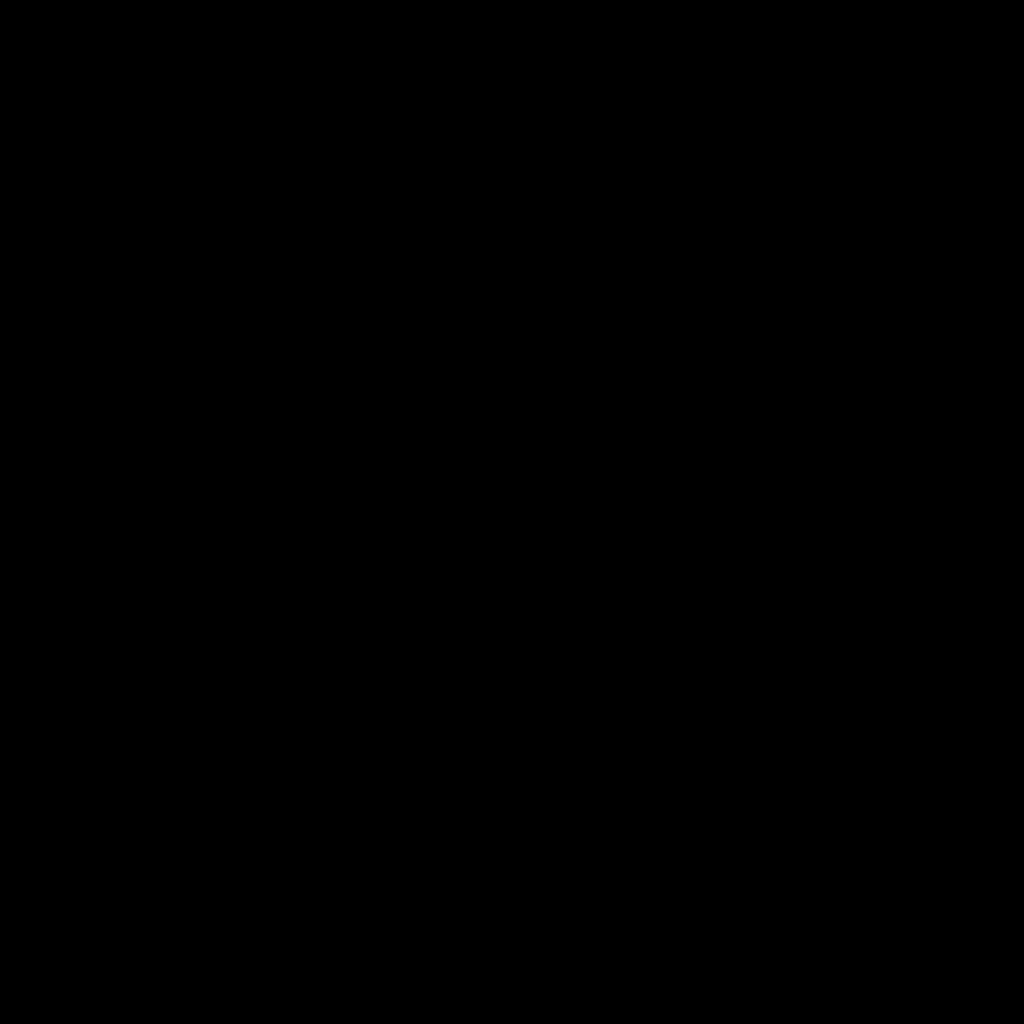

Supplement: Supplementary file 3 — Source data Fig. 1 [file 44318_2024_169_MOESM3_ESM.zip › SD_Figure_1.zip/Figure 1/1G/Control_Series005_ch01.tif]

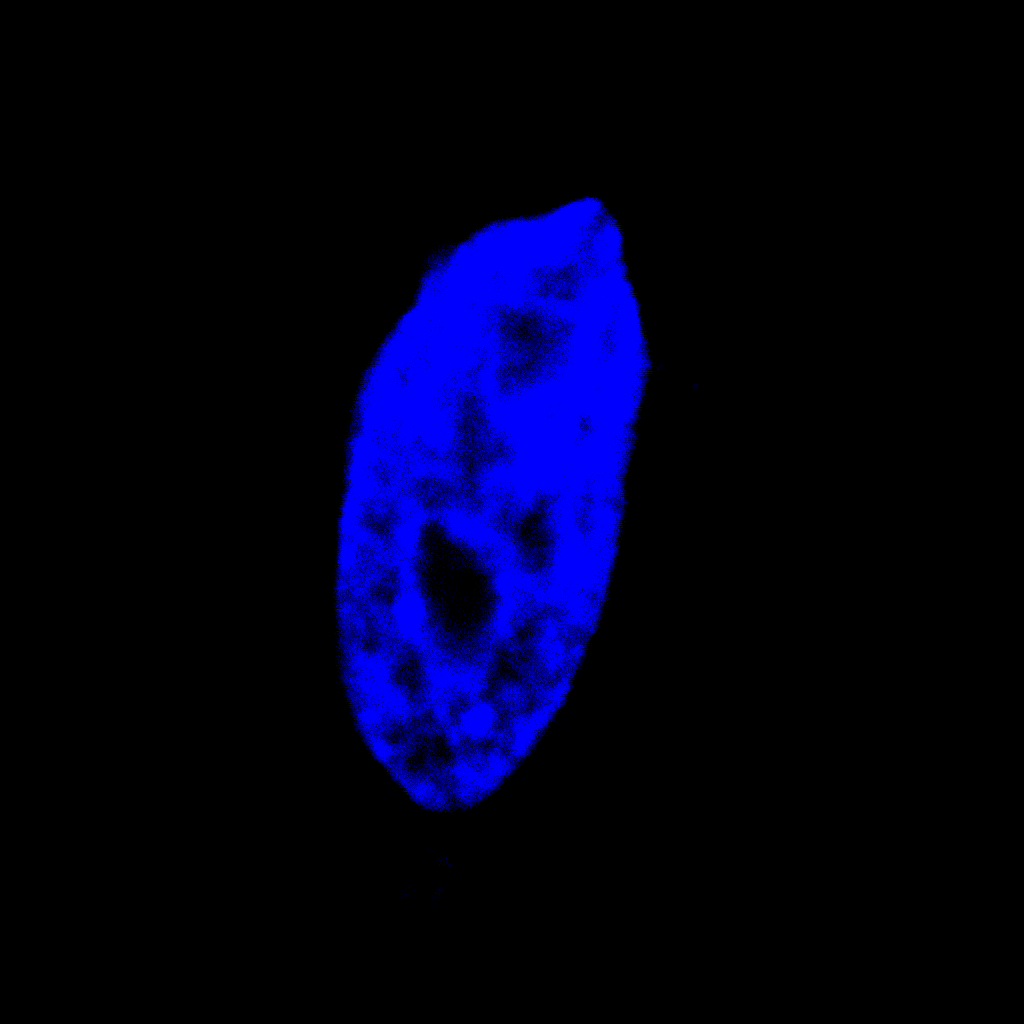

Supplement: Supplementary file 3 — Source data Fig. 1 [file 44318_2024_169_MOESM3_ESM.zip › SD_Figure_1.zip/Figure 1/1G/Control_Series006.tif]

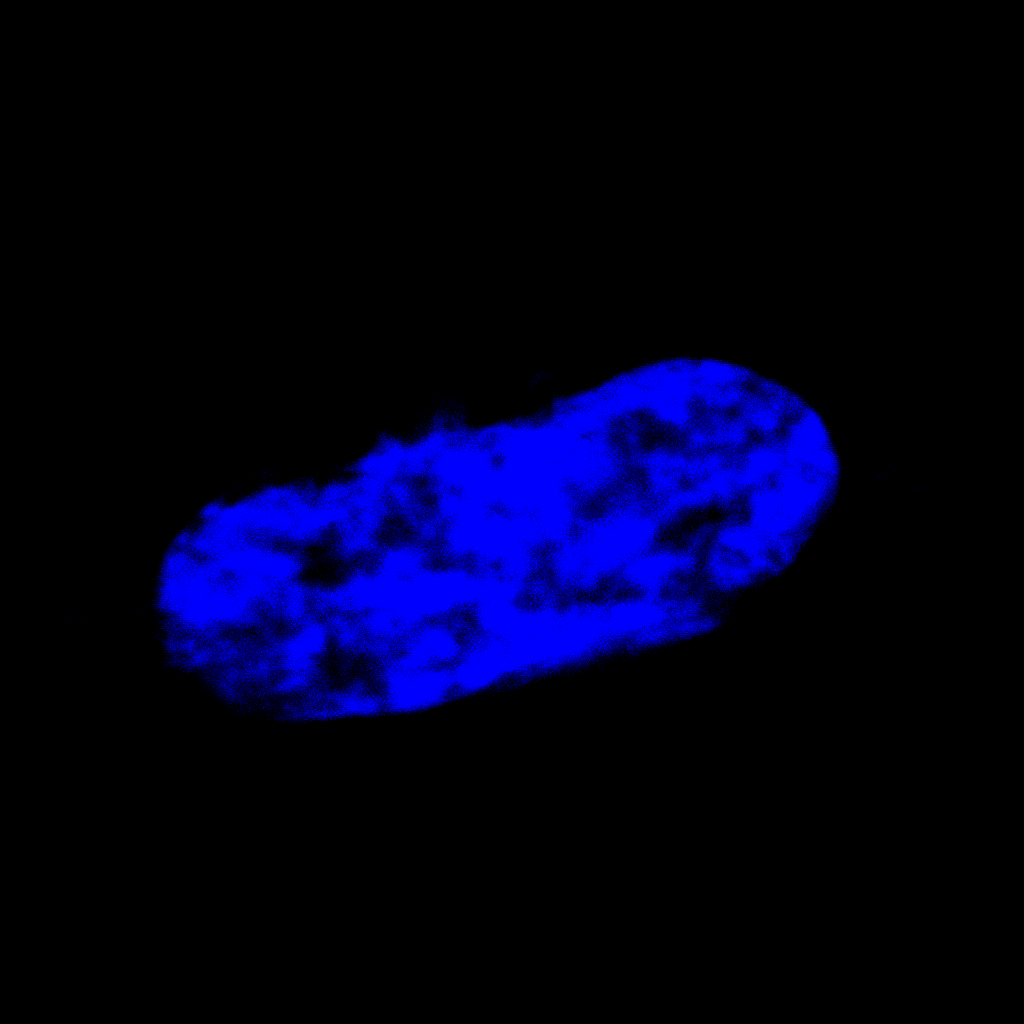

Supplement: Supplementary file 3 — Source data Fig. 1 [file 44318_2024_169_MOESM3_ESM.zip › SD_Figure_1.zip/Figure 1/1G/Control_Series007.tif]

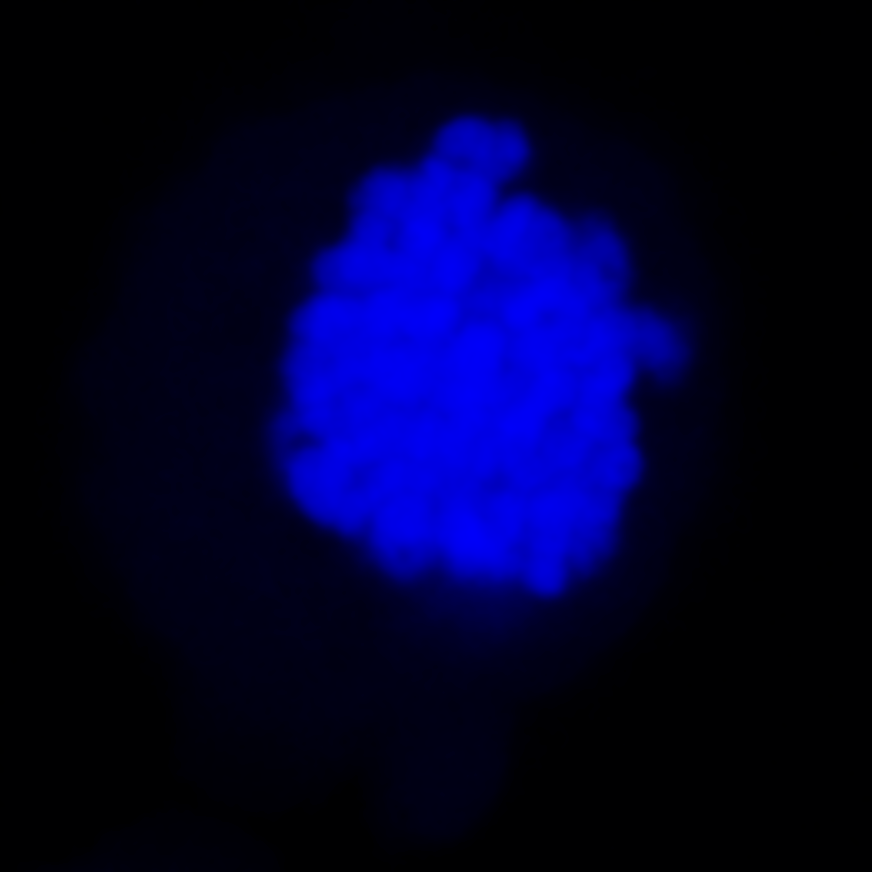

Supplement: Supplementary file 3 — Source data Fig. 1 [file 44318_2024_169_MOESM3_ESM.zip › SD_Figure_1.zip/Figure 1/1G/Fig 1G_pS61TDP1_+CPT blue.tif]

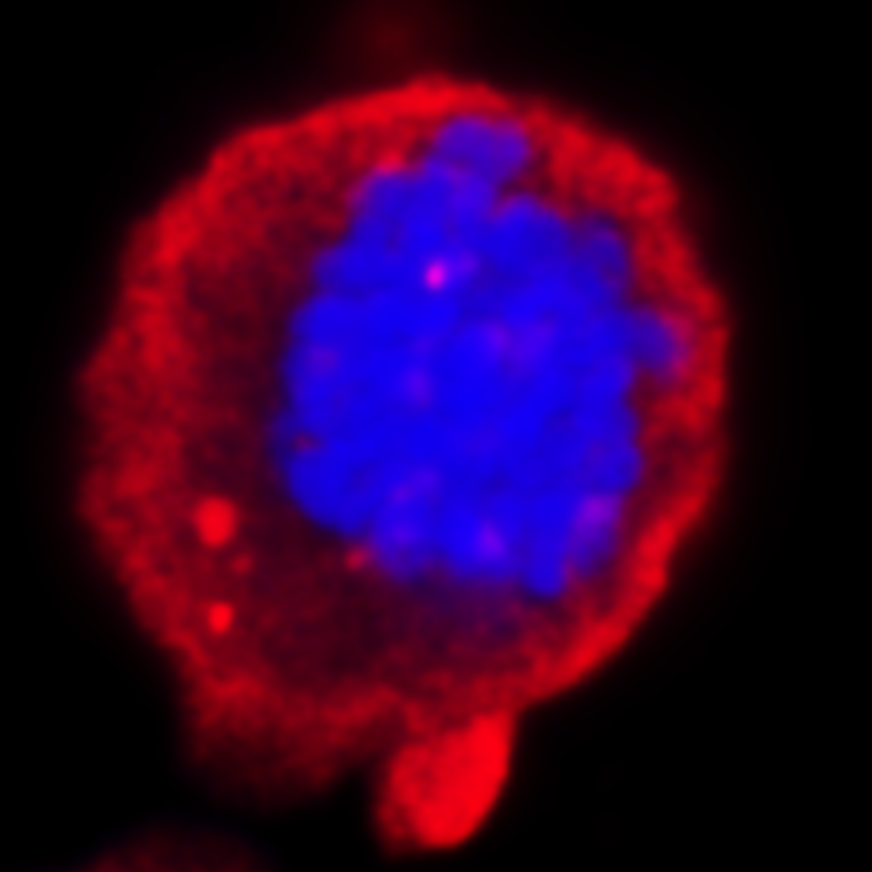

Supplement: Supplementary file 3 — Source data Fig. 1 [file 44318_2024_169_MOESM3_ESM.zip › SD_Figure_1.zip/Figure 1/1G/Fig 1G_pS61TDP1_+CPT merged.tif]

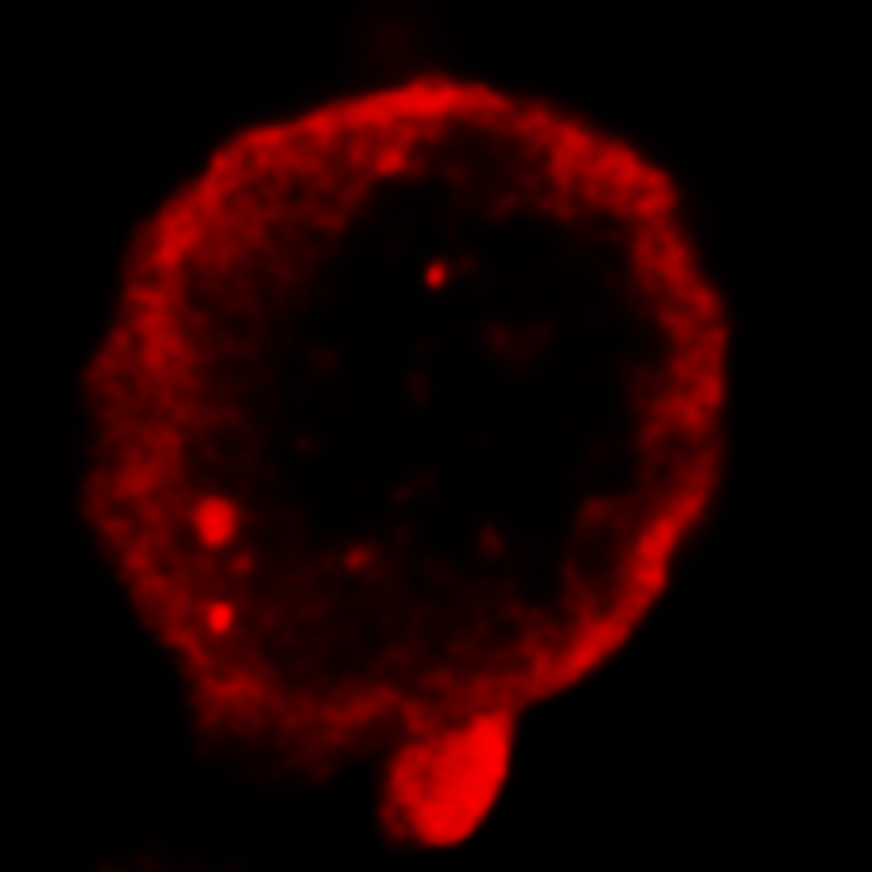

Supplement: Supplementary file 3 — Source data Fig. 1 [file 44318_2024_169_MOESM3_ESM.zip › SD_Figure_1.zip/Figure 1/1G/Fig 1G_pS61TDP1_+CPT red.tif]

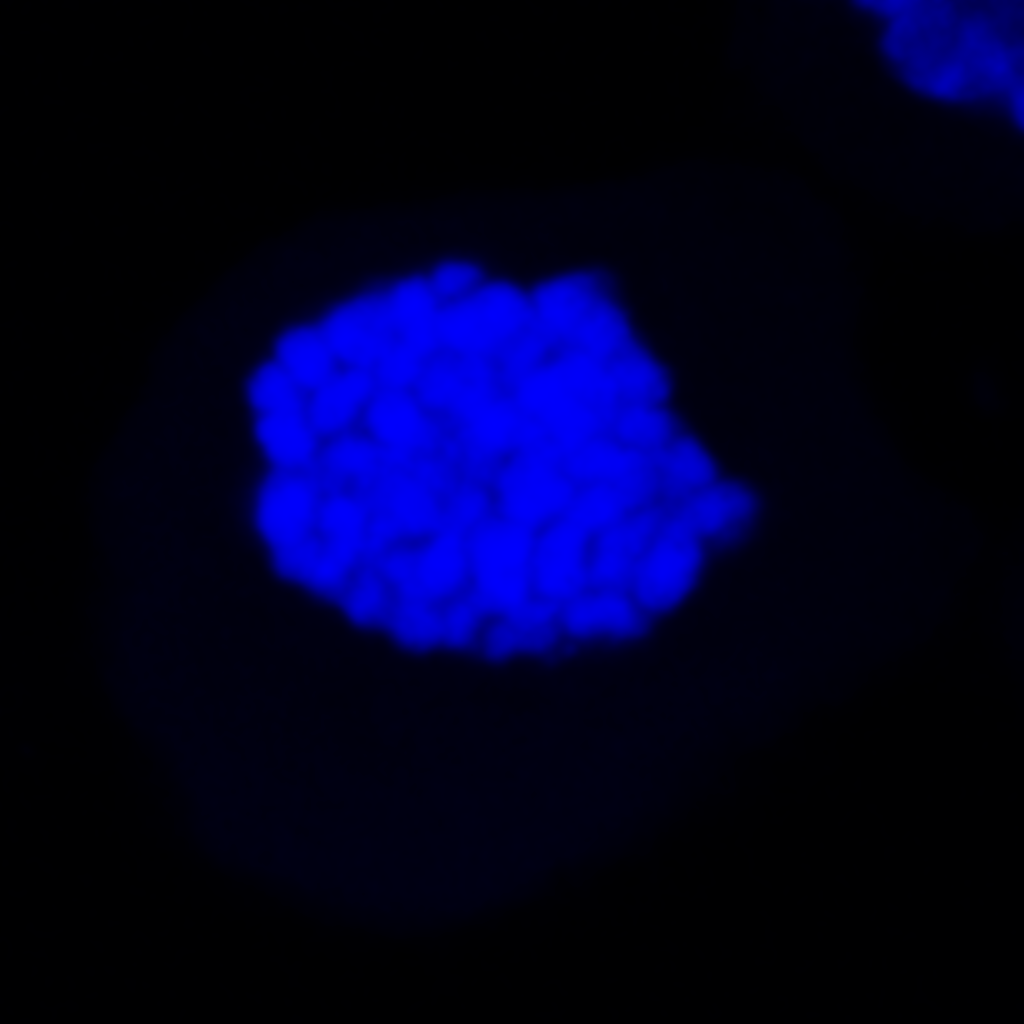

Supplement: Supplementary file 3 — Source data Fig. 1 [file 44318_2024_169_MOESM3_ESM.zip › SD_Figure_1.zip/Figure 1/1G/Fig 1G_pS61TDP1_Aph blue.tif]

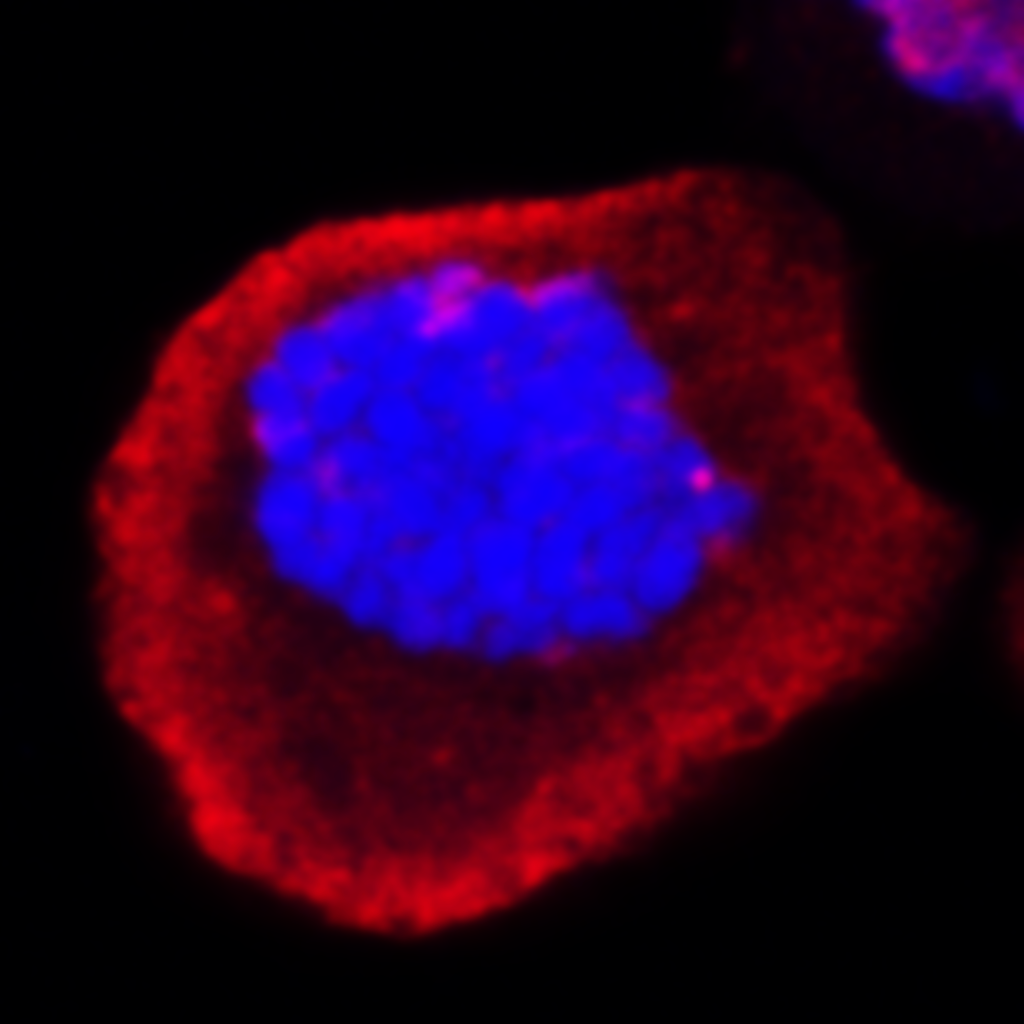

Supplement: Supplementary file 3 — Source data Fig. 1 [file 44318_2024_169_MOESM3_ESM.zip › SD_Figure_1.zip/Figure 1/1G/Fig 1G_pS61TDP1_Aph merged.tif]

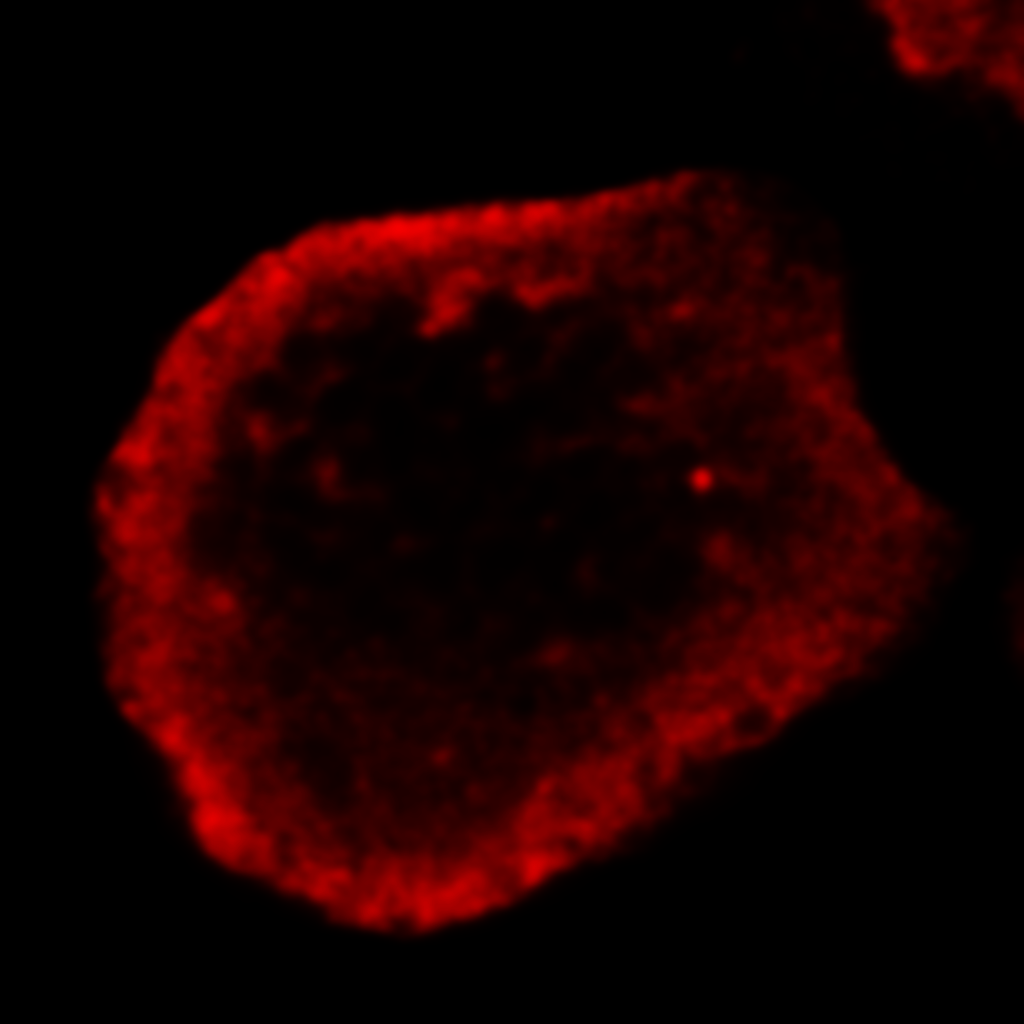

Supplement: Supplementary file 3 — Source data Fig. 1 [file 44318_2024_169_MOESM3_ESM.zip › SD_Figure_1.zip/Figure 1/1G/Fig 1G_pS61TDP1_Aph red.tif]

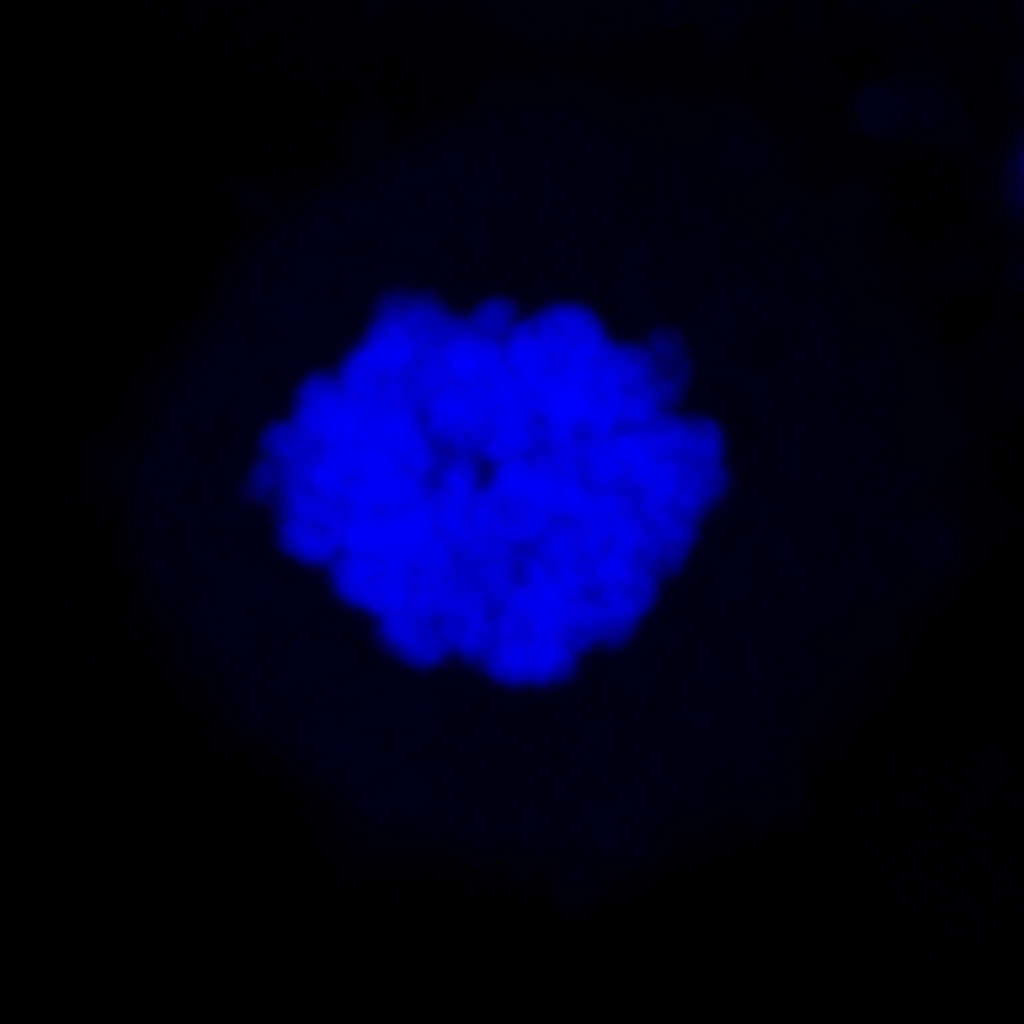

Supplement: Supplementary file 3 — Source data Fig. 1 [file 44318_2024_169_MOESM3_ESM.zip › SD_Figure_1.zip/Figure 1/1G/Fig 1G_pS61TDP1_-CPT blue.tif]

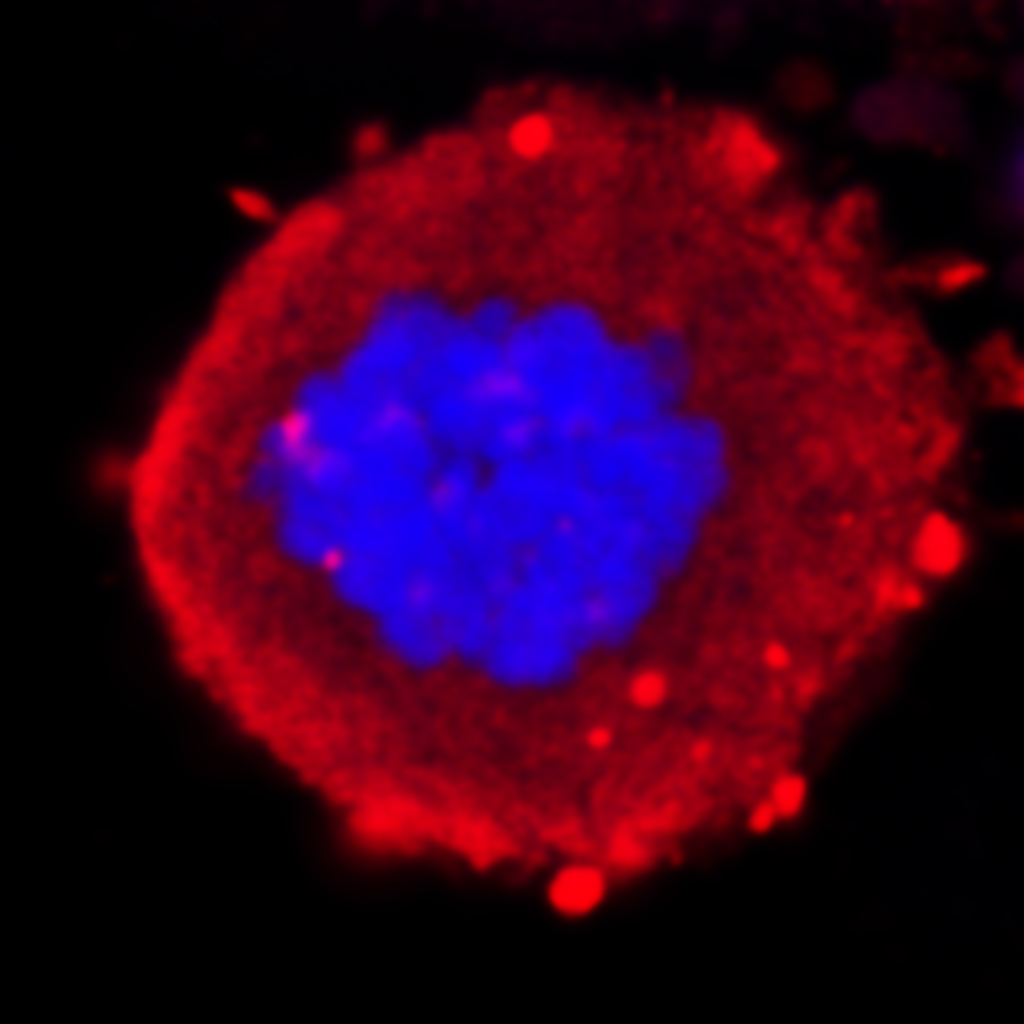

Supplement: Supplementary file 3 — Source data Fig. 1 [file 44318_2024_169_MOESM3_ESM.zip › SD_Figure_1.zip/Figure 1/1G/Fig 1G_pS61TDP1_-CPT merged.tif]

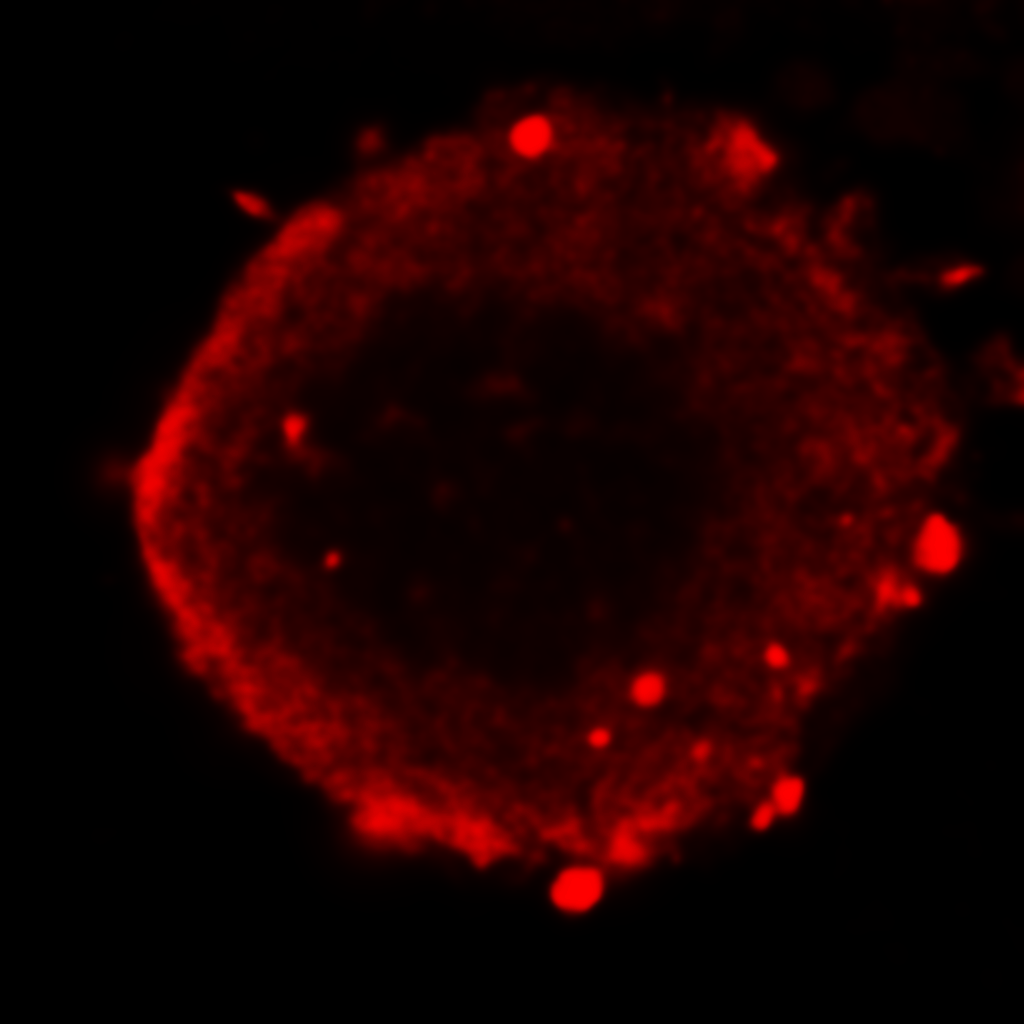

Supplement: Supplementary file 3 — Source data Fig. 1 [file 44318_2024_169_MOESM3_ESM.zip › SD_Figure_1.zip/Figure 1/1G/Fig 1G_pS61TDP1_-CPT red.tif]

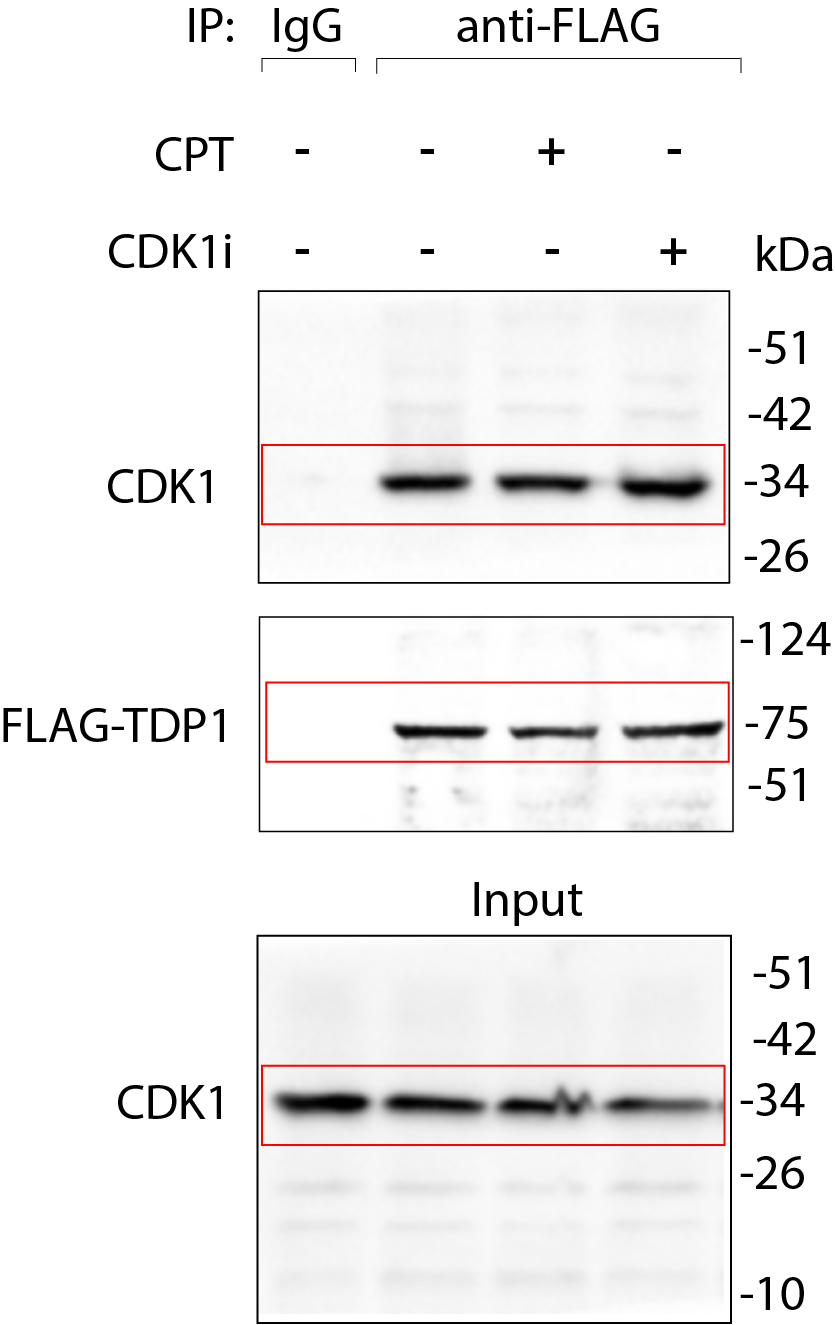

Supplement: Supplementary file 4 — Source data Fig. 2 [file 44318_2024_169_MOESM4_ESM.zip › SD_Figure_2.zip/Figure 2/2A/Fig 2A.tif]

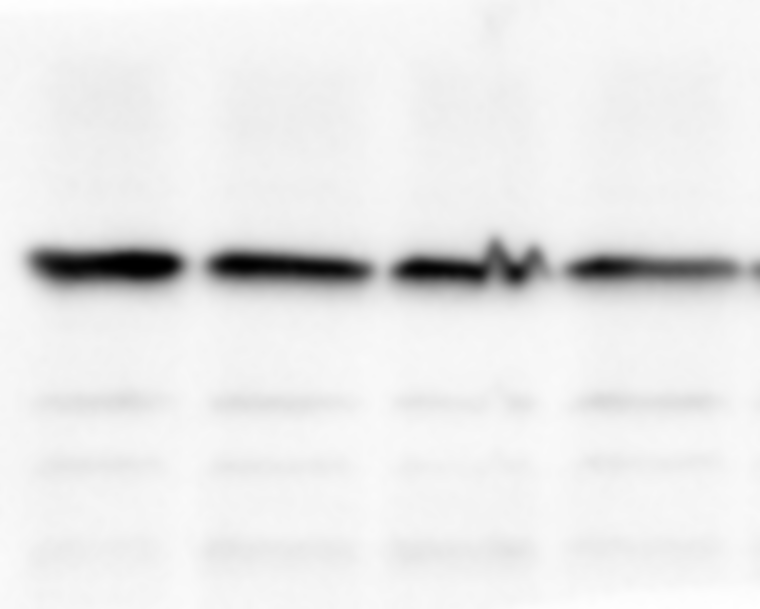

Supplement: Supplementary file 4 — Source data Fig. 2 [file 44318_2024_169_MOESM4_ESM.zip › SD_Figure_2.zip/Figure 2/2A/Fig 2A_CDK1_Input.tif]

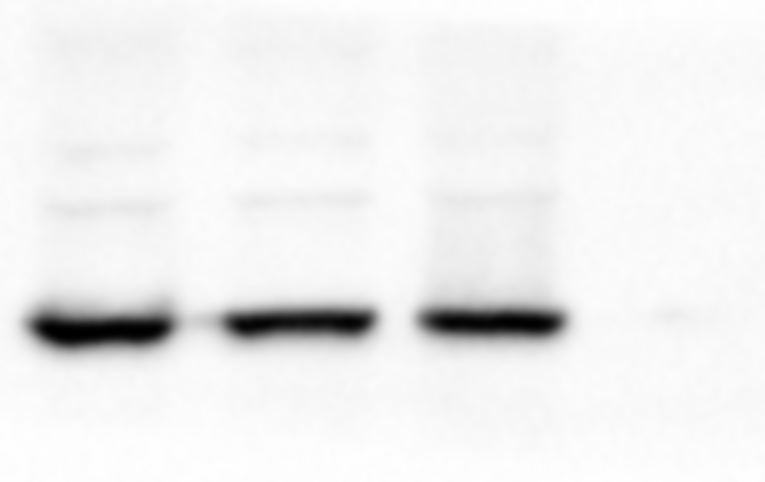

Supplement: Supplementary file 4 — Source data Fig. 2 [file 44318_2024_169_MOESM4_ESM.zip › SD_Figure_2.zip/Figure 2/2A/Fig 2A_CDK1_IP.tif]

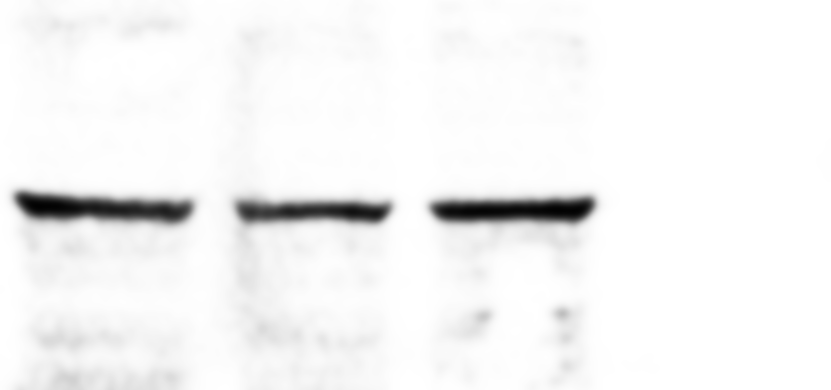

Supplement: Supplementary file 4 — Source data Fig. 2 [file 44318_2024_169_MOESM4_ESM.zip › SD_Figure_2.zip/Figure 2/2A/Fig 2A_TDP1_IP.tif]

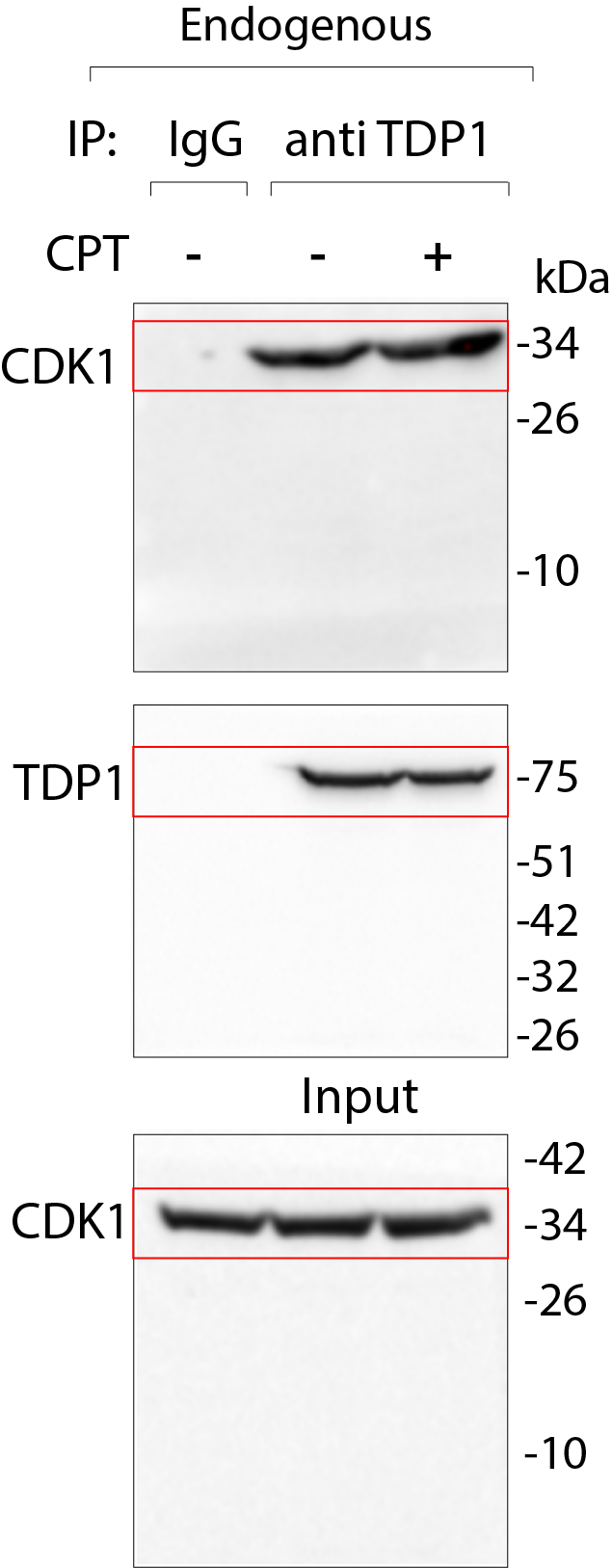

Supplement: Supplementary file 4 — Source data Fig. 2 [file 44318_2024_169_MOESM4_ESM.zip › SD_Figure_2.zip/Figure 2/2B/Fig 2B.tif]

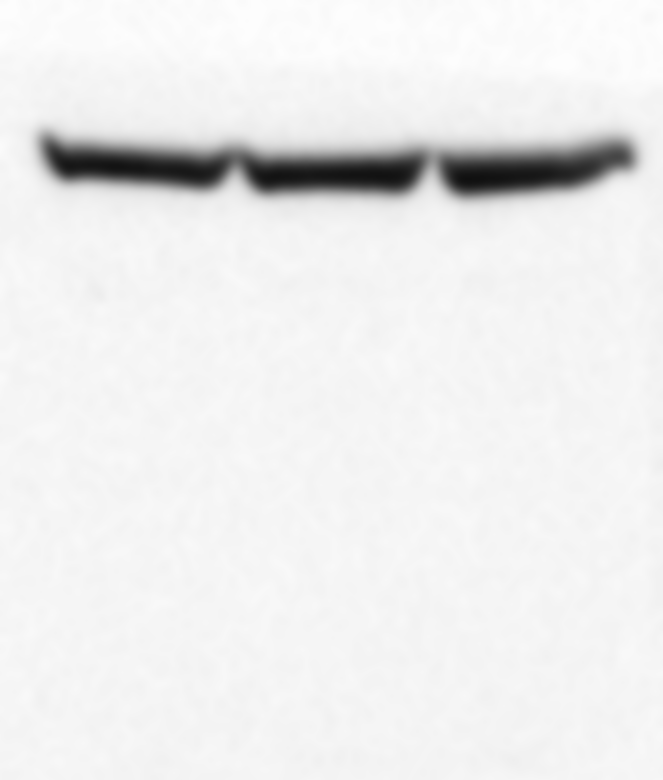

Supplement: Supplementary file 4 — Source data Fig. 2 [file 44318_2024_169_MOESM4_ESM.zip › SD_Figure_2.zip/Figure 2/2B/Fig 2B_CDK1_Input.tif]

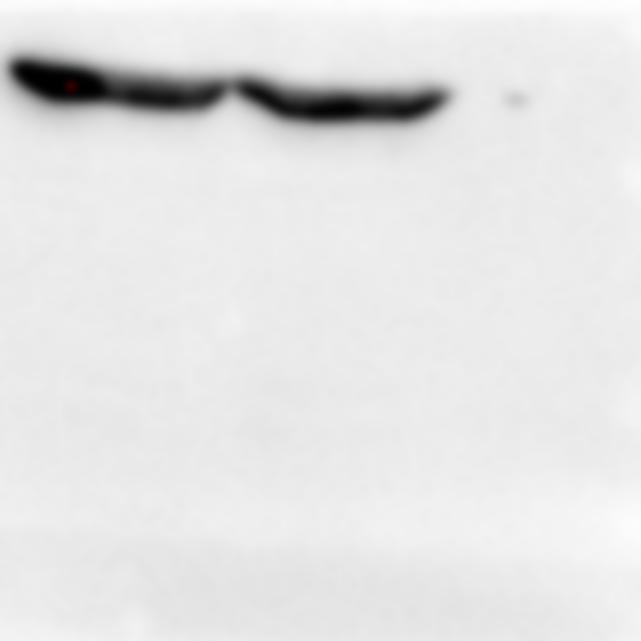

Supplement: Supplementary file 4 — Source data Fig. 2 [file 44318_2024_169_MOESM4_ESM.zip › SD_Figure_2.zip/Figure 2/2B/Fig 2B_CDK1_IP.tif]

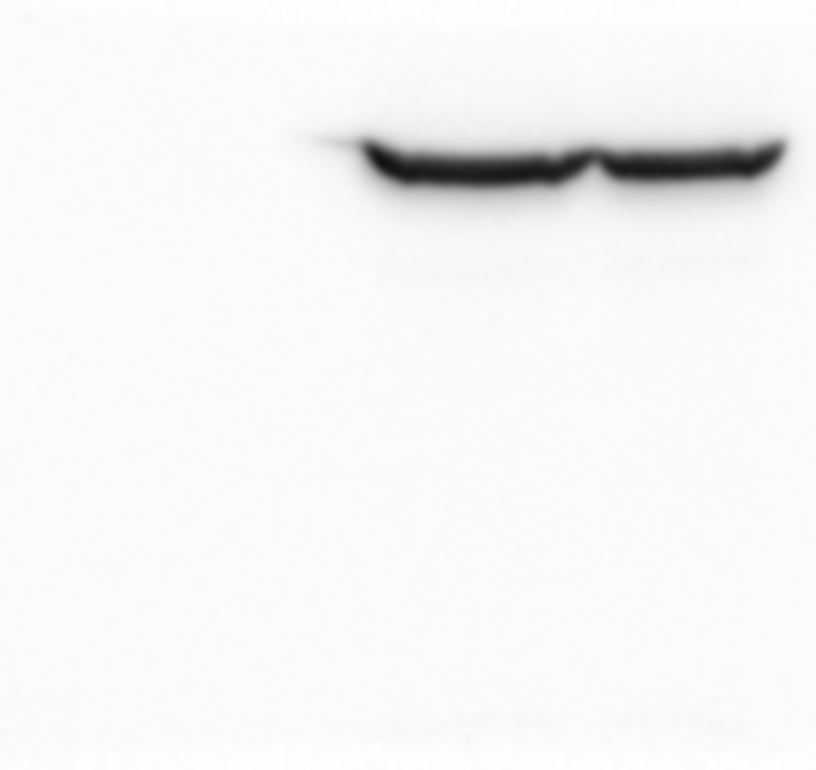

Supplement: Supplementary file 4 — Source data Fig. 2 [file 44318_2024_169_MOESM4_ESM.zip › SD_Figure_2.zip/Figure 2/2B/Fig 2B_TDP1_IP.tif]

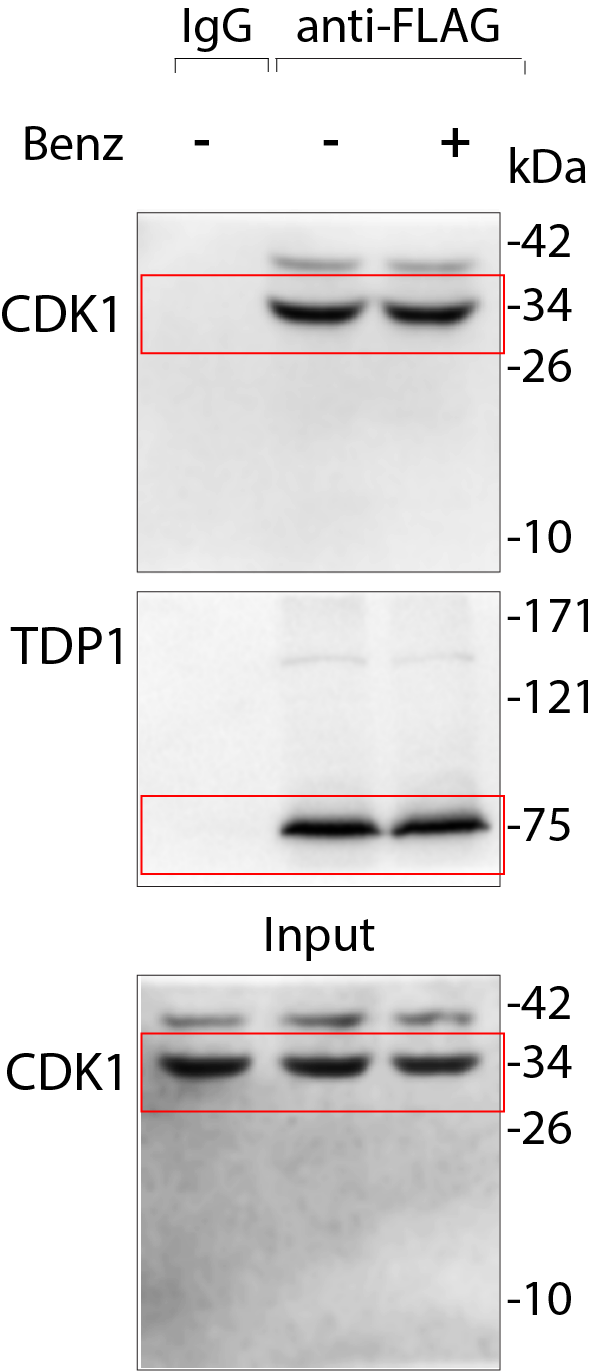

Supplement: Supplementary file 4 — Source data Fig. 2 [file 44318_2024_169_MOESM4_ESM.zip › SD_Figure_2.zip/Figure 2/2C/Fig 2C.tif]

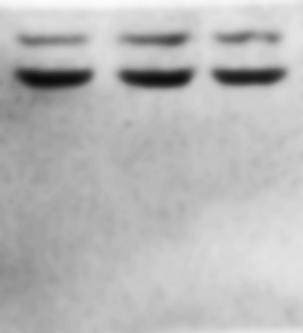

Supplement: Supplementary file 4 — Source data Fig. 2 [file 44318_2024_169_MOESM4_ESM.zip › SD_Figure_2.zip/Figure 2/2C/Fig 2C_CDK1_Input.tif]

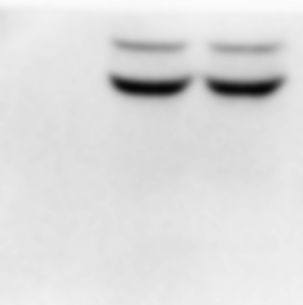

Supplement: Supplementary file 4 — Source data Fig. 2 [file 44318_2024_169_MOESM4_ESM.zip › SD_Figure_2.zip/Figure 2/2C/Fig 2C_CDK1_IP.tif]

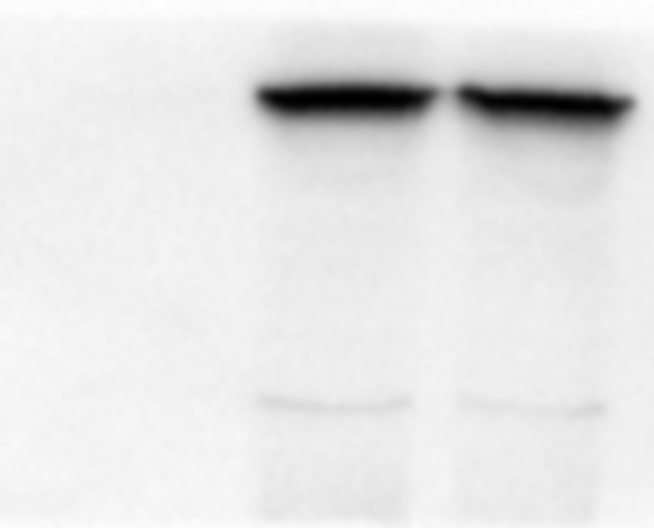

Supplement: Supplementary file 4 — Source data Fig. 2 [file 44318_2024_169_MOESM4_ESM.zip › SD_Figure_2.zip/Figure 2/2C/Fig 2C_TDP1_IP.tif]

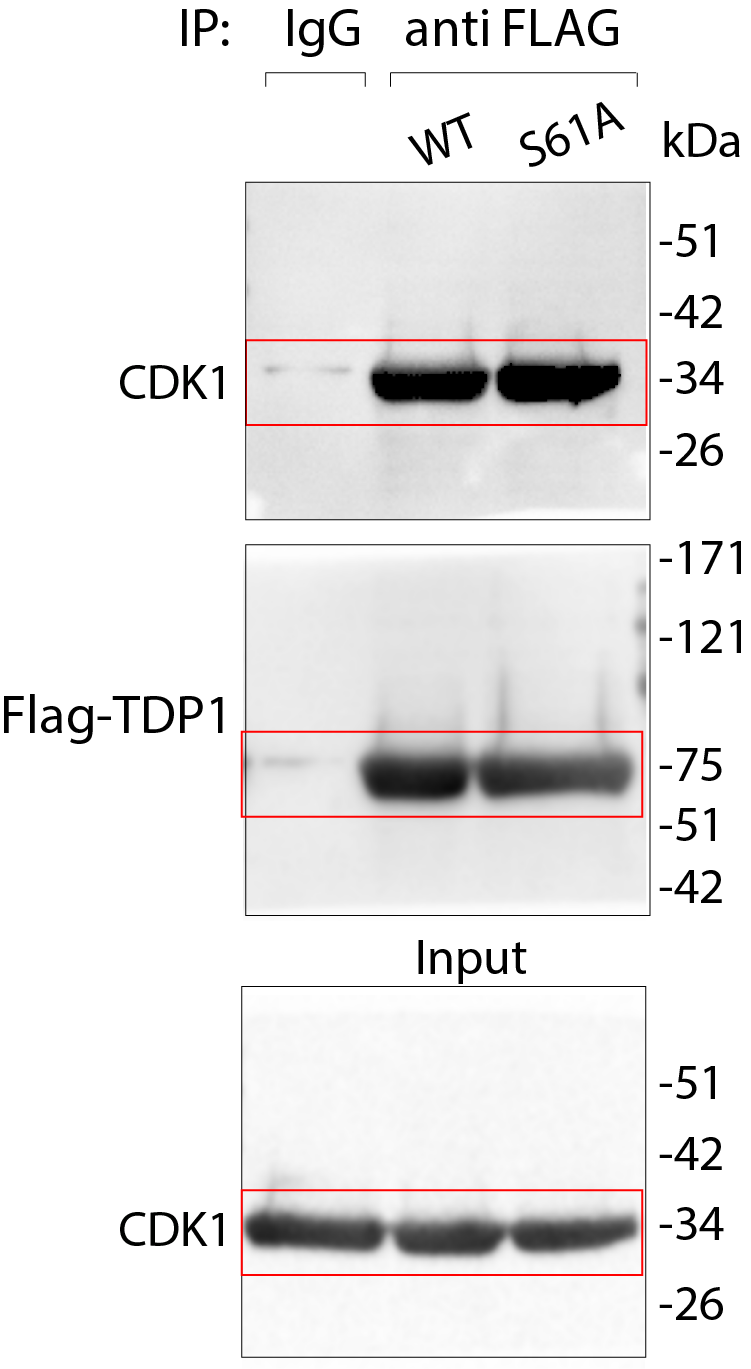

Supplement: Supplementary file 4 — Source data Fig. 2 [file 44318_2024_169_MOESM4_ESM.zip › SD_Figure_2.zip/Figure 2/2D/Fig 2D.tif]

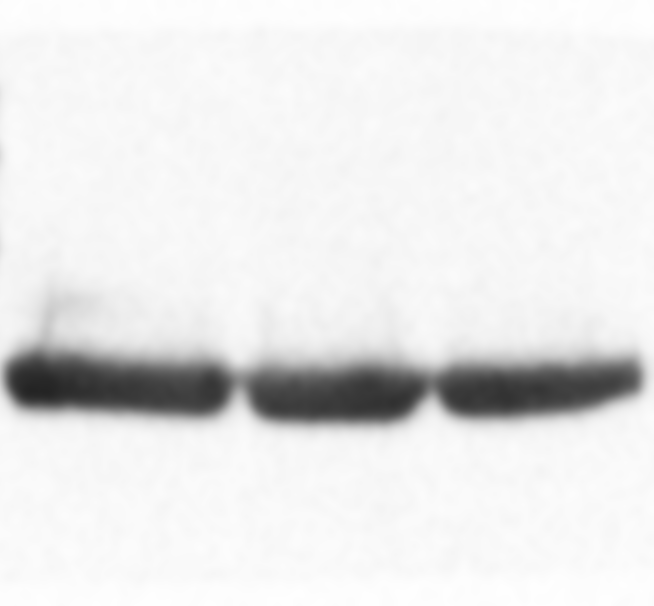

Supplement: Supplementary file 4 — Source data Fig. 2 [file 44318_2024_169_MOESM4_ESM.zip › SD_Figure_2.zip/Figure 2/2D/Fig 2D_CDK1_input.tif]

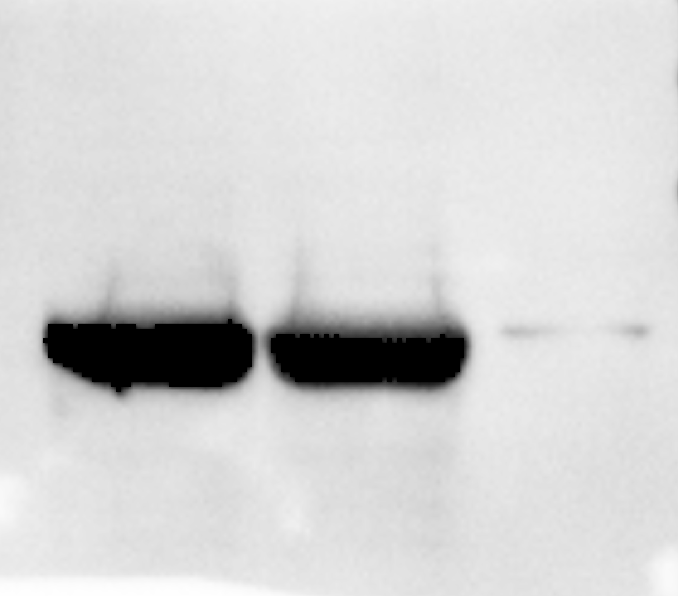

Supplement: Supplementary file 4 — Source data Fig. 2 [file 44318_2024_169_MOESM4_ESM.zip › SD_Figure_2.zip/Figure 2/2D/Fig 2D_CDK1_IP.tif]

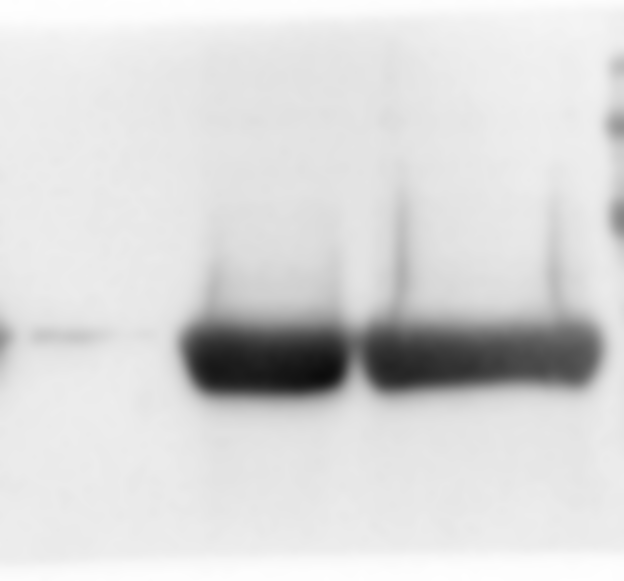

Supplement: Supplementary file 4 — Source data Fig. 2 [file 44318_2024_169_MOESM4_ESM.zip › SD_Figure_2.zip/Figure 2/2D/Fig 2D_TDP1_IP.tif]

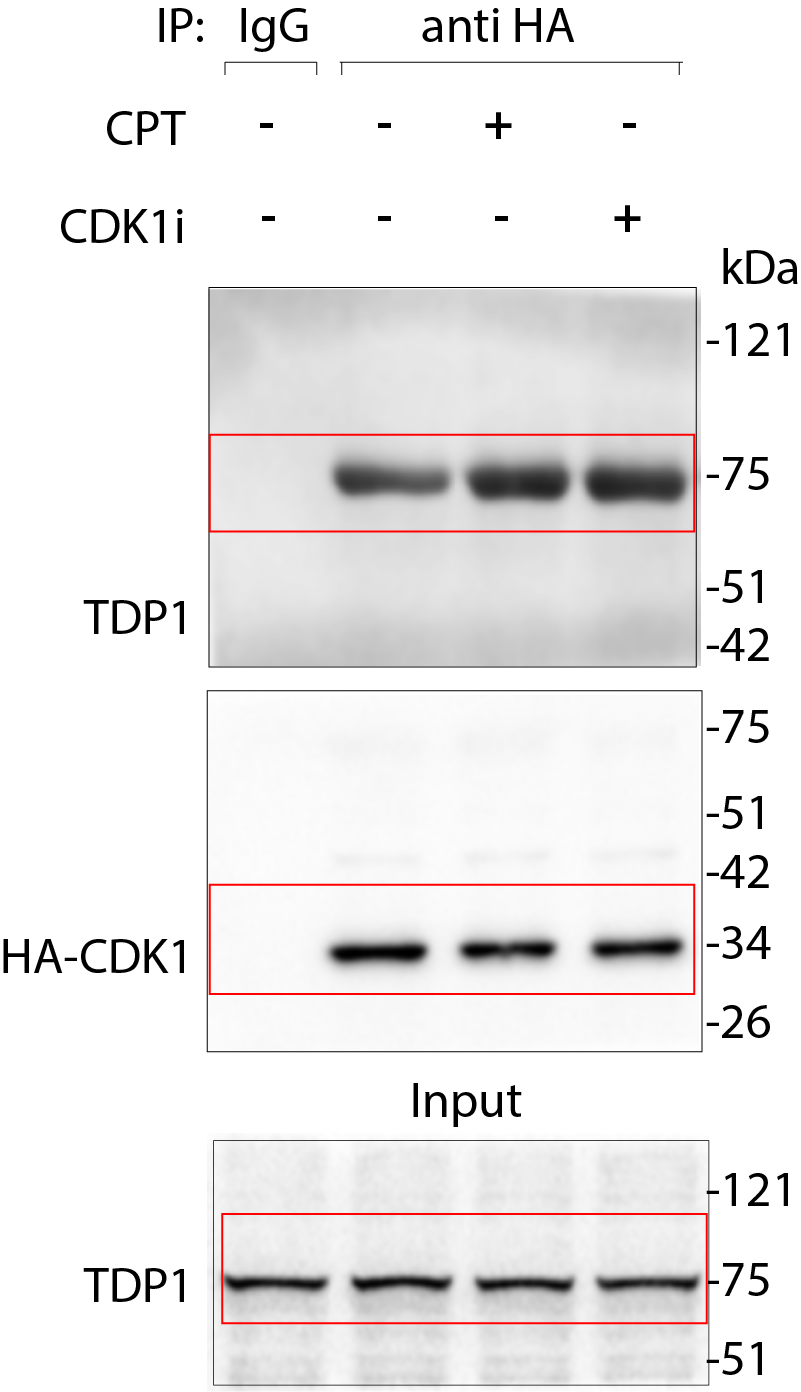

Supplement: Supplementary file 4 — Source data Fig. 2 [file 44318_2024_169_MOESM4_ESM.zip › SD_Figure_2.zip/Figure 2/2E/Fig 2F.tif]

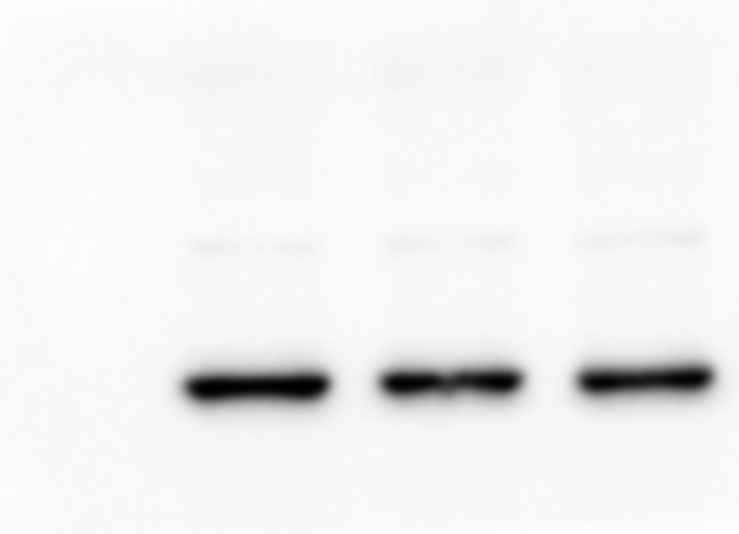

Supplement: Supplementary file 4 — Source data Fig. 2 [file 44318_2024_169_MOESM4_ESM.zip › SD_Figure_2.zip/Figure 2/2E/Fig 2F_CDK1_IP.tif]

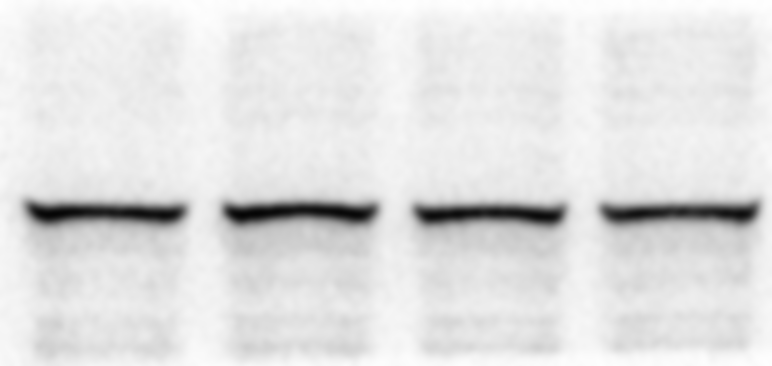

Supplement: Supplementary file 4 — Source data Fig. 2 [file 44318_2024_169_MOESM4_ESM.zip › SD_Figure_2.zip/Figure 2/2E/Fig 2F_TDP1_input.tif]

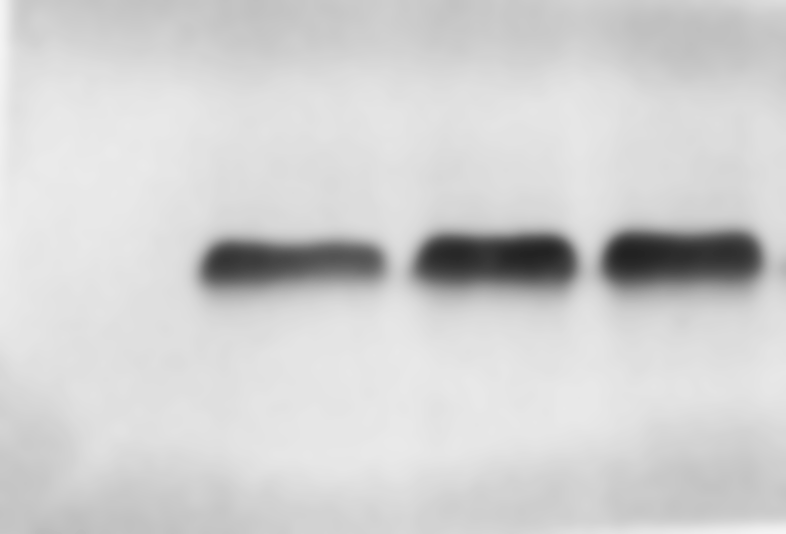

Supplement: Supplementary file 4 — Source data Fig. 2 [file 44318_2024_169_MOESM4_ESM.zip › SD_Figure_2.zip/Figure 2/2E/Fig 2F_TDP1_IP.tif]

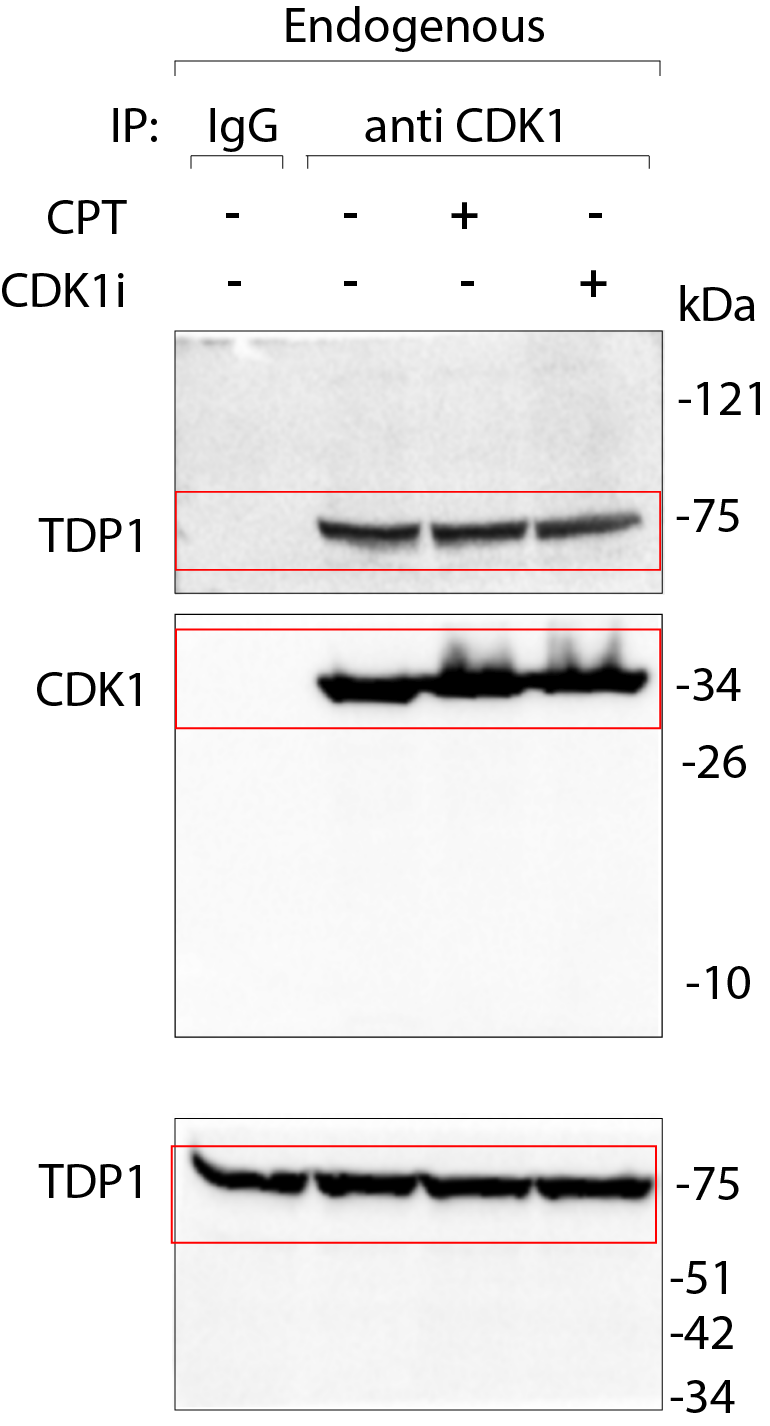

Supplement: Supplementary file 4 — Source data Fig. 2 [file 44318_2024_169_MOESM4_ESM.zip › SD_Figure_2.zip/Figure 2/2F/Fig 2F.tif]

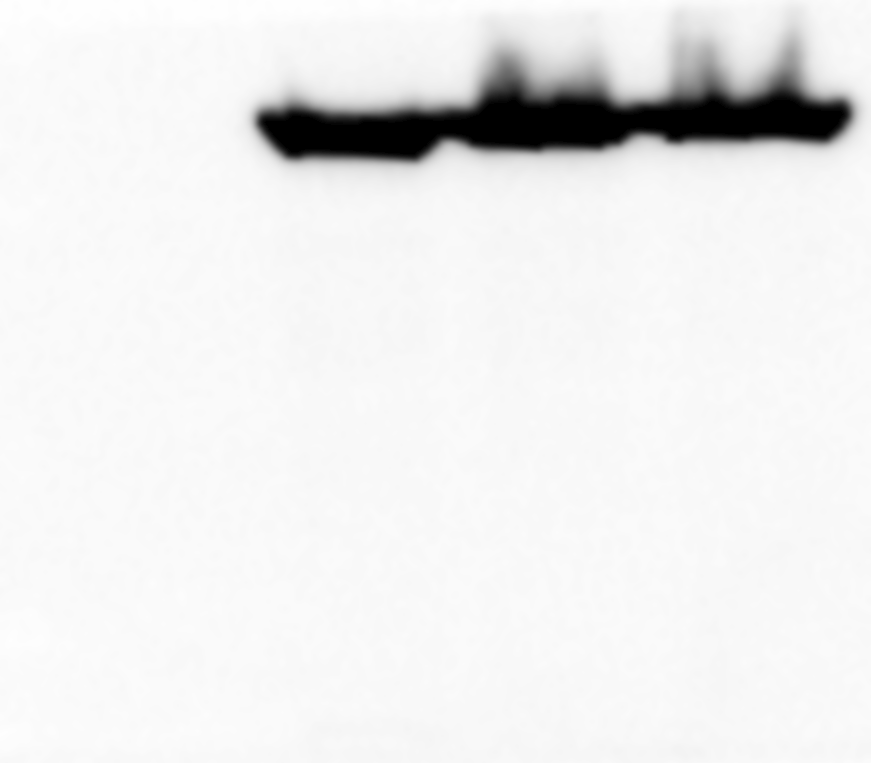

Supplement: Supplementary file 4 — Source data Fig. 2 [file 44318_2024_169_MOESM4_ESM.zip › SD_Figure_2.zip/Figure 2/2F/Fig 2F_CDK1_IP.tif]

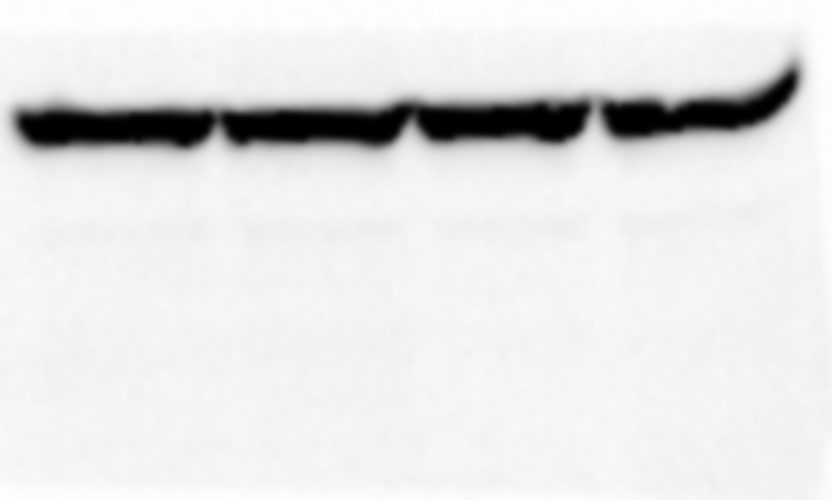

Supplement: Supplementary file 4 — Source data Fig. 2 [file 44318_2024_169_MOESM4_ESM.zip › SD_Figure_2.zip/Figure 2/2F/Fig 2F_TDP1_input.tif]

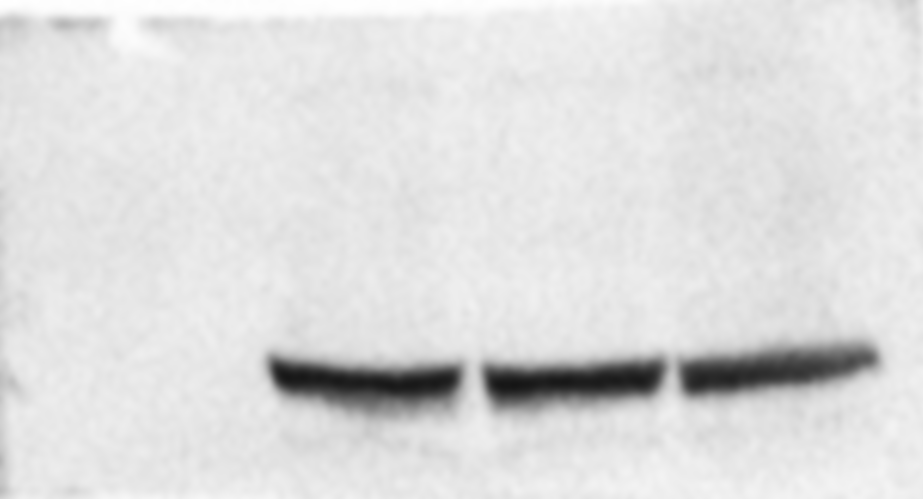

Supplement: Supplementary file 4 — Source data Fig. 2 [file 44318_2024_169_MOESM4_ESM.zip › SD_Figure_2.zip/Figure 2/2F/Fig 2F_TDP1_IP.tif]

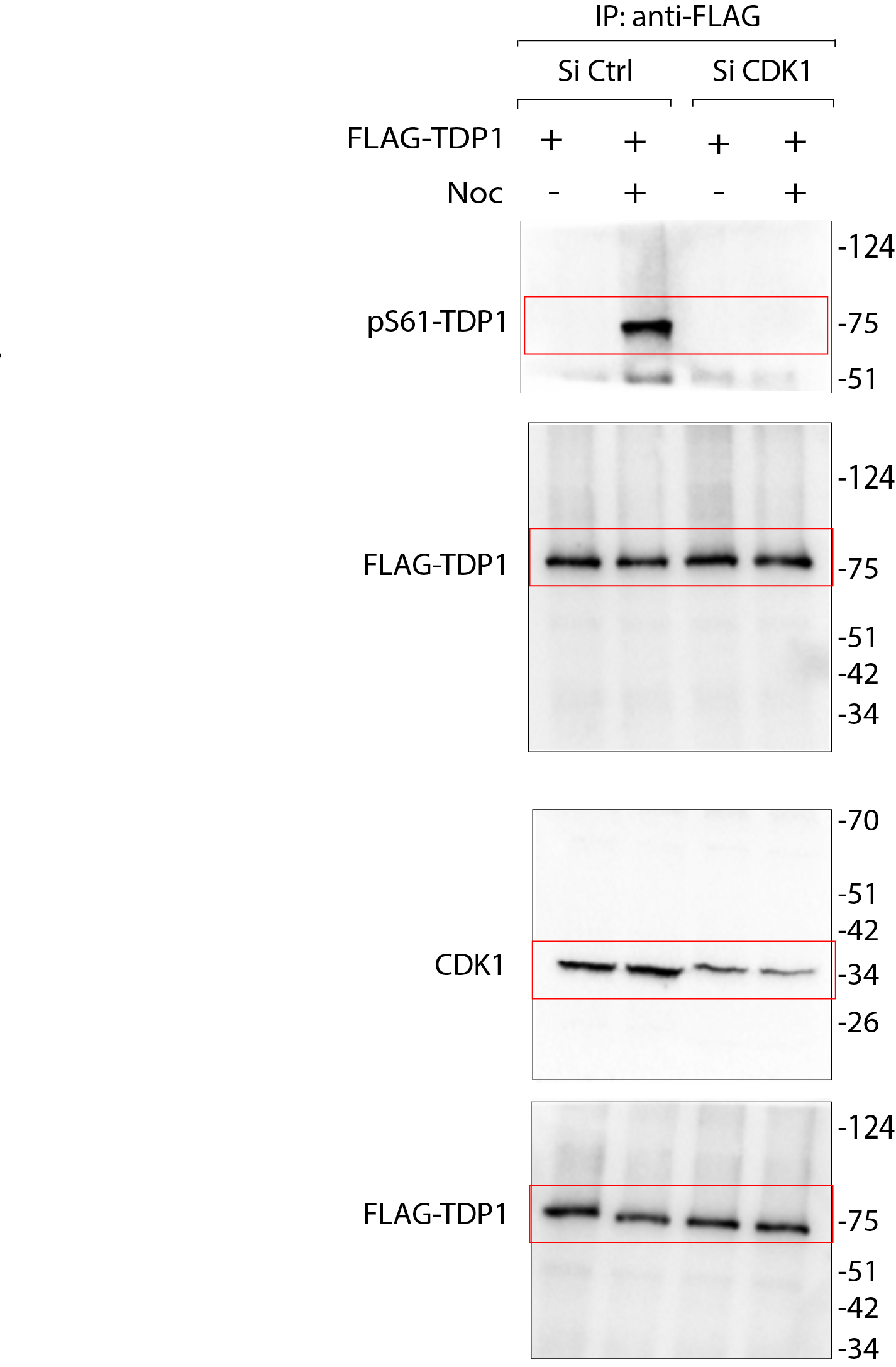

Supplement: Supplementary file 4 — Source data Fig. 2 [file 44318_2024_169_MOESM4_ESM.zip › SD_Figure_2.zip/Figure 2/2G/Fig 2G.tif]

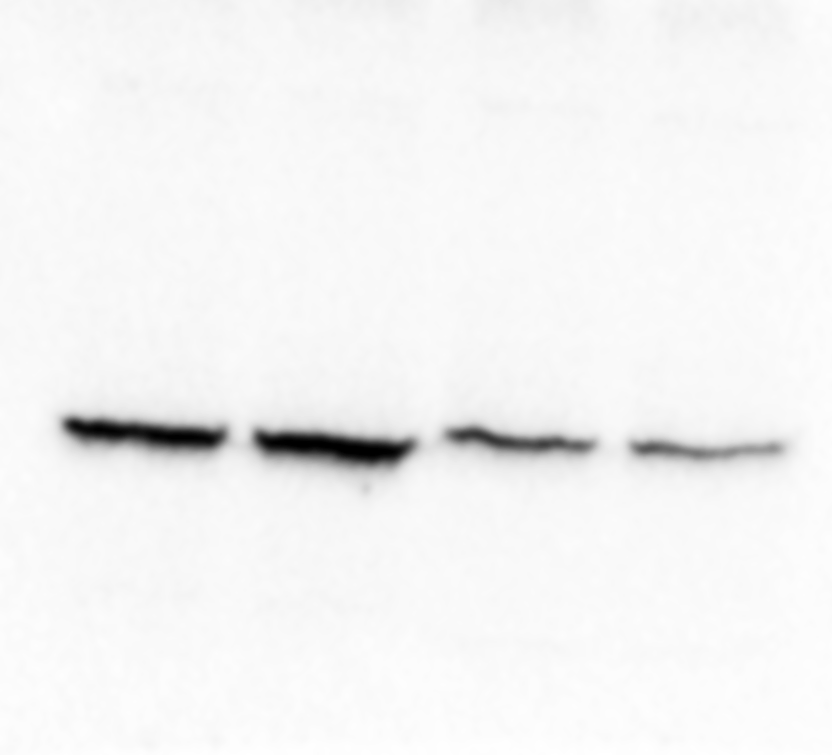

Supplement: Supplementary file 4 — Source data Fig. 2 [file 44318_2024_169_MOESM4_ESM.zip › SD_Figure_2.zip/Figure 2/2G/Fig 2G_CDK1_input.tif]

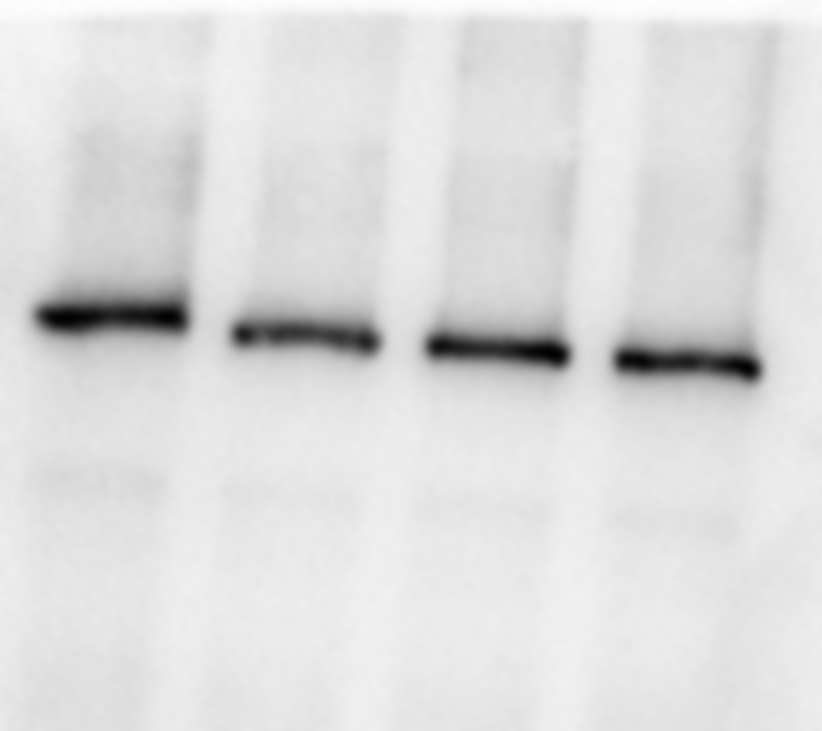

Supplement: Supplementary file 4 — Source data Fig. 2 [file 44318_2024_169_MOESM4_ESM.zip › SD_Figure_2.zip/Figure 2/2G/Fig 2G_FLAG-TDP1_input.tif]

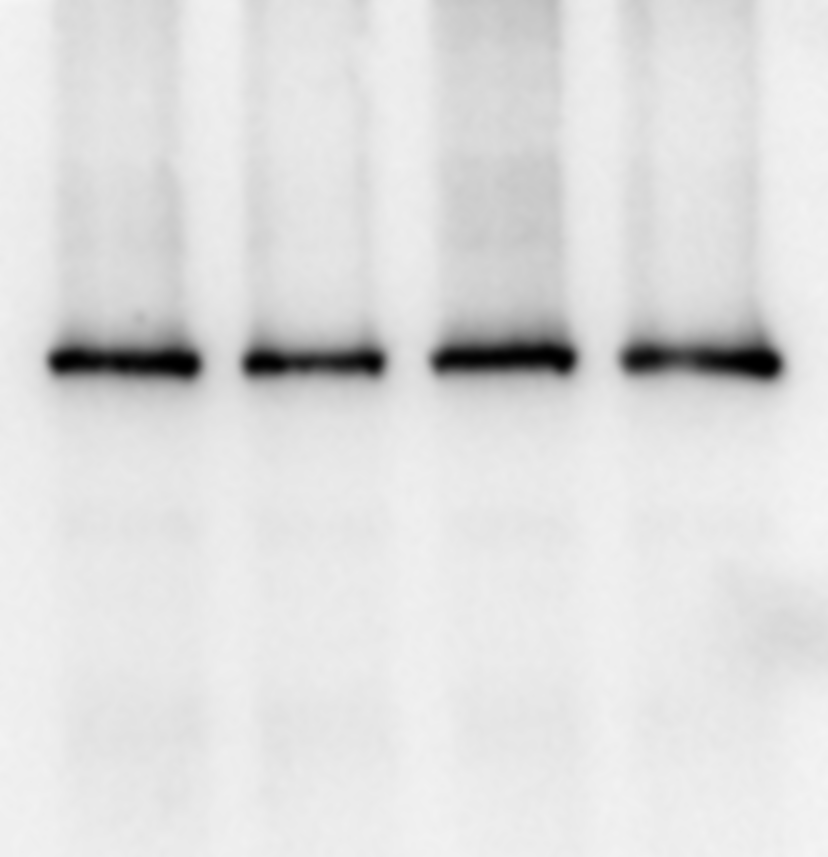

Supplement: Supplementary file 4 — Source data Fig. 2 [file 44318_2024_169_MOESM4_ESM.zip › SD_Figure_2.zip/Figure 2/2G/Fig 2H_FLAG-TDP1_IP.tif]

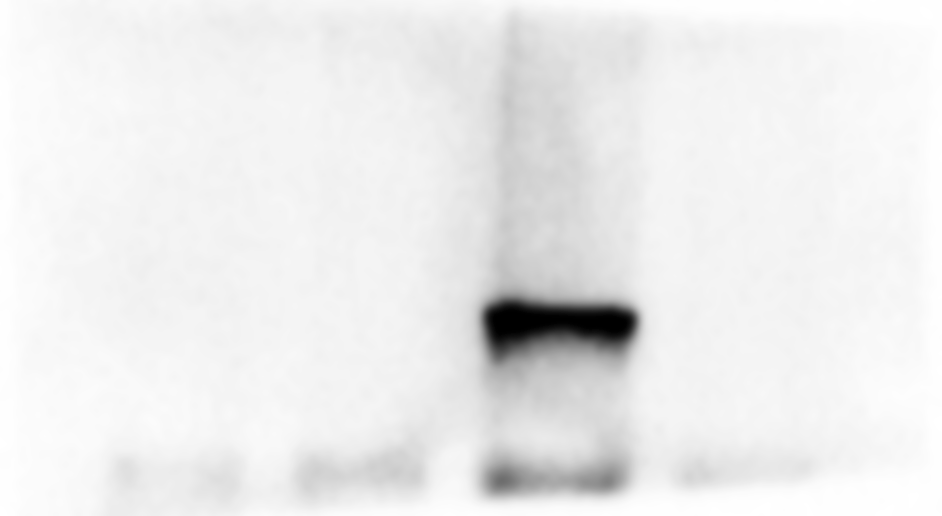

Supplement: Supplementary file 4 — Source data Fig. 2 [file 44318_2024_169_MOESM4_ESM.zip › SD_Figure_2.zip/Figure 2/2G/Fig 2H_pS61-TDP1.tif]

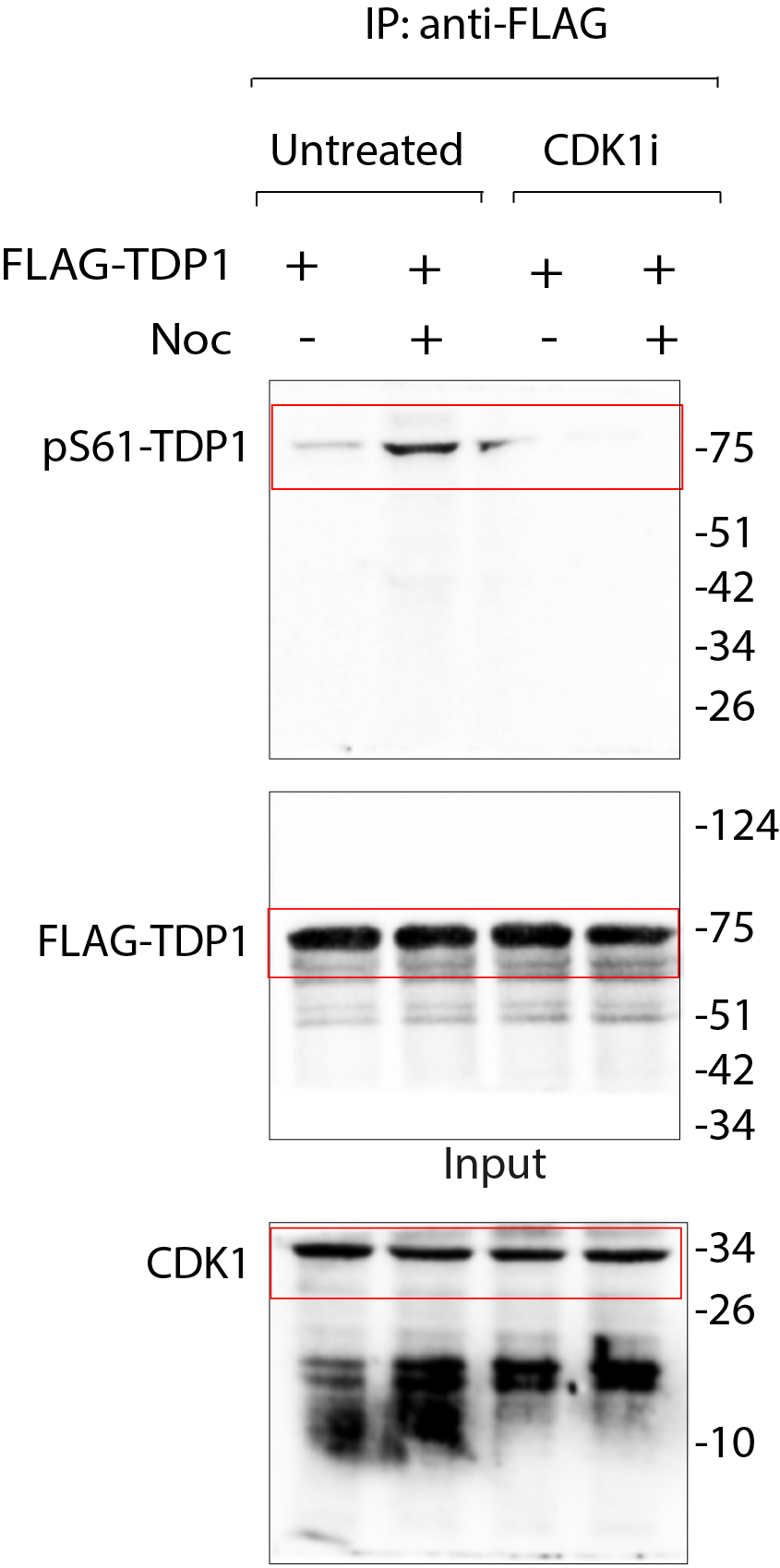

Supplement: Supplementary file 4 — Source data Fig. 2 [file 44318_2024_169_MOESM4_ESM.zip › SD_Figure_2.zip/Figure 2/2H/Fig 2H.tif]

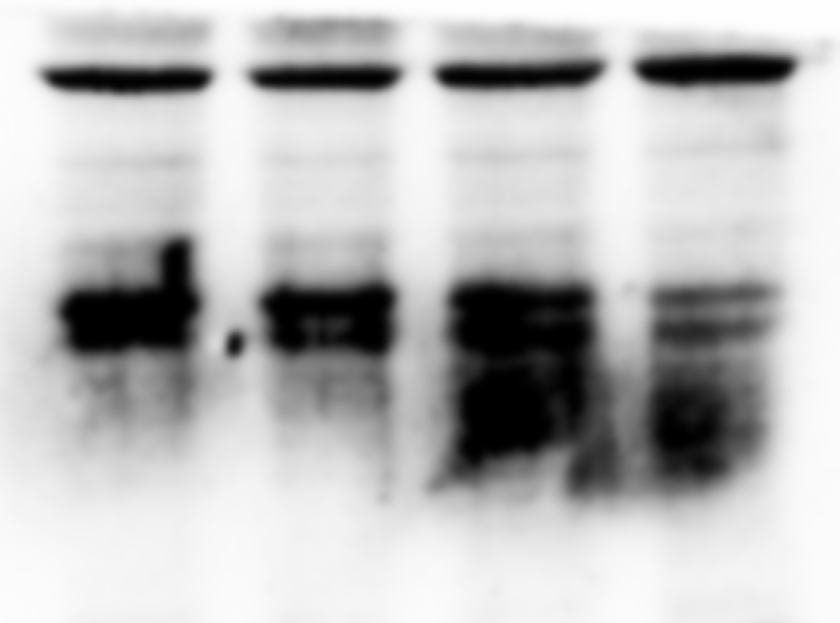

Supplement: Supplementary file 4 — Source data Fig. 2 [file 44318_2024_169_MOESM4_ESM.zip › SD_Figure_2.zip/Figure 2/2H/Fig 2H_CDK1_input.tif]

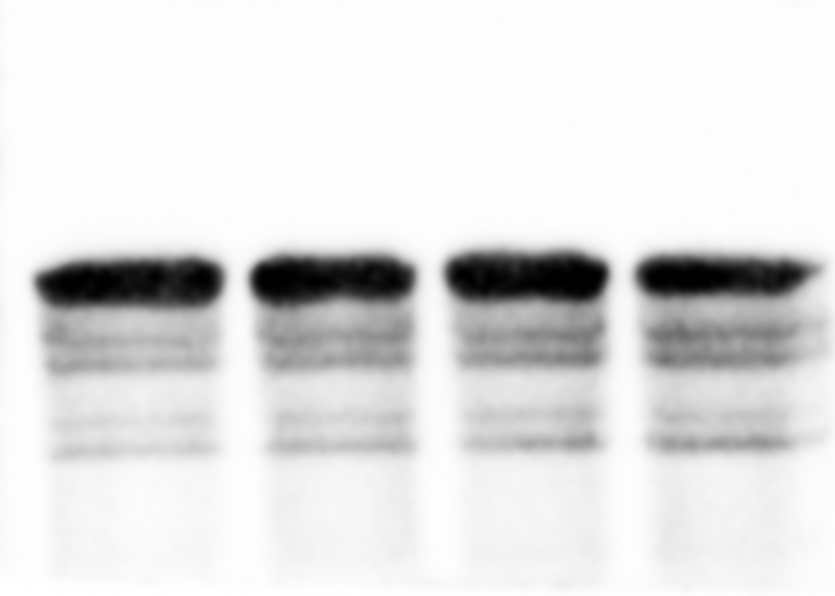

Supplement: Supplementary file 4 — Source data Fig. 2 [file 44318_2024_169_MOESM4_ESM.zip › SD_Figure_2.zip/Figure 2/2H/Fig 2H_FLAG-TDP1.tif]

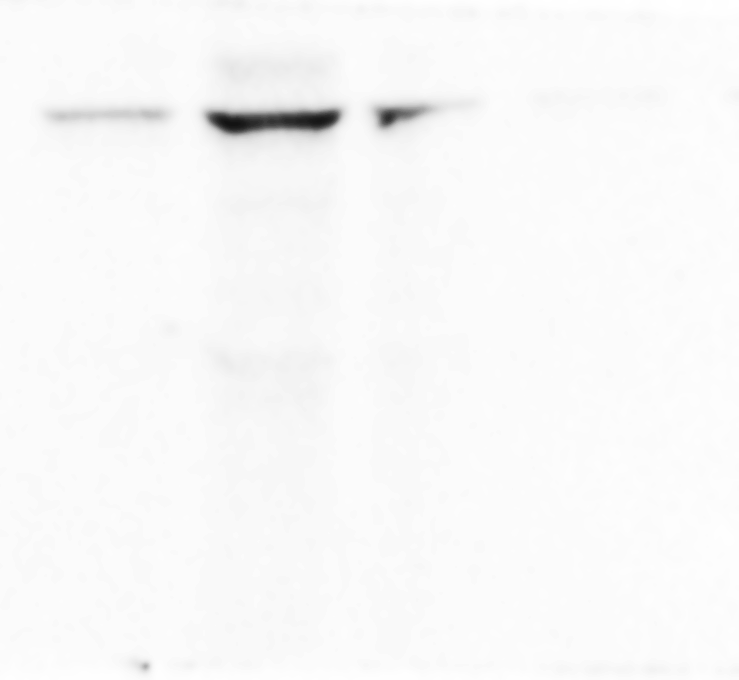

Supplement: Supplementary file 4 — Source data Fig. 2 [file 44318_2024_169_MOESM4_ESM.zip › SD_Figure_2.zip/Figure 2/2H/Fig 2H_pS61-TDP1.tif]

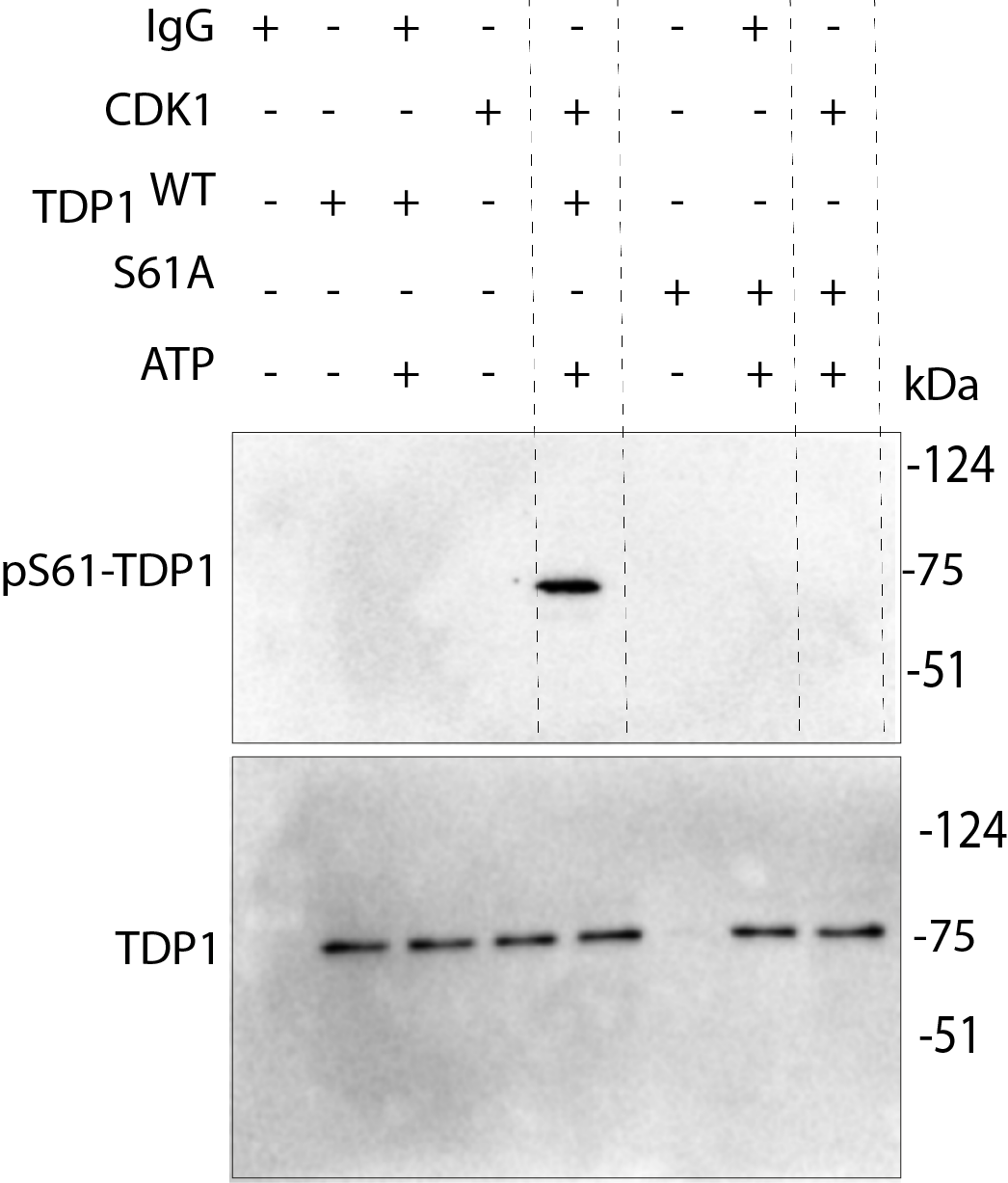

Supplement: Supplementary file 4 — Source data Fig. 2 [file 44318_2024_169_MOESM4_ESM.zip › SD_Figure_2.zip/Figure 2/2I/Fig 2I.tif]

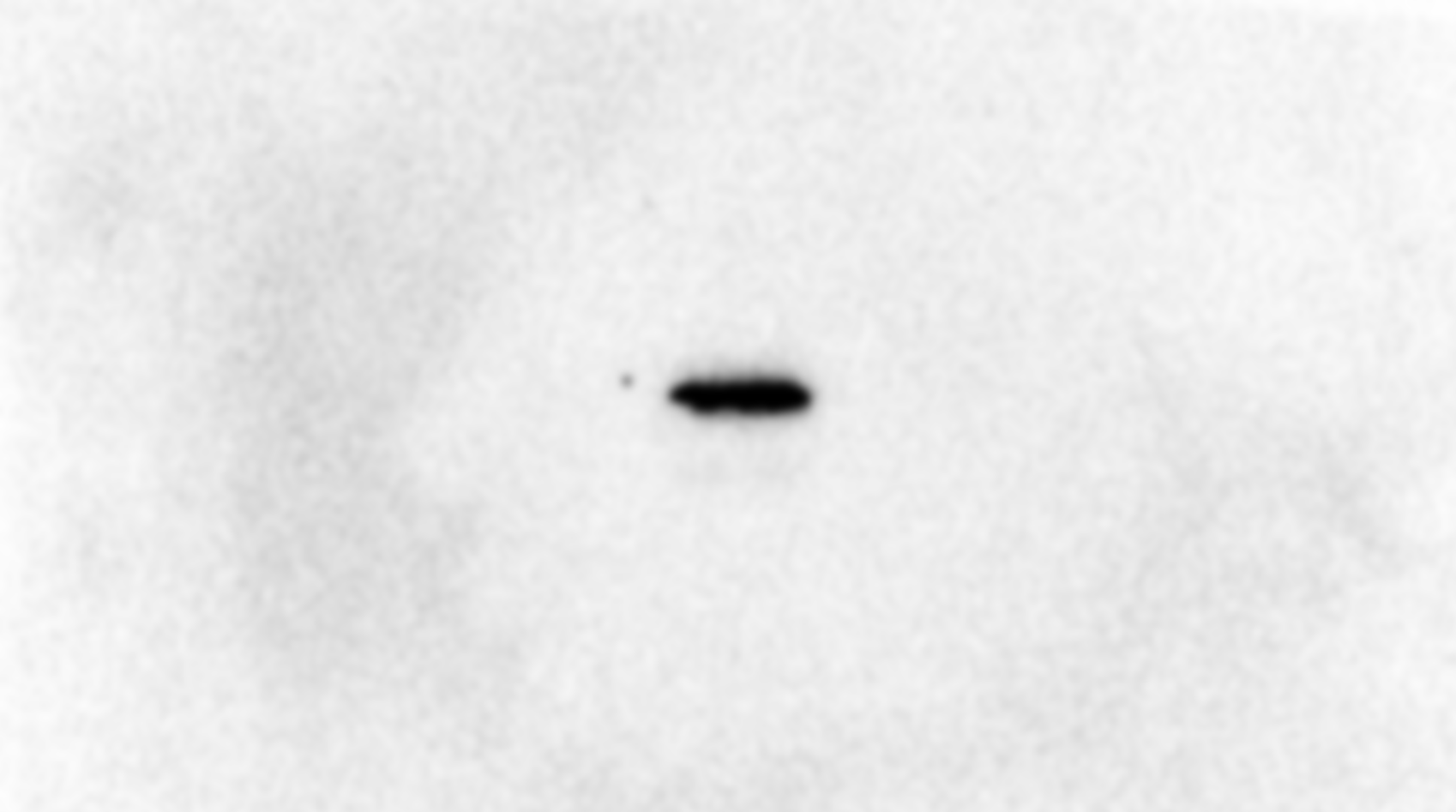

Supplement: Supplementary file 4 — Source data Fig. 2 [file 44318_2024_169_MOESM4_ESM.zip › SD_Figure_2.zip/Figure 2/2I/Fig 2I_pS61-TDP1.tif]

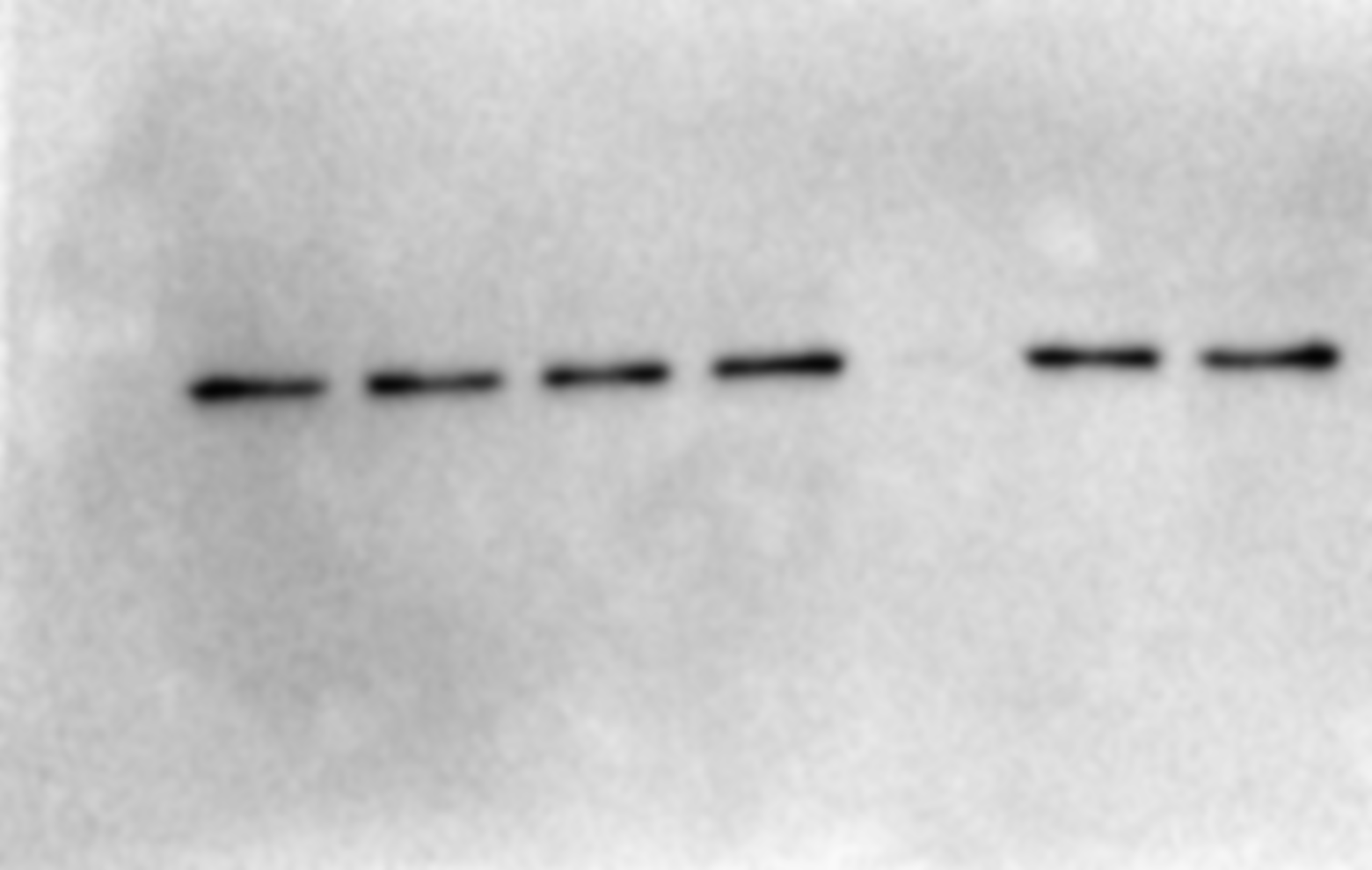

Supplement: Supplementary file 4 — Source data Fig. 2 [file 44318_2024_169_MOESM4_ESM.zip › SD_Figure_2.zip/Figure 2/2I/Fig 2I_TDP1.tif]

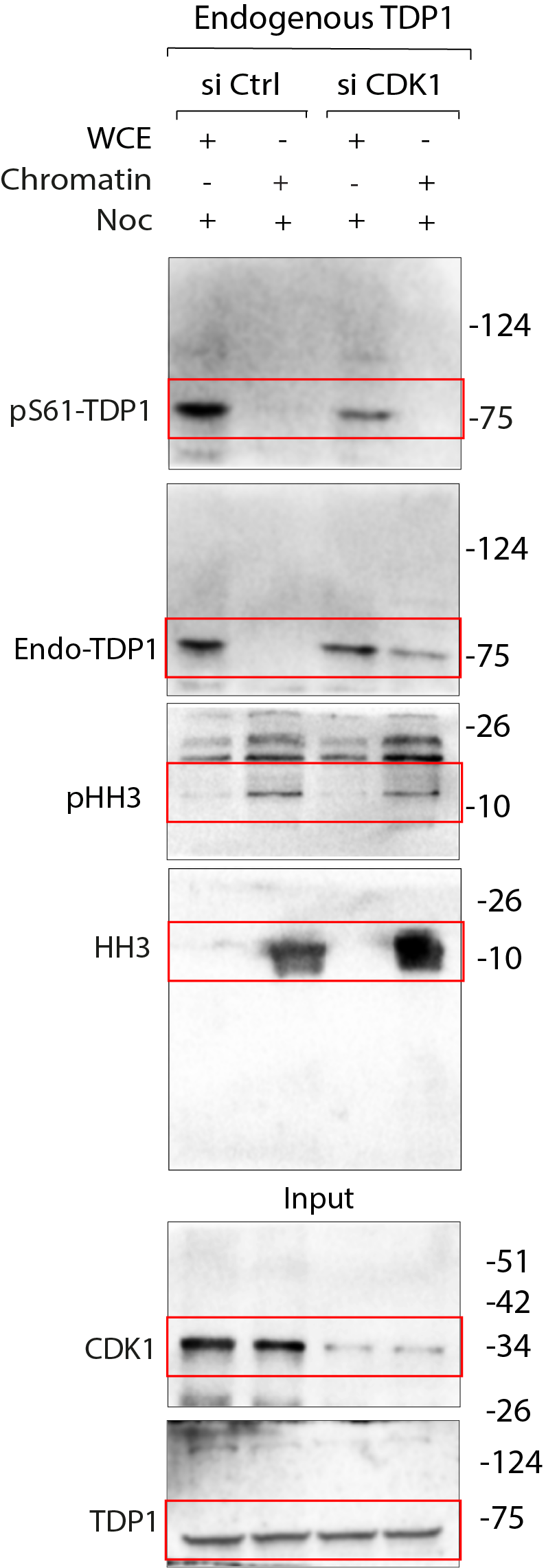

Supplement: Supplementary file 5 — Source data Fig. 3 [file 44318_2024_169_MOESM5_ESM.zip › SD_Figure_3.zip/Figure 3/3A/Fig 3A.tif]

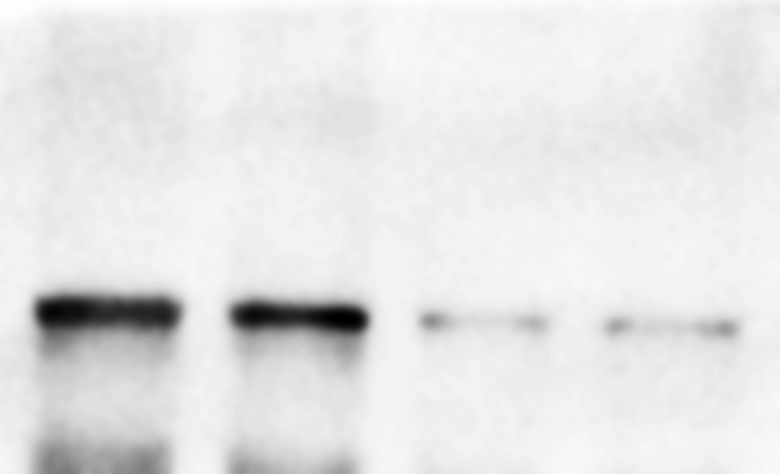

Supplement: Supplementary file 5 — Source data Fig. 3 [file 44318_2024_169_MOESM5_ESM.zip › SD_Figure_3.zip/Figure 3/3A/Fig 3A_CDK1.tif]

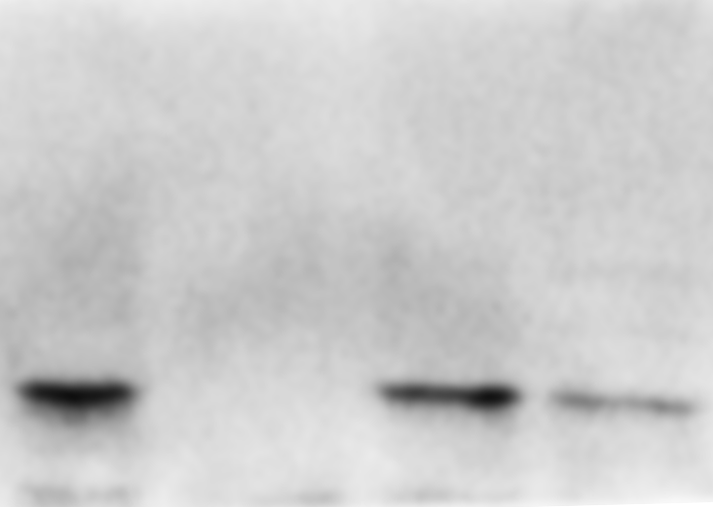

Supplement: Supplementary file 5 — Source data Fig. 3 [file 44318_2024_169_MOESM5_ESM.zip › SD_Figure_3.zip/Figure 3/3A/Fig 3A_endo-TDP1.tif]

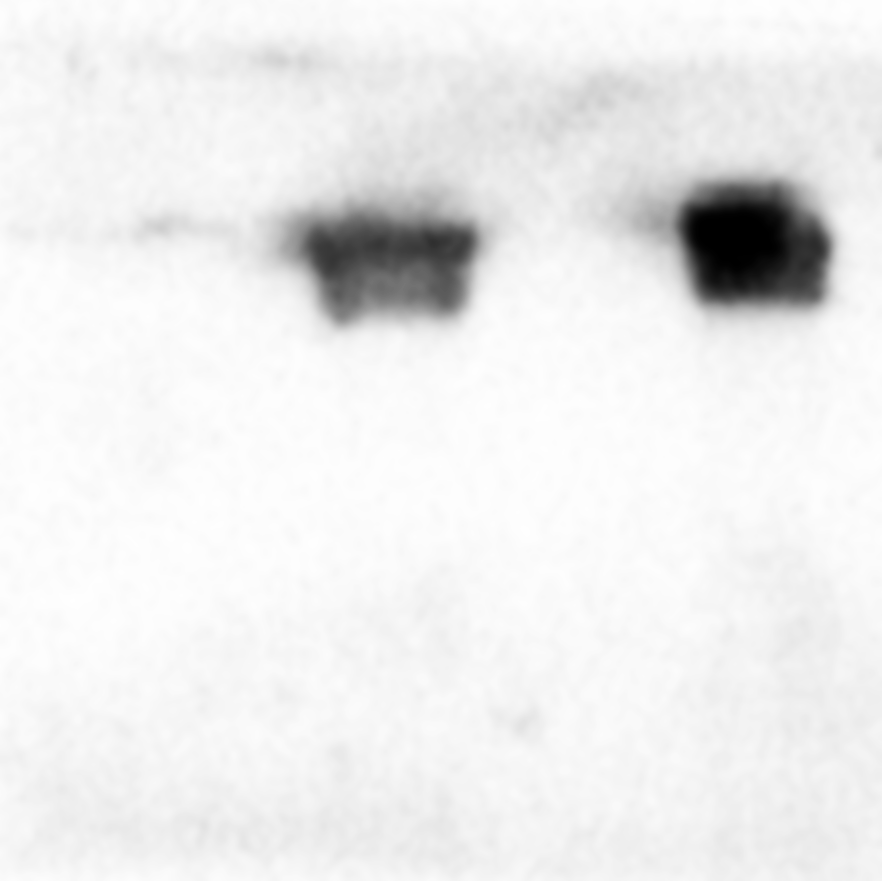

Supplement: Supplementary file 5 — Source data Fig. 3 [file 44318_2024_169_MOESM5_ESM.zip › SD_Figure_3.zip/Figure 3/3A/Fig 3A_HH3.tif]

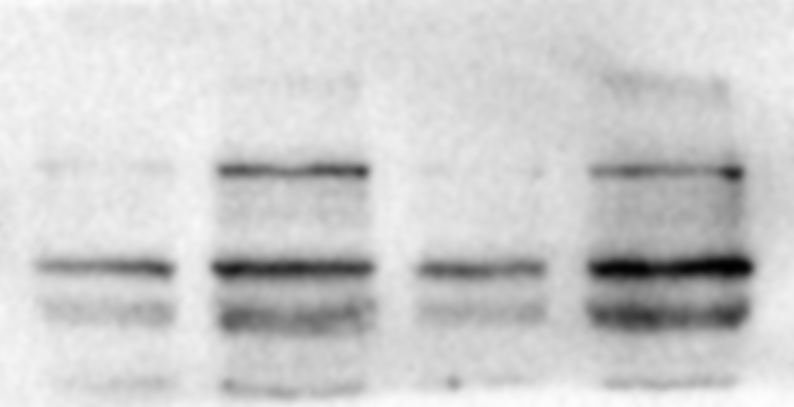

Supplement: Supplementary file 5 — Source data Fig. 3 [file 44318_2024_169_MOESM5_ESM.zip › SD_Figure_3.zip/Figure 3/3A/Fig 3A_pHH3.tif]

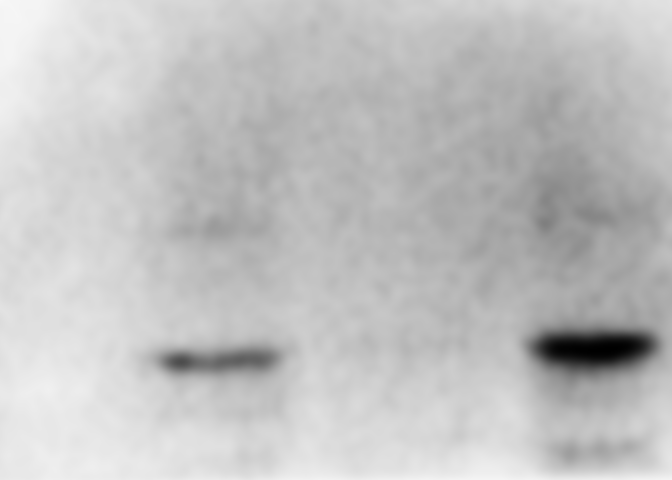

Supplement: Supplementary file 5 — Source data Fig. 3 [file 44318_2024_169_MOESM5_ESM.zip › SD_Figure_3.zip/Figure 3/3A/Fig 3A_pS61-TDP1.tif]

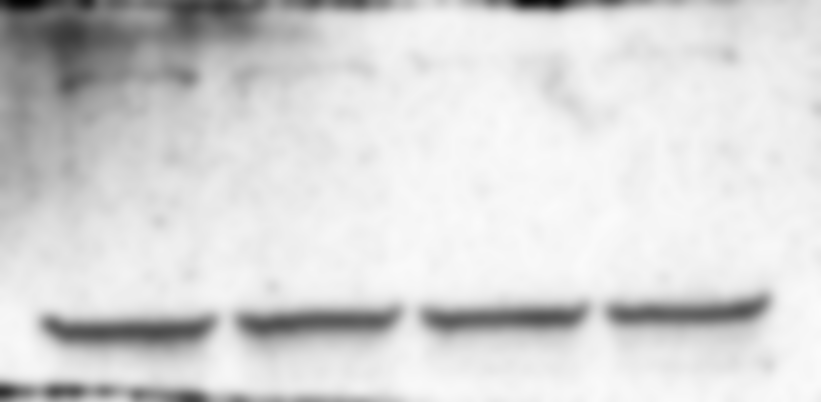

Supplement: Supplementary file 5 — Source data Fig. 3 [file 44318_2024_169_MOESM5_ESM.zip › SD_Figure_3.zip/Figure 3/3A/Fig 3A_TDP1.tif]

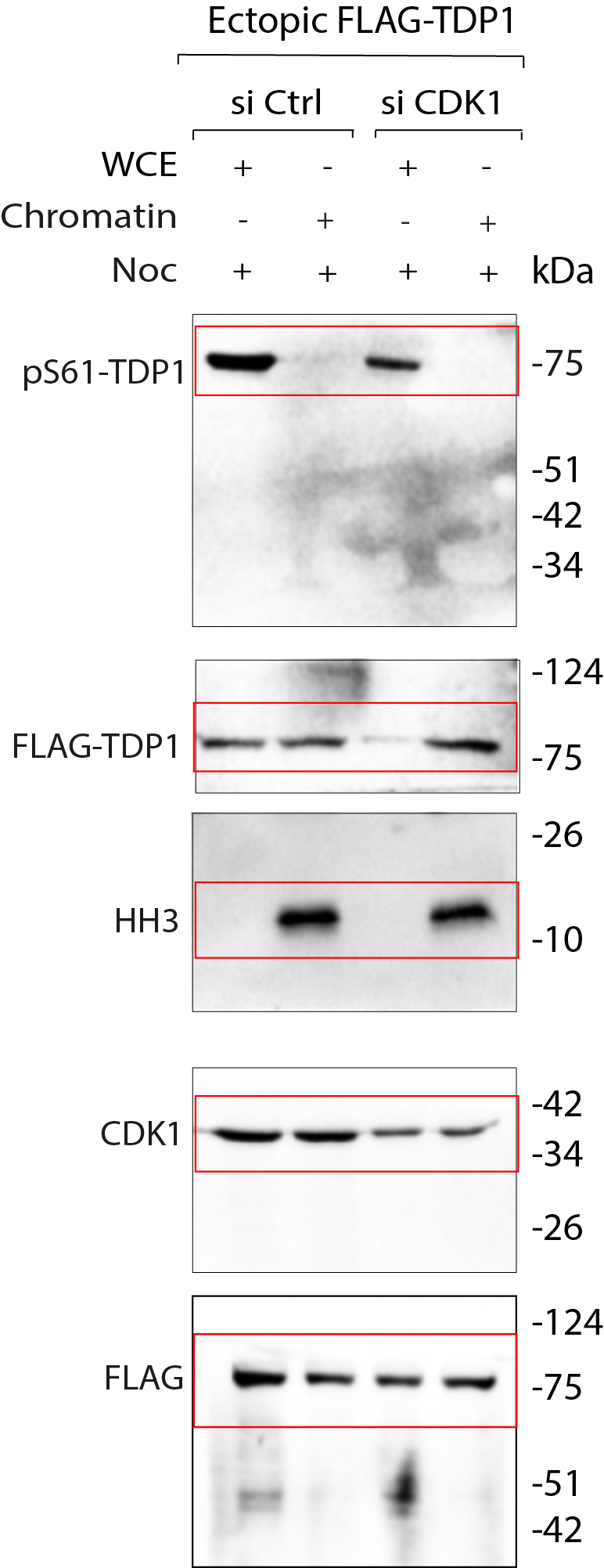

Supplement: Supplementary file 5 — Source data Fig. 3 [file 44318_2024_169_MOESM5_ESM.zip › SD_Figure_3.zip/Figure 3/3C/Fig 3C.tif]

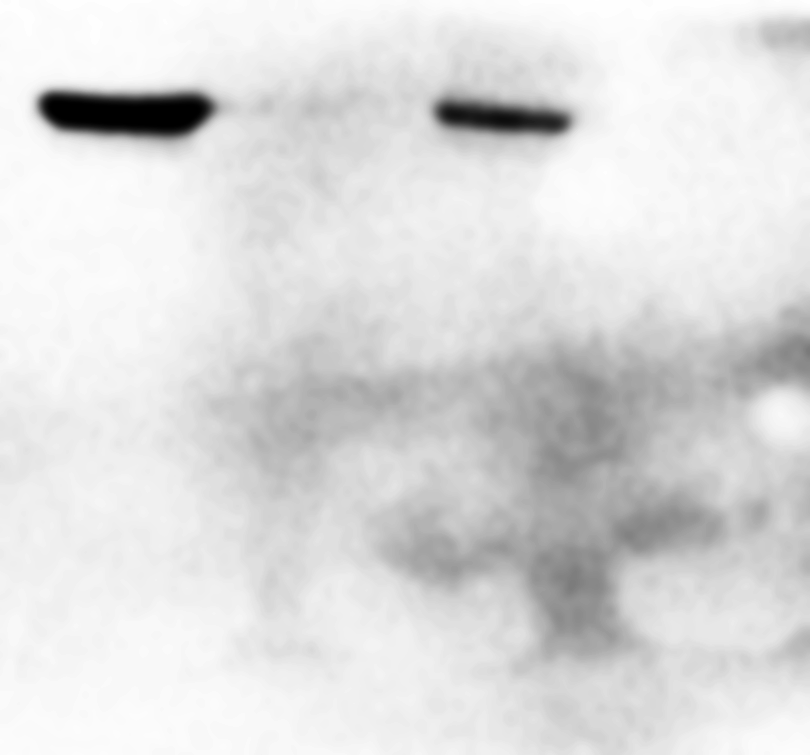

Supplement: Supplementary file 5 — Source data Fig. 3 [file 44318_2024_169_MOESM5_ESM.zip › SD_Figure_3.zip/Figure 3/3C/Fig 3C.tif]

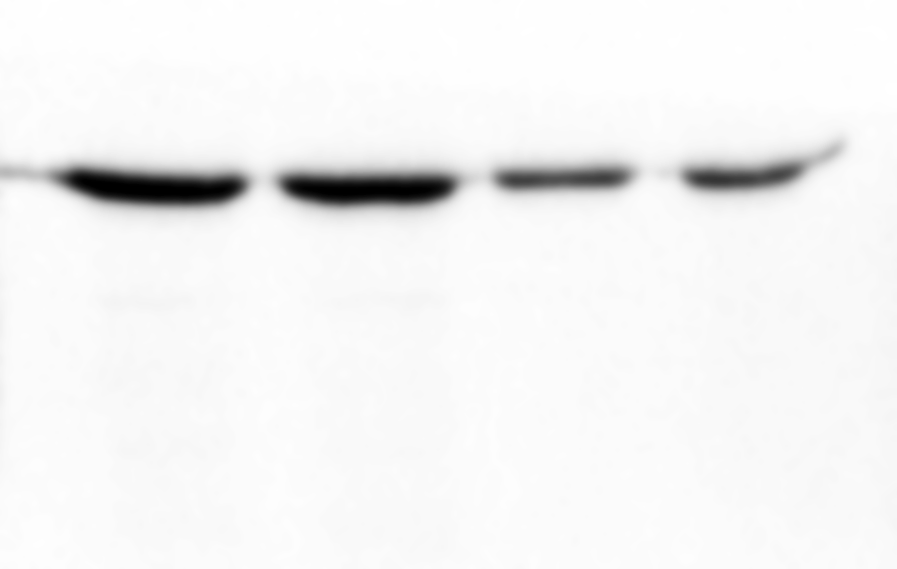

Supplement: Supplementary file 5 — Source data Fig. 3 [file 44318_2024_169_MOESM5_ESM.zip › SD_Figure_3.zip/Figure 3/3C/Fig 3C_CDK1.tif]

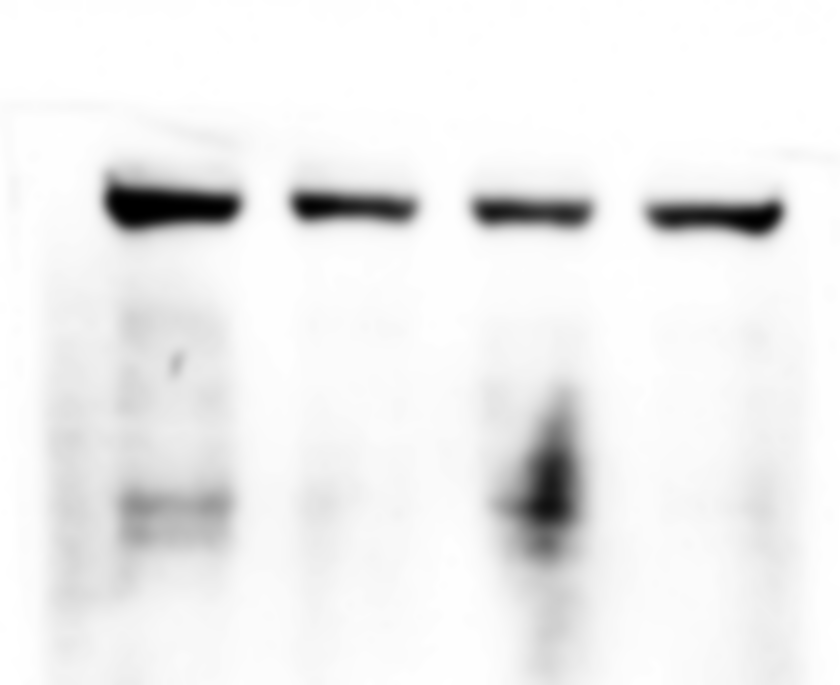

Supplement: Supplementary file 5 — Source data Fig. 3 [file 44318_2024_169_MOESM5_ESM.zip › SD_Figure_3.zip/Figure 3/3C/Fig 3C_FLAG.tif]

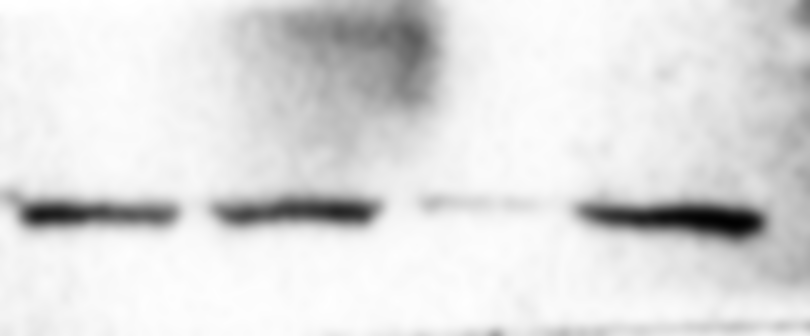

Supplement: Supplementary file 5 — Source data Fig. 3 [file 44318_2024_169_MOESM5_ESM.zip › SD_Figure_3.zip/Figure 3/3C/Fig 3C_FLAG-TDP1.tif]

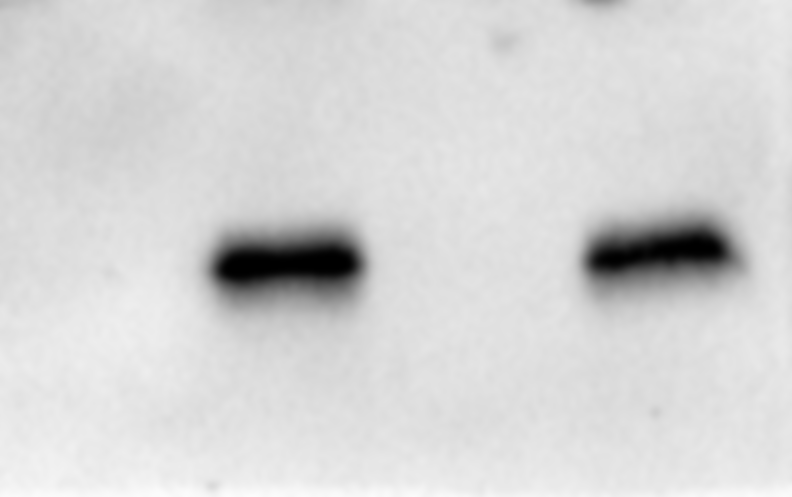

Supplement: Supplementary file 5 — Source data Fig. 3 [file 44318_2024_169_MOESM5_ESM.zip › SD_Figure_3.zip/Figure 3/3C/Fig 3C_HH3.tif]

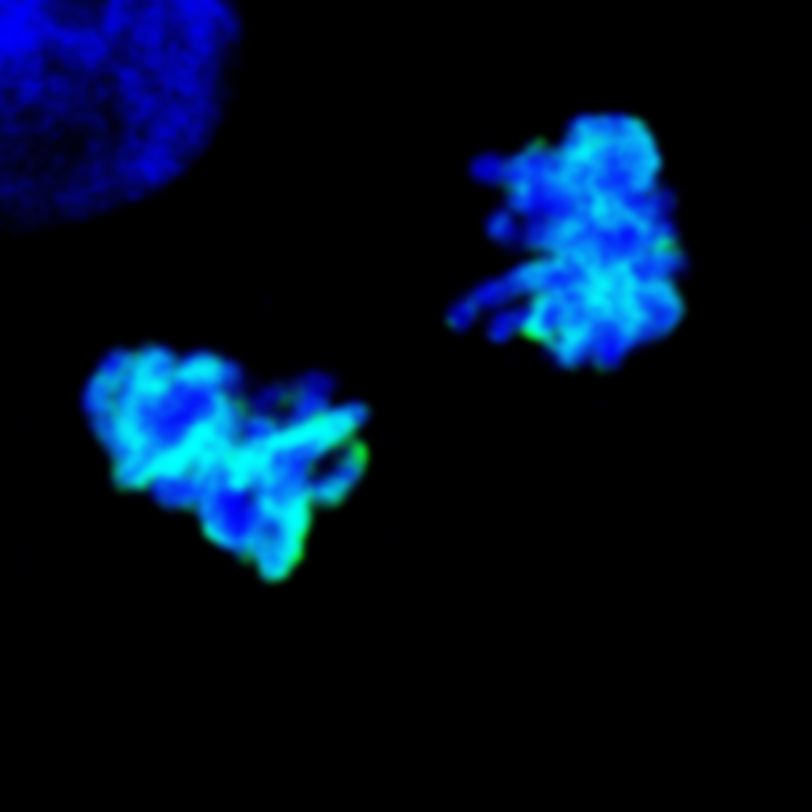

Supplement: Supplementary file 5 — Source data Fig. 3 [file 44318_2024_169_MOESM5_ESM.zip › SD_Figure_3.zip/Figure 3/3F/+RO/3F_ana.tif]

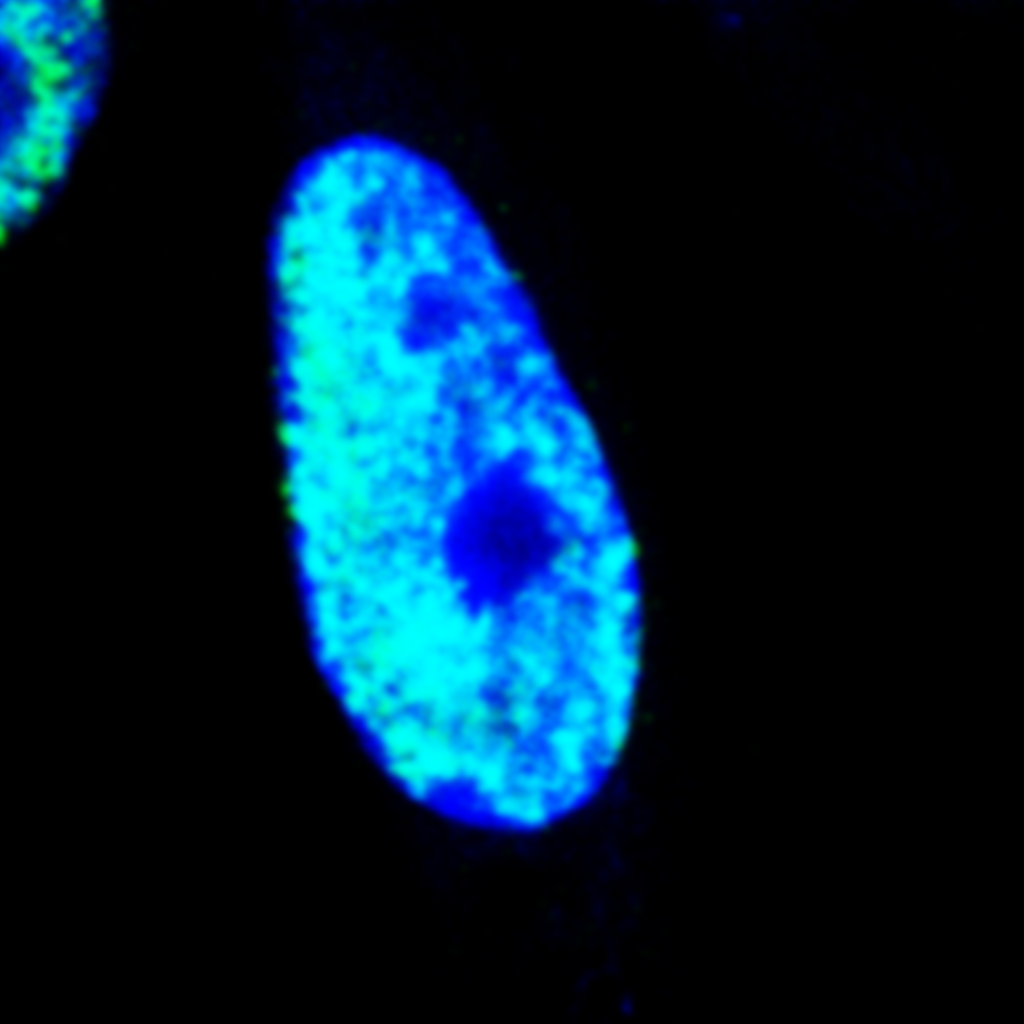

Supplement: Supplementary file 5 — Source data Fig. 3 [file 44318_2024_169_MOESM5_ESM.zip › SD_Figure_3.zip/Figure 3/3F/+RO/3F_Inter.tif]

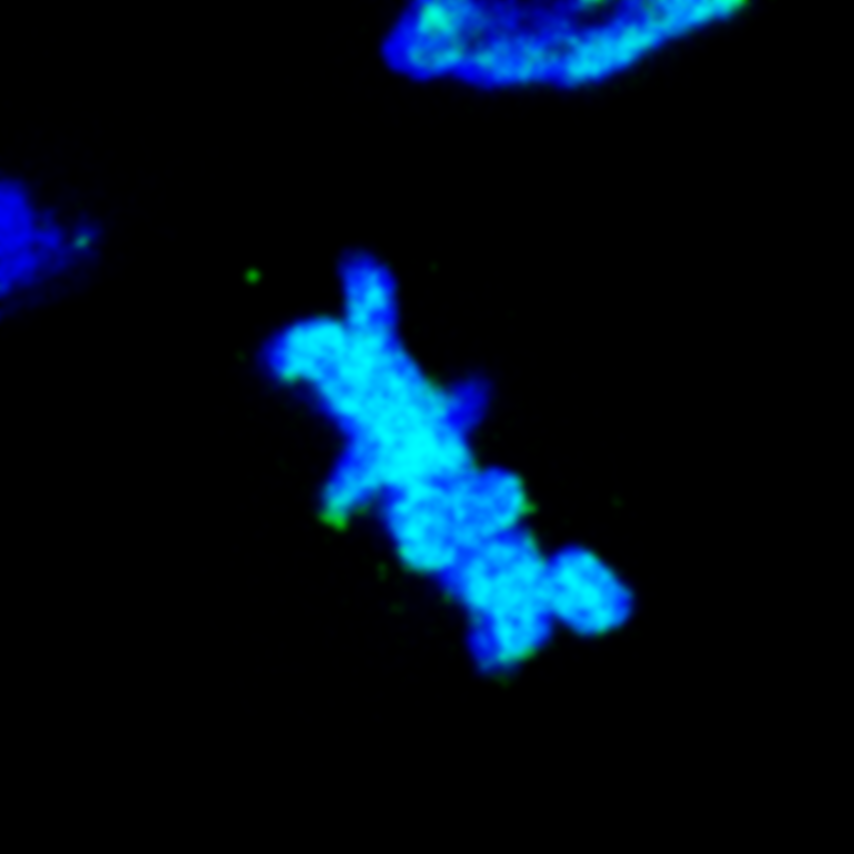

Supplement: Supplementary file 5 — Source data Fig. 3 [file 44318_2024_169_MOESM5_ESM.zip › SD_Figure_3.zip/Figure 3/3F/+RO/3F_meta.tif]

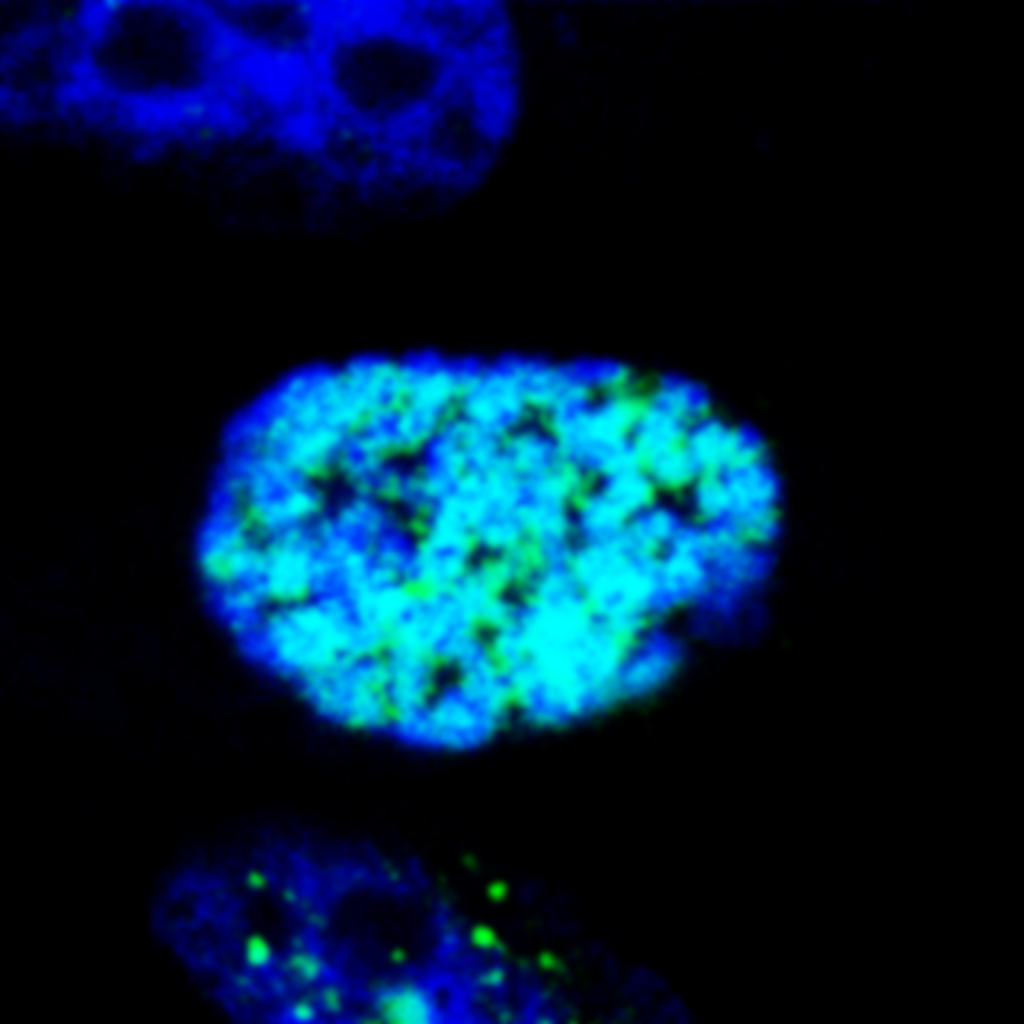

Supplement: Supplementary file 5 — Source data Fig. 3 [file 44318_2024_169_MOESM5_ESM.zip › SD_Figure_3.zip/Figure 3/3F/+RO/3F_pro.tif]

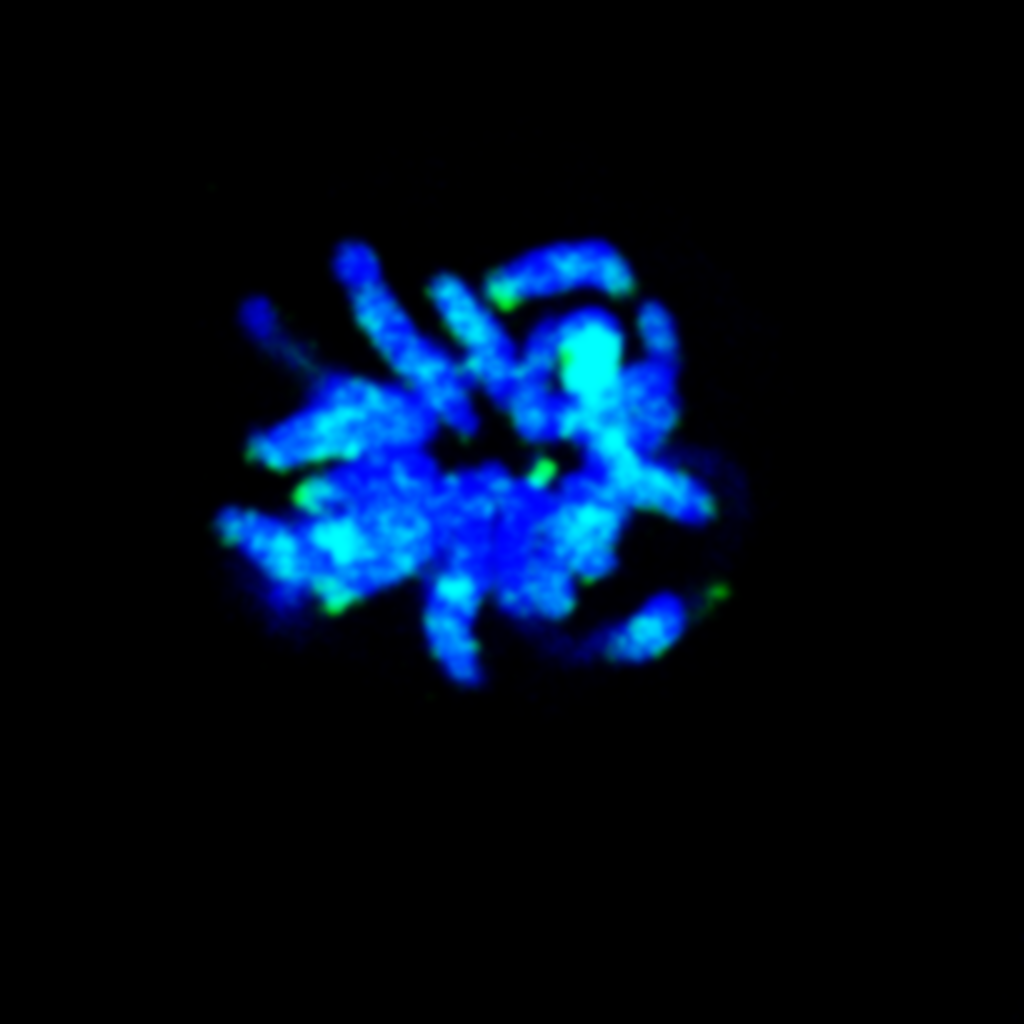

Supplement: Supplementary file 5 — Source data Fig. 3 [file 44318_2024_169_MOESM5_ESM.zip › SD_Figure_3.zip/Figure 3/3F/+RO/3F_prometa.tif]

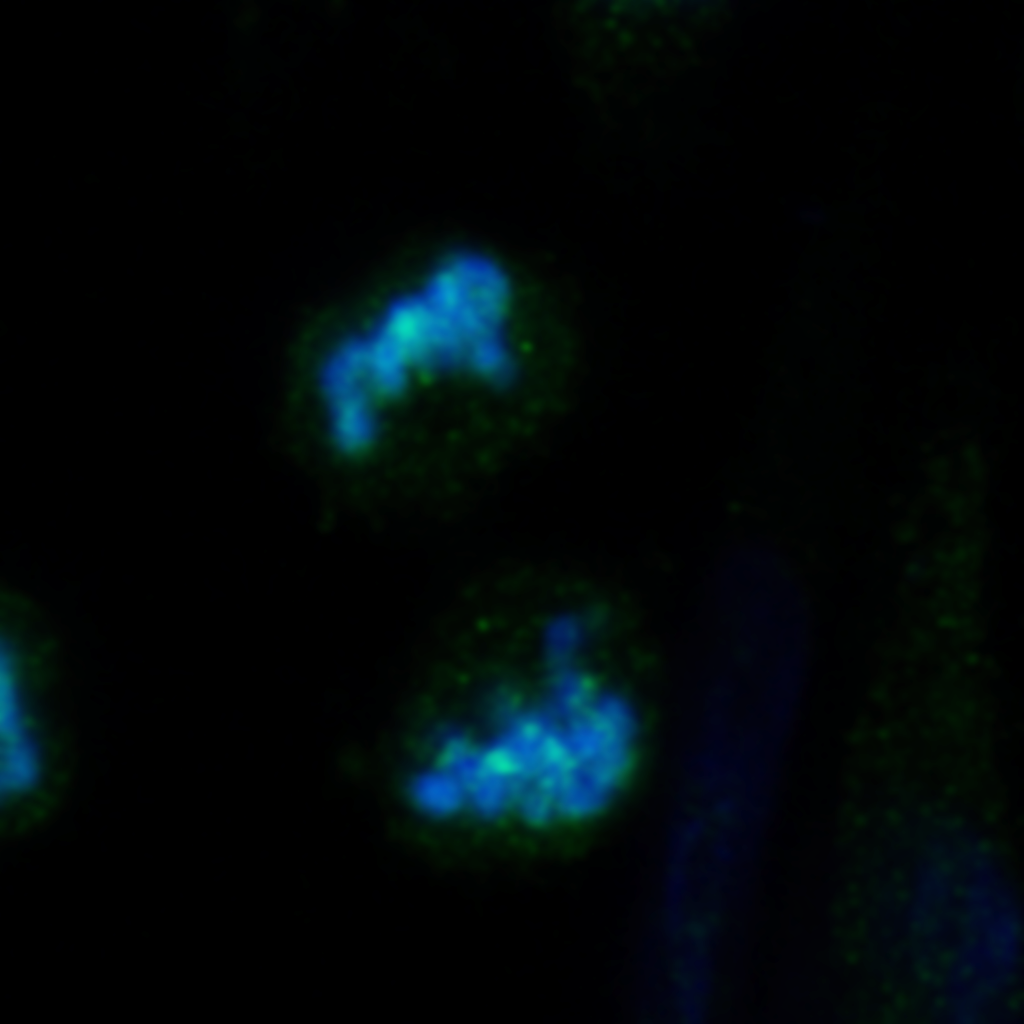

Supplement: Supplementary file 5 — Source data Fig. 3 [file 44318_2024_169_MOESM5_ESM.zip › SD_Figure_3.zip/Figure 3/3F/+RO/3F_telo.tif]

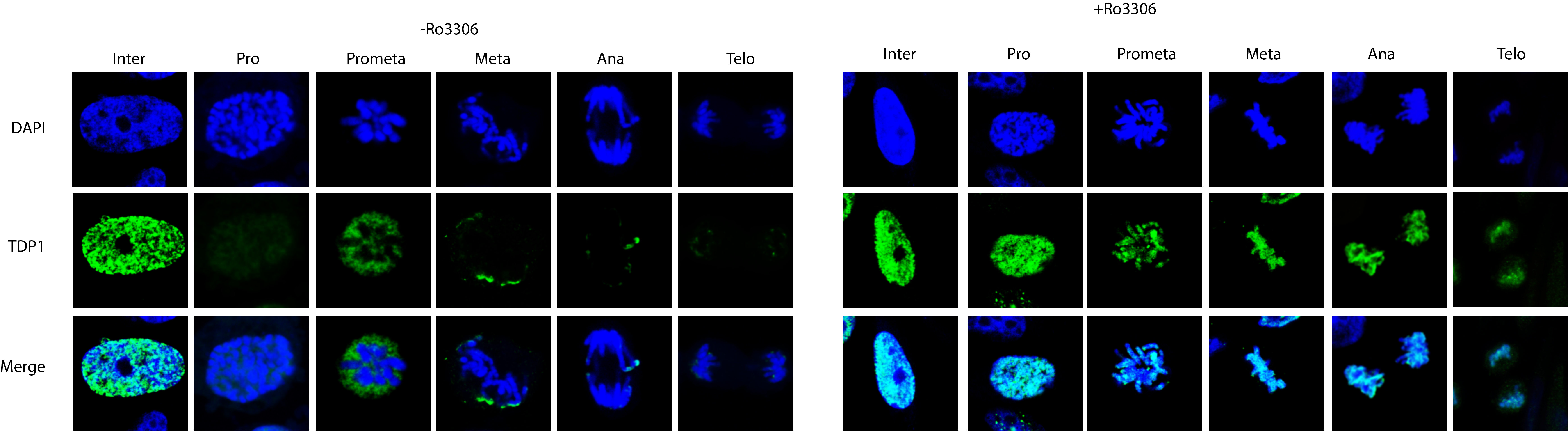

Supplement: Supplementary file 5 — Source data Fig. 3 [file 44318_2024_169_MOESM5_ESM.zip › SD_Figure_3.zip/Figure 3/3F/Fig 3F.tif]

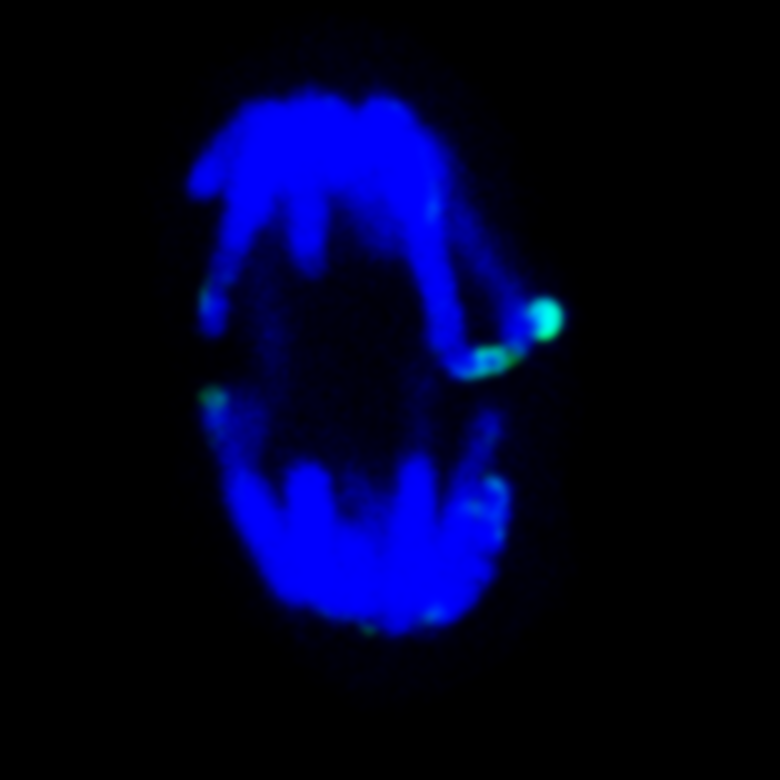

Supplement: Supplementary file 5 — Source data Fig. 3 [file 44318_2024_169_MOESM5_ESM.zip › SD_Figure_3.zip/Figure 3/3F/-RO/3F_Anaphase.tif]

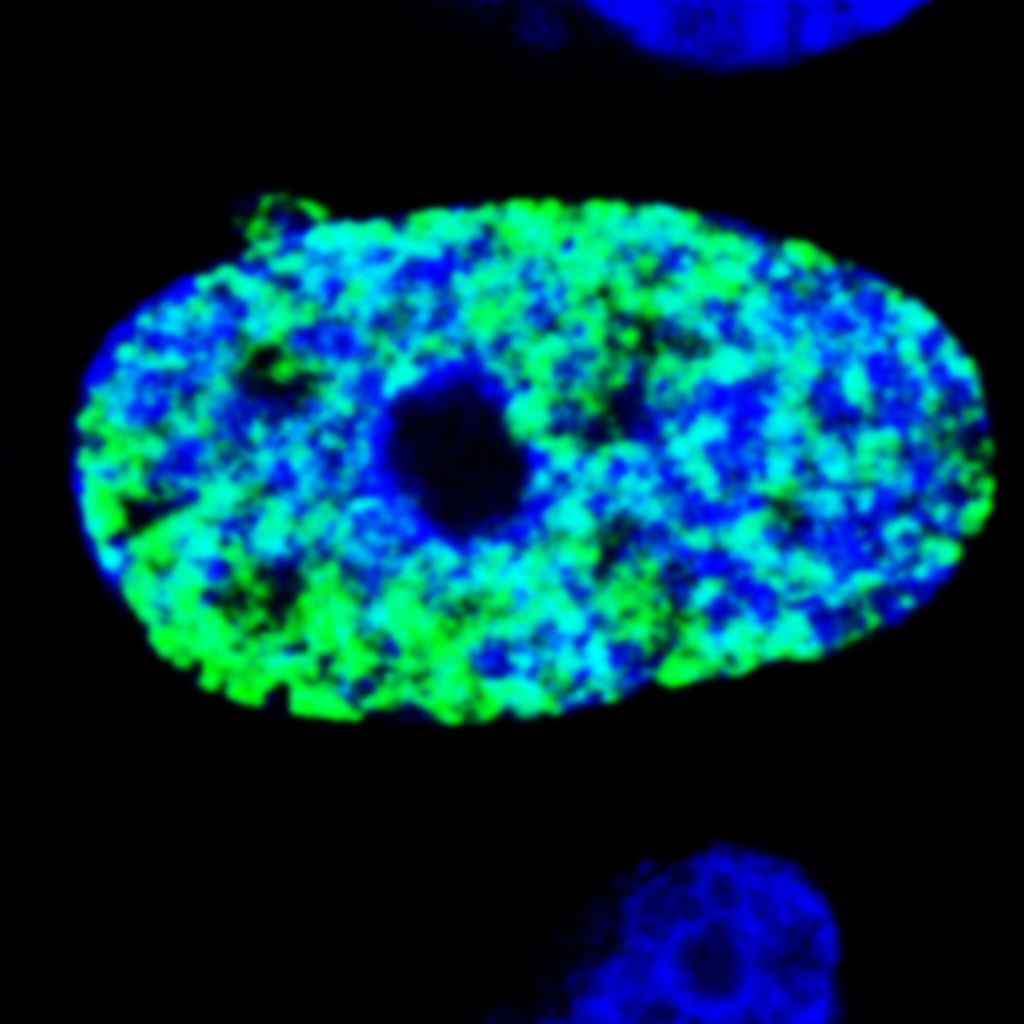

Supplement: Supplementary file 5 — Source data Fig. 3 [file 44318_2024_169_MOESM5_ESM.zip › SD_Figure_3.zip/Figure 3/3F/-RO/3F_Interphase.tif]

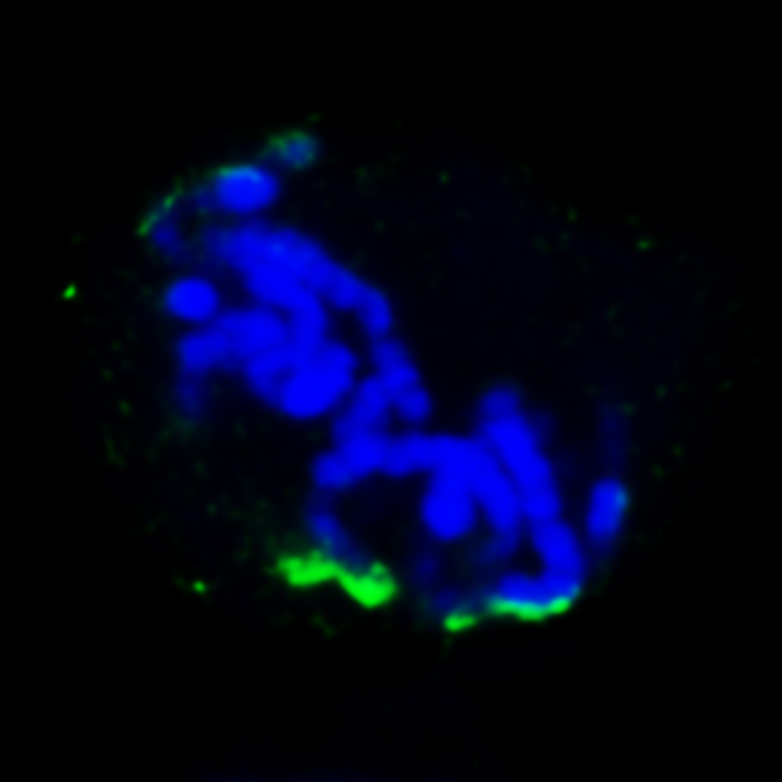

Supplement: Supplementary file 5 — Source data Fig. 3 [file 44318_2024_169_MOESM5_ESM.zip › SD_Figure_3.zip/Figure 3/3F/-RO/3F_Metaphase.tif]

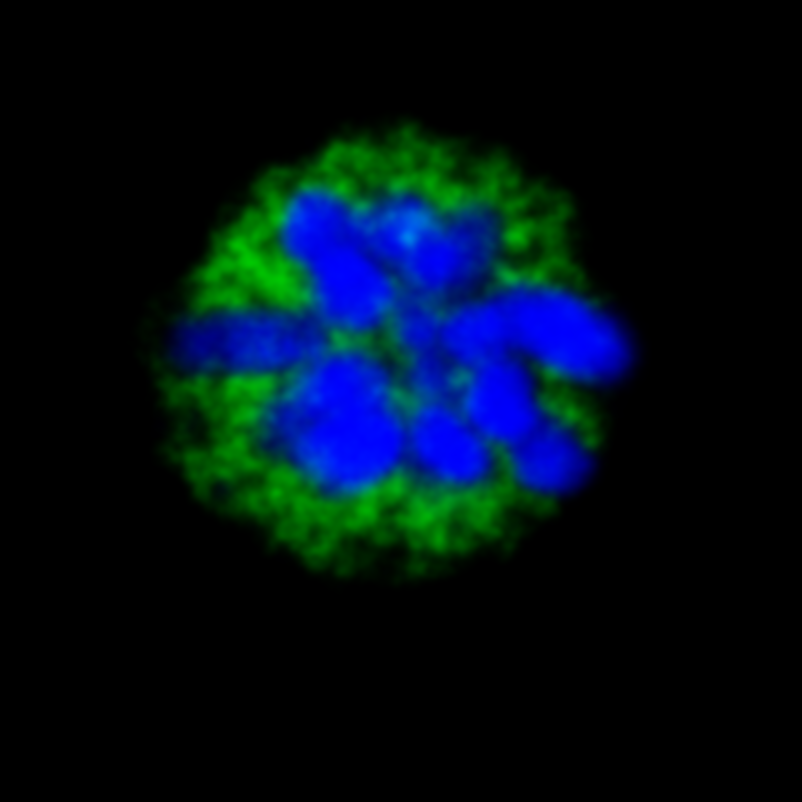

Supplement: Supplementary file 5 — Source data Fig. 3 [file 44318_2024_169_MOESM5_ESM.zip › SD_Figure_3.zip/Figure 3/3F/-RO/3F_prometaphase.tif]

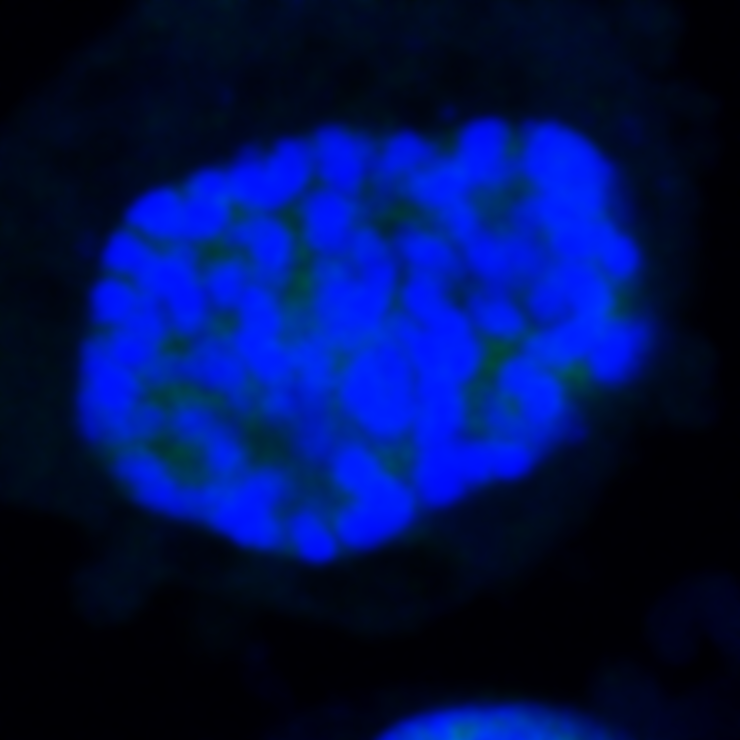

Supplement: Supplementary file 5 — Source data Fig. 3 [file 44318_2024_169_MOESM5_ESM.zip › SD_Figure_3.zip/Figure 3/3F/-RO/3F_Prophase.tif]

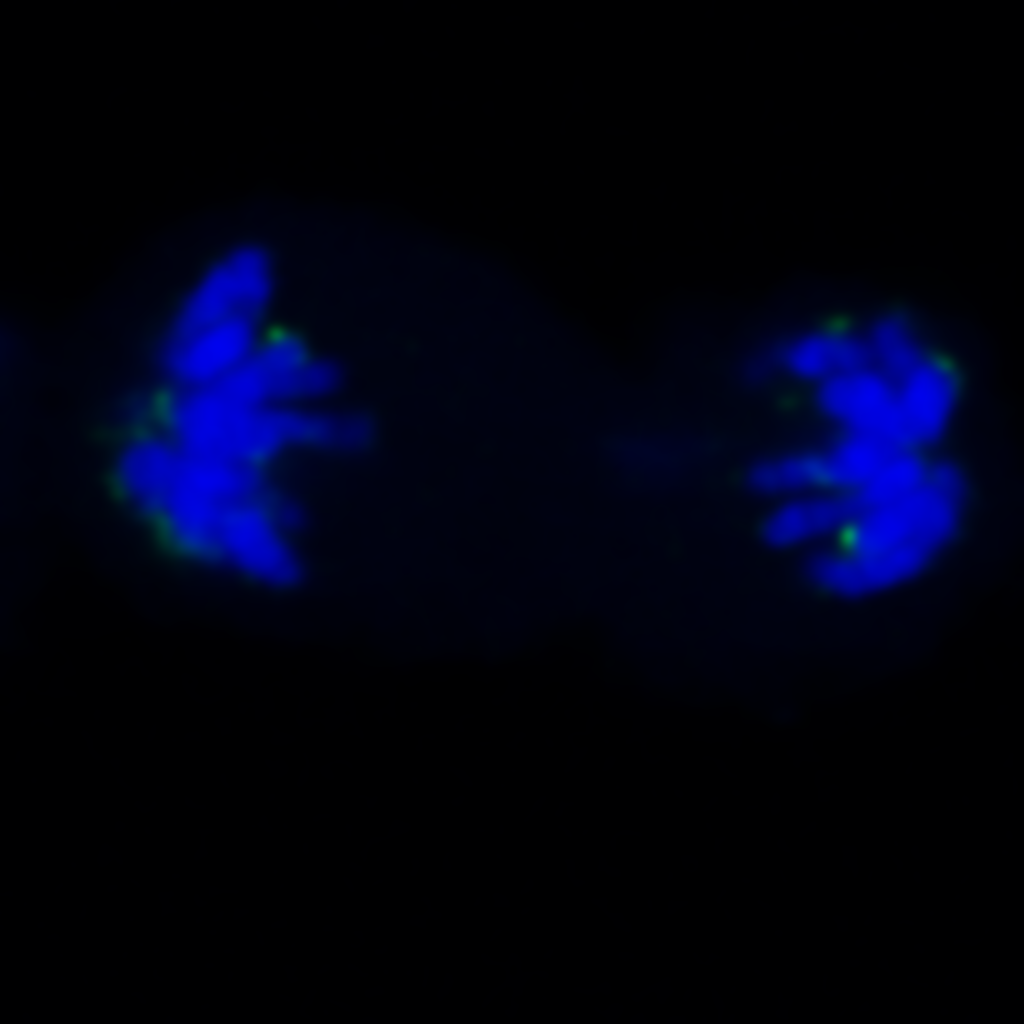

Supplement: Supplementary file 5 — Source data Fig. 3 [file 44318_2024_169_MOESM5_ESM.zip › SD_Figure_3.zip/Figure 3/3F/-RO/3F_Telophase.tif]

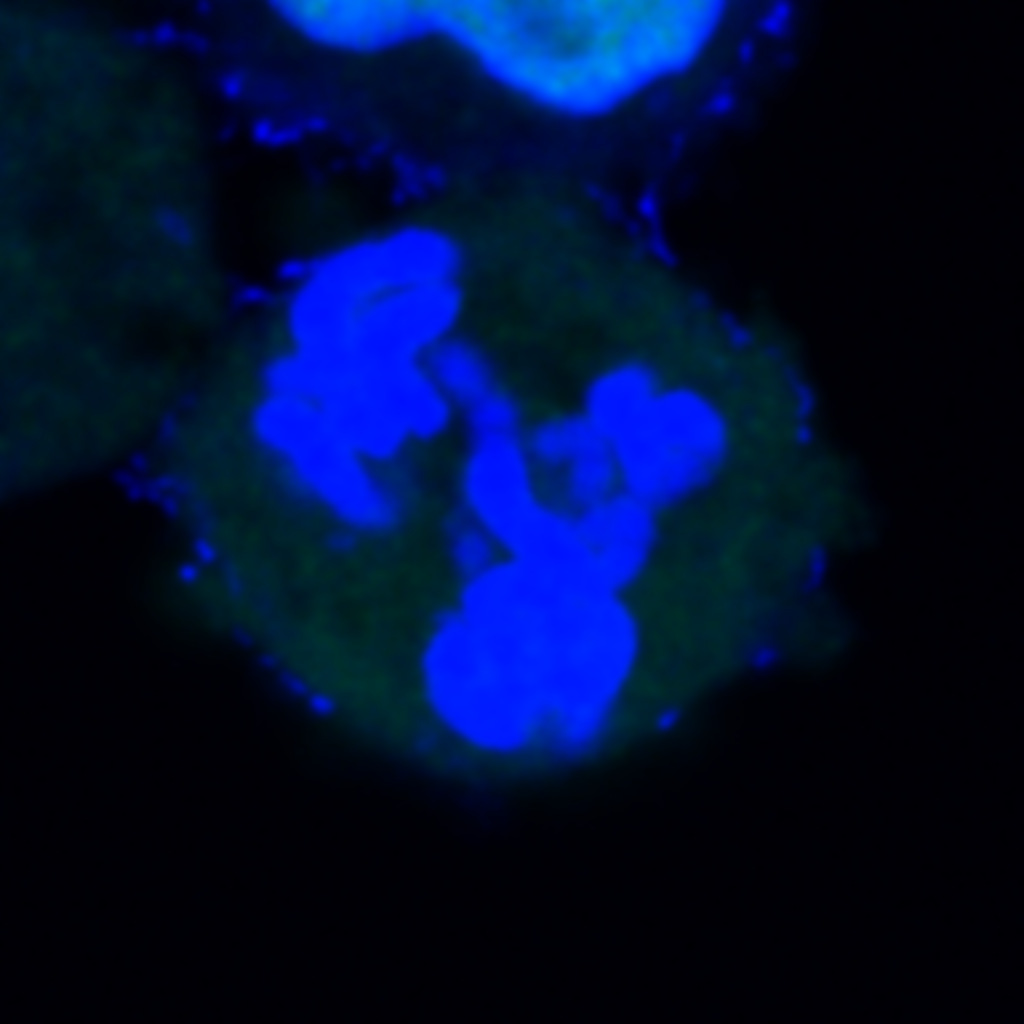

Supplement: Supplementary file 5 — Source data Fig. 3 [file 44318_2024_169_MOESM5_ESM.zip › SD_Figure_3.zip/Figure 3/3I/+CPT/Anaphase.tif]

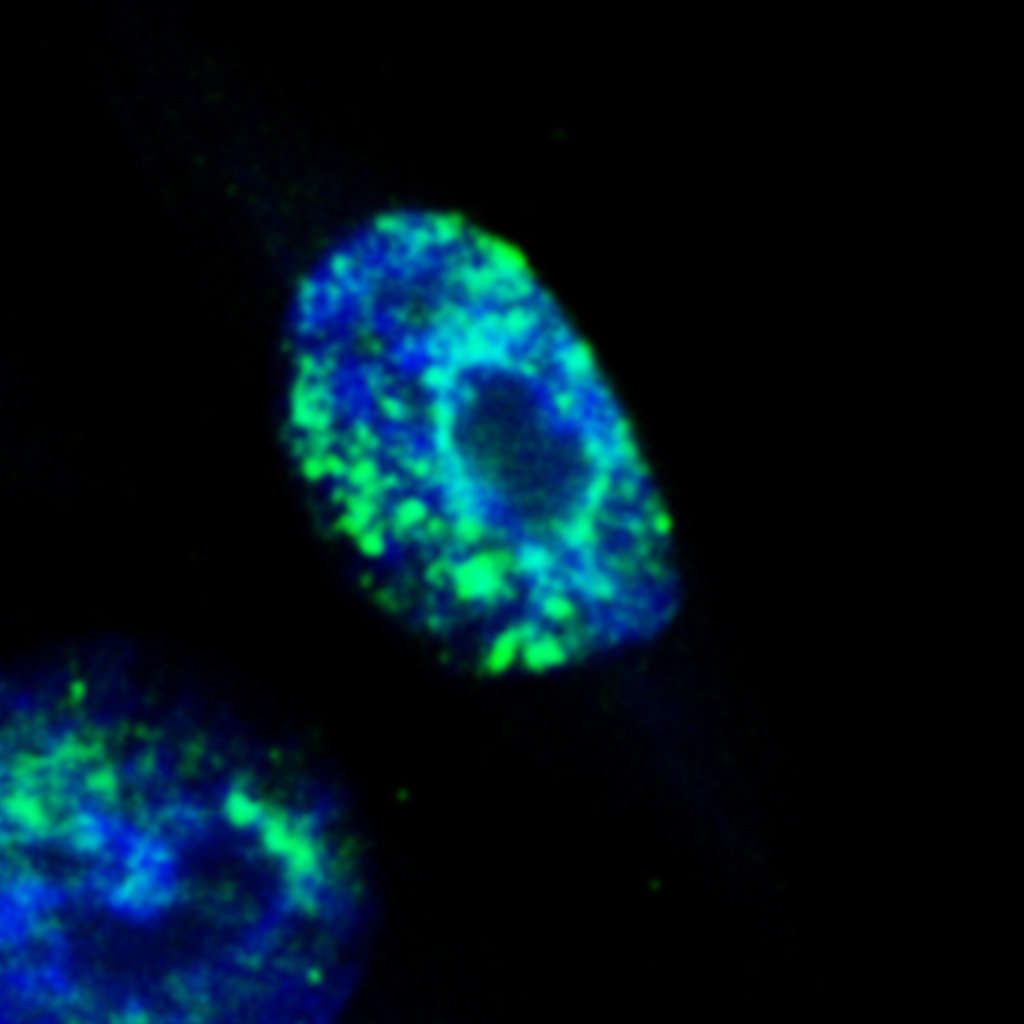

Supplement: Supplementary file 5 — Source data Fig. 3 [file 44318_2024_169_MOESM5_ESM.zip › SD_Figure_3.zip/Figure 3/3I/+CPT/Inter.tif]

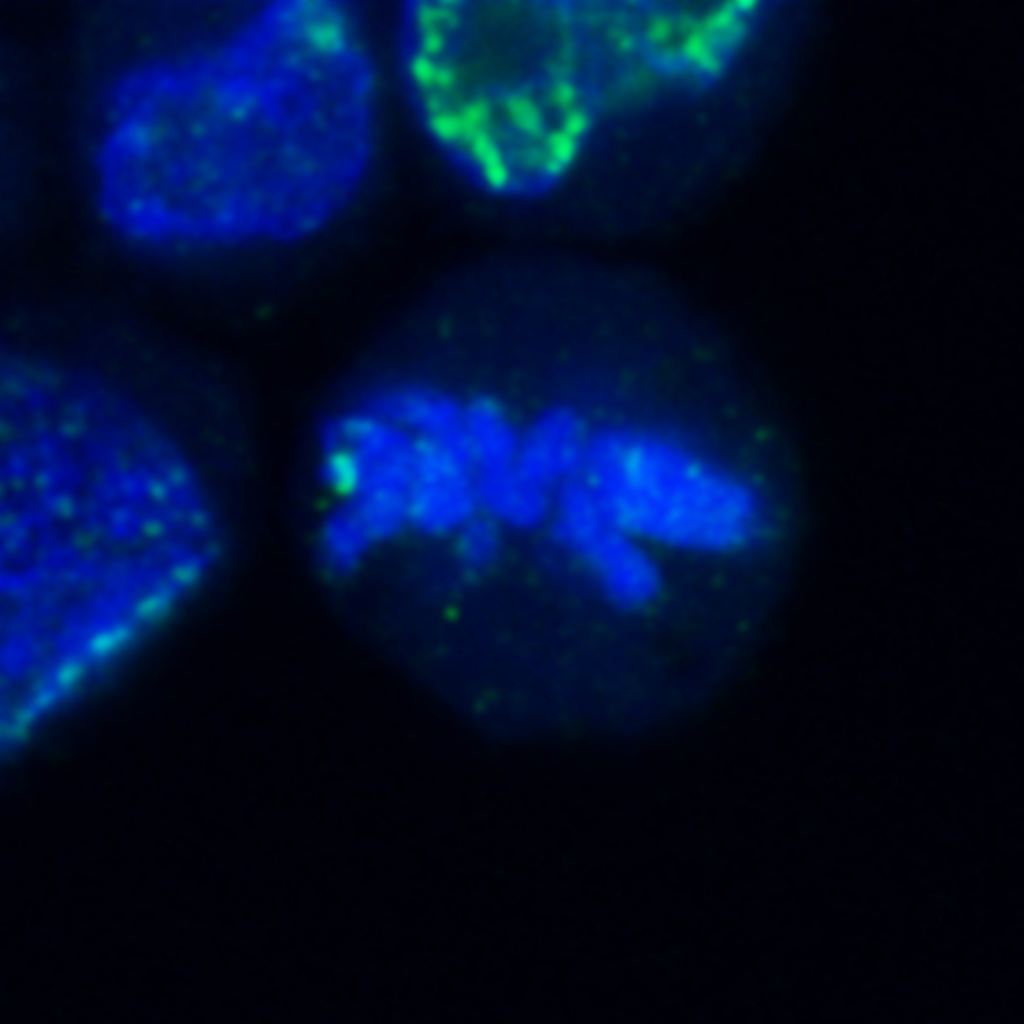

Supplement: Supplementary file 5 — Source data Fig. 3 [file 44318_2024_169_MOESM5_ESM.zip › SD_Figure_3.zip/Figure 3/3I/+CPT/meta.tif]

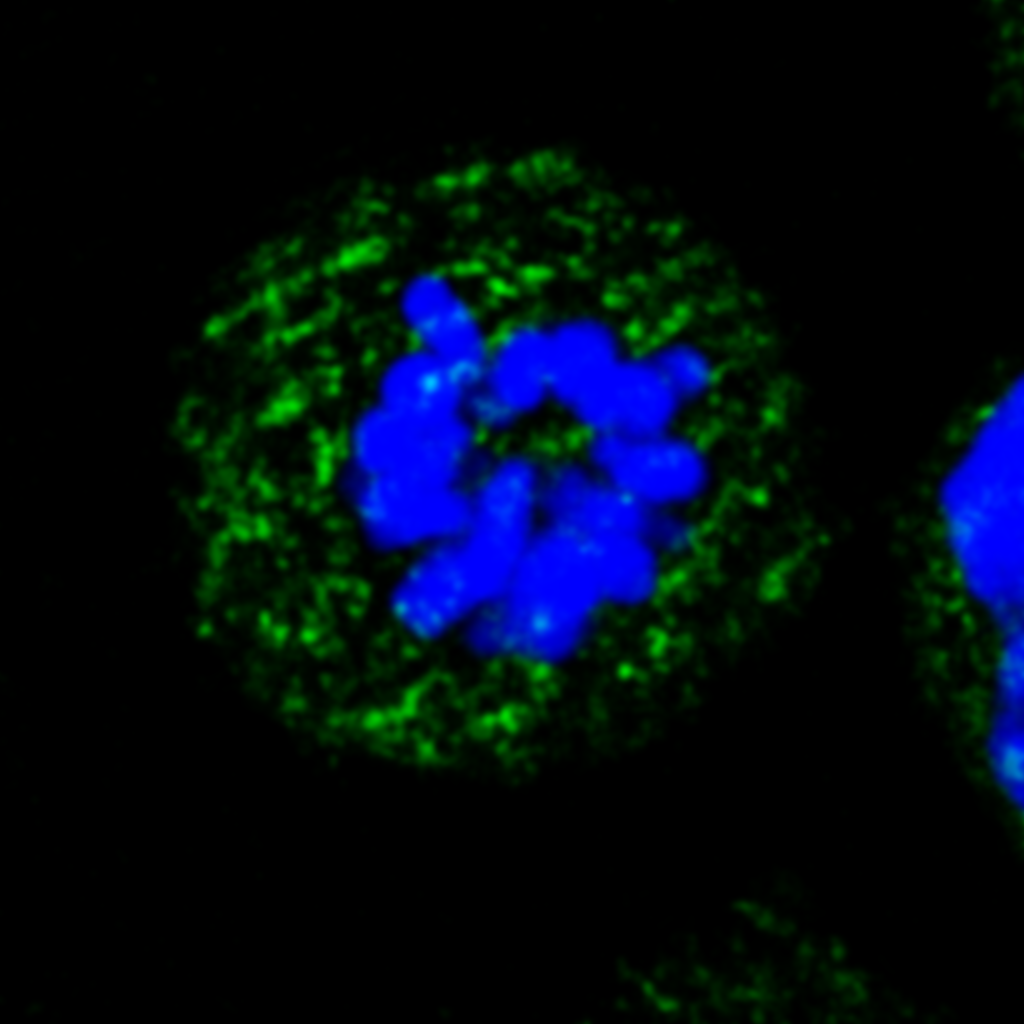

Supplement: Supplementary file 5 — Source data Fig. 3 [file 44318_2024_169_MOESM5_ESM.zip › SD_Figure_3.zip/Figure 3/3I/+CPT/prometa.tif]

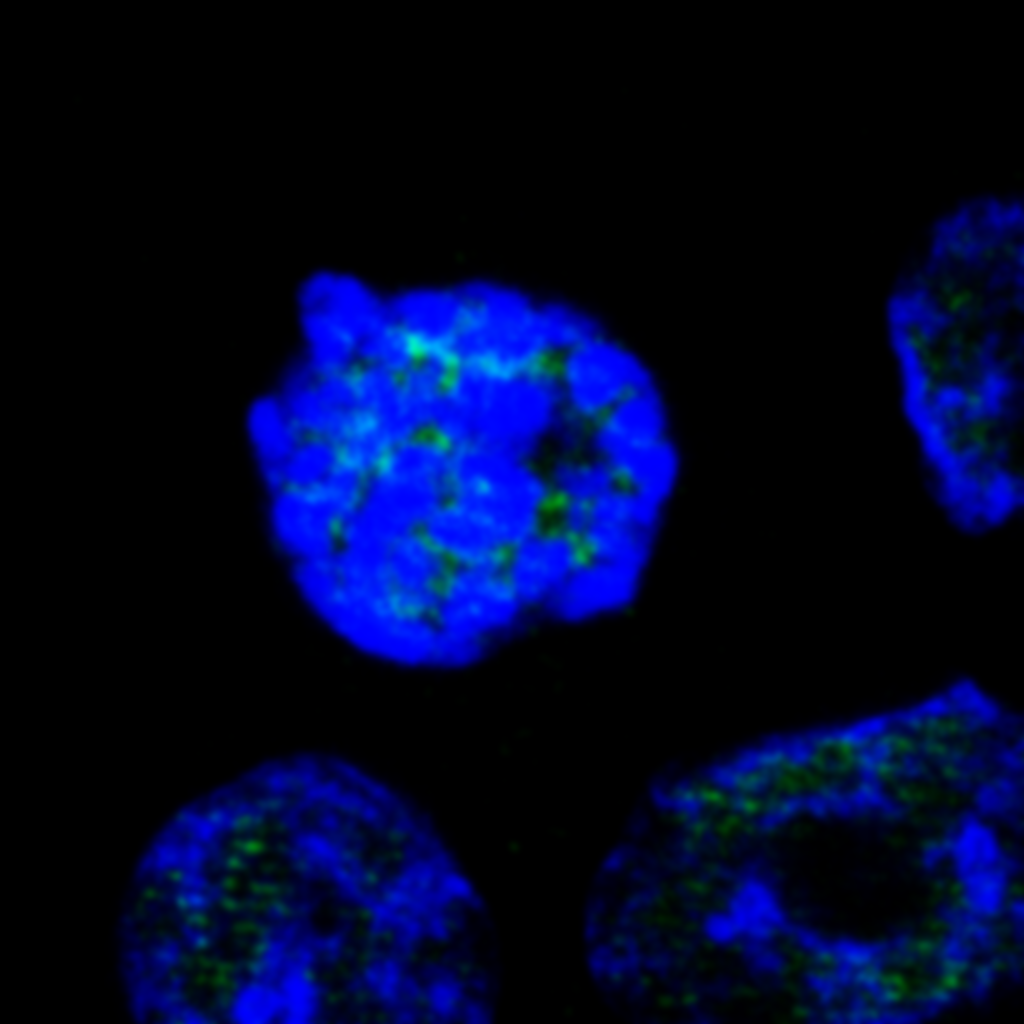

Supplement: Supplementary file 5 — Source data Fig. 3 [file 44318_2024_169_MOESM5_ESM.zip › SD_Figure_3.zip/Figure 3/3I/+CPT/Prophase.tif]

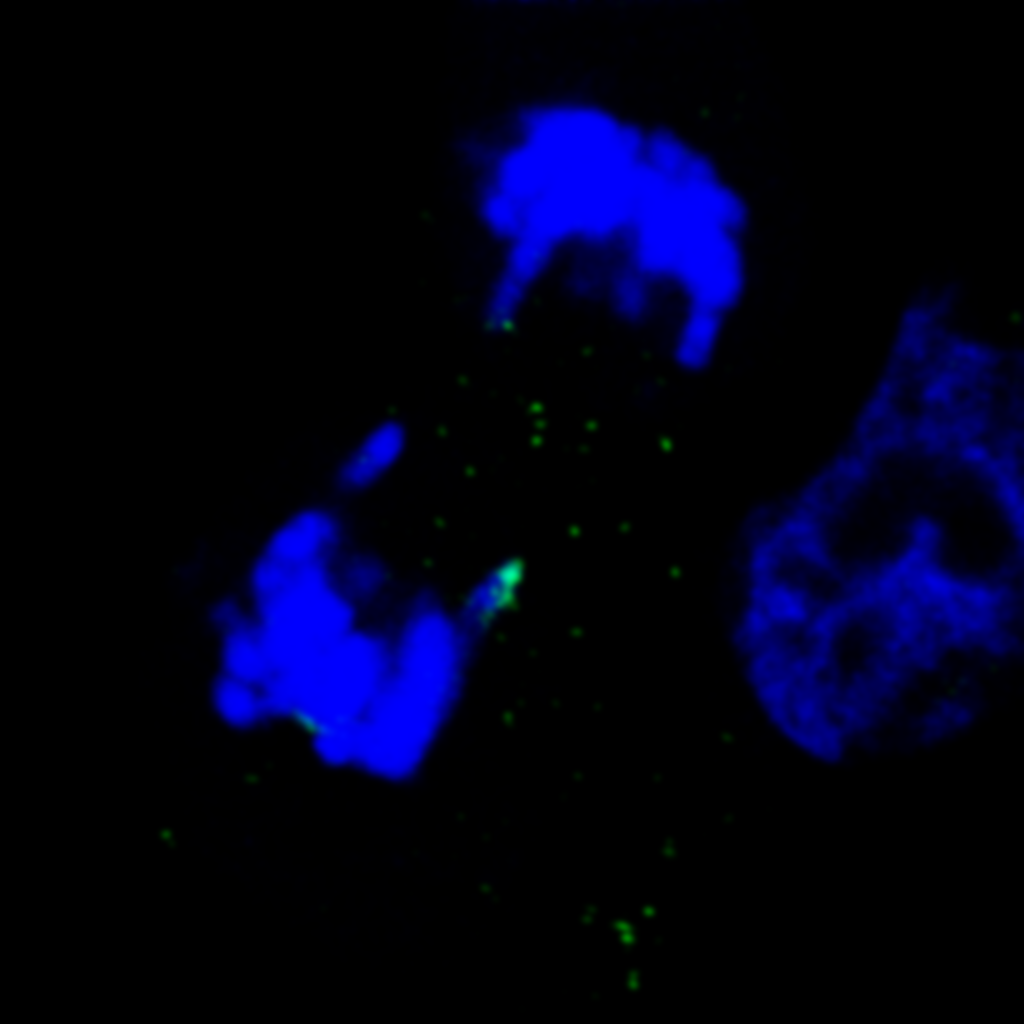

Supplement: Supplementary file 5 — Source data Fig. 3 [file 44318_2024_169_MOESM5_ESM.zip › SD_Figure_3.zip/Figure 3/3I/+CPT/telophase.tif]

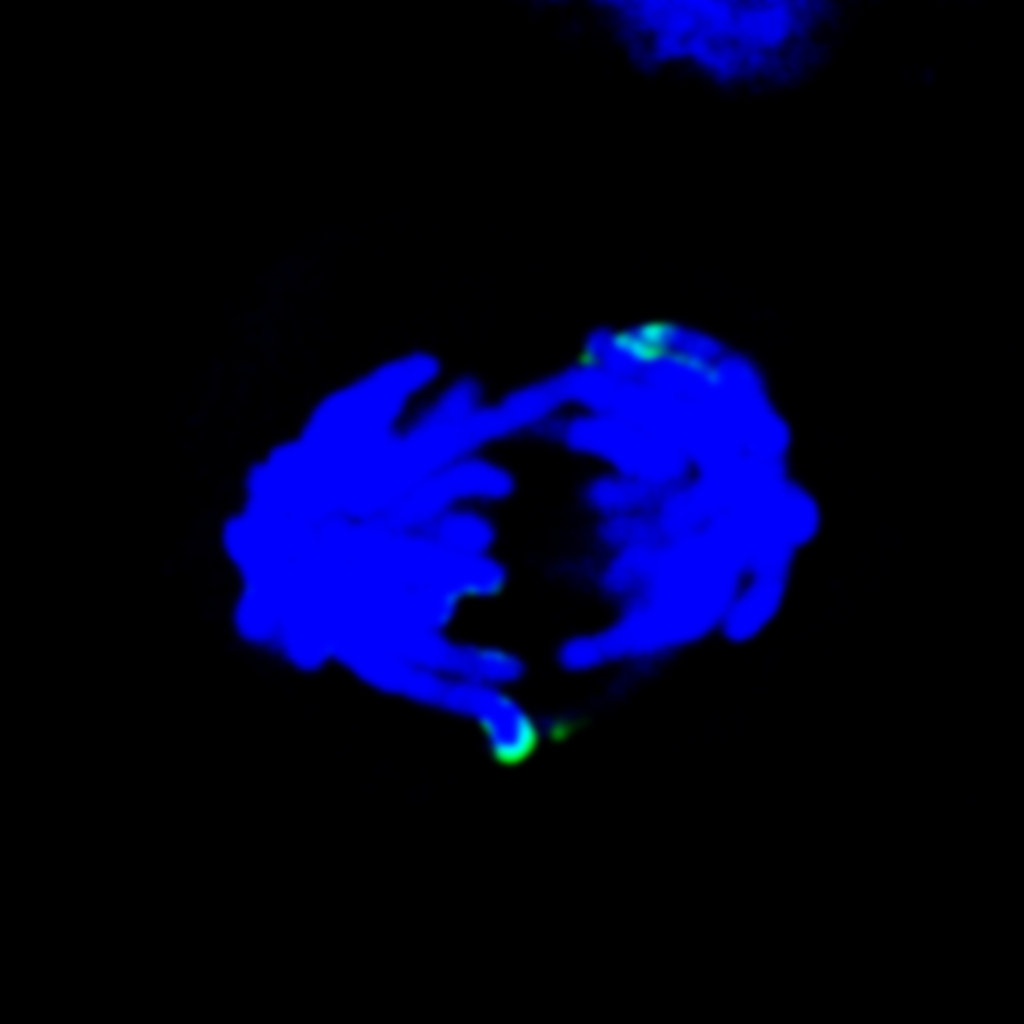

Supplement: Supplementary file 5 — Source data Fig. 3 [file 44318_2024_169_MOESM5_ESM.zip › SD_Figure_3.zip/Figure 3/3I/-CPT/ana.tif]

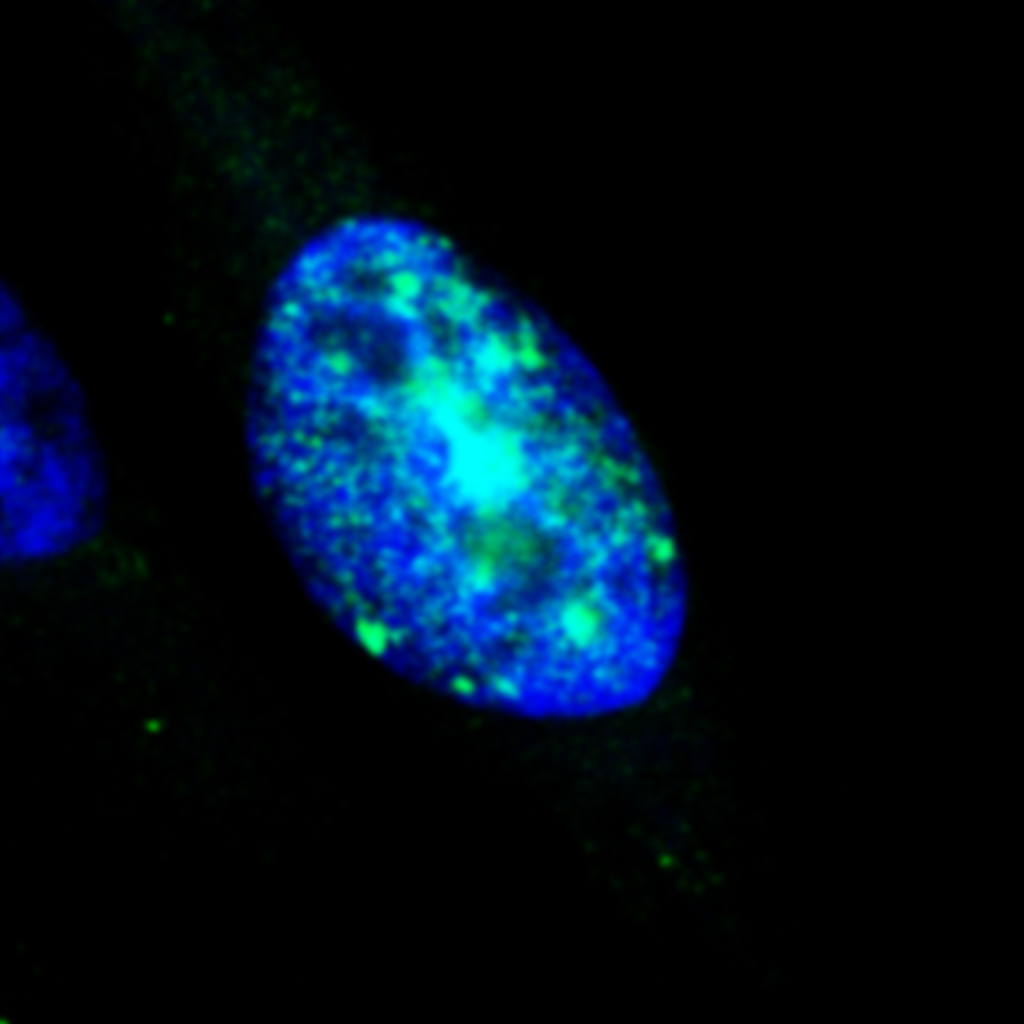

Supplement: Supplementary file 5 — Source data Fig. 3 [file 44318_2024_169_MOESM5_ESM.zip › SD_Figure_3.zip/Figure 3/3I/-CPT/Inter.tif]

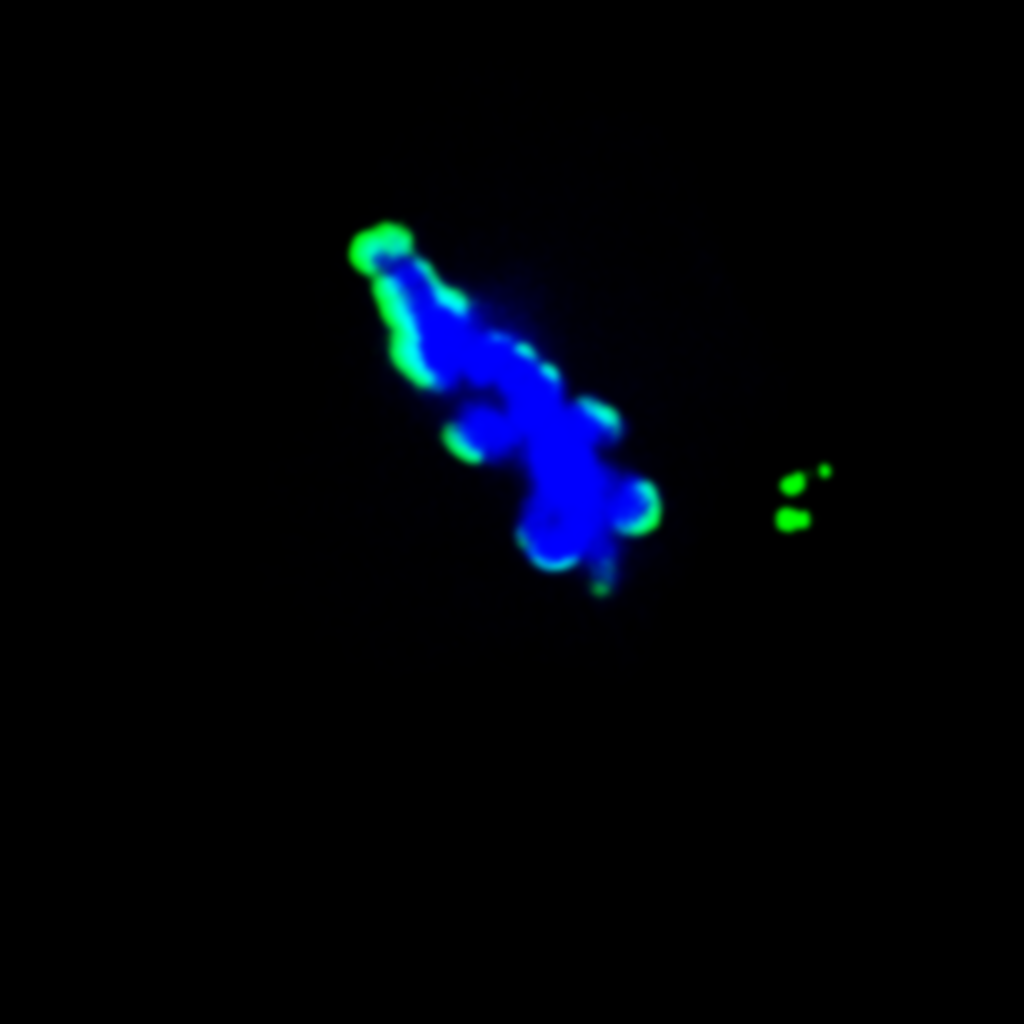

Supplement: Supplementary file 5 — Source data Fig. 3 [file 44318_2024_169_MOESM5_ESM.zip › SD_Figure_3.zip/Figure 3/3I/-CPT/Metaphase.tif]
