# Supplementary figures and images for: TDP1 phosphorylation by CDK1 in mitosis promotes MUS81-dependent repair of trapped Top1-DNA covalent complexes (part 2 of 3)
Source: EMBO J. 2024 Jul 16;43(17):3710–32. doi: 10.1038/s44318-024-00169-3 (PMC11377750; doi:10.1038/s44318-024-00169-3)

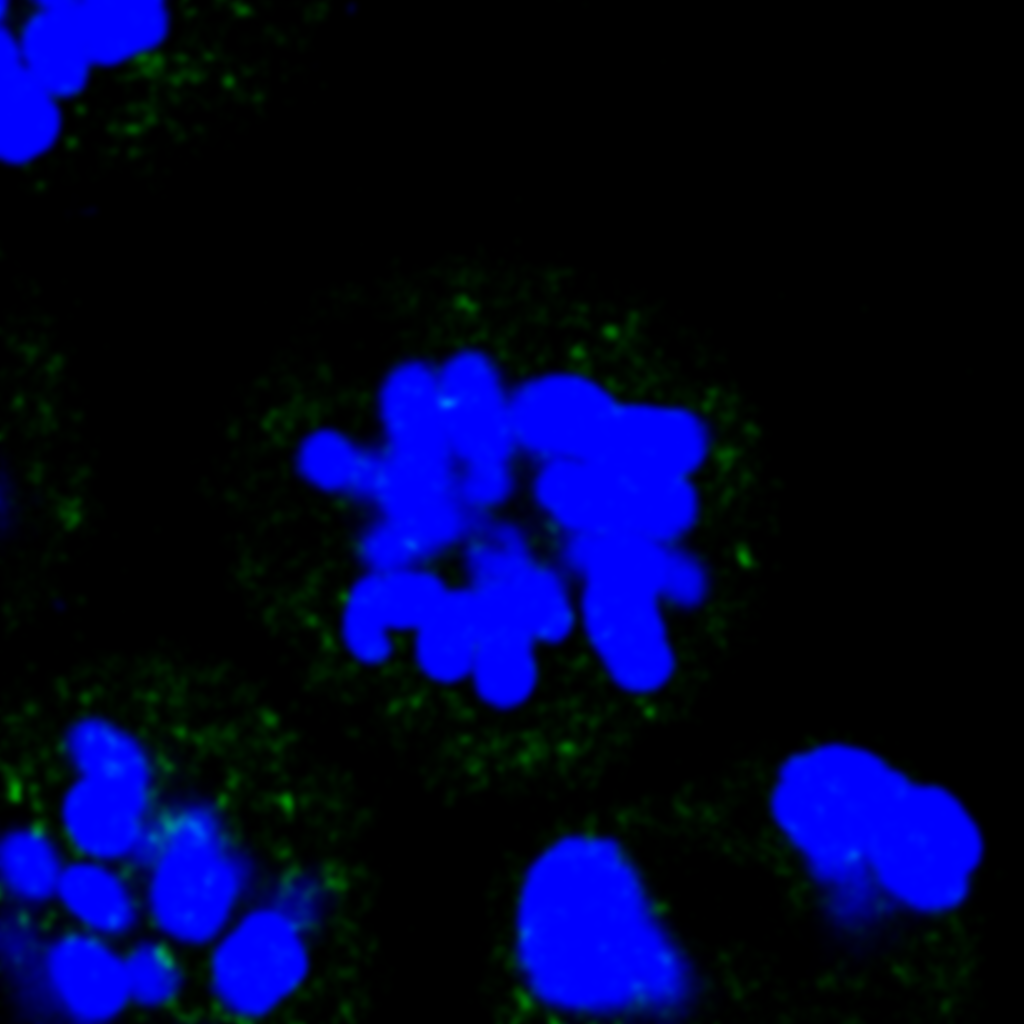

Supplement: Supplementary file 5 — Source data Fig. 3 [file 44318_2024_169_MOESM5_ESM.zip › SD_Figure_3.zip/Figure 3/3I/-CPT/prometa.tif]

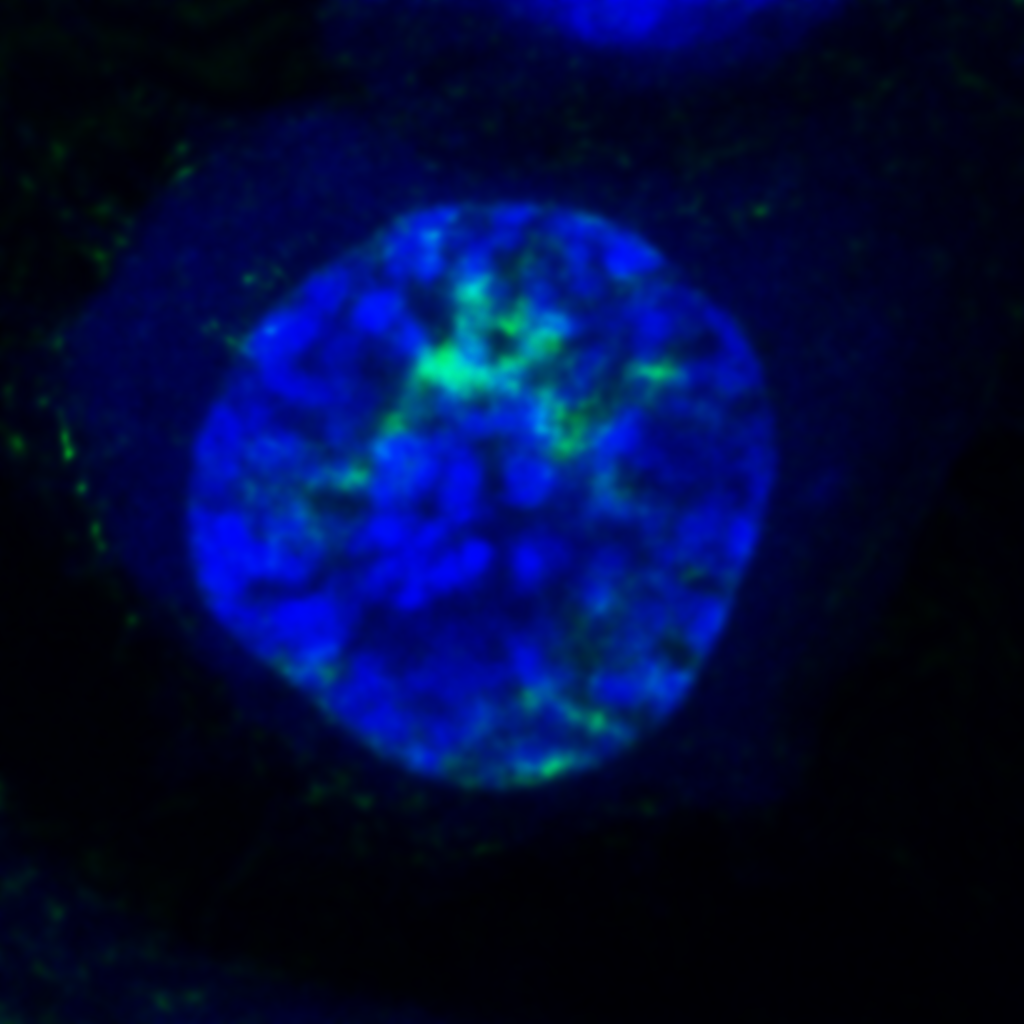

Supplement: Supplementary file 5 — Source data Fig. 3 [file 44318_2024_169_MOESM5_ESM.zip › SD_Figure_3.zip/Figure 3/3I/-CPT/prophase.tif]

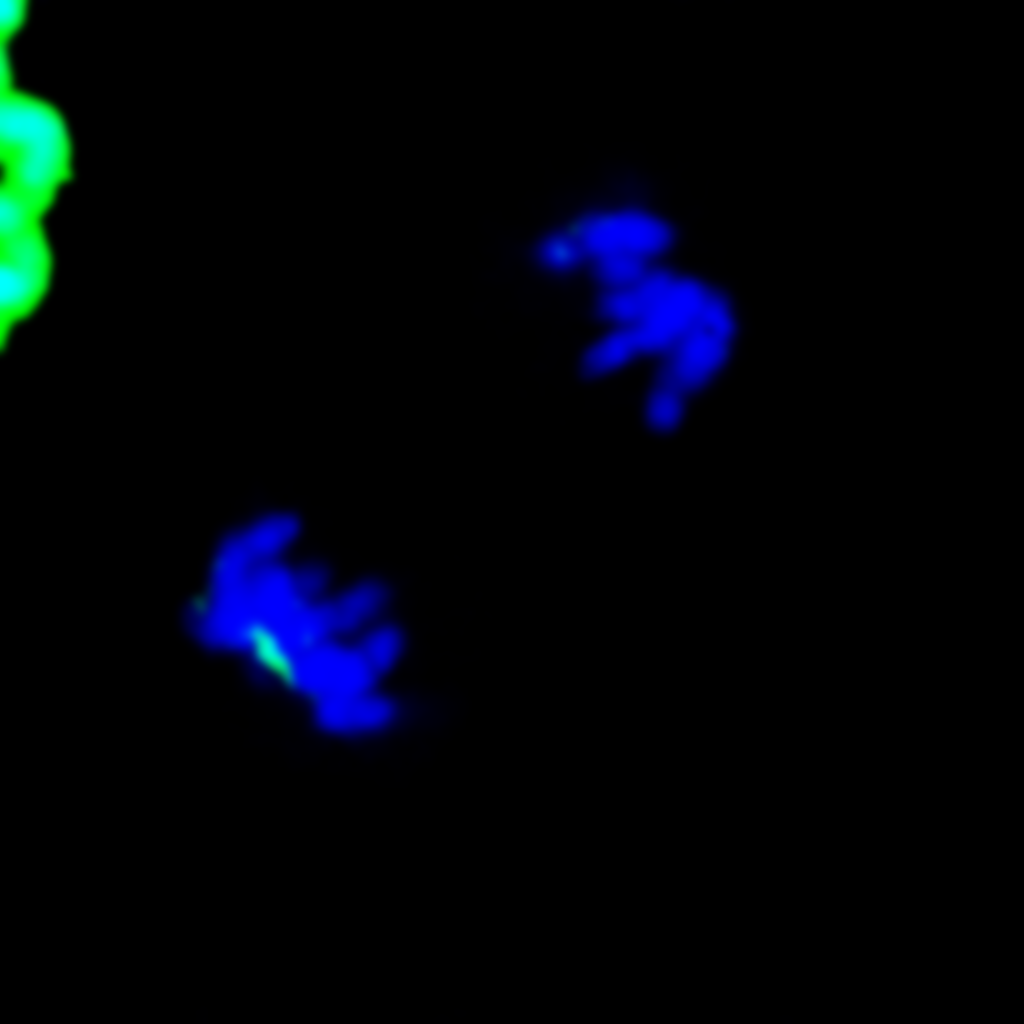

Supplement: Supplementary file 5 — Source data Fig. 3 [file 44318_2024_169_MOESM5_ESM.zip › SD_Figure_3.zip/Figure 3/3I/-CPT/Telo.tif]

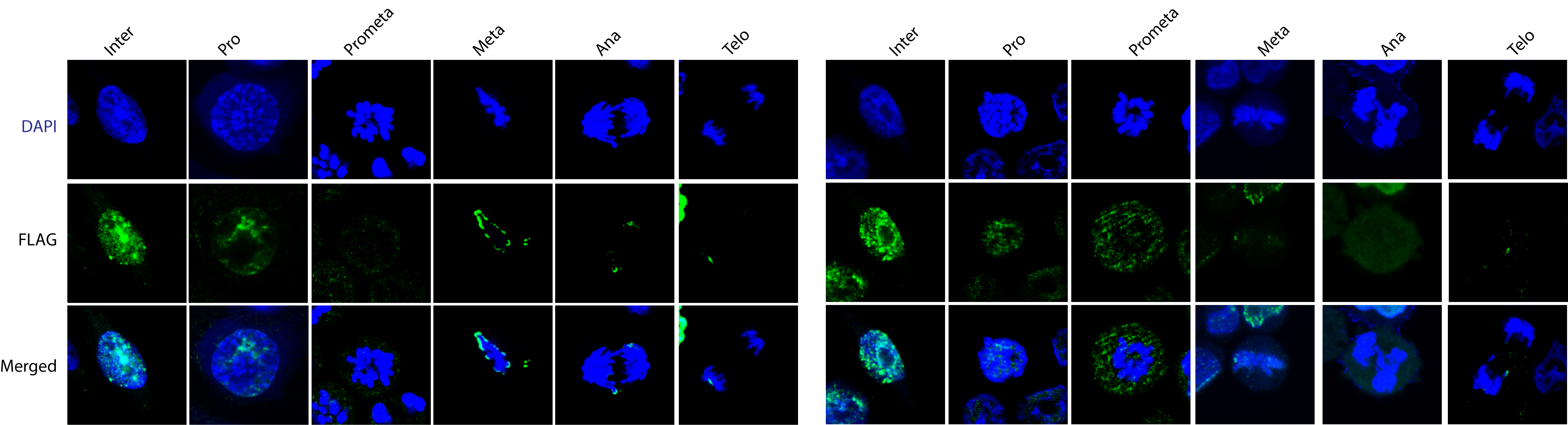

Supplement: Supplementary file 5 — Source data Fig. 3 [file 44318_2024_169_MOESM5_ESM.zip › SD_Figure_3.zip/Figure 3/3I/Fig 3I.tif]

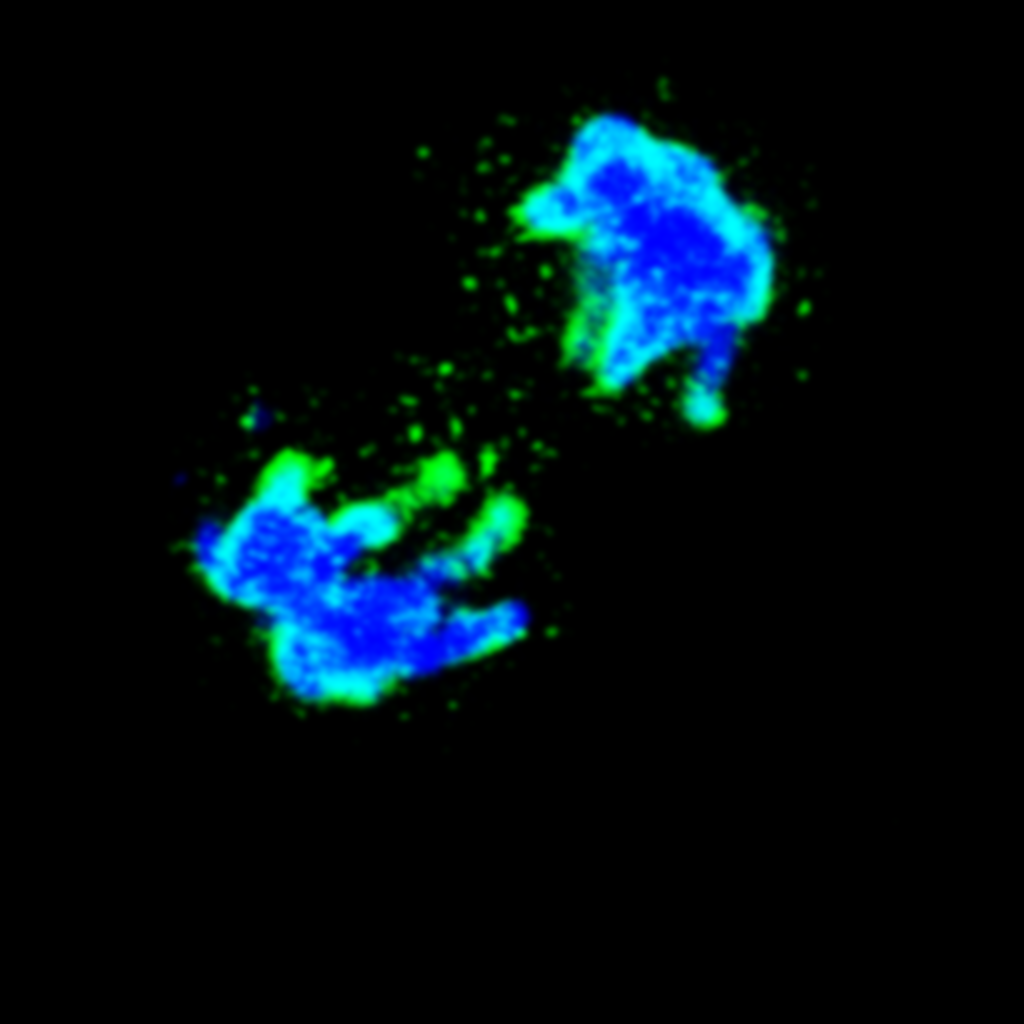

Supplement: Supplementary file 5 — Source data Fig. 3 [file 44318_2024_169_MOESM5_ESM.zip › SD_Figure_3.zip/Figure 3/3J/+CPT/anaphase.tif]

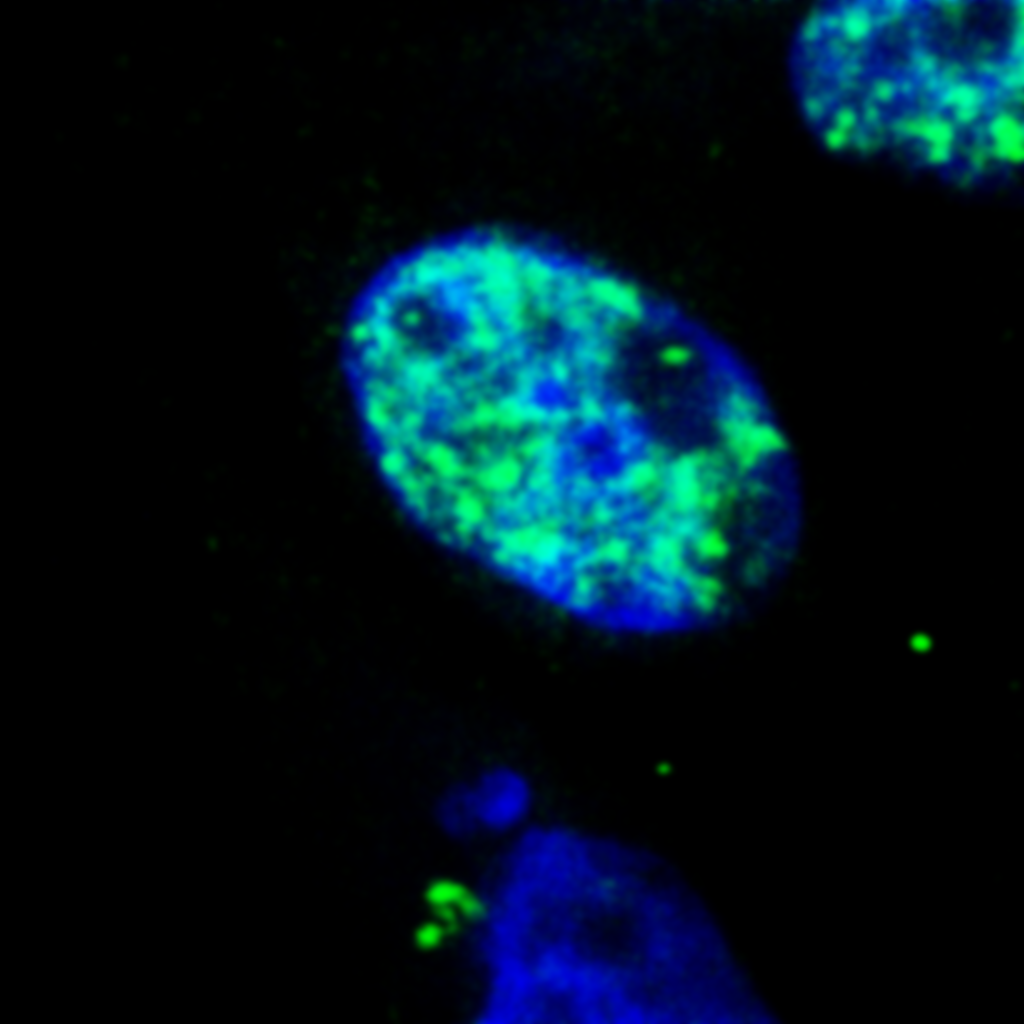

Supplement: Supplementary file 5 — Source data Fig. 3 [file 44318_2024_169_MOESM5_ESM.zip › SD_Figure_3.zip/Figure 3/3J/+CPT/Fig 3I_inter.tif]

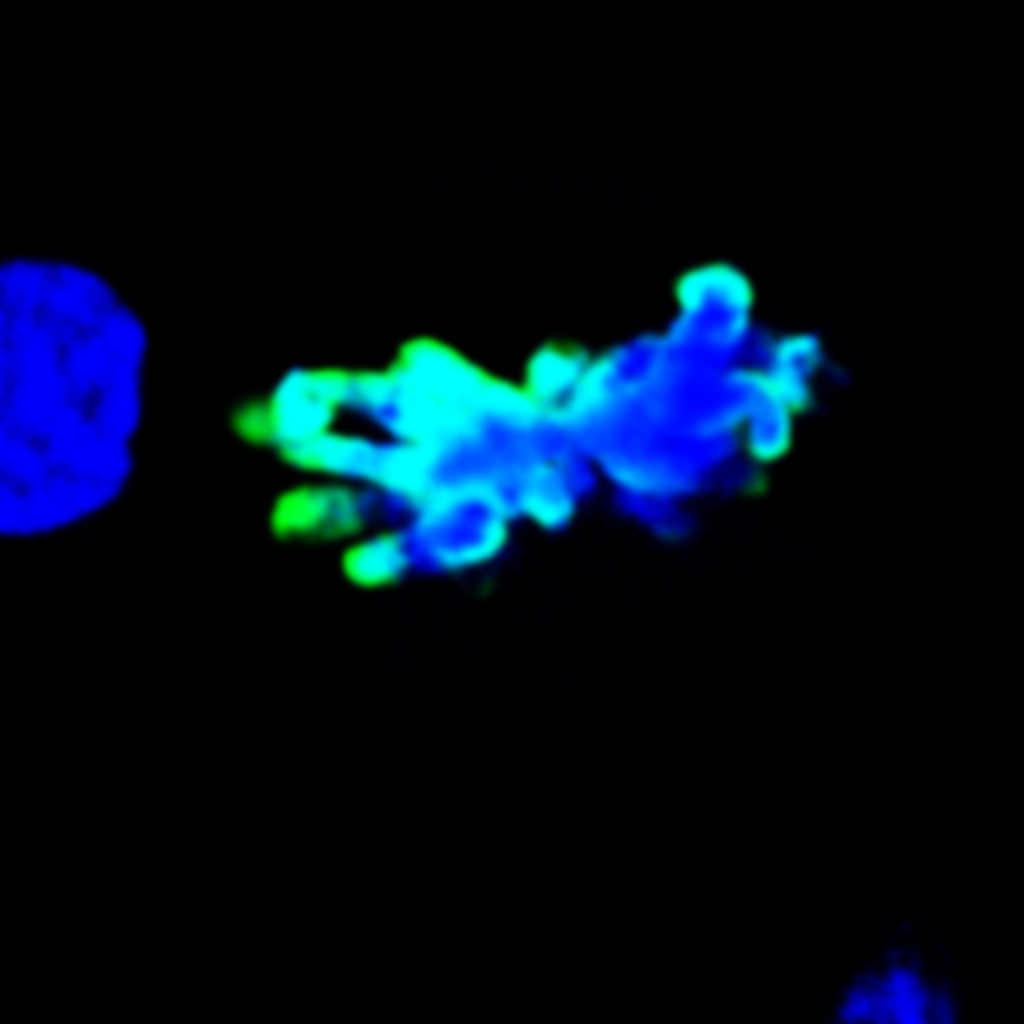

Supplement: Supplementary file 5 — Source data Fig. 3 [file 44318_2024_169_MOESM5_ESM.zip › SD_Figure_3.zip/Figure 3/3J/+CPT/metaphase.tif]

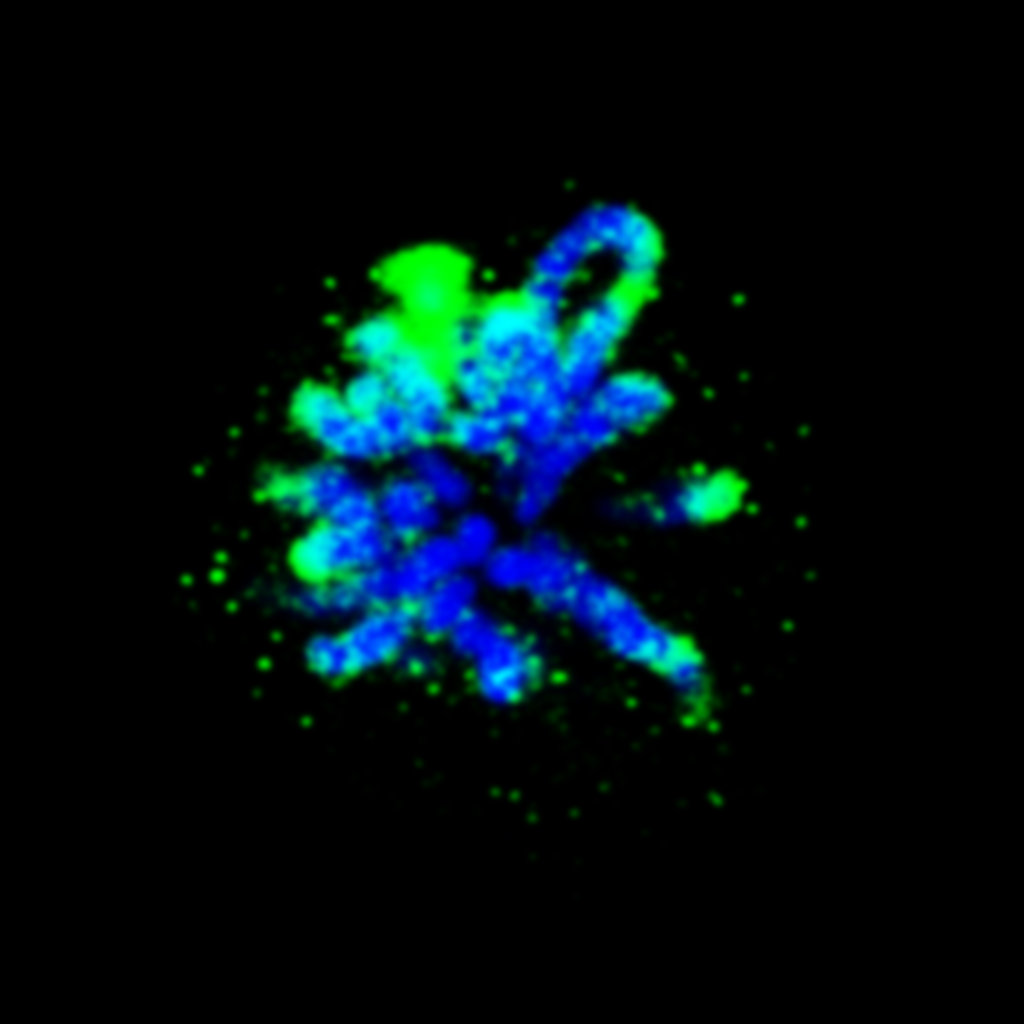

Supplement: Supplementary file 5 — Source data Fig. 3 [file 44318_2024_169_MOESM5_ESM.zip › SD_Figure_3.zip/Figure 3/3J/+CPT/prometa.tif]

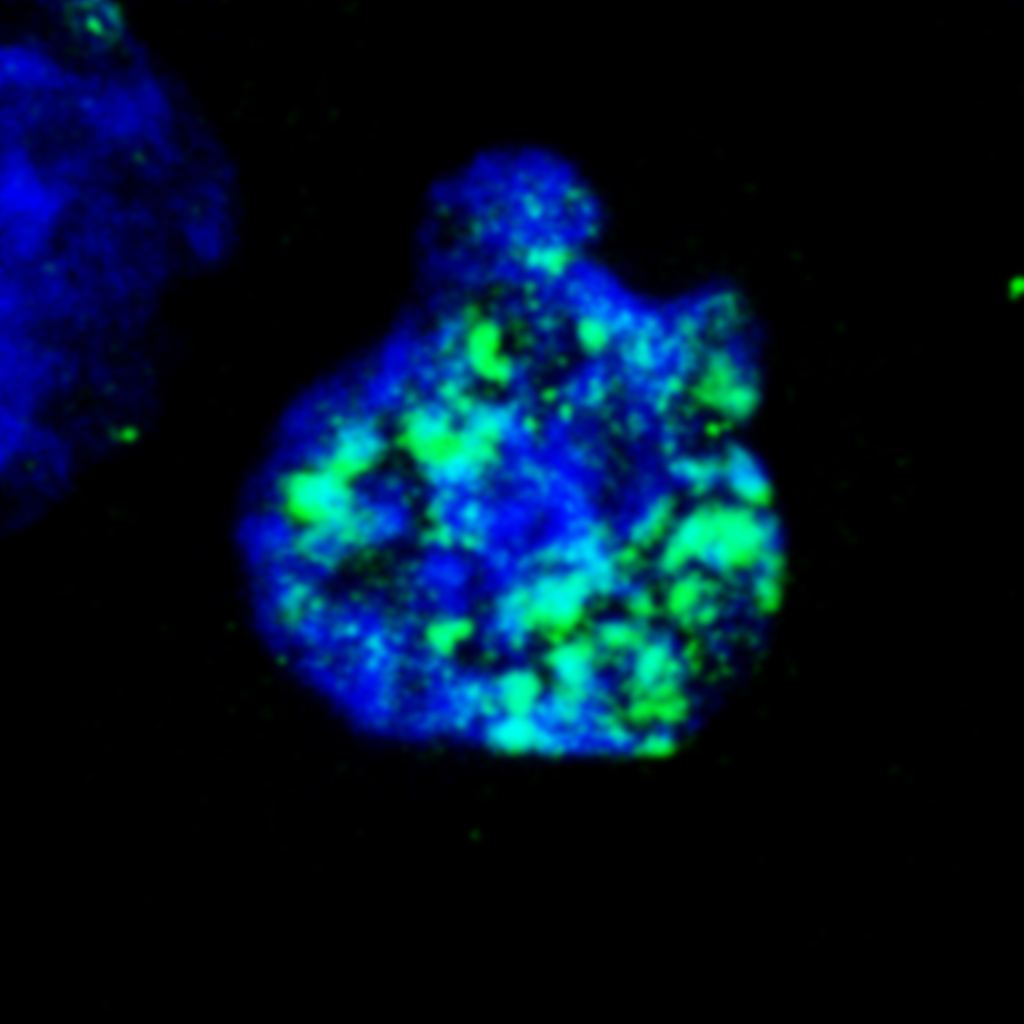

Supplement: Supplementary file 5 — Source data Fig. 3 [file 44318_2024_169_MOESM5_ESM.zip › SD_Figure_3.zip/Figure 3/3J/+CPT/prophase.tif]

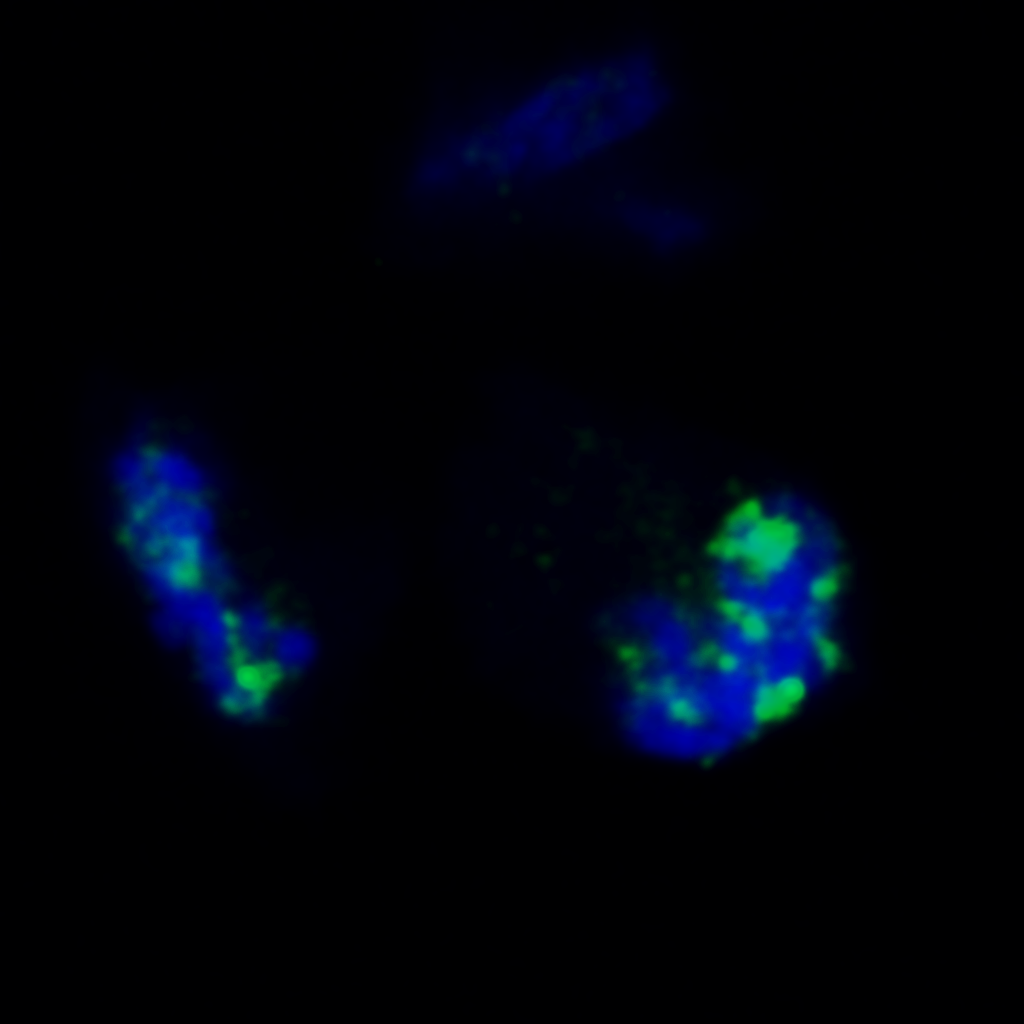

Supplement: Supplementary file 5 — Source data Fig. 3 [file 44318_2024_169_MOESM5_ESM.zip › SD_Figure_3.zip/Figure 3/3J/+CPT/telo.tif]

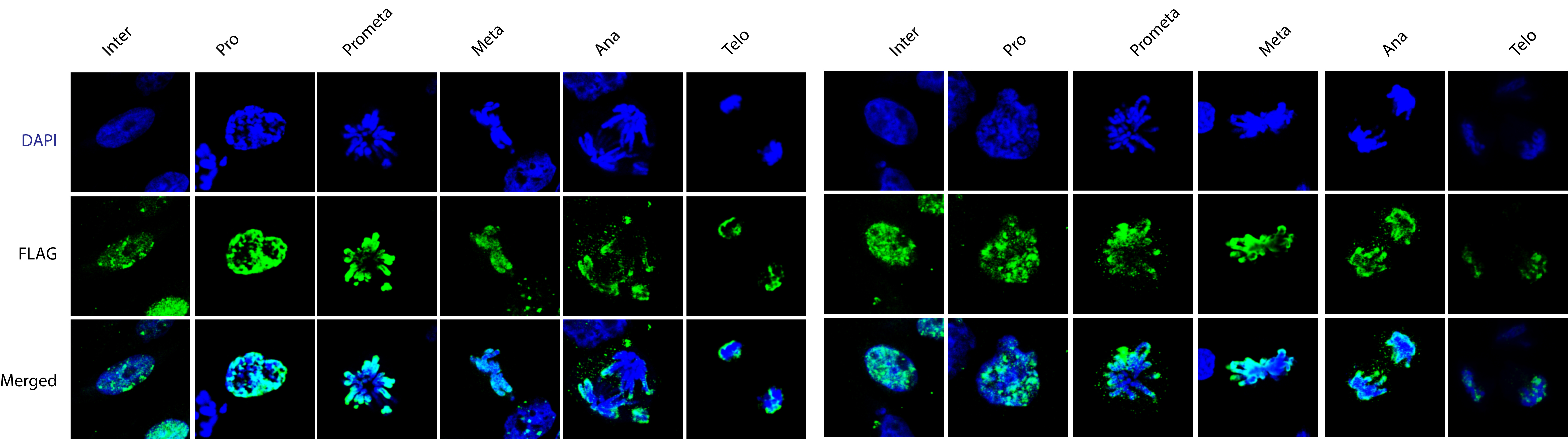

Supplement: Supplementary file 5 — Source data Fig. 3 [file 44318_2024_169_MOESM5_ESM.zip › SD_Figure_3.zip/Figure 3/3J/3J.tif]

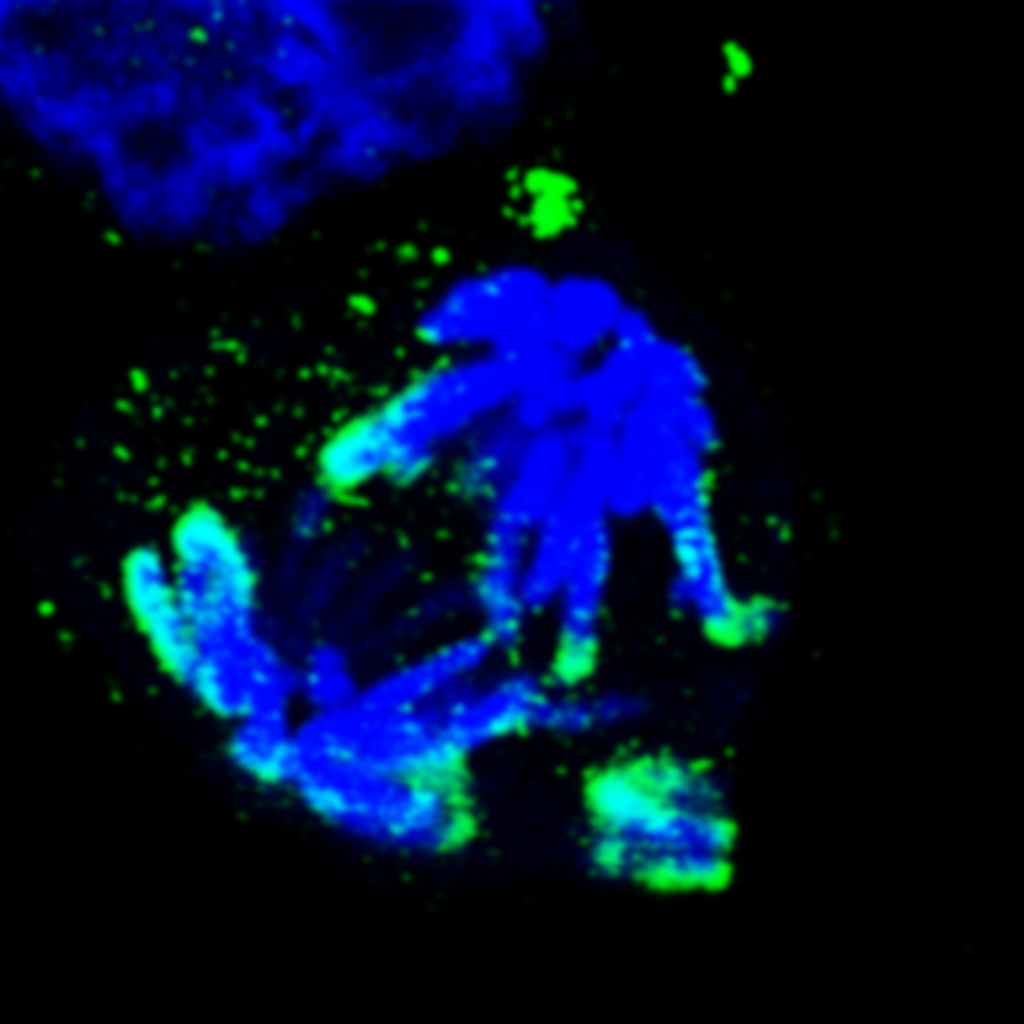

Supplement: Supplementary file 5 — Source data Fig. 3 [file 44318_2024_169_MOESM5_ESM.zip › SD_Figure_3.zip/Figure 3/3J/-CPT/anaphase.tif]

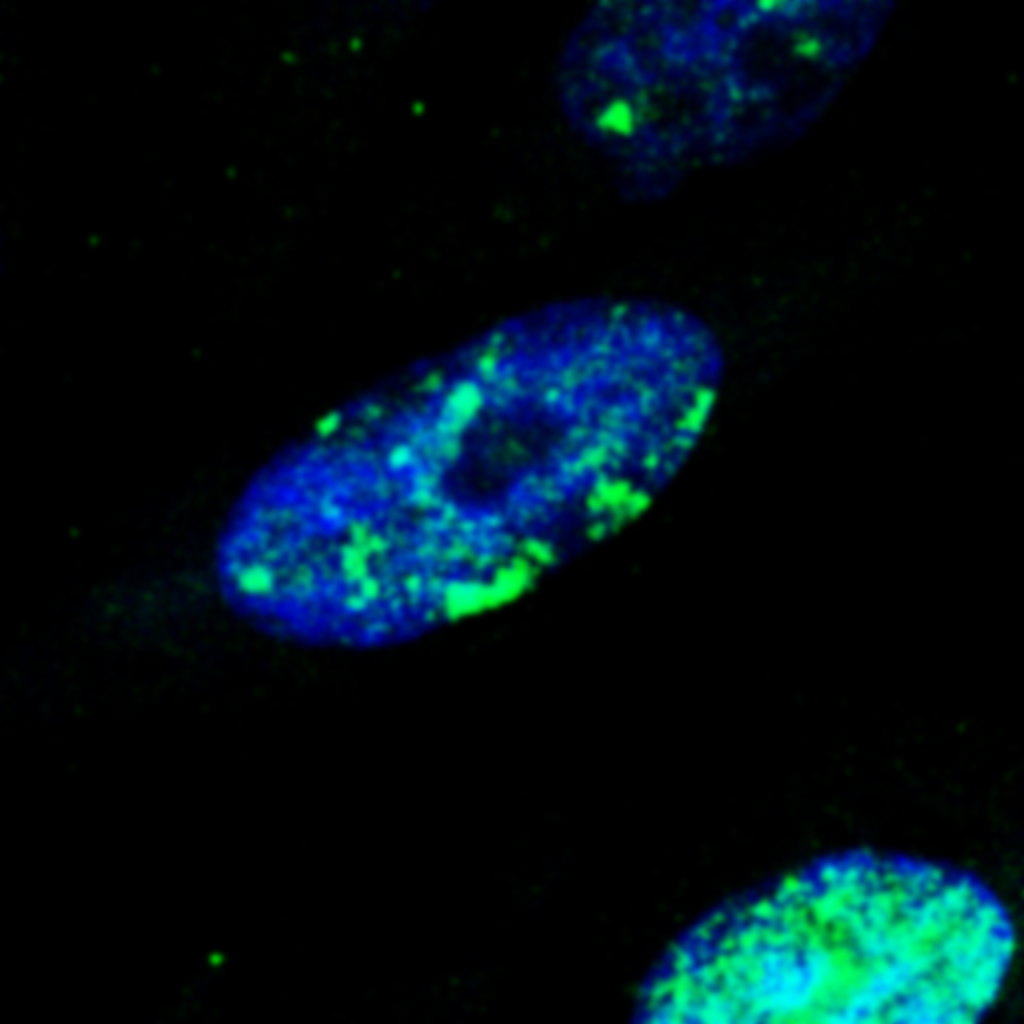

Supplement: Supplementary file 5 — Source data Fig. 3 [file 44318_2024_169_MOESM5_ESM.zip › SD_Figure_3.zip/Figure 3/3J/-CPT/Inter.tif]

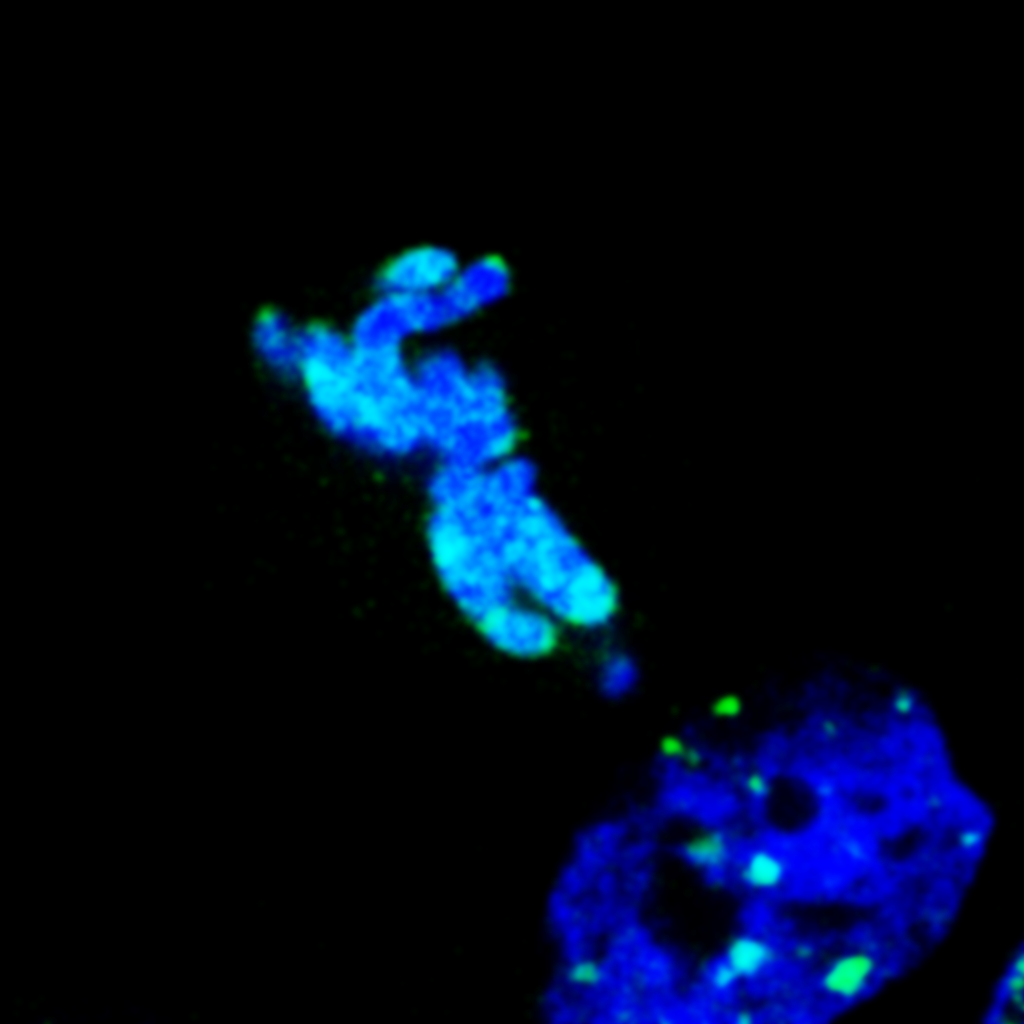

Supplement: Supplementary file 5 — Source data Fig. 3 [file 44318_2024_169_MOESM5_ESM.zip › SD_Figure_3.zip/Figure 3/3J/-CPT/metaphase.tif]

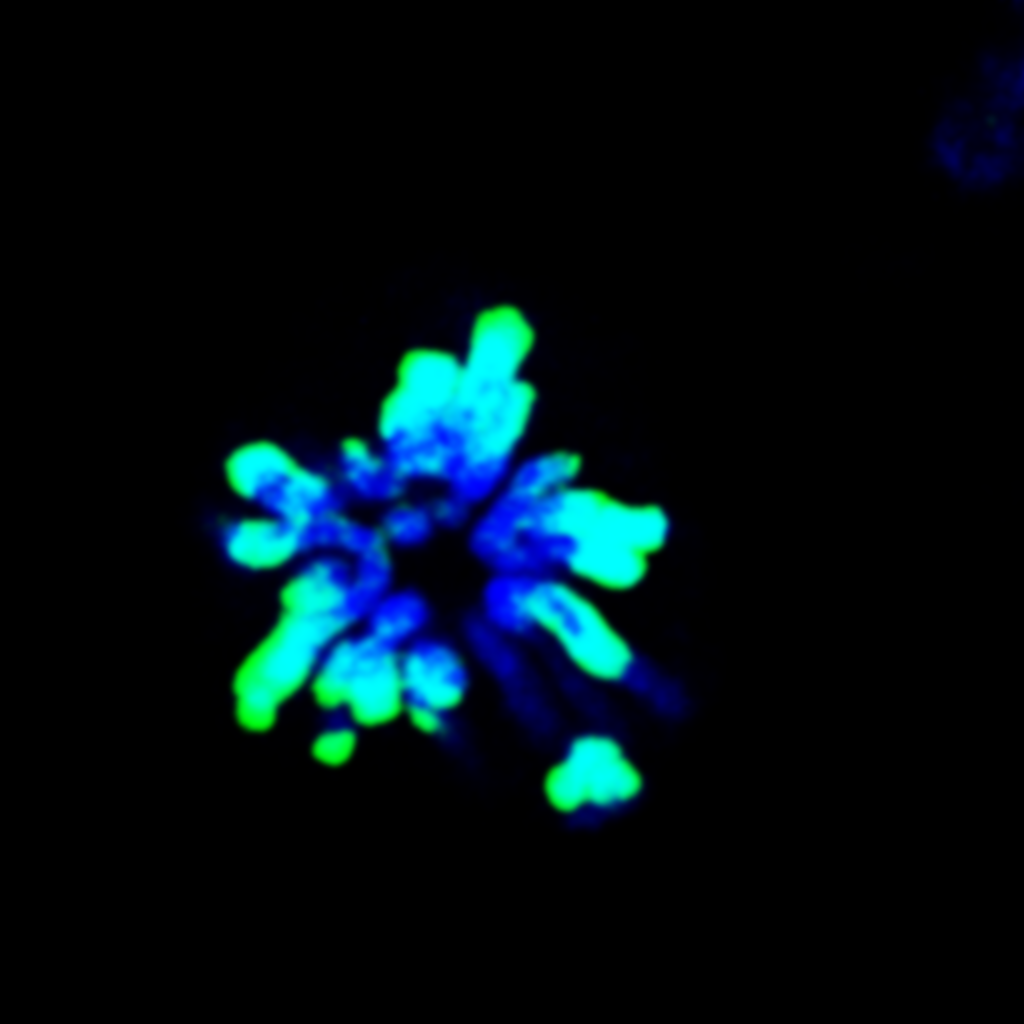

Supplement: Supplementary file 5 — Source data Fig. 3 [file 44318_2024_169_MOESM5_ESM.zip › SD_Figure_3.zip/Figure 3/3J/-CPT/prometaphase.tif]

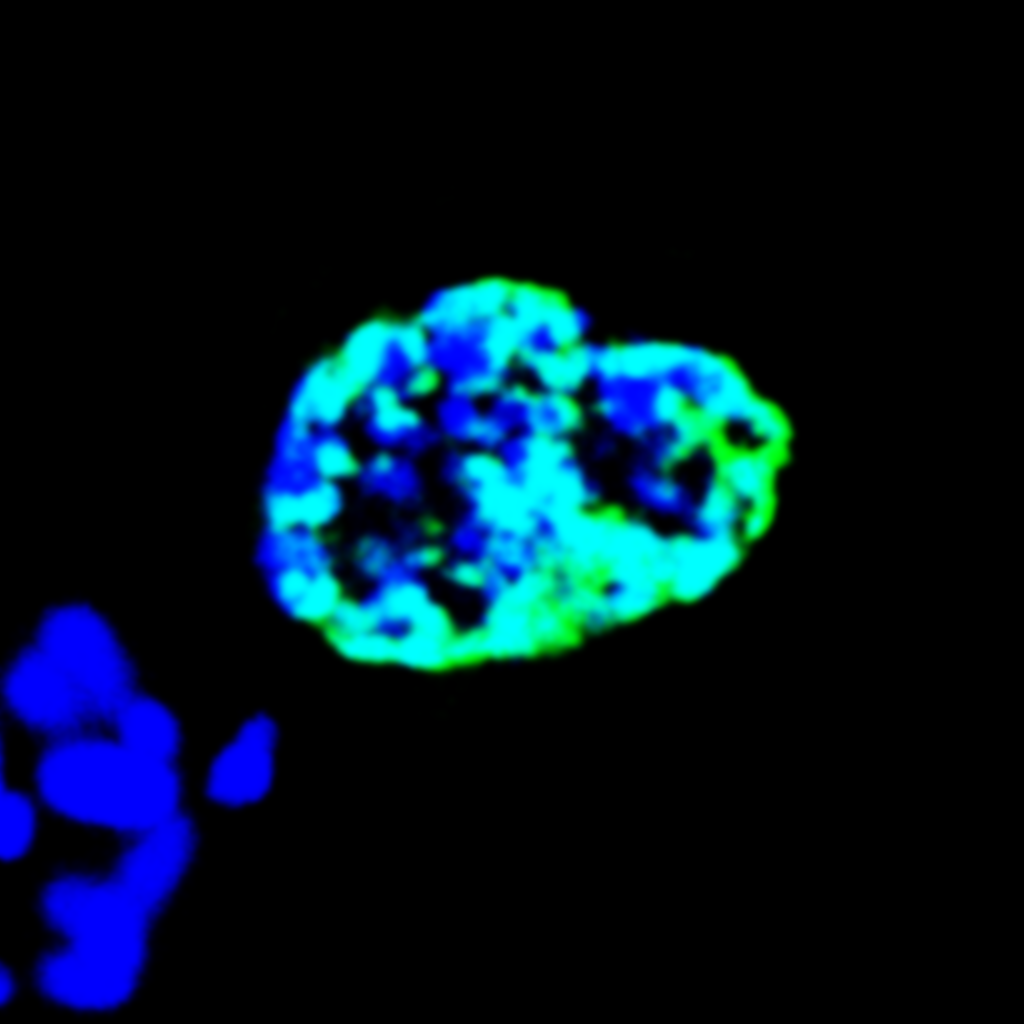

Supplement: Supplementary file 5 — Source data Fig. 3 [file 44318_2024_169_MOESM5_ESM.zip › SD_Figure_3.zip/Figure 3/3J/-CPT/prophase.tif]

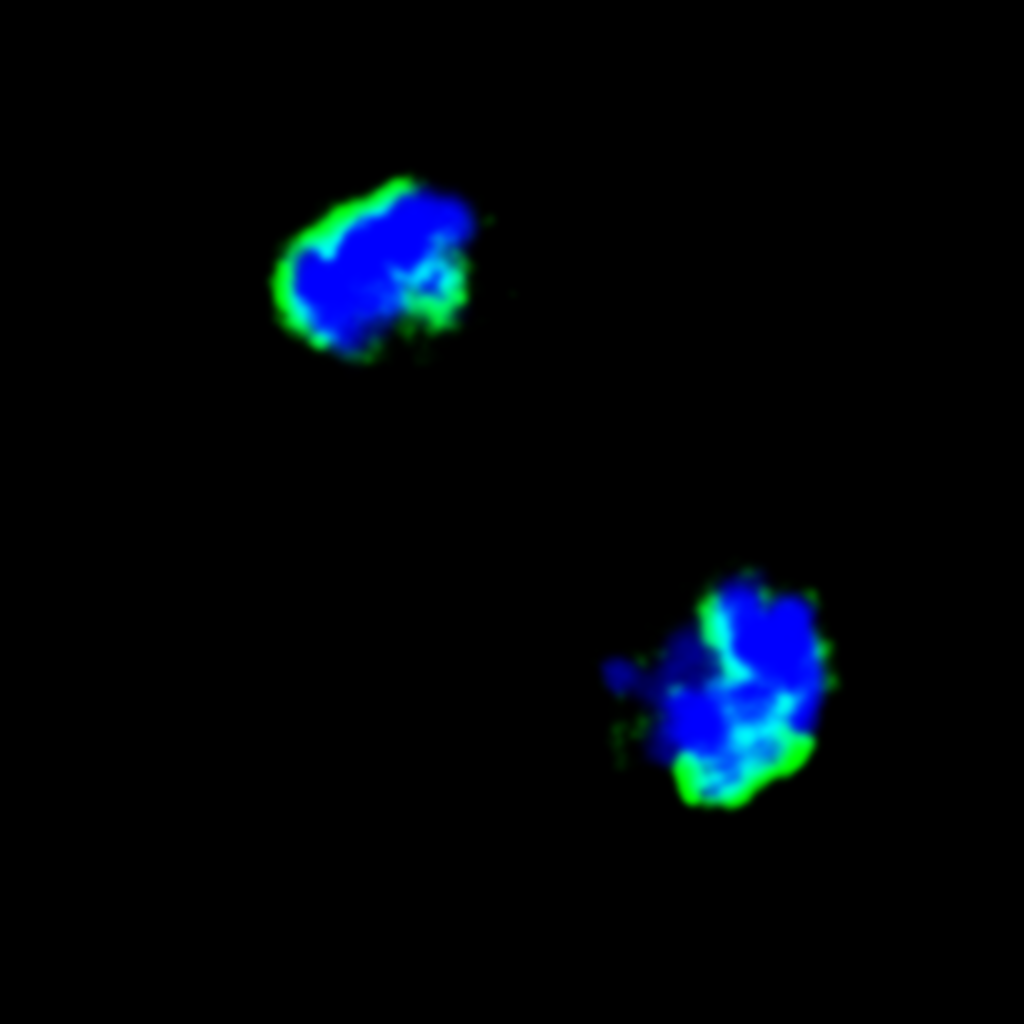

Supplement: Supplementary file 5 — Source data Fig. 3 [file 44318_2024_169_MOESM5_ESM.zip › SD_Figure_3.zip/Figure 3/3J/-CPT/Telophase.tif]

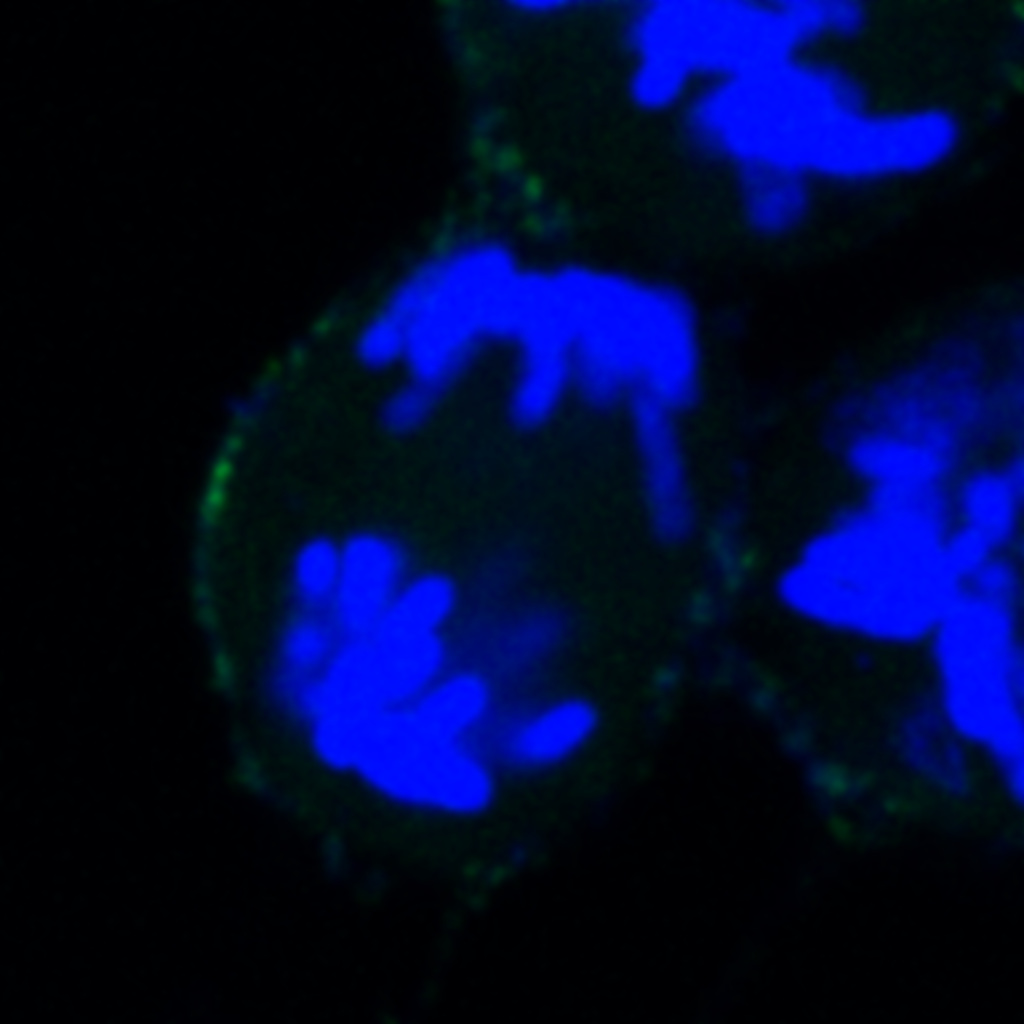

Supplement: Supplementary file 5 — Source data Fig. 3 [file 44318_2024_169_MOESM5_ESM.zip › SD_Figure_3.zip/Figure 3/3K/+CPT/ana.tif]

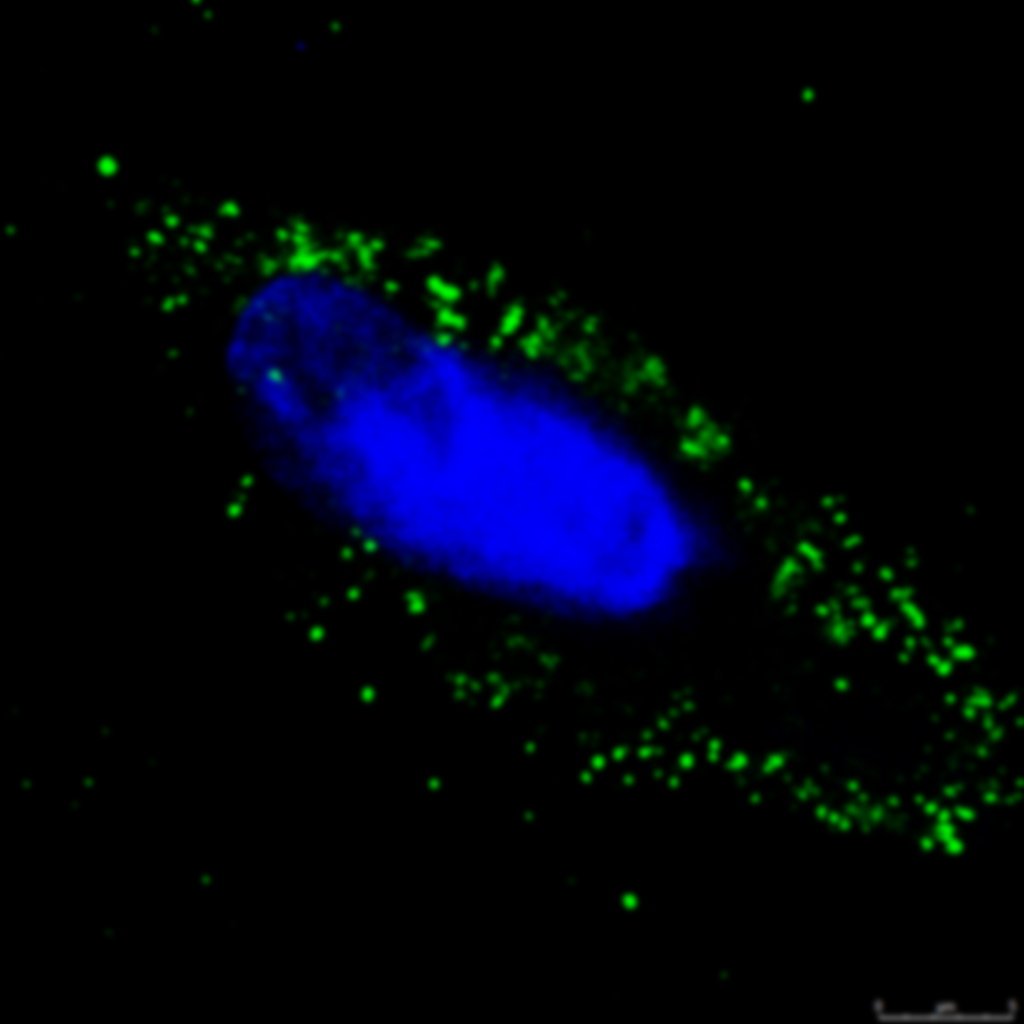

Supplement: Supplementary file 5 — Source data Fig. 3 [file 44318_2024_169_MOESM5_ESM.zip › SD_Figure_3.zip/Figure 3/3K/+CPT/inter.tif]

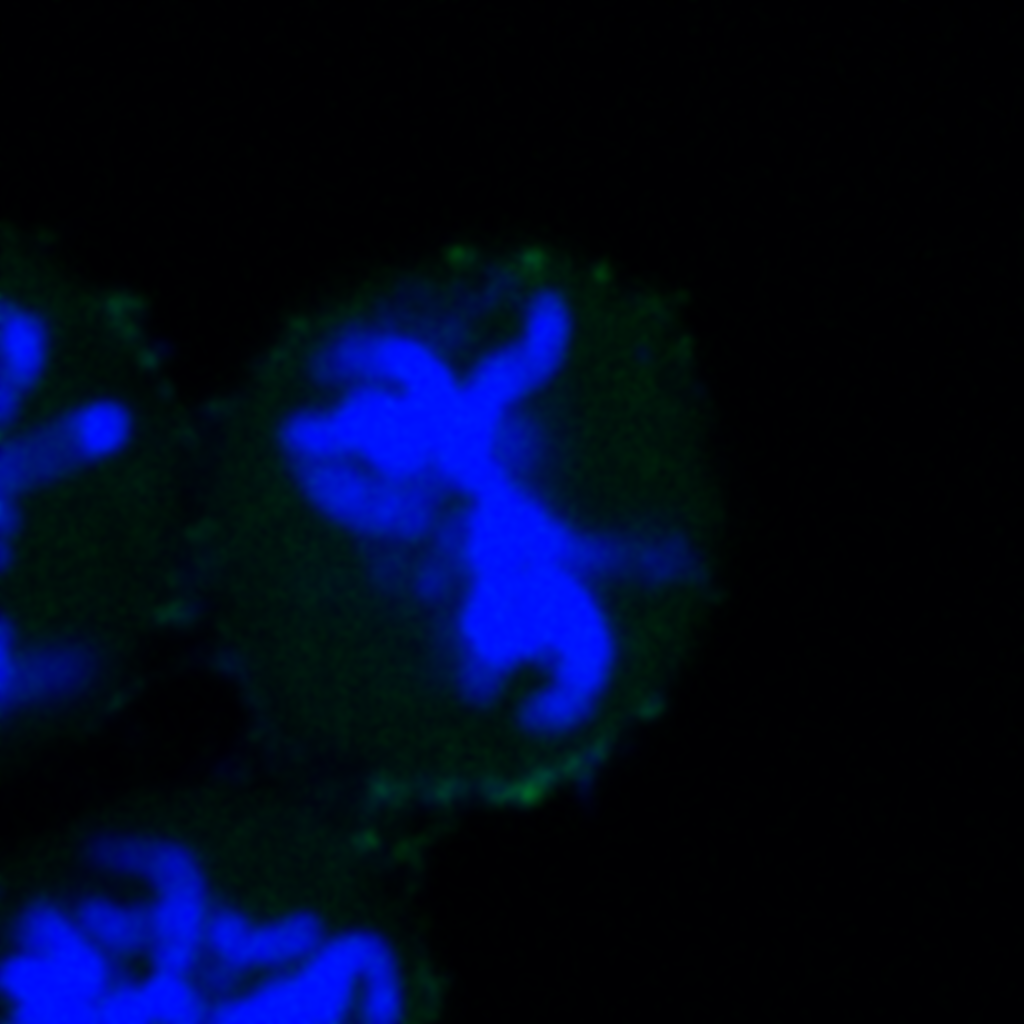

Supplement: Supplementary file 5 — Source data Fig. 3 [file 44318_2024_169_MOESM5_ESM.zip › SD_Figure_3.zip/Figure 3/3K/+CPT/meta.tif]

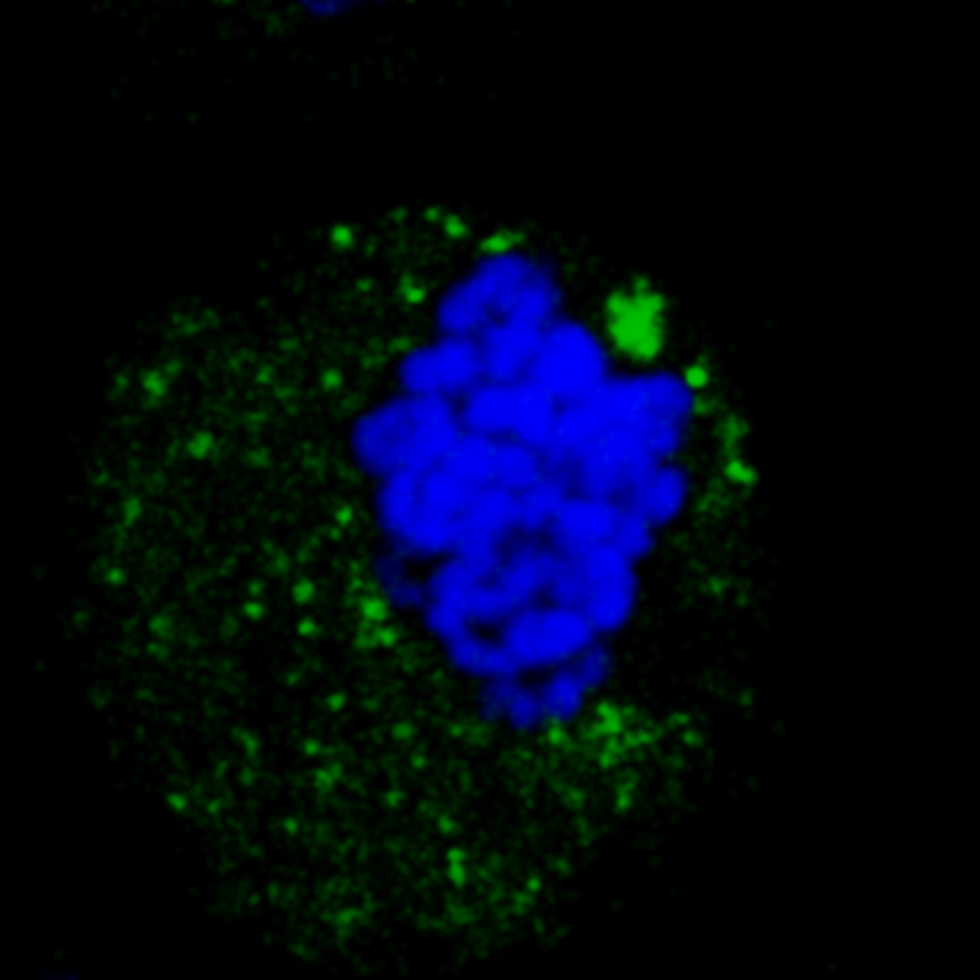

Supplement: Supplementary file 5 — Source data Fig. 3 [file 44318_2024_169_MOESM5_ESM.zip › SD_Figure_3.zip/Figure 3/3K/+CPT/pro.tif]

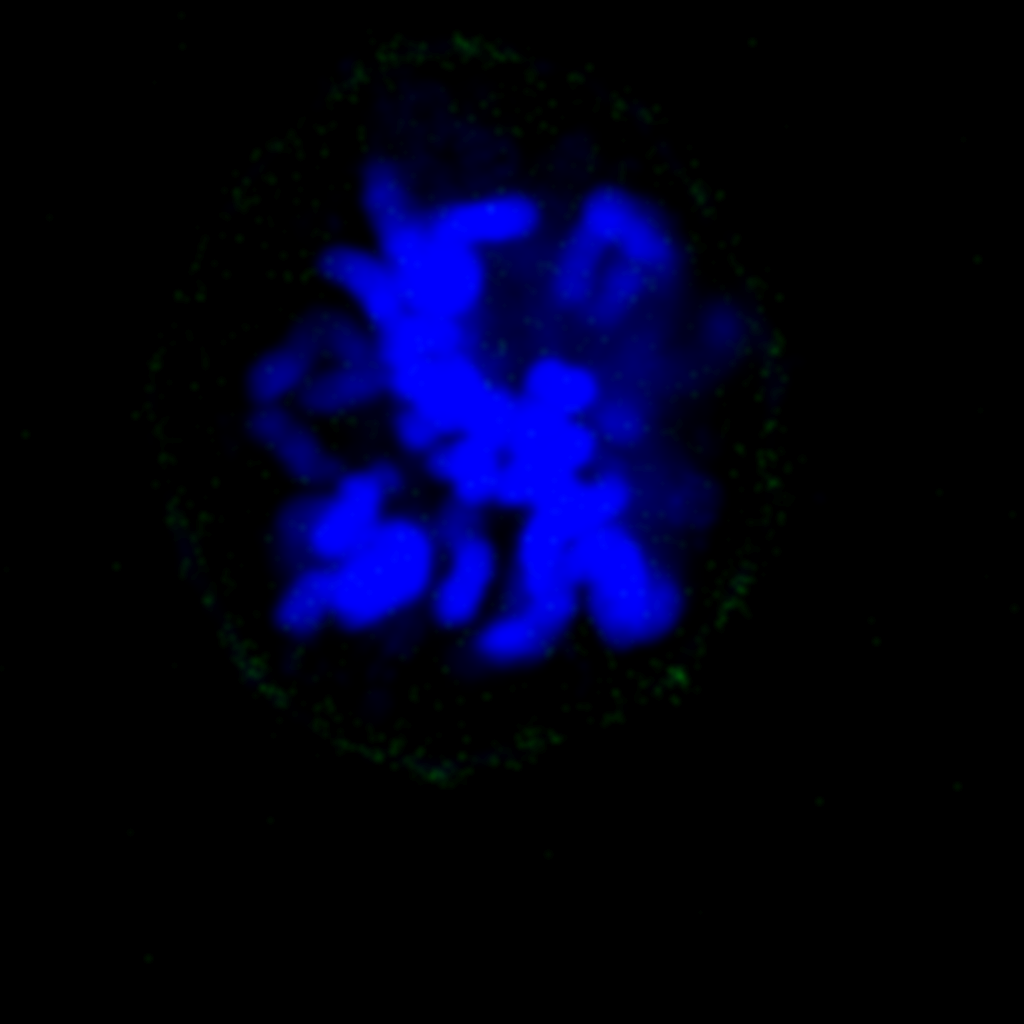

Supplement: Supplementary file 5 — Source data Fig. 3 [file 44318_2024_169_MOESM5_ESM.zip › SD_Figure_3.zip/Figure 3/3K/+CPT/prometa.tif]

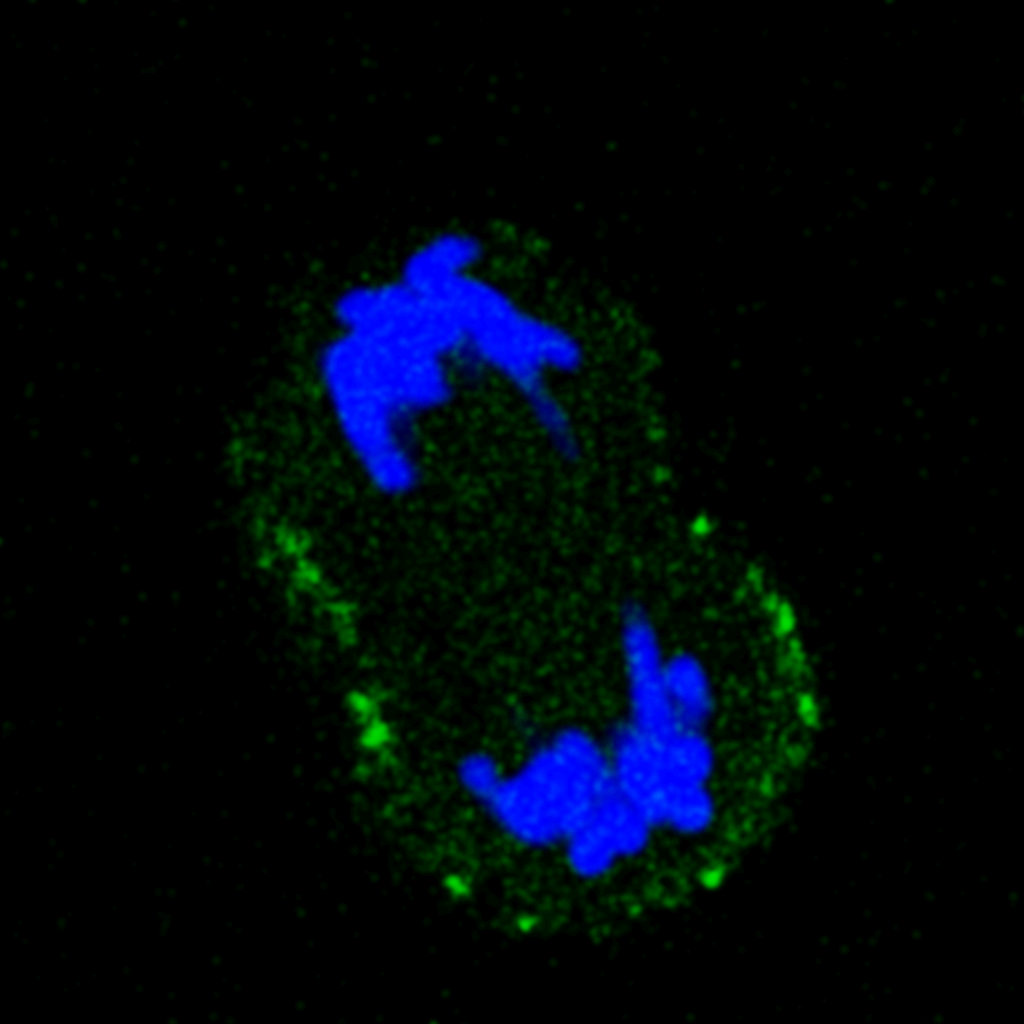

Supplement: Supplementary file 5 — Source data Fig. 3 [file 44318_2024_169_MOESM5_ESM.zip › SD_Figure_3.zip/Figure 3/3K/+CPT/telo.tif]

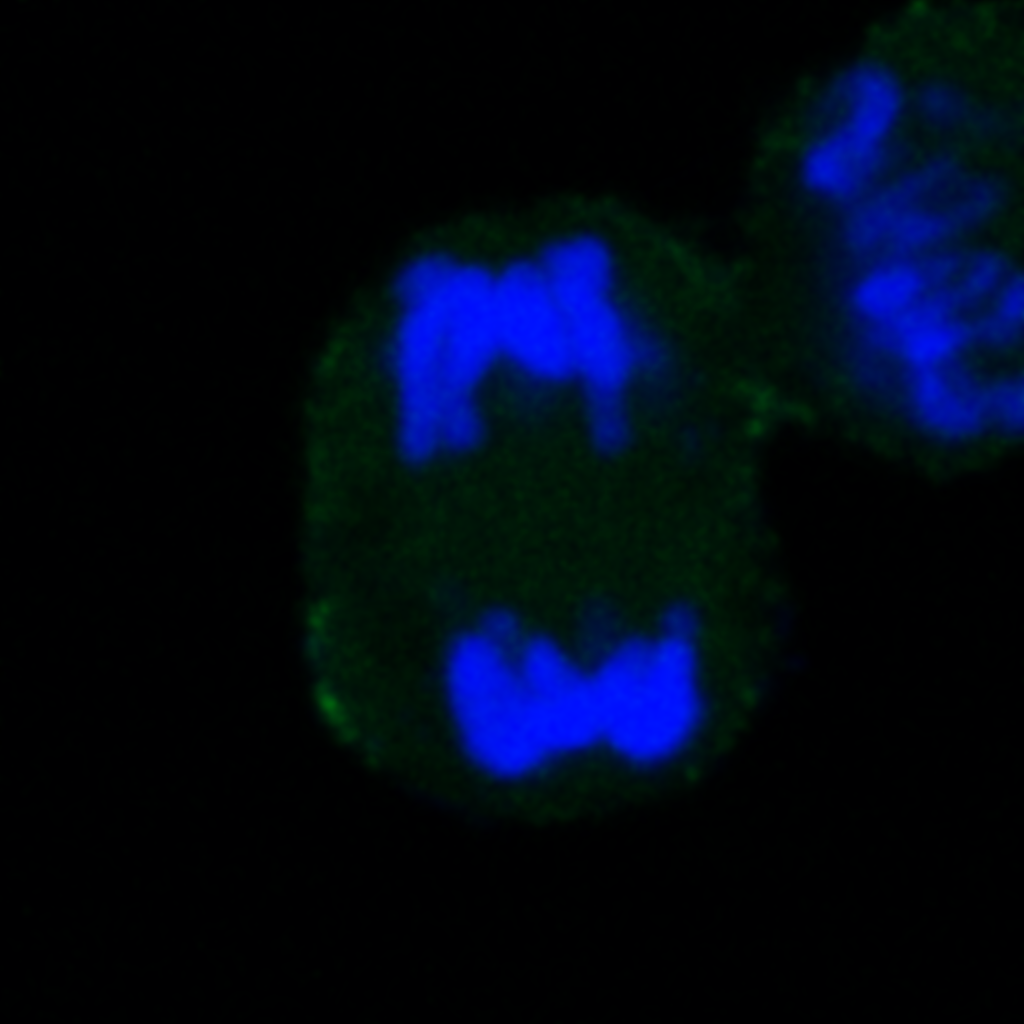

Supplement: Supplementary file 5 — Source data Fig. 3 [file 44318_2024_169_MOESM5_ESM.zip › SD_Figure_3.zip/Figure 3/3K/-CPT/ana.tif]

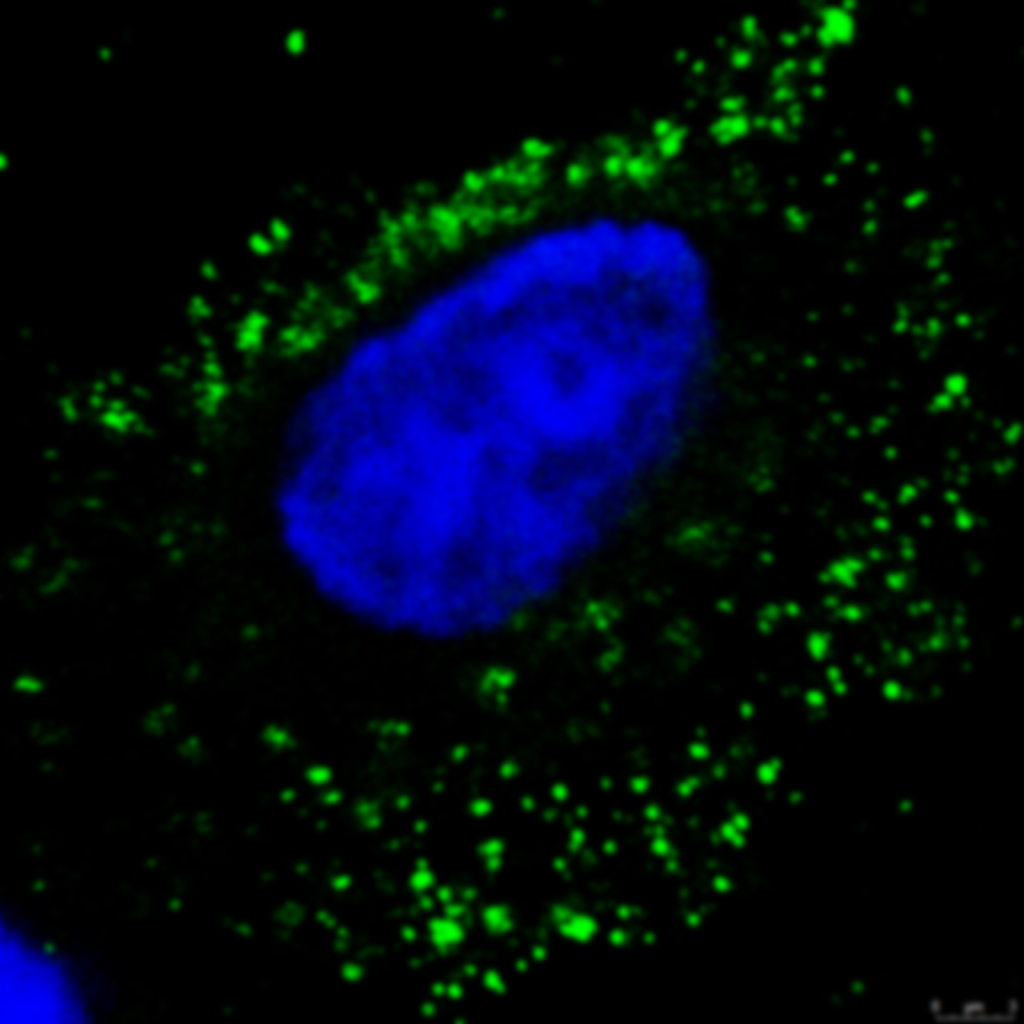

Supplement: Supplementary file 5 — Source data Fig. 3 [file 44318_2024_169_MOESM5_ESM.zip › SD_Figure_3.zip/Figure 3/3K/-CPT/inter.tif]

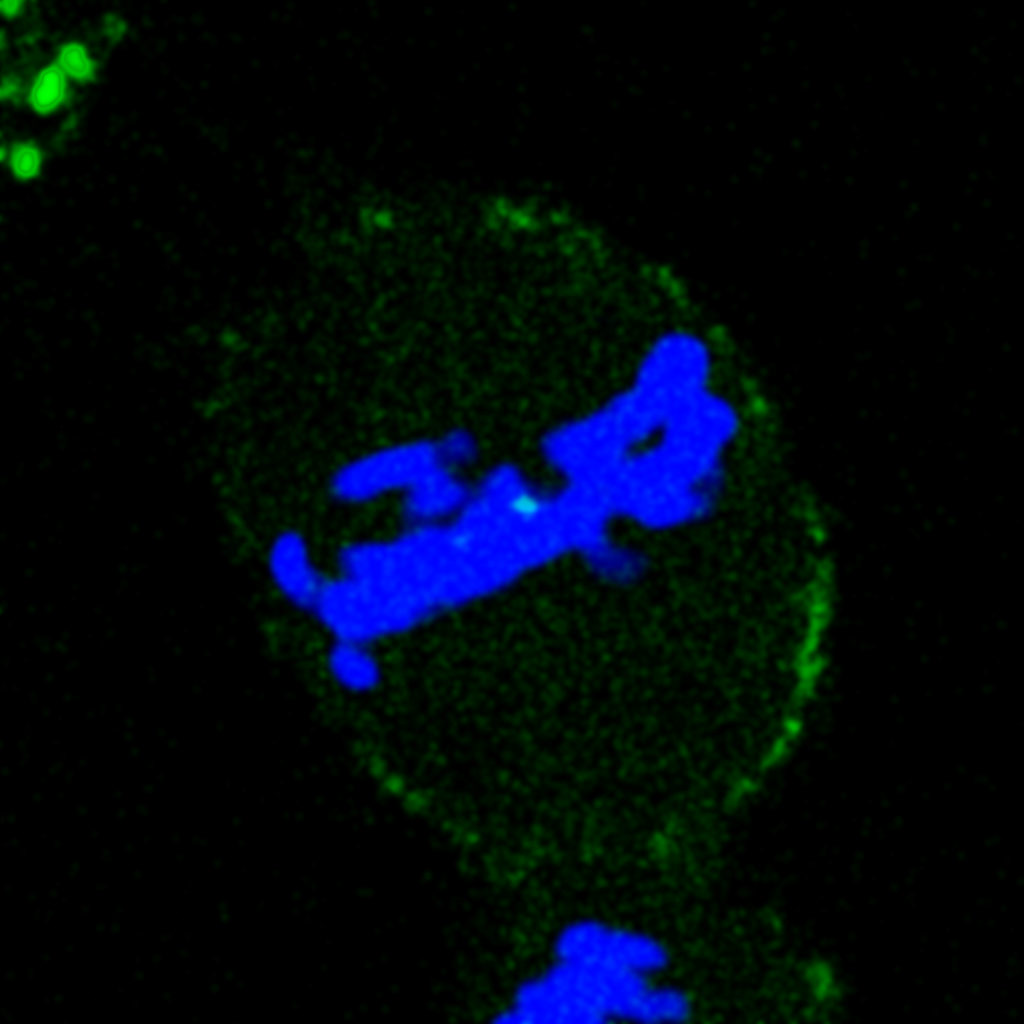

Supplement: Supplementary file 5 — Source data Fig. 3 [file 44318_2024_169_MOESM5_ESM.zip › SD_Figure_3.zip/Figure 3/3K/-CPT/meta.tif]

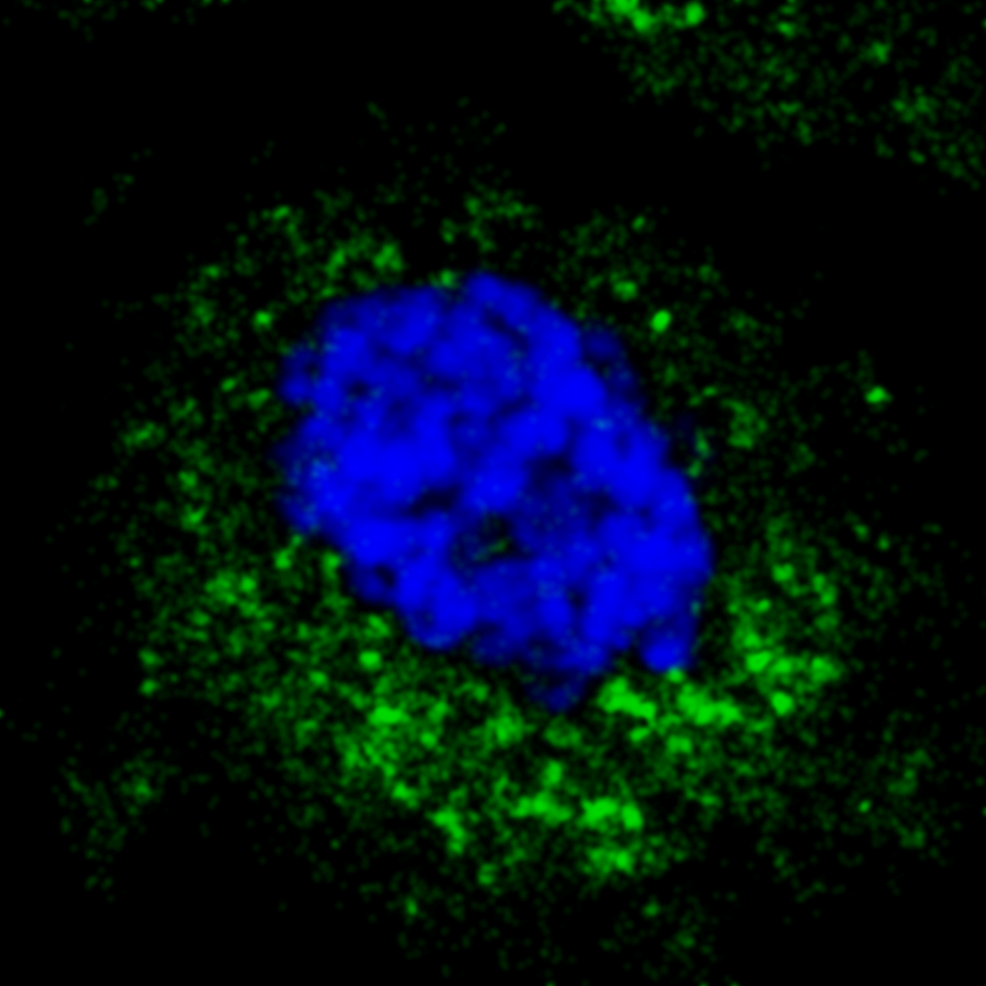

Supplement: Supplementary file 5 — Source data Fig. 3 [file 44318_2024_169_MOESM5_ESM.zip › SD_Figure_3.zip/Figure 3/3K/-CPT/pro.tif]

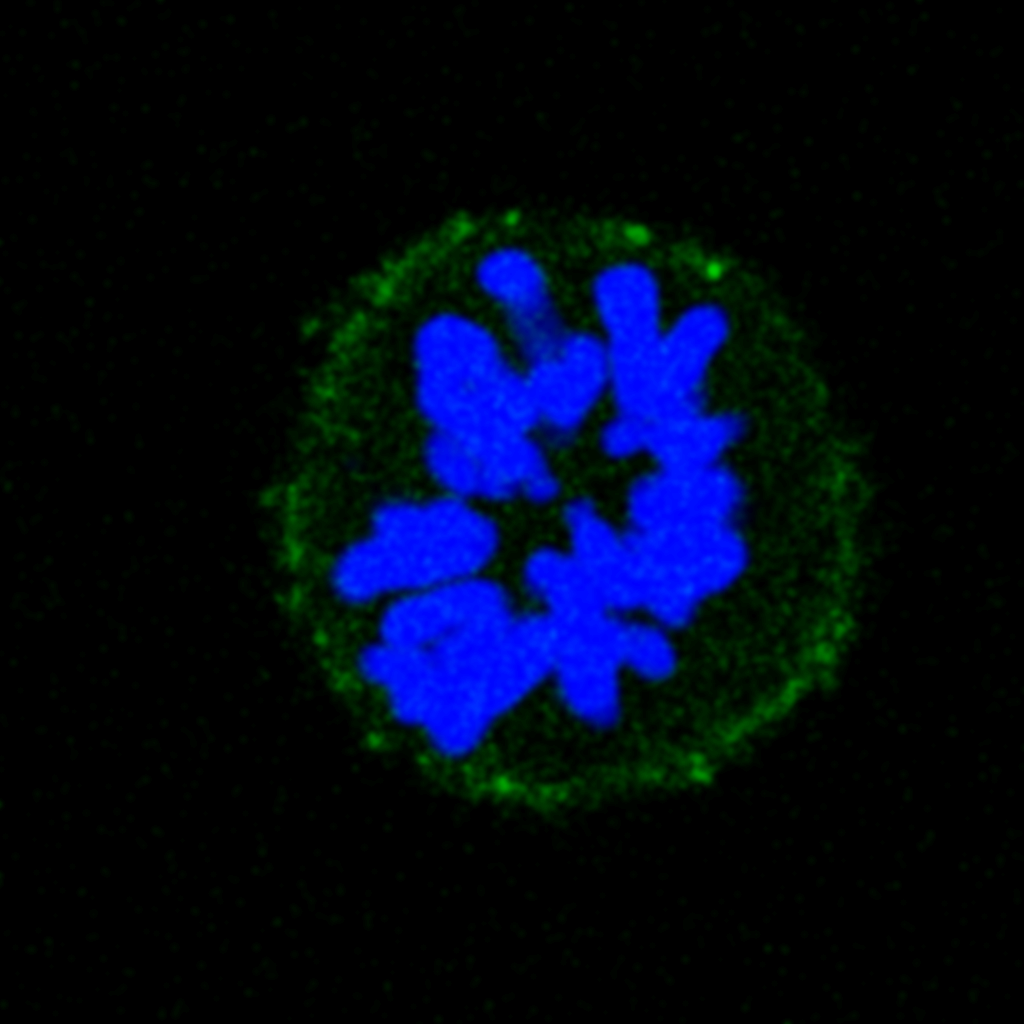

Supplement: Supplementary file 5 — Source data Fig. 3 [file 44318_2024_169_MOESM5_ESM.zip › SD_Figure_3.zip/Figure 3/3K/-CPT/prometa.tif]

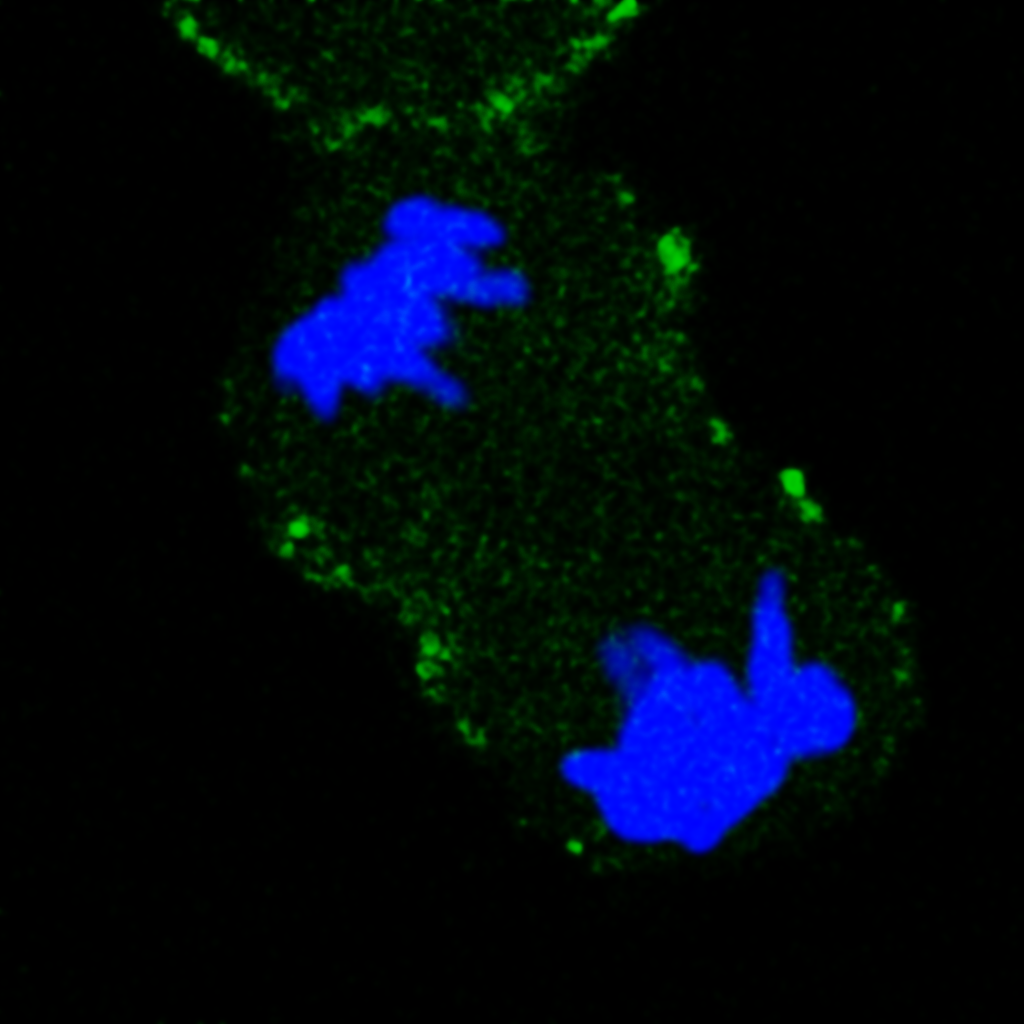

Supplement: Supplementary file 5 — Source data Fig. 3 [file 44318_2024_169_MOESM5_ESM.zip › SD_Figure_3.zip/Figure 3/3K/-CPT/telo.tif]

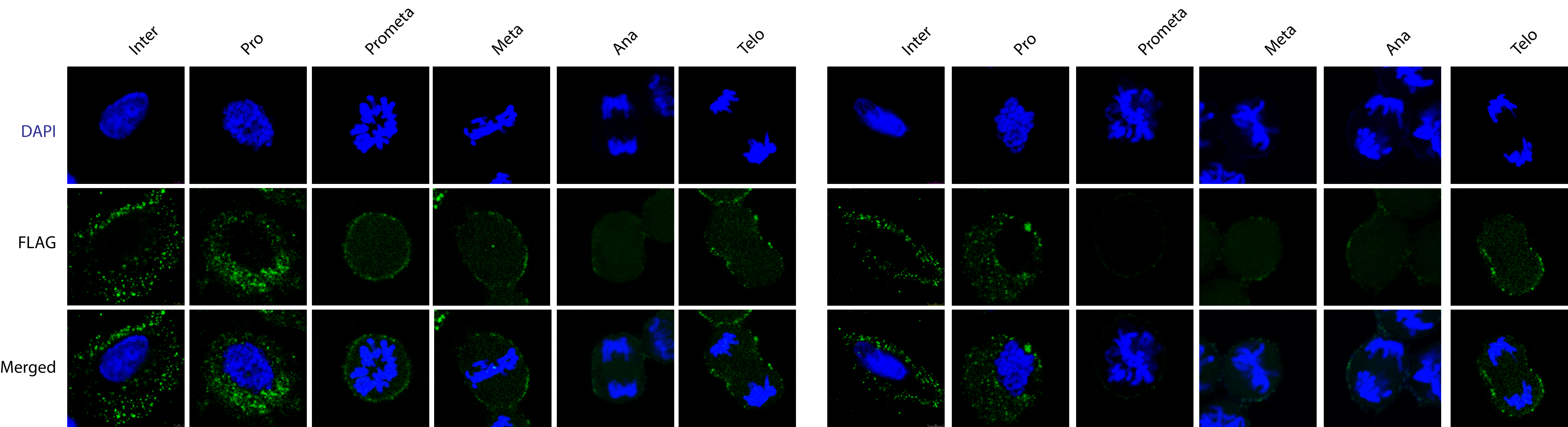

Supplement: Supplementary file 5 — Source data Fig. 3 [file 44318_2024_169_MOESM5_ESM.zip › SD_Figure_3.zip/Figure 3/3K/Fig 3K.tif]

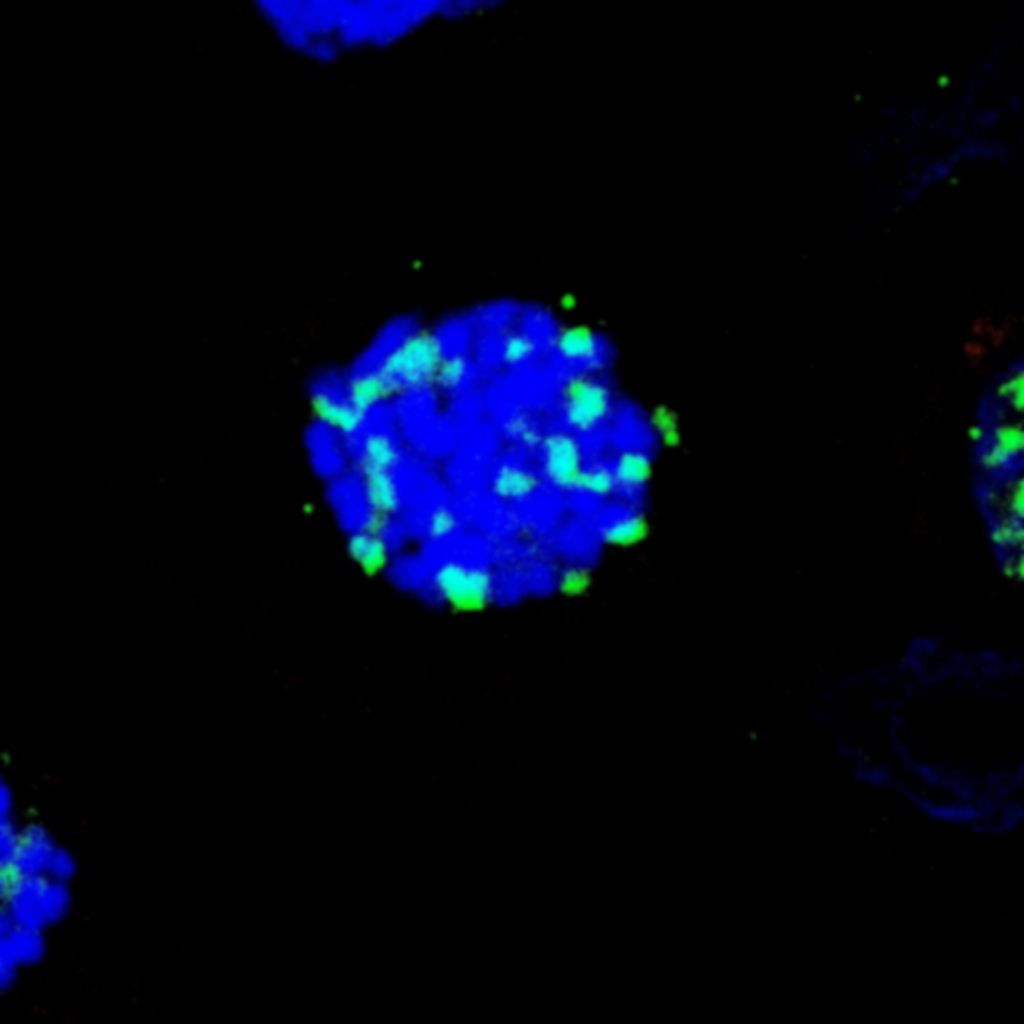

Supplement: Supplementary file 7 — Source data Fig. 5 [file 44318_2024_169_MOESM7_ESM.zip › SD_Figure_5.zip/Figure 5/5A/EV_CPT.tif]

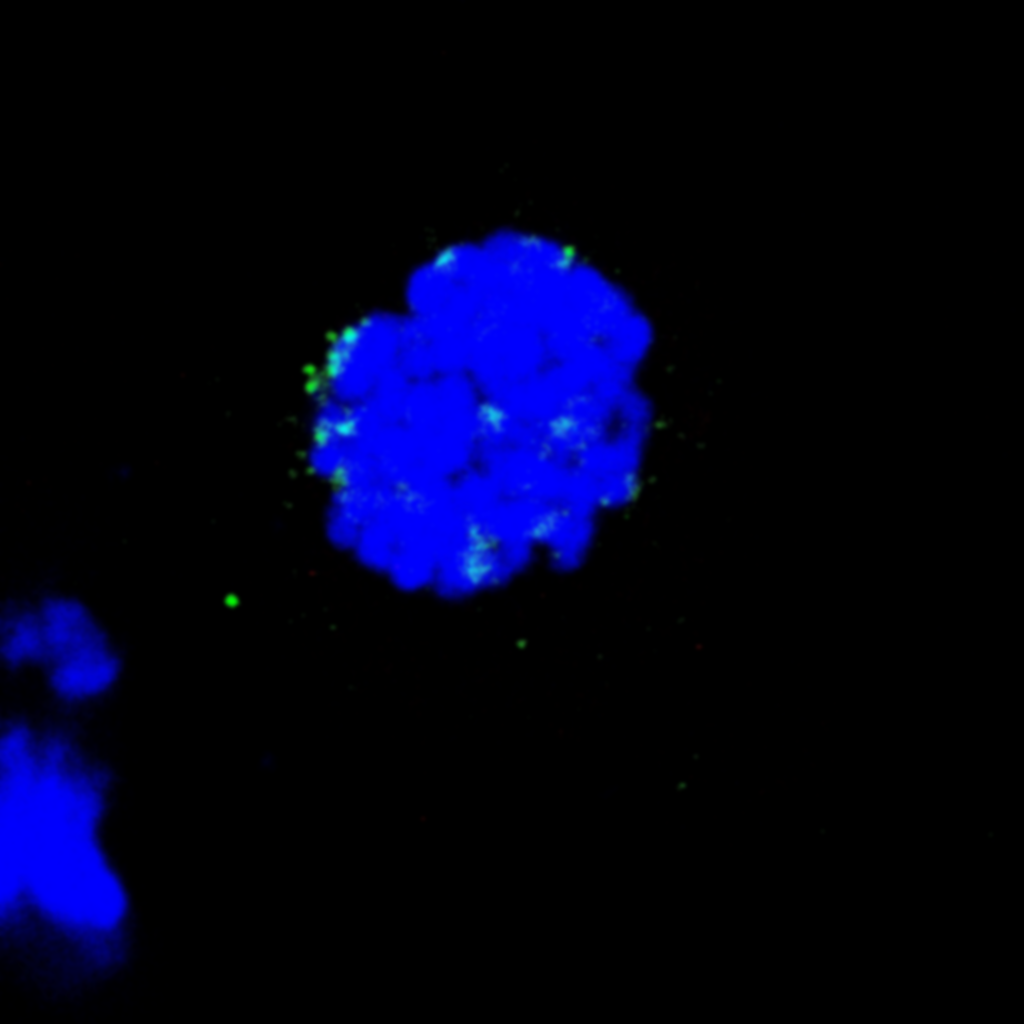

Supplement: Supplementary file 7 — Source data Fig. 5 [file 44318_2024_169_MOESM7_ESM.zip › SD_Figure_5.zip/Figure 5/5A/EV_un.tif]

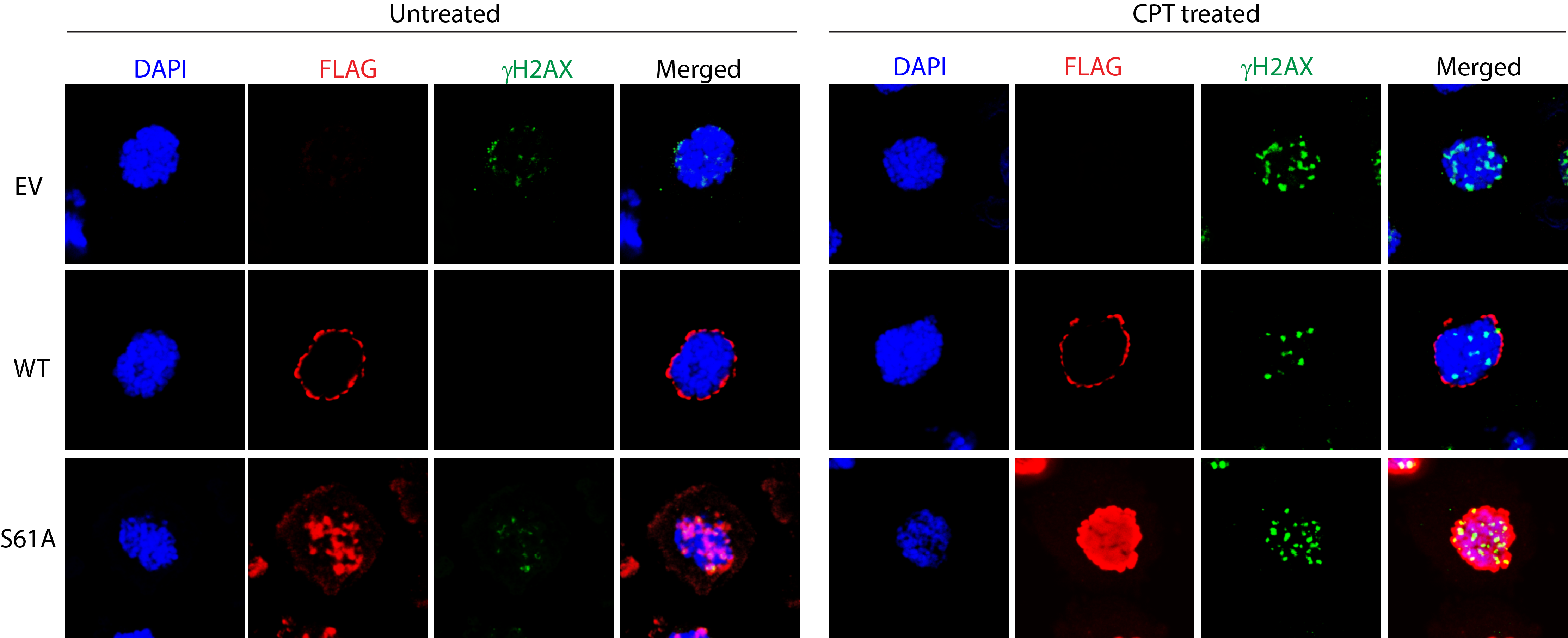

Supplement: Supplementary file 7 — Source data Fig. 5 [file 44318_2024_169_MOESM7_ESM.zip › SD_Figure_5.zip/Figure 5/5A/Fig 5A.tif]

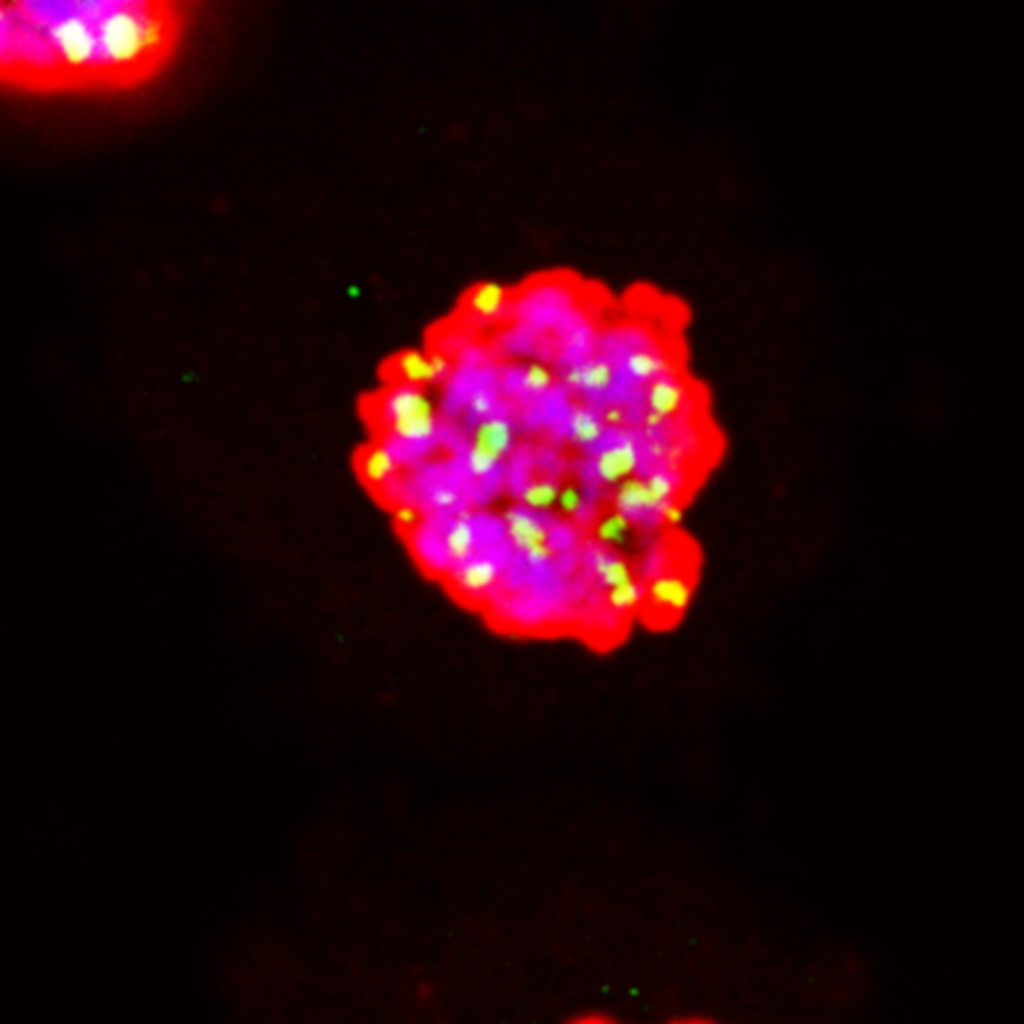

Supplement: Supplementary file 7 — Source data Fig. 5 [file 44318_2024_169_MOESM7_ESM.zip › SD_Figure_5.zip/Figure 5/5A/S61A_CPT.tif]

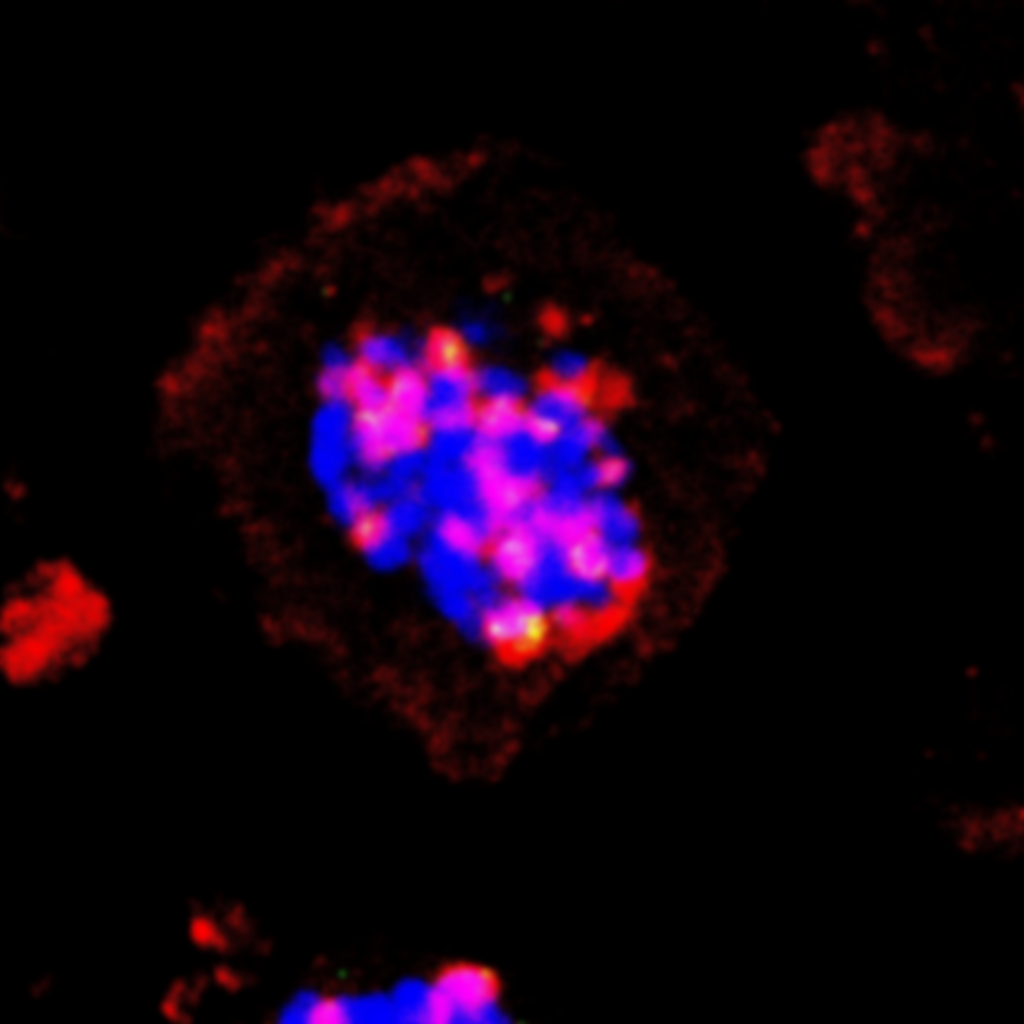

Supplement: Supplementary file 7 — Source data Fig. 5 [file 44318_2024_169_MOESM7_ESM.zip › SD_Figure_5.zip/Figure 5/5A/S61A_UT.tif]

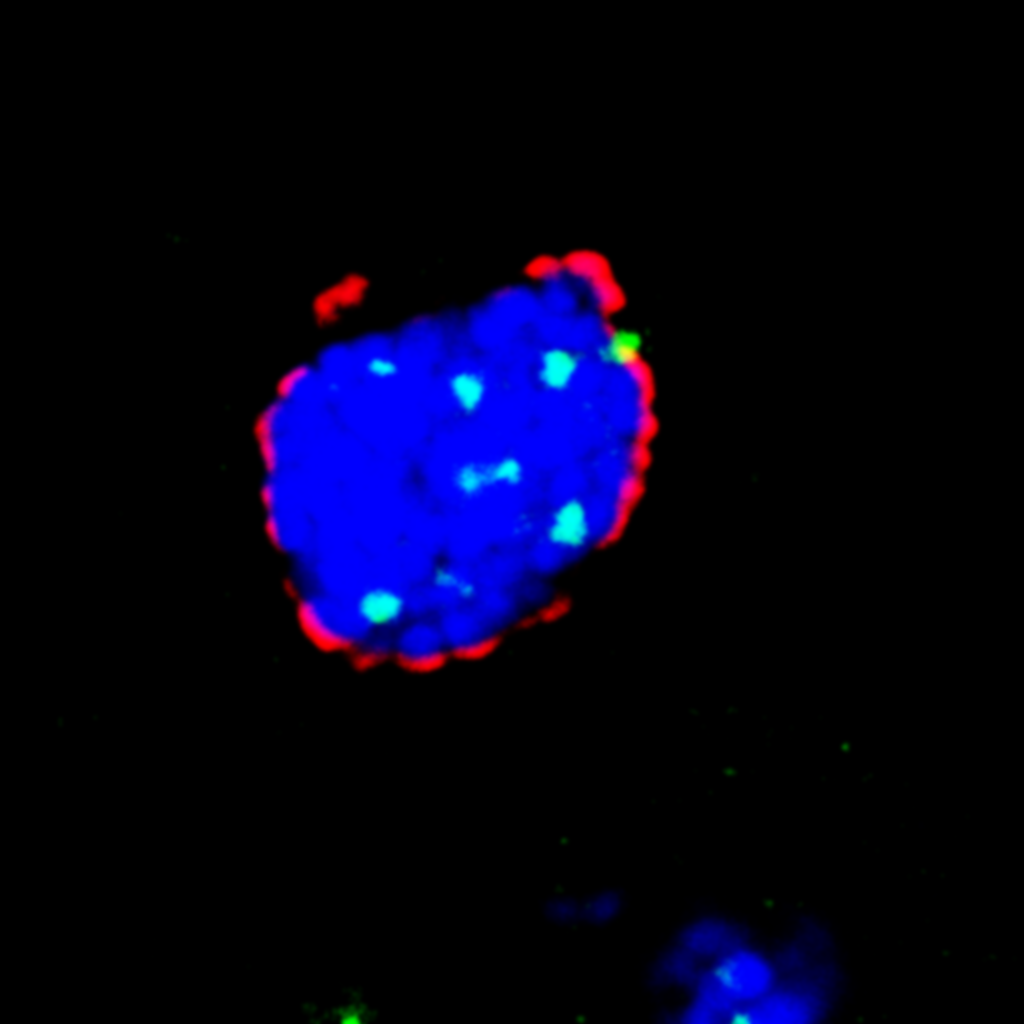

Supplement: Supplementary file 7 — Source data Fig. 5 [file 44318_2024_169_MOESM7_ESM.zip › SD_Figure_5.zip/Figure 5/5A/WT_CPT.tif]

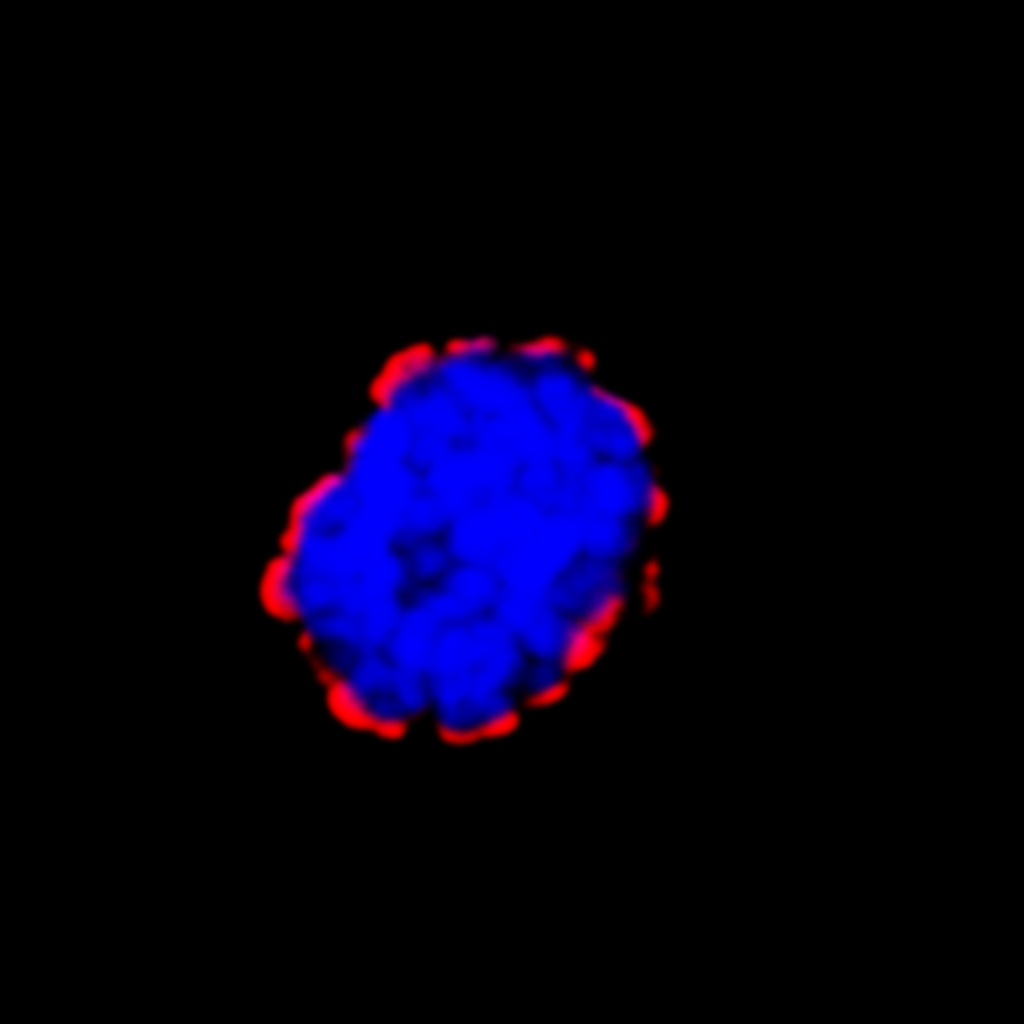

Supplement: Supplementary file 7 — Source data Fig. 5 [file 44318_2024_169_MOESM7_ESM.zip › SD_Figure_5.zip/Figure 5/5A/WT_un.tif]

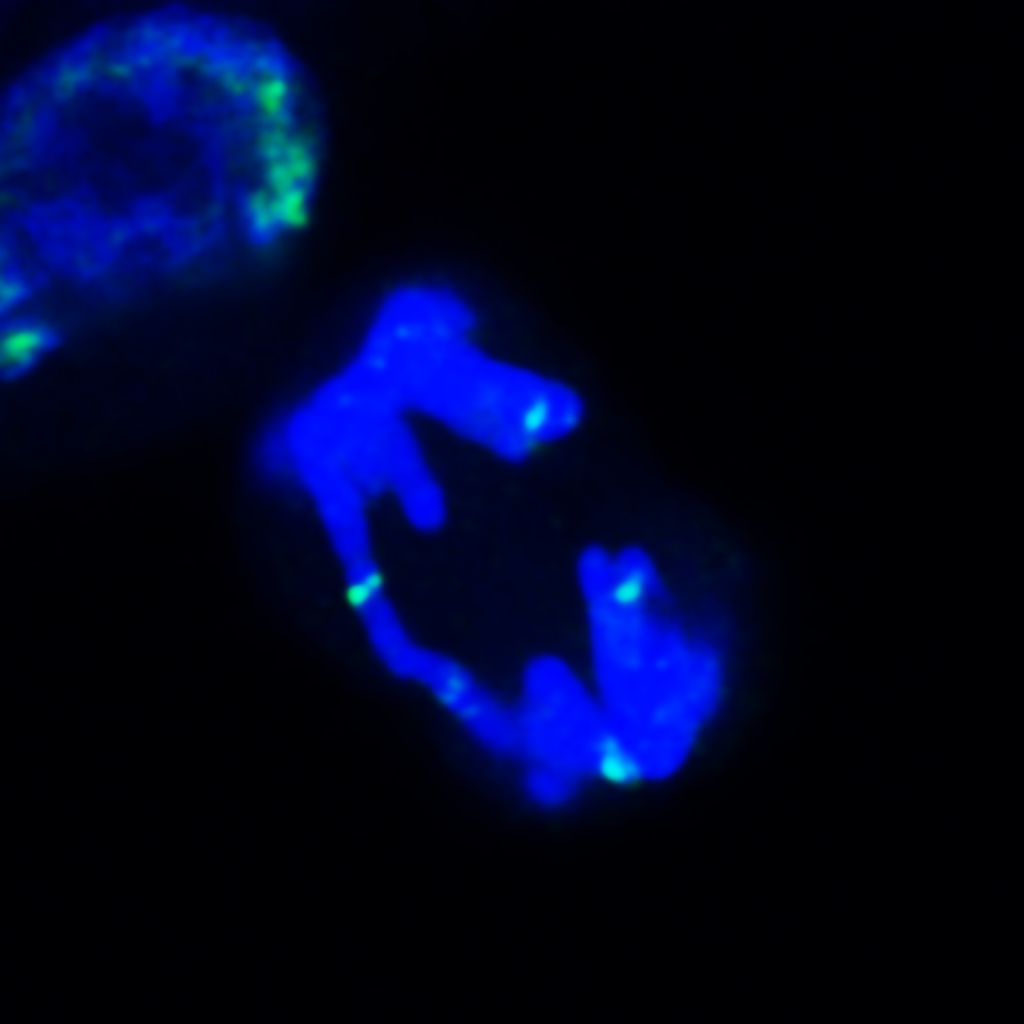

Supplement: Supplementary file 7 — Source data Fig. 5 [file 44318_2024_169_MOESM7_ESM.zip › SD_Figure_5.zip/Figure 5/5C/AB.tif]

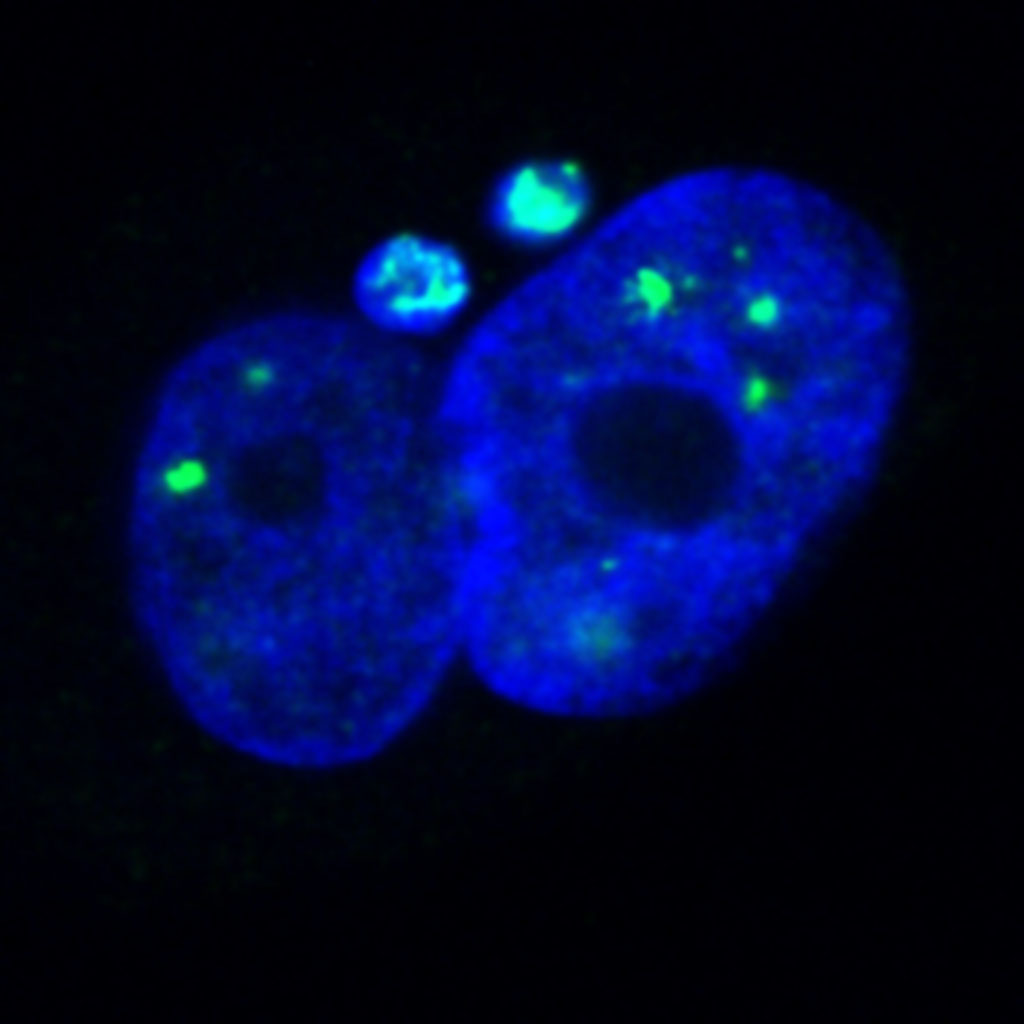

Supplement: Supplementary file 7 — Source data Fig. 5 [file 44318_2024_169_MOESM7_ESM.zip › SD_Figure_5.zip/Figure 5/5E/5E.jpg]

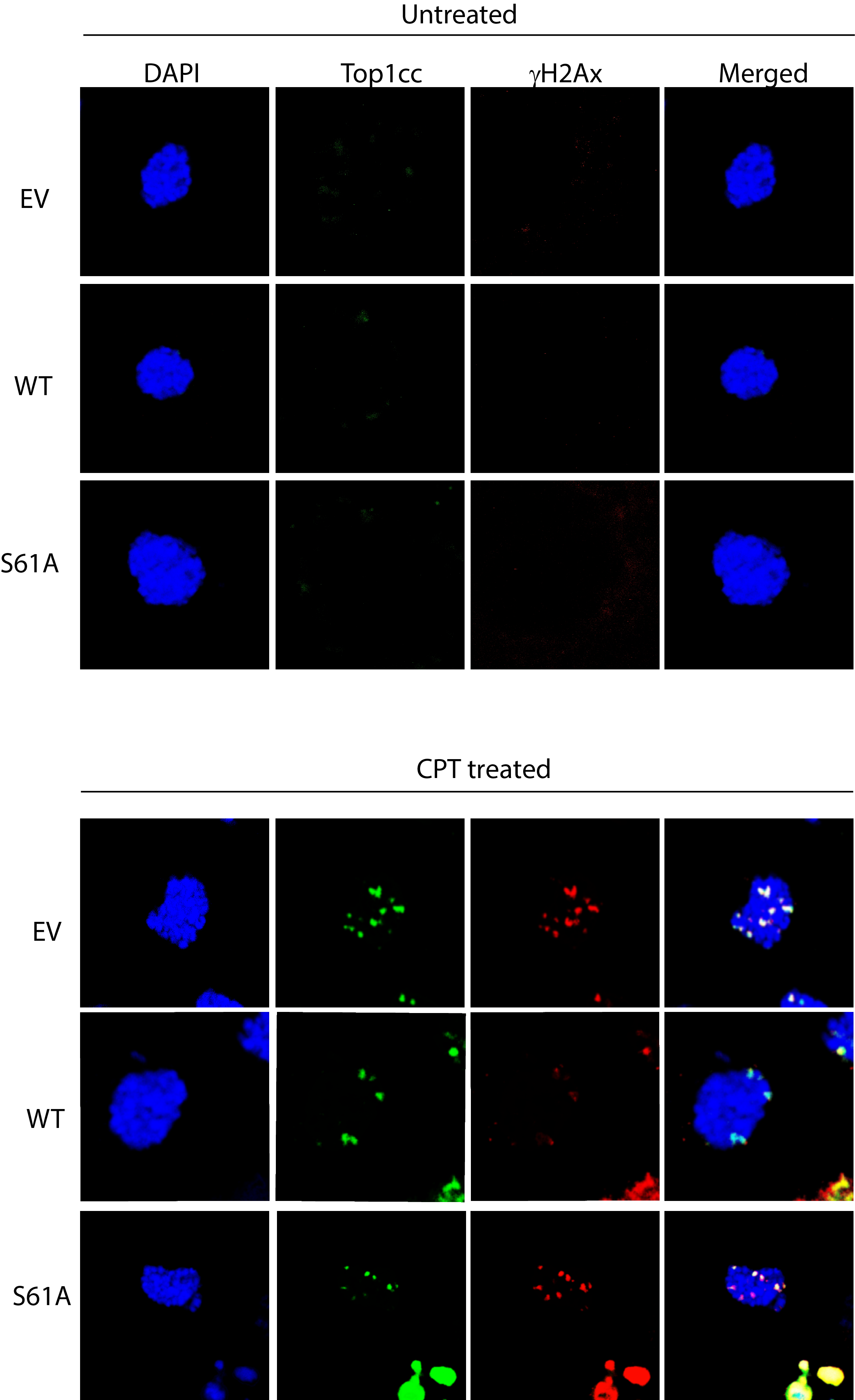

Supplement: Supplementary file 7 — Source data Fig. 5 [file 44318_2024_169_MOESM7_ESM.zip › SD_Figure_5.zip/Figure 5/5G/5G.tif]

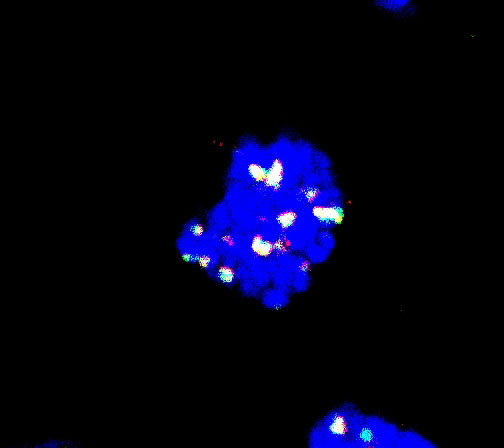

Supplement: Supplementary file 7 — Source data Fig. 5 [file 44318_2024_169_MOESM7_ESM.zip › SD_Figure_5.zip/Figure 5/5G/EV_CPT.jpg]

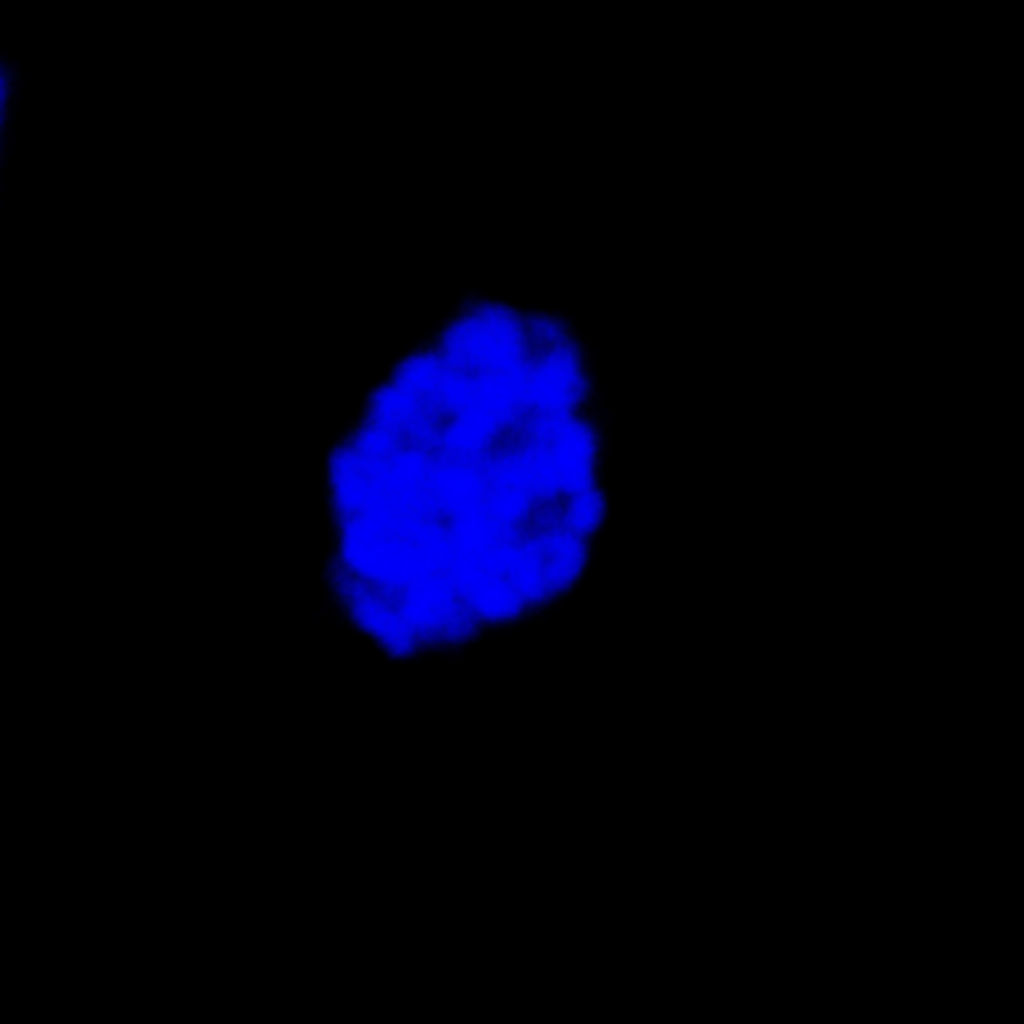

Supplement: Supplementary file 7 — Source data Fig. 5 [file 44318_2024_169_MOESM7_ESM.zip › SD_Figure_5.zip/Figure 5/5G/EV_UT.tif]

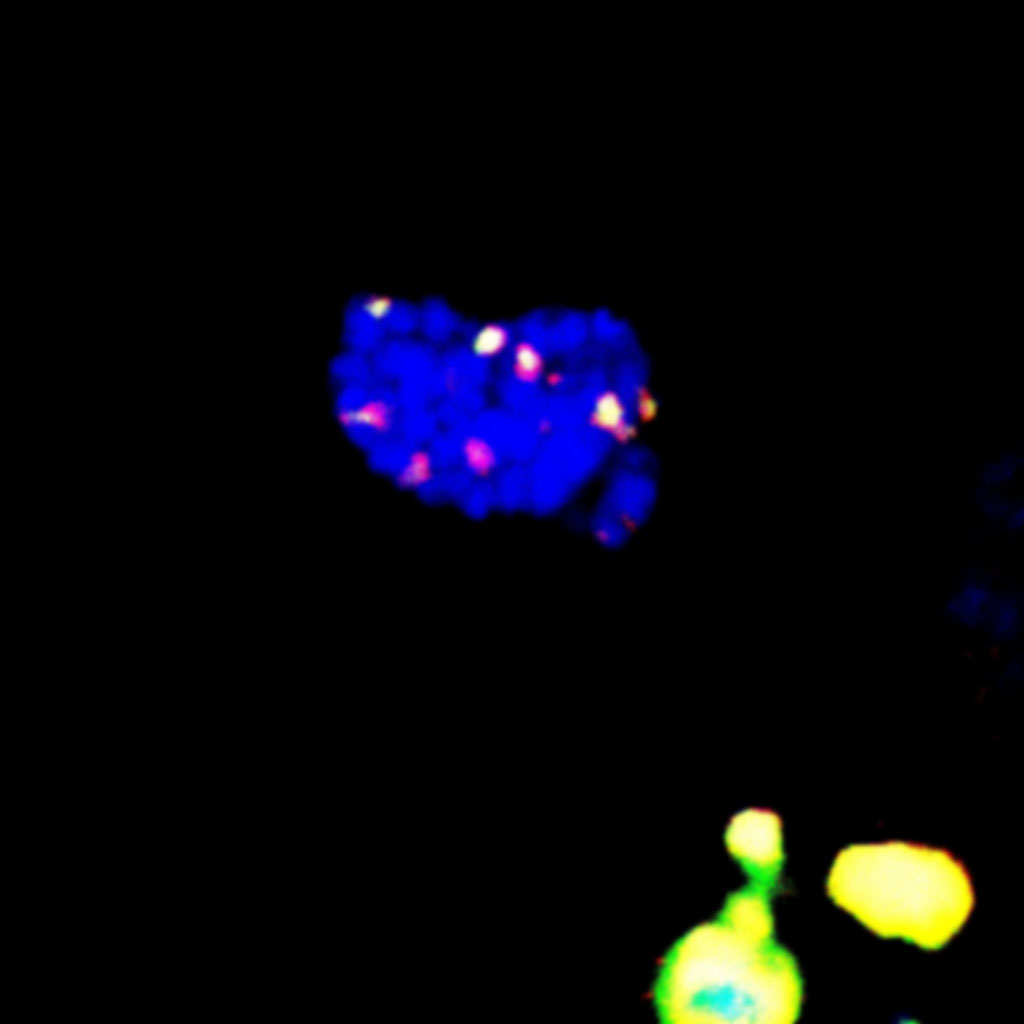

Supplement: Supplementary file 7 — Source data Fig. 5 [file 44318_2024_169_MOESM7_ESM.zip › SD_Figure_5.zip/Figure 5/5G/S61A_CPT.jpg]

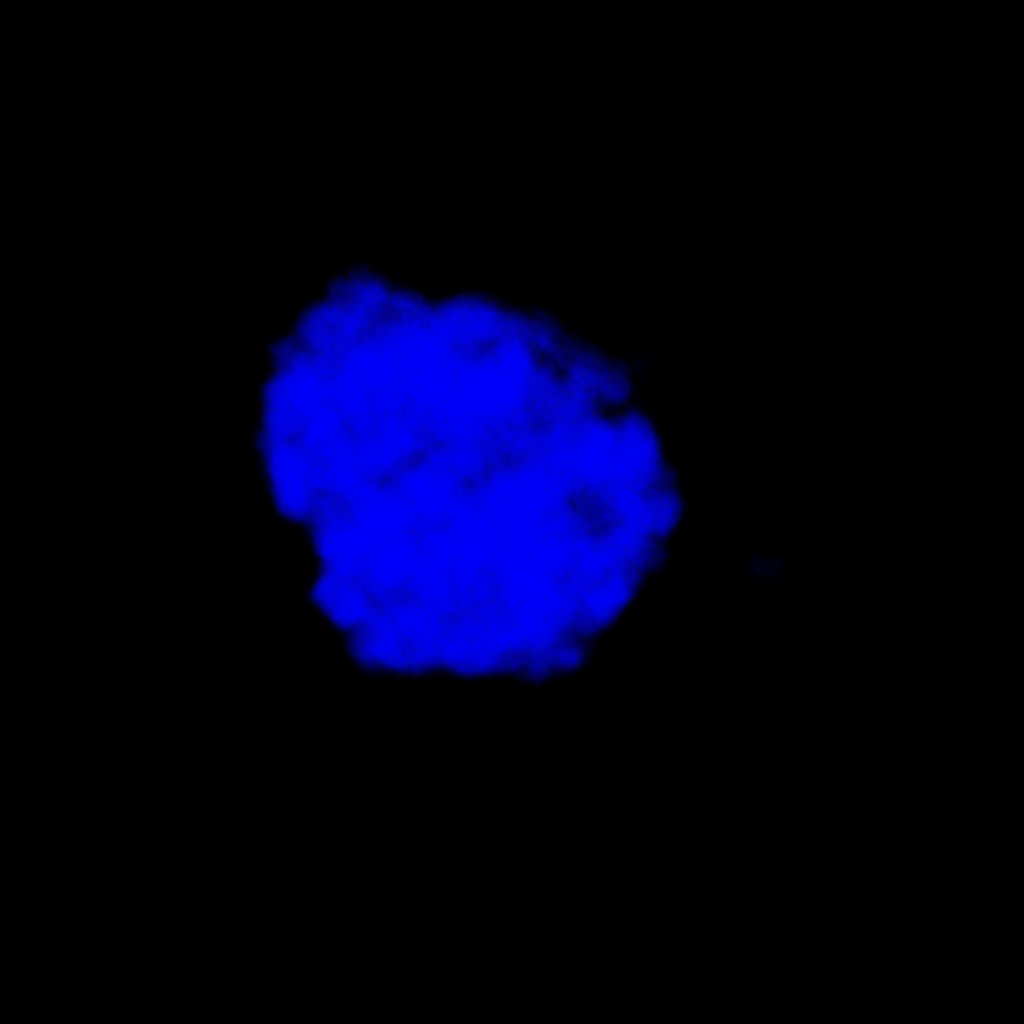

Supplement: Supplementary file 7 — Source data Fig. 5 [file 44318_2024_169_MOESM7_ESM.zip › SD_Figure_5.zip/Figure 5/5G/S61A_UT.tif]

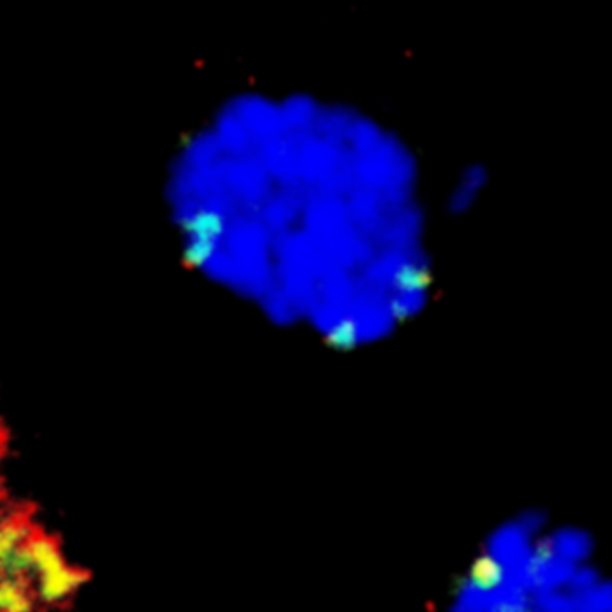

Supplement: Supplementary file 7 — Source data Fig. 5 [file 44318_2024_169_MOESM7_ESM.zip › SD_Figure_5.zip/Figure 5/5G/WT_CPT.tif]

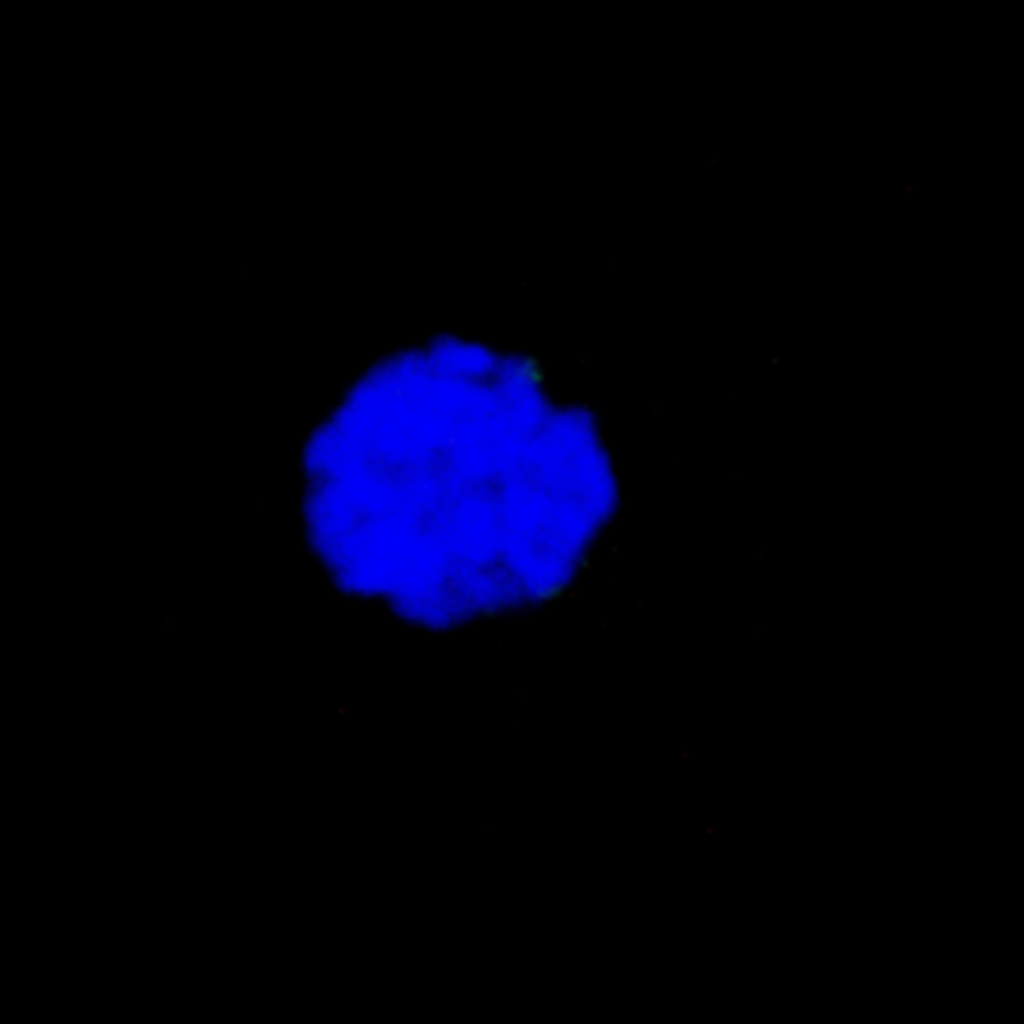

Supplement: Supplementary file 7 — Source data Fig. 5 [file 44318_2024_169_MOESM7_ESM.zip › SD_Figure_5.zip/Figure 5/5G/WT_UT.tif]

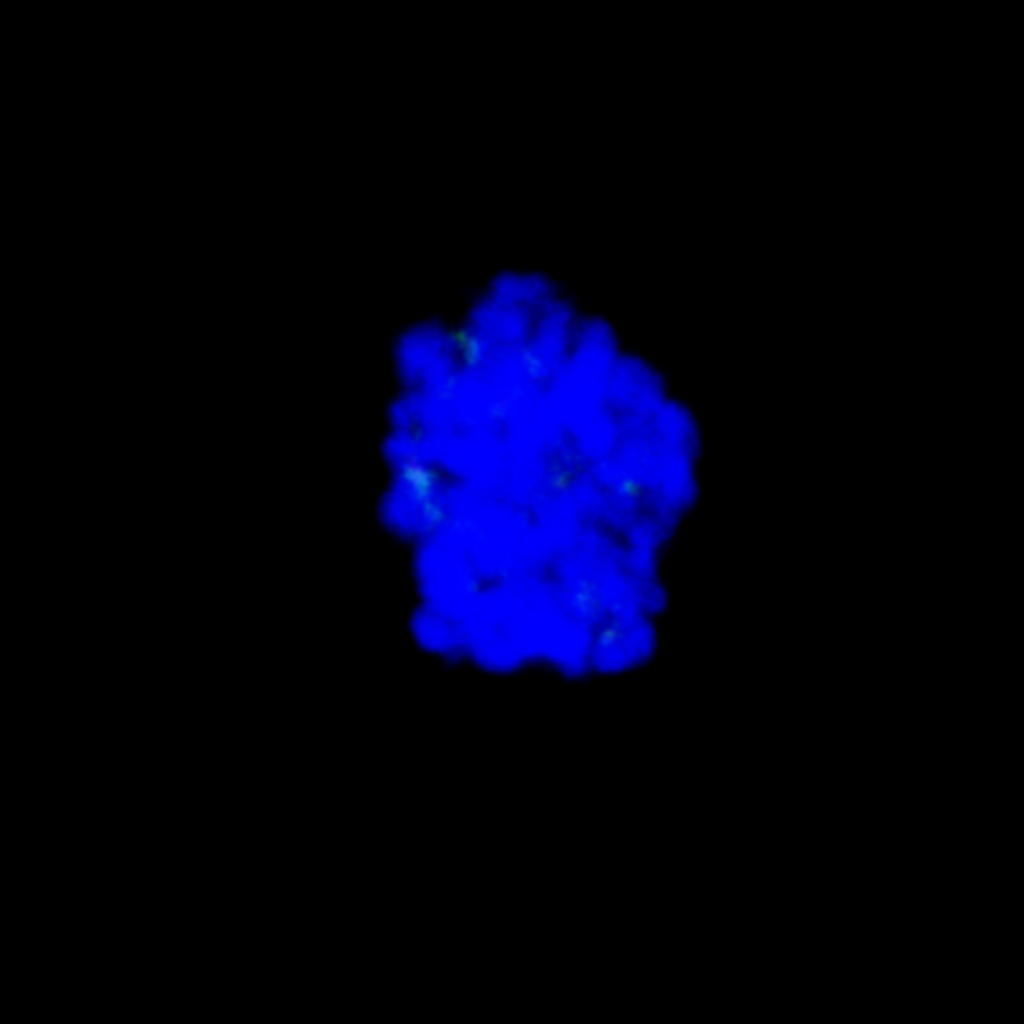

Supplement: Supplementary file 8 — Source data Fig. 6 [file 44318_2024_169_MOESM8_ESM.zip › SD_Figure_6.zip/Figure 6/6A/EV.tif]

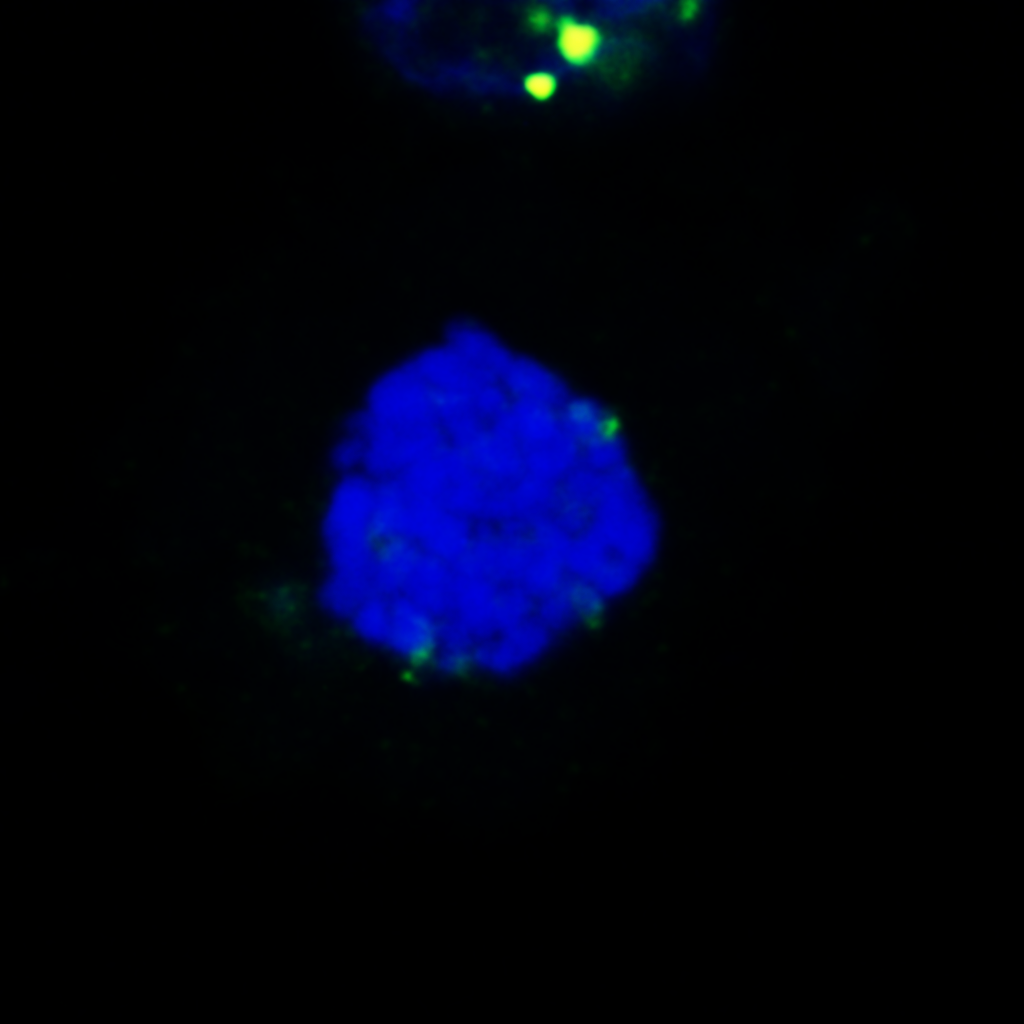

Supplement: Supplementary file 8 — Source data Fig. 6 [file 44318_2024_169_MOESM8_ESM.zip › SD_Figure_6.zip/Figure 6/6A/S61A.tif]

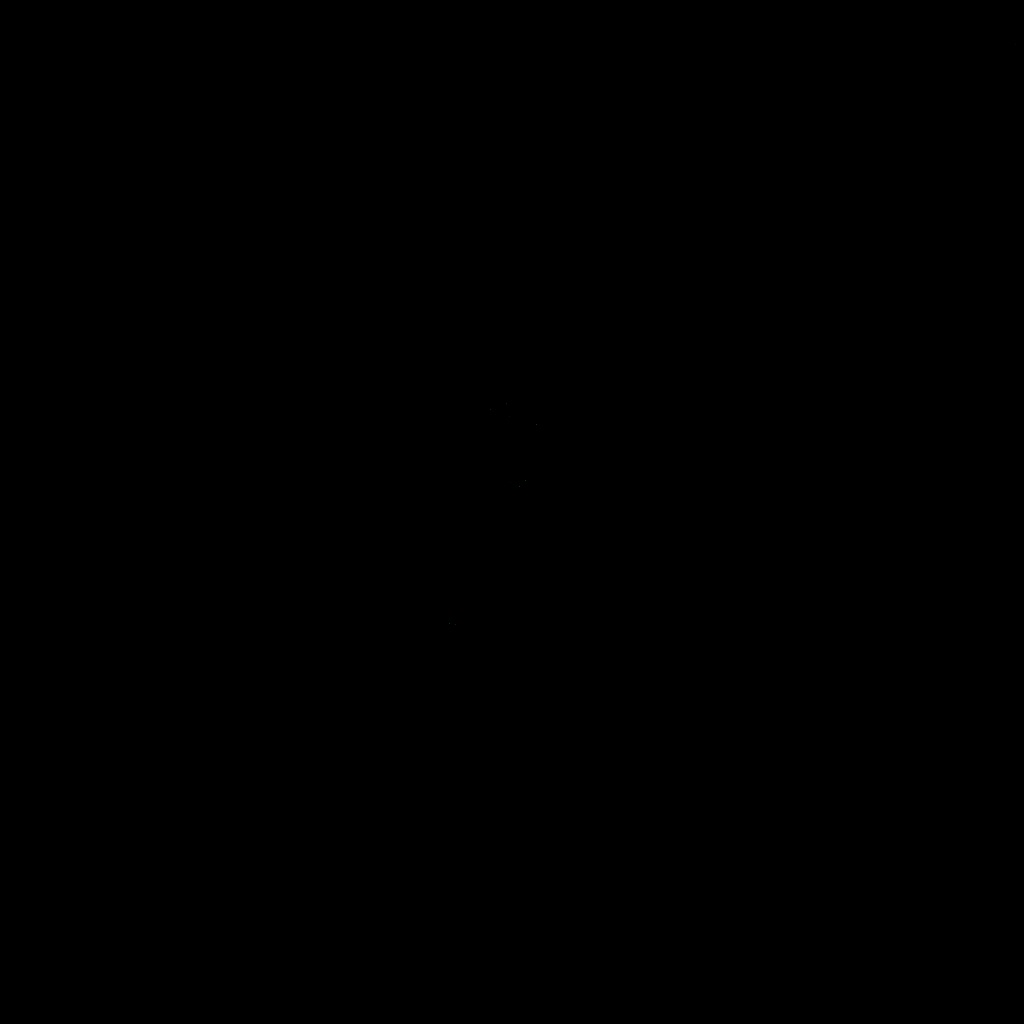

Supplement: Supplementary file 8 — Source data Fig. 6 [file 44318_2024_169_MOESM8_ESM.zip › SD_Figure_6.zip/Figure 6/6A/WT.tif]

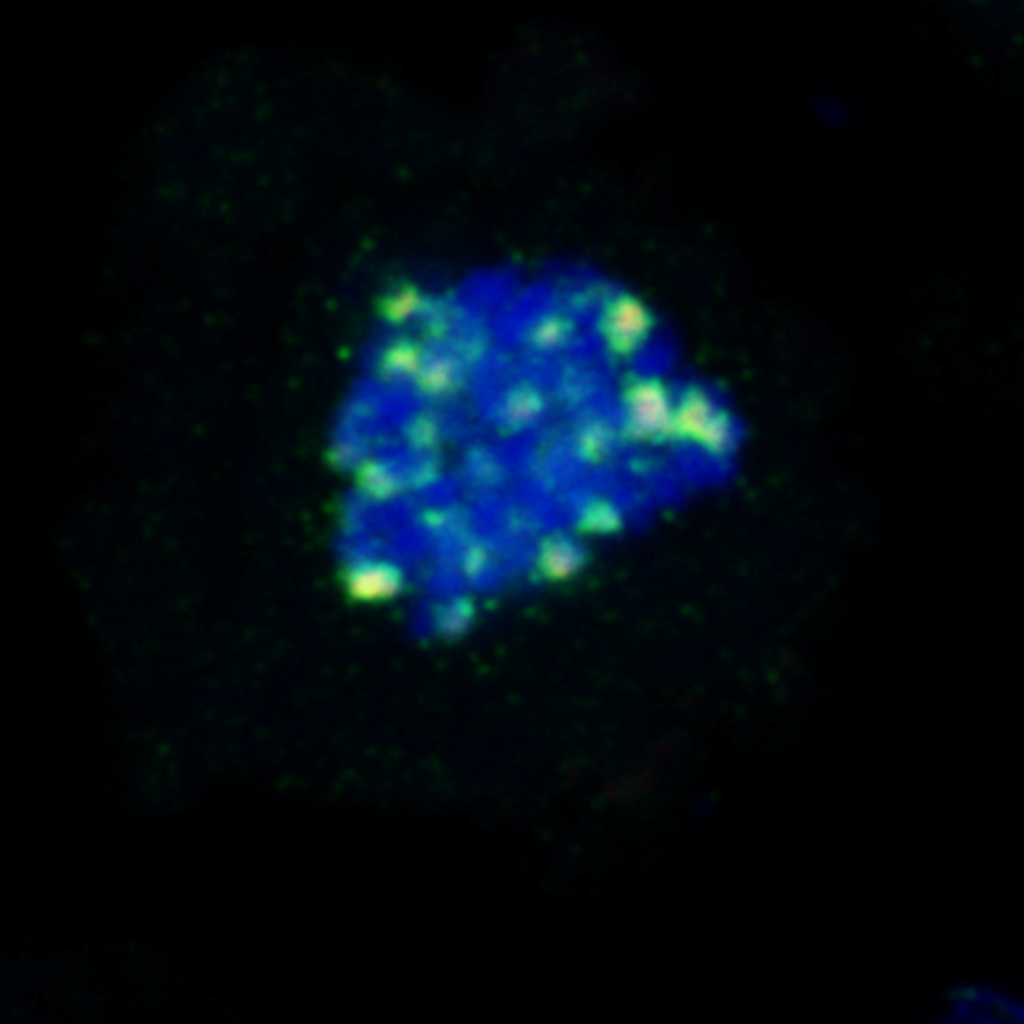

Supplement: Supplementary file 8 — Source data Fig. 6 [file 44318_2024_169_MOESM8_ESM.zip › SD_Figure_6.zip/Figure 6/6B/EV.tif]

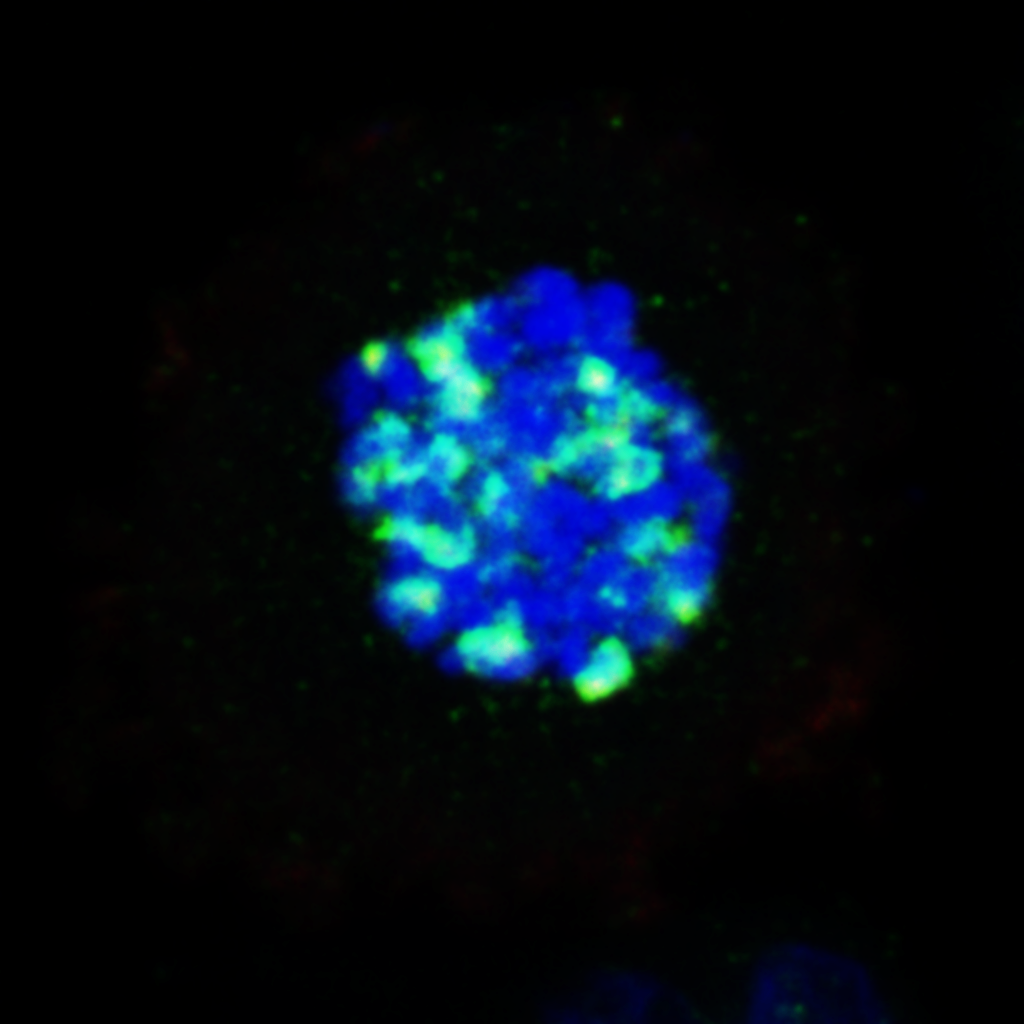

Supplement: Supplementary file 8 — Source data Fig. 6 [file 44318_2024_169_MOESM8_ESM.zip › SD_Figure_6.zip/Figure 6/6B/S61A.tif]

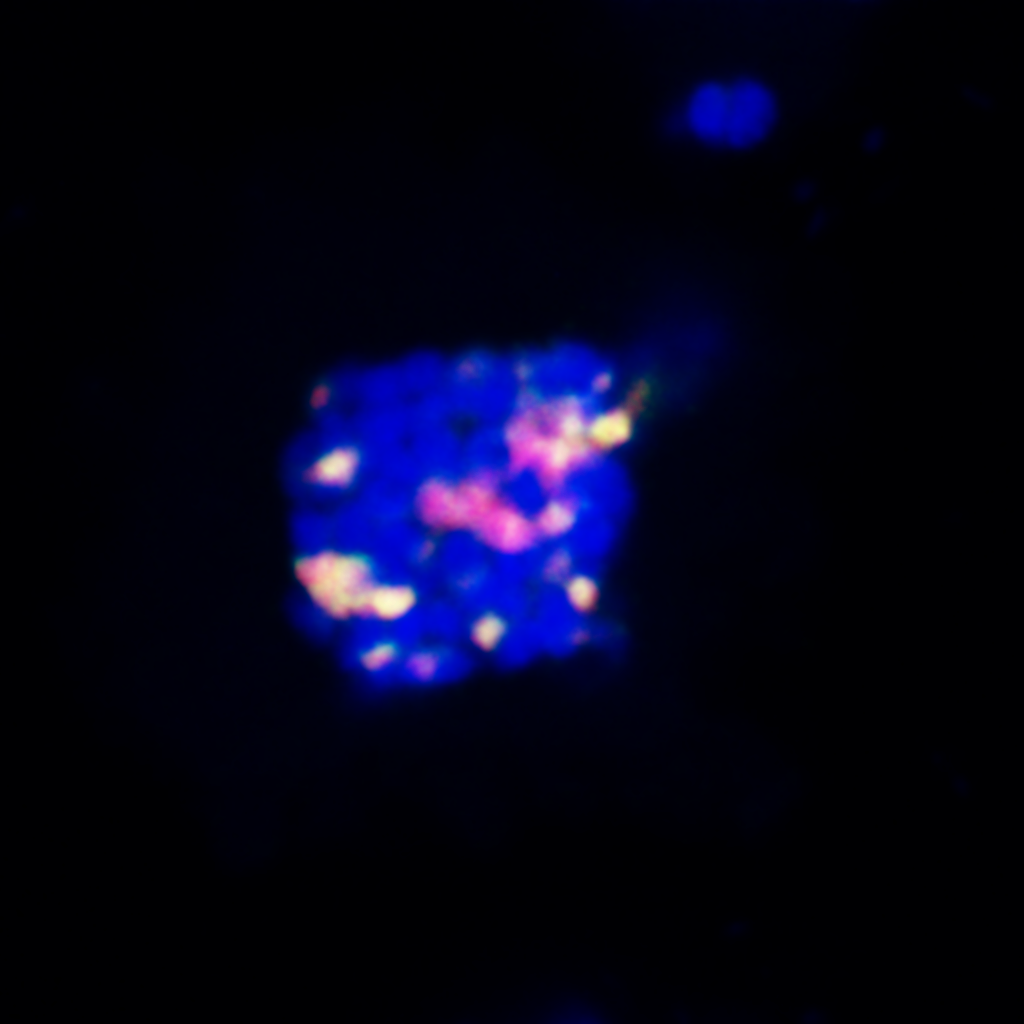

Supplement: Supplementary file 8 — Source data Fig. 6 [file 44318_2024_169_MOESM8_ESM.zip › SD_Figure_6.zip/Figure 6/6B/WT.tif]

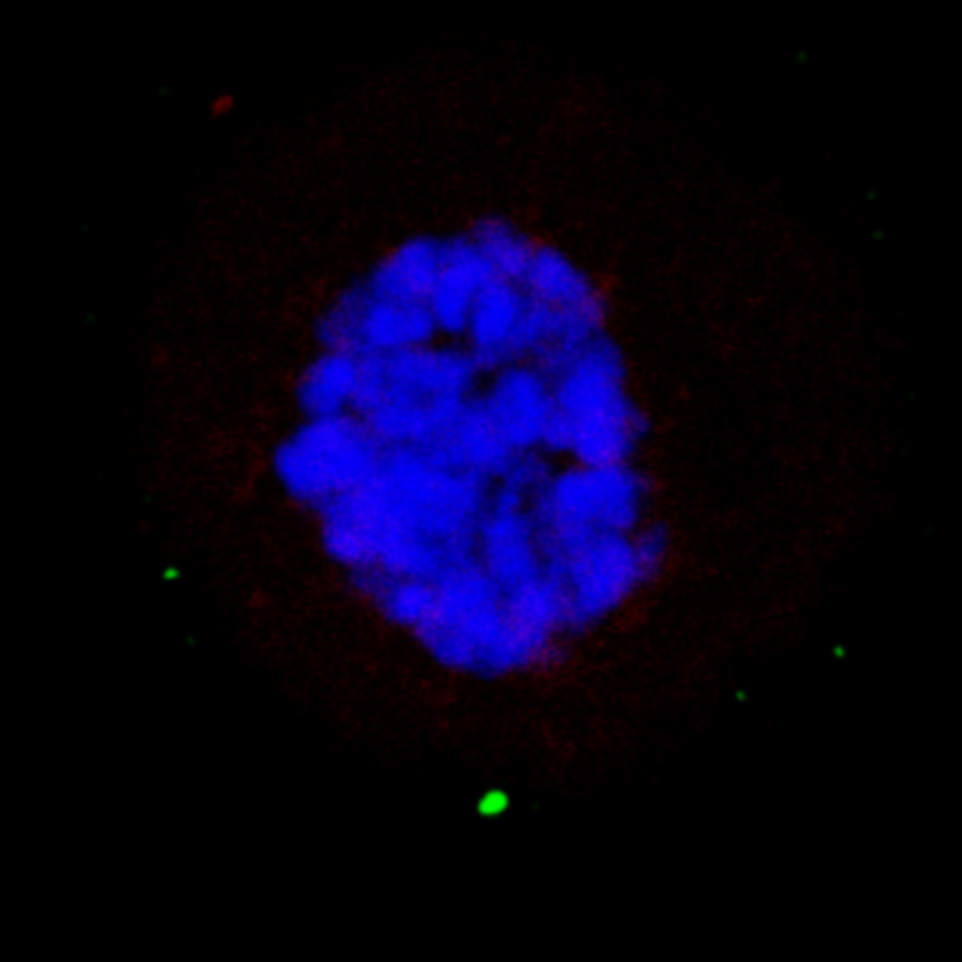

Supplement: Supplementary file 8 — Source data Fig. 6 [file 44318_2024_169_MOESM8_ESM.zip › SD_Figure_6.zip/Figure 6/6C/EV.tif]

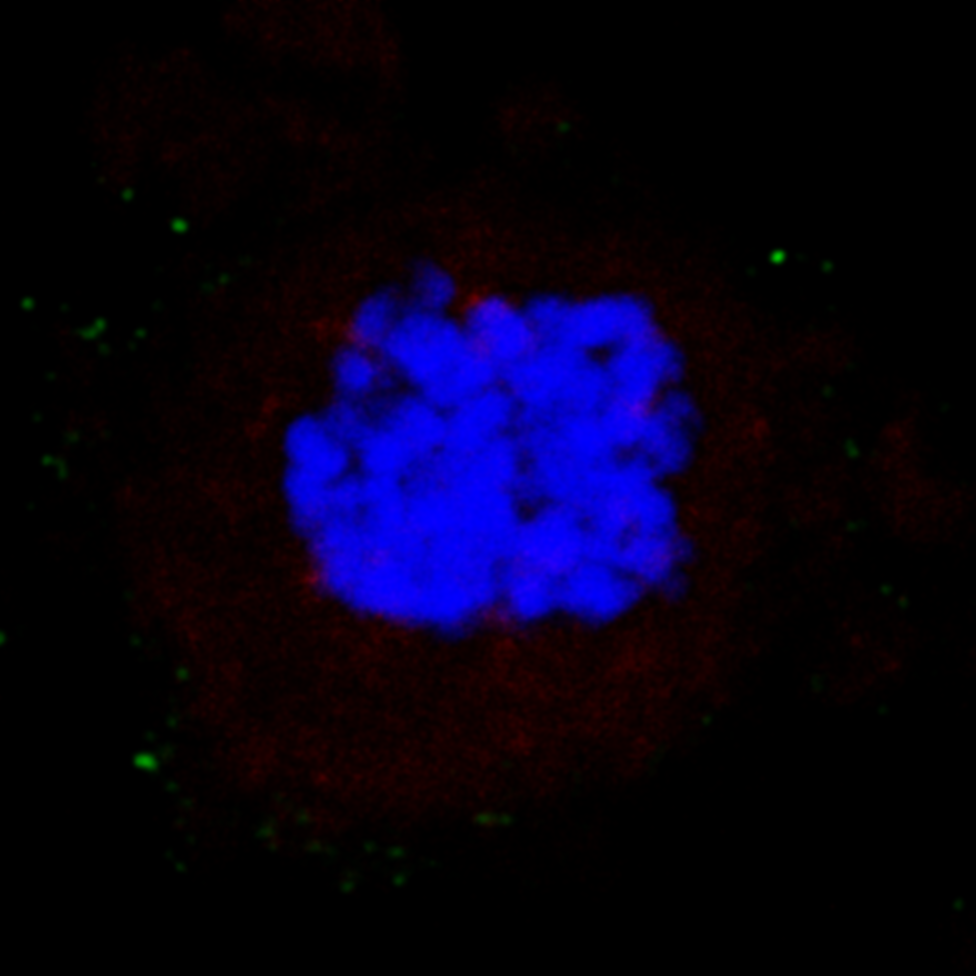

Supplement: Supplementary file 8 — Source data Fig. 6 [file 44318_2024_169_MOESM8_ESM.zip › SD_Figure_6.zip/Figure 6/6C/S61A.tif]

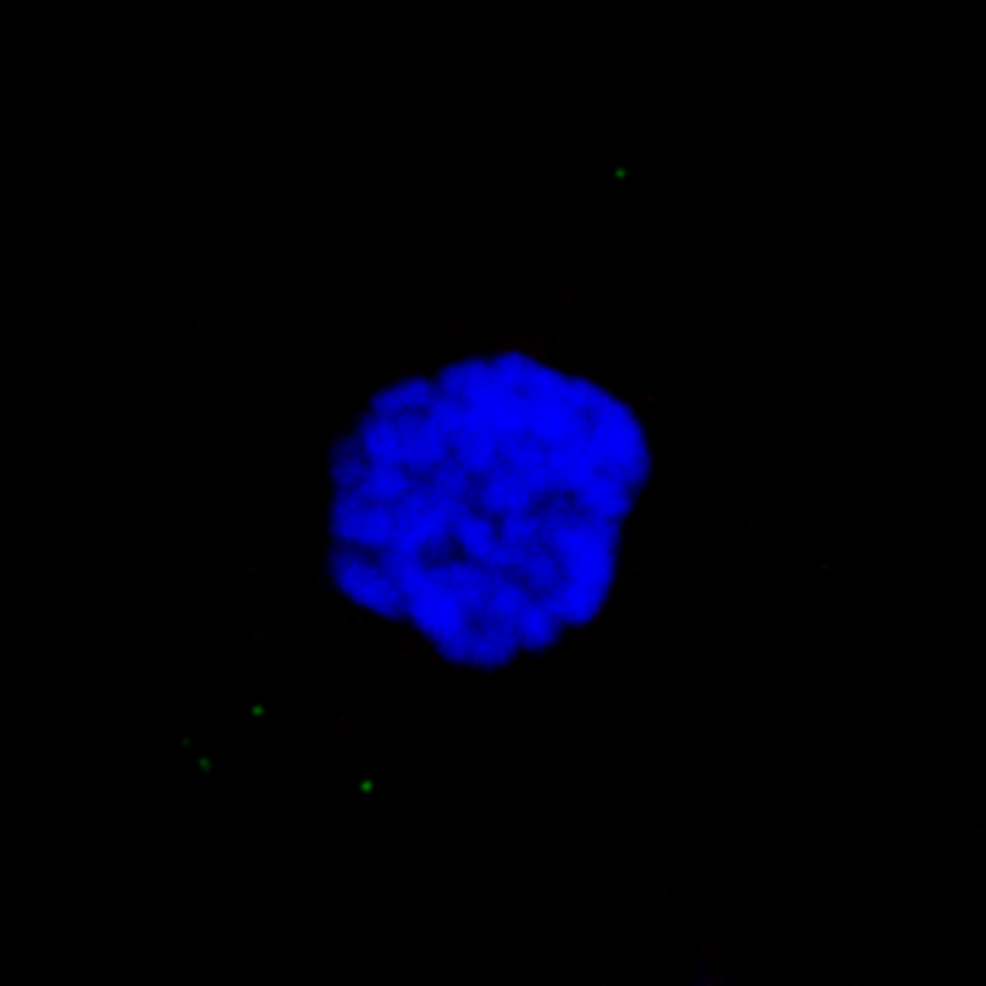

Supplement: Supplementary file 8 — Source data Fig. 6 [file 44318_2024_169_MOESM8_ESM.zip › SD_Figure_6.zip/Figure 6/6C/WT.tif]

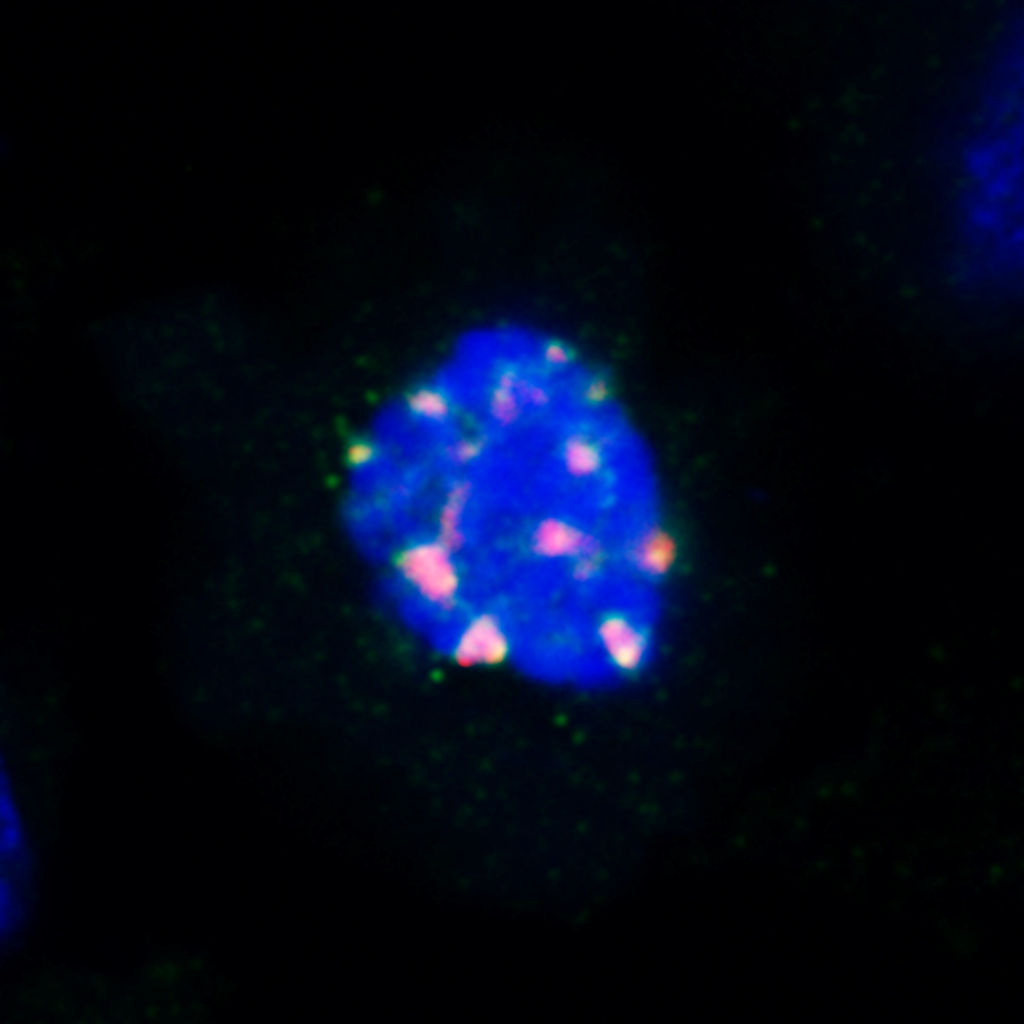

Supplement: Supplementary file 8 — Source data Fig. 6 [file 44318_2024_169_MOESM8_ESM.zip › SD_Figure_6.zip/Figure 6/6D/EV.tif]

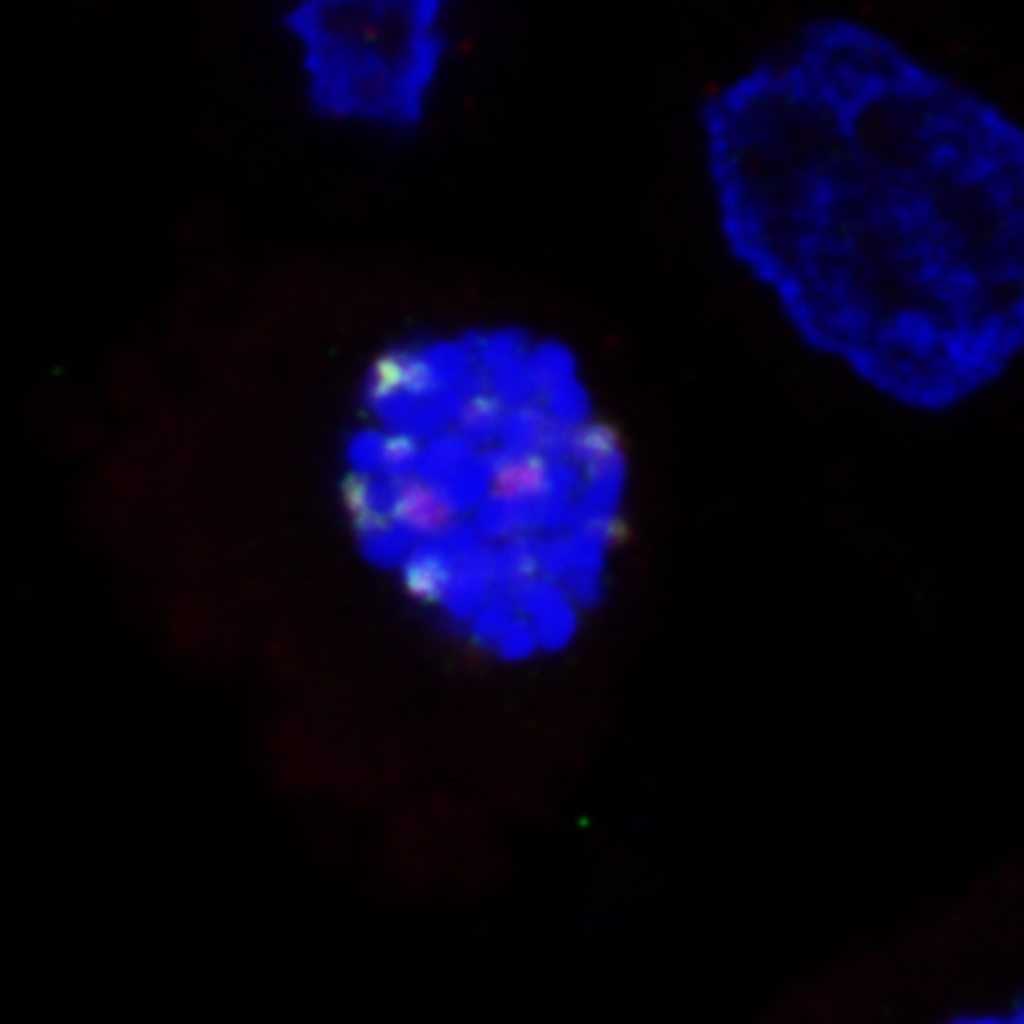

Supplement: Supplementary file 8 — Source data Fig. 6 [file 44318_2024_169_MOESM8_ESM.zip › SD_Figure_6.zip/Figure 6/6D/S61A.tif]

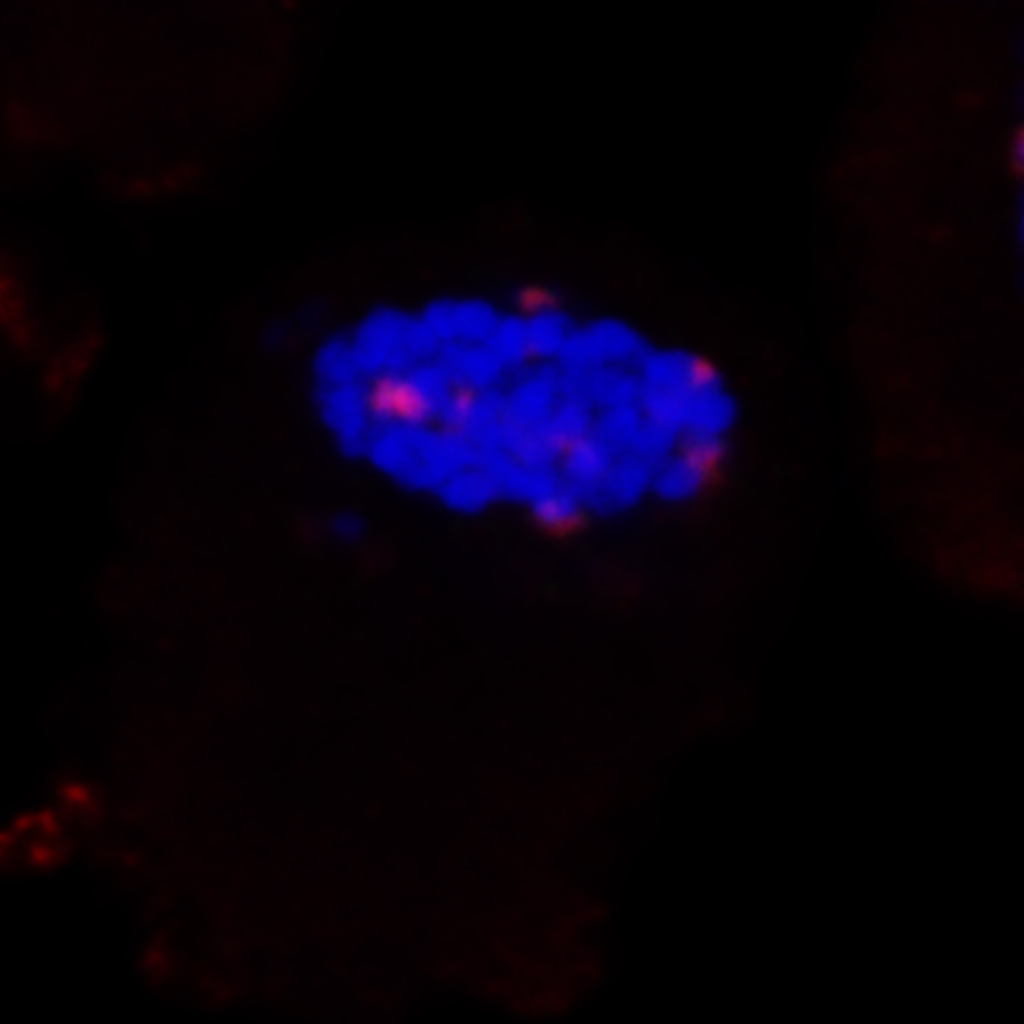

Supplement: Supplementary file 8 — Source data Fig. 6 [file 44318_2024_169_MOESM8_ESM.zip › SD_Figure_6.zip/Figure 6/6D/WT.tif]

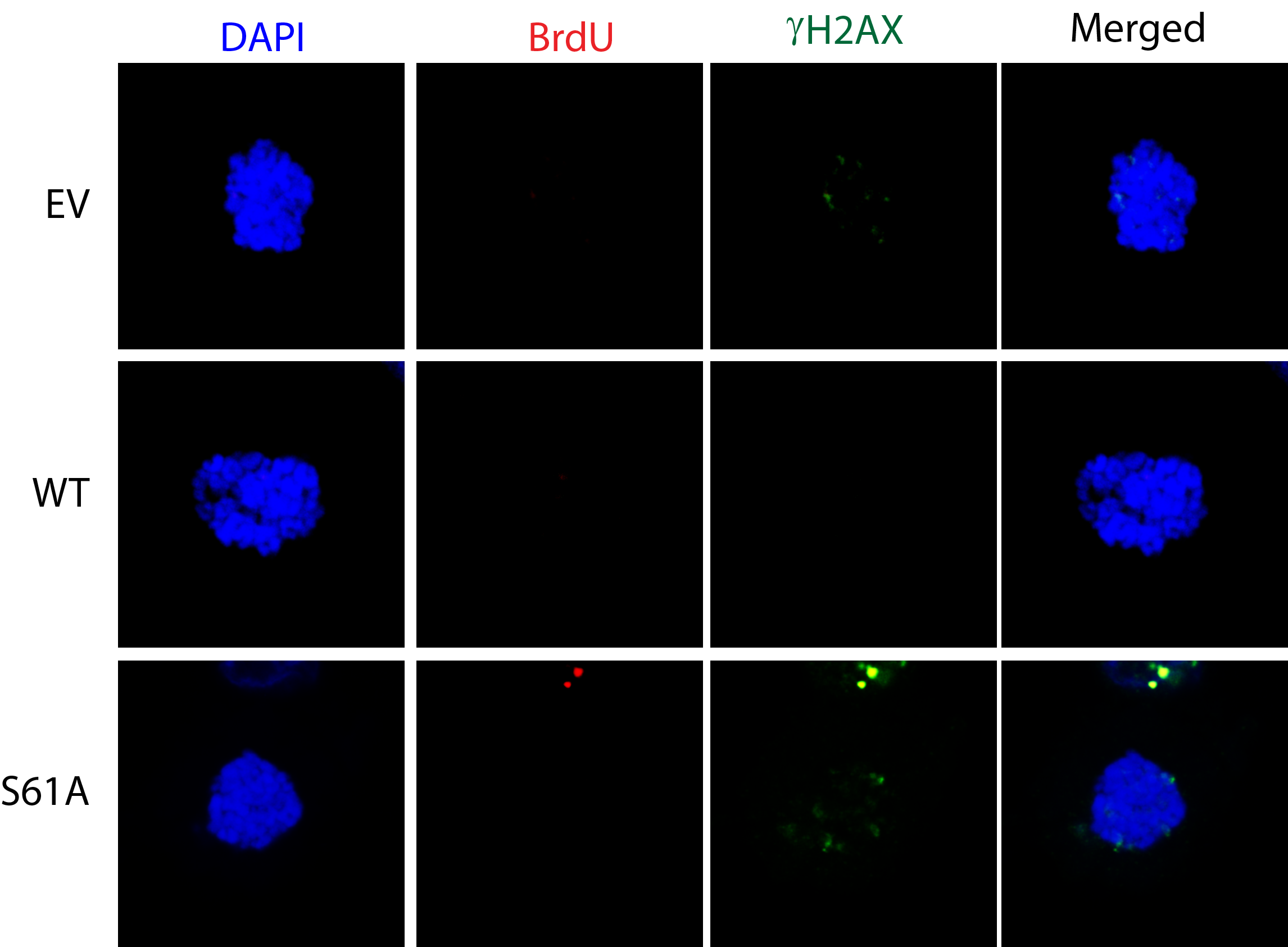

Supplement: Supplementary file 8 — Source data Fig. 6 [file 44318_2024_169_MOESM8_ESM.zip › SD_Figure_6.zip/Figure 6/Fig 6A.tif]

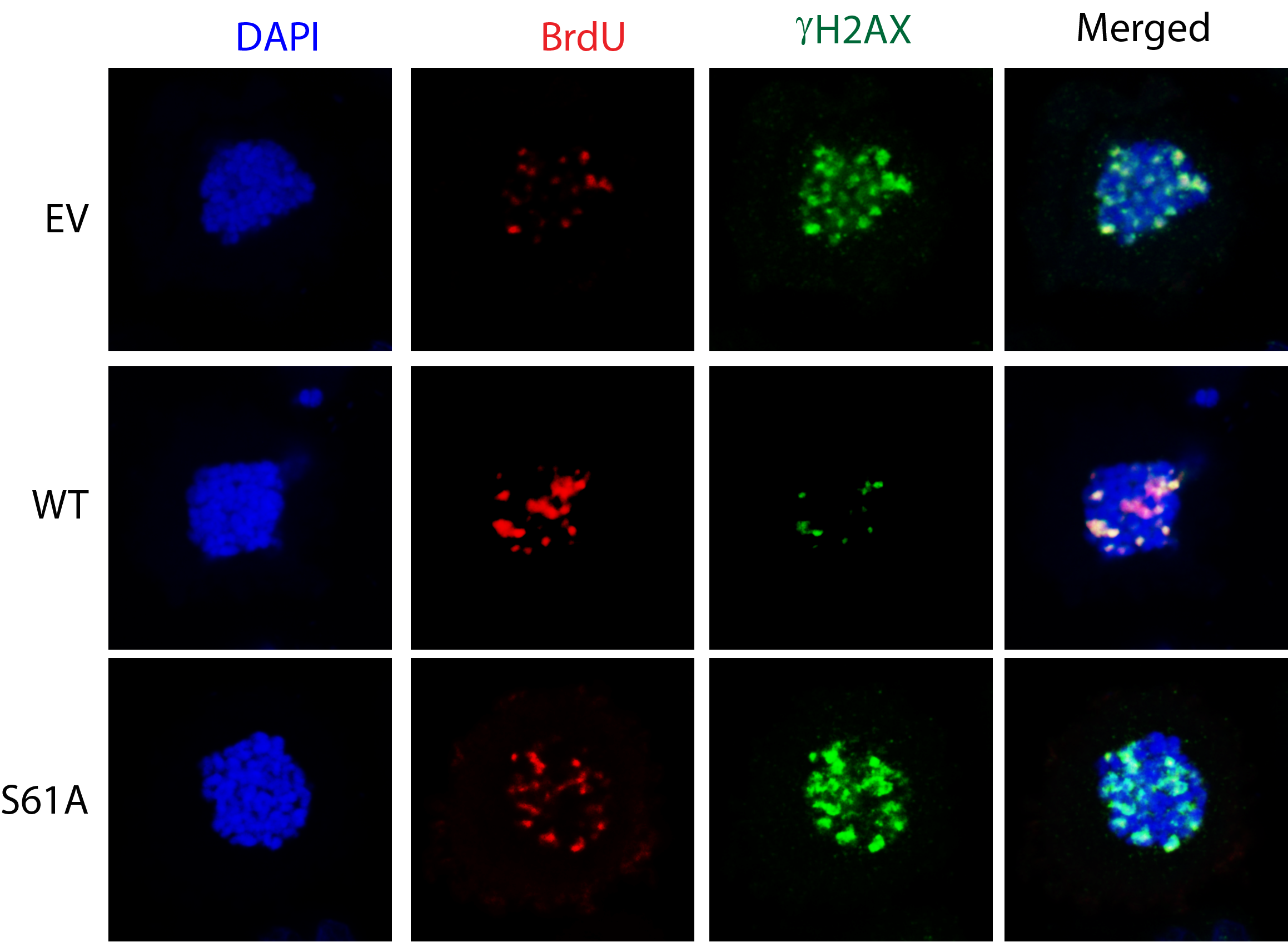

Supplement: Supplementary file 8 — Source data Fig. 6 [file 44318_2024_169_MOESM8_ESM.zip › SD_Figure_6.zip/Figure 6/Fig 6B.tif]

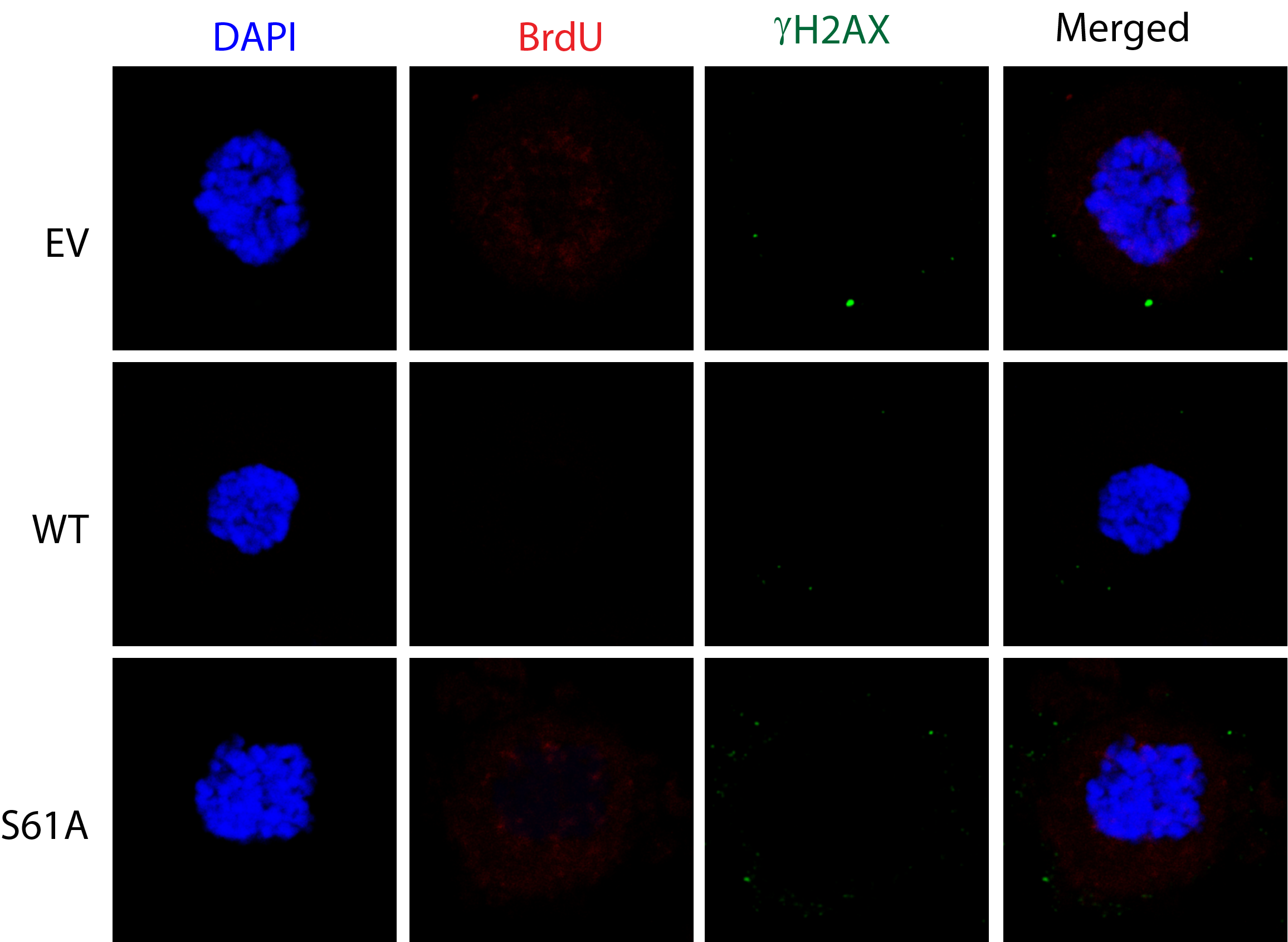

Supplement: Supplementary file 8 — Source data Fig. 6 [file 44318_2024_169_MOESM8_ESM.zip › SD_Figure_6.zip/Figure 6/Fig 6C.tif]

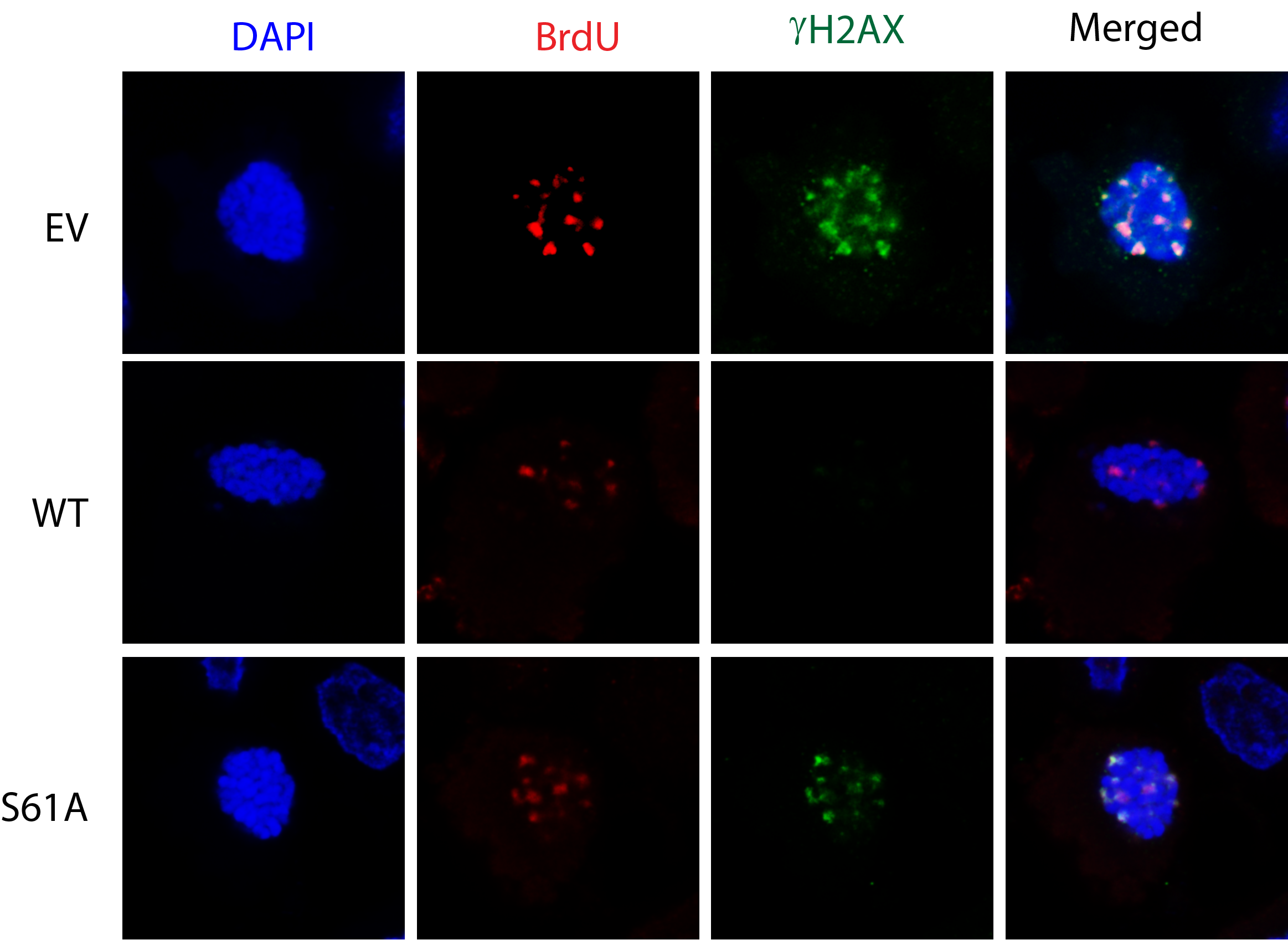

Supplement: Supplementary file 8 — Source data Fig. 6 [file 44318_2024_169_MOESM8_ESM.zip › SD_Figure_6.zip/Figure 6/Fig 6D.tif]

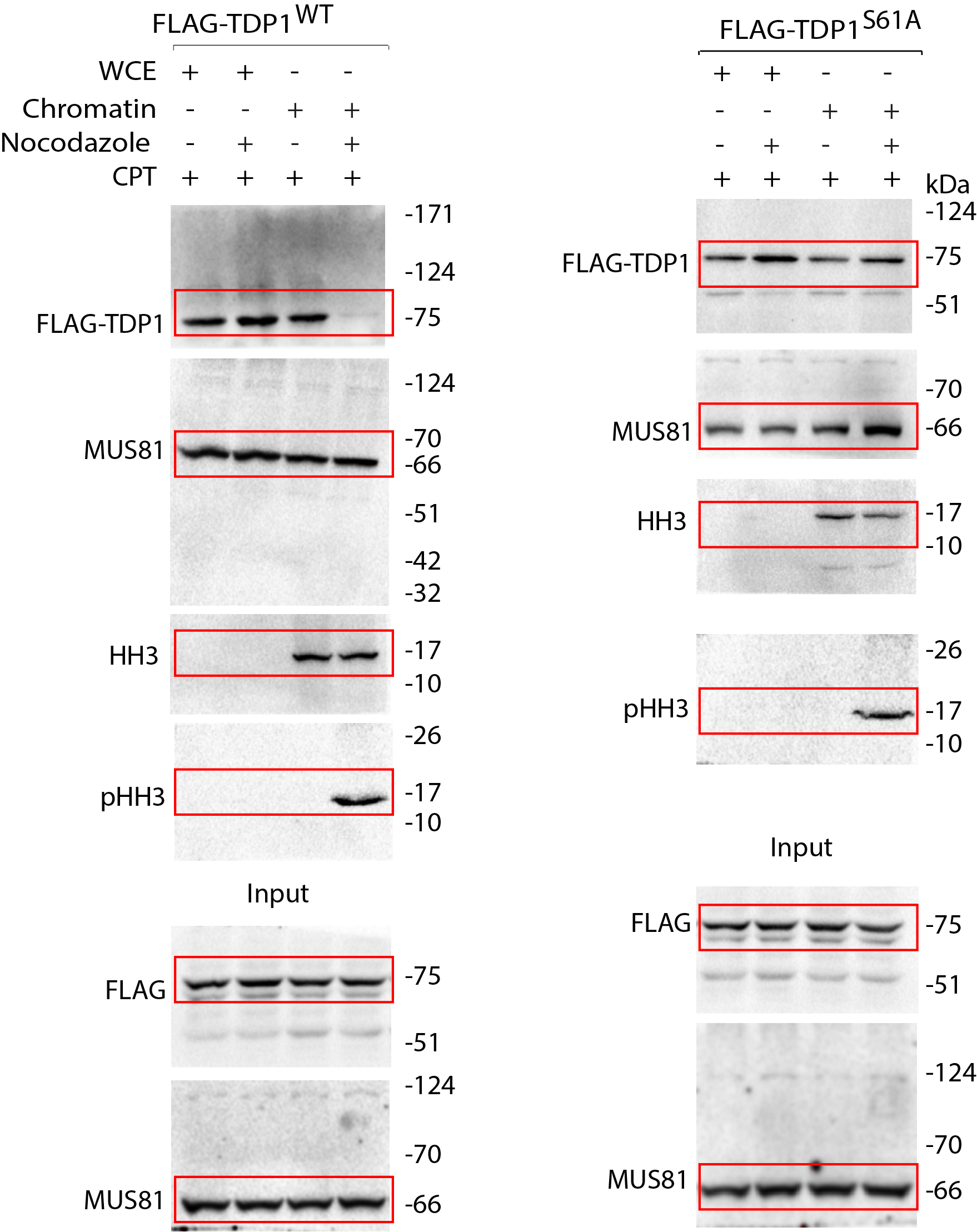

Supplement: Supplementary file 8 — Source data Fig. 6 [file 44318_2024_169_MOESM8_ESM.zip › SD_Figure_6.zip/Figure 6/Fig 6G/Fig 6G.tif]

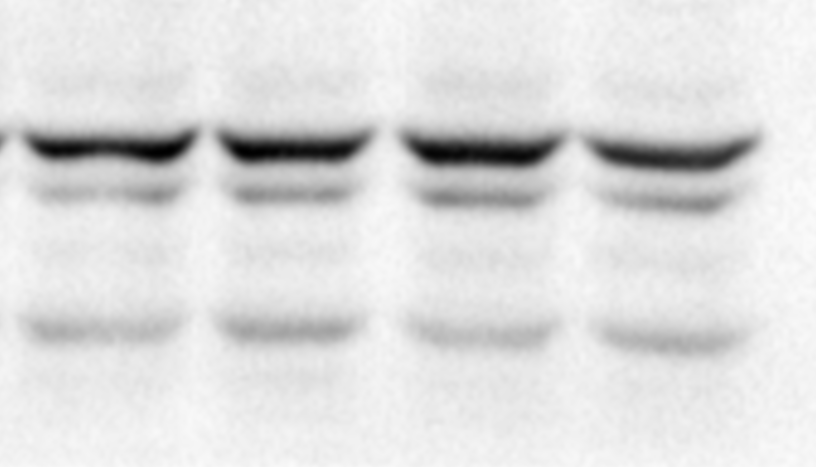

Supplement: Supplementary file 8 — Source data Fig. 6 [file 44318_2024_169_MOESM8_ESM.zip › SD_Figure_6.zip/Figure 6/Fig 6G/FLAGTDP1S61A input.tif]

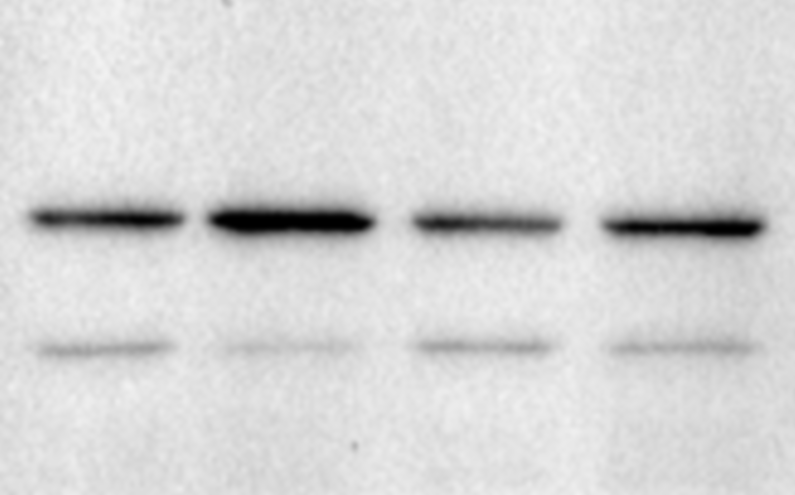

Supplement: Supplementary file 8 — Source data Fig. 6 [file 44318_2024_169_MOESM8_ESM.zip › SD_Figure_6.zip/Figure 6/Fig 6G/FLAGTDP1S61A.tif]

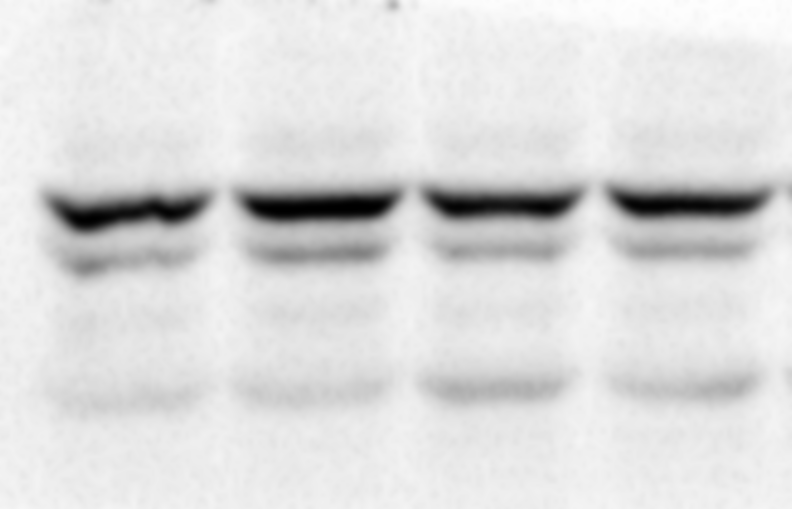

Supplement: Supplementary file 8 — Source data Fig. 6 [file 44318_2024_169_MOESM8_ESM.zip › SD_Figure_6.zip/Figure 6/Fig 6G/FLAGTDP1WT input.tif]

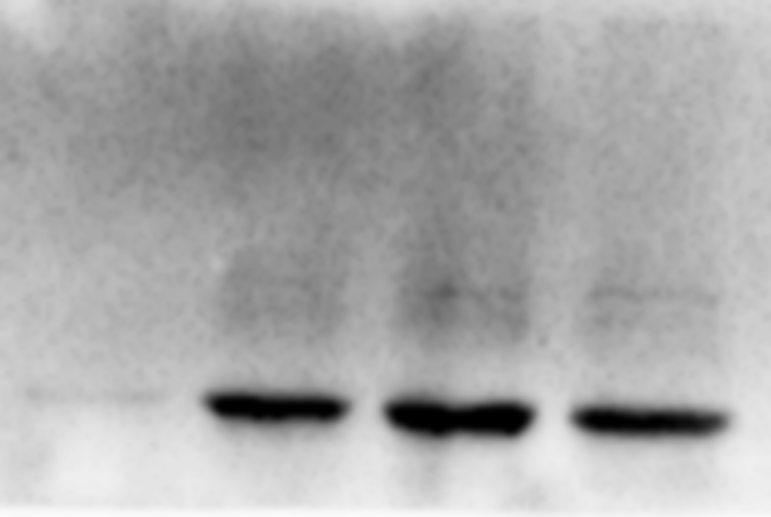

Supplement: Supplementary file 8 — Source data Fig. 6 [file 44318_2024_169_MOESM8_ESM.zip › SD_Figure_6.zip/Figure 6/Fig 6G/FLAGTDP1WT.tif]

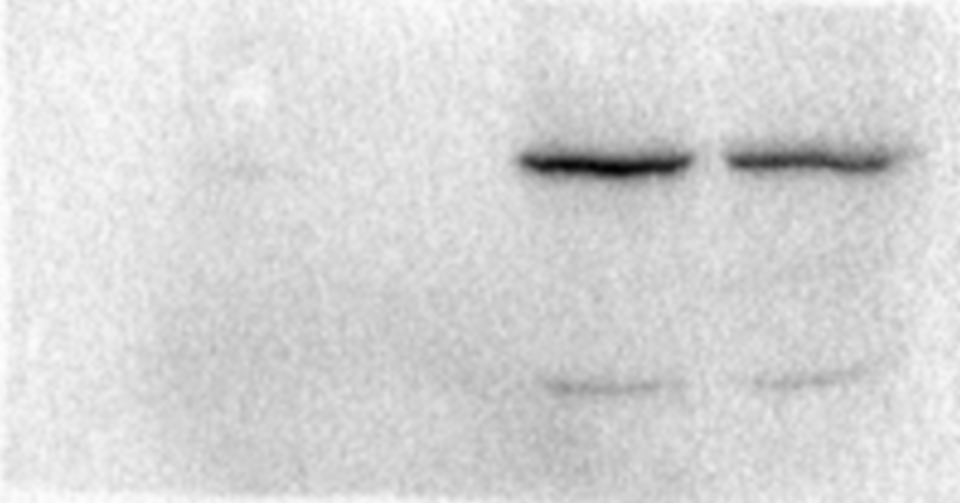

Supplement: Supplementary file 8 — Source data Fig. 6 [file 44318_2024_169_MOESM8_ESM.zip › SD_Figure_6.zip/Figure 6/Fig 6G/HH3 S61A.tif]

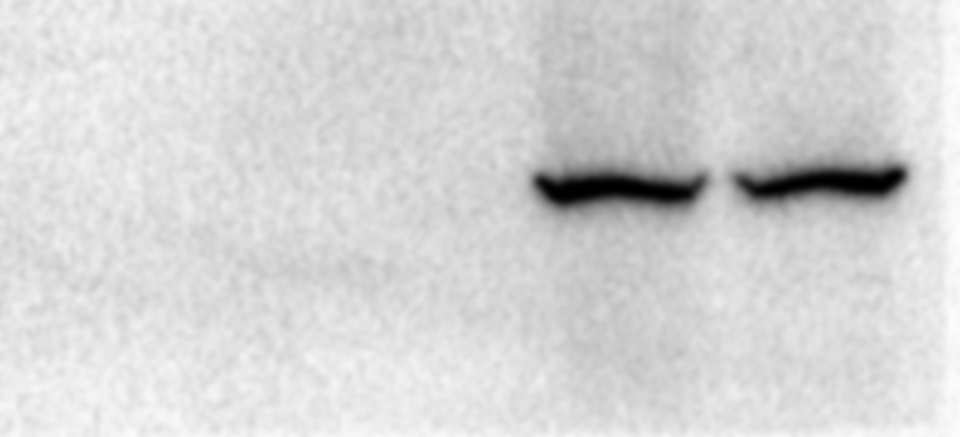

Supplement: Supplementary file 8 — Source data Fig. 6 [file 44318_2024_169_MOESM8_ESM.zip › SD_Figure_6.zip/Figure 6/Fig 6G/HH3 WT.tif]

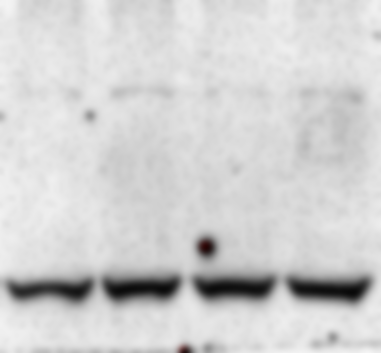

Supplement: Supplementary file 8 — Source data Fig. 6 [file 44318_2024_169_MOESM8_ESM.zip › SD_Figure_6.zip/Figure 6/Fig 6G/MUS81 S61A input.tif]

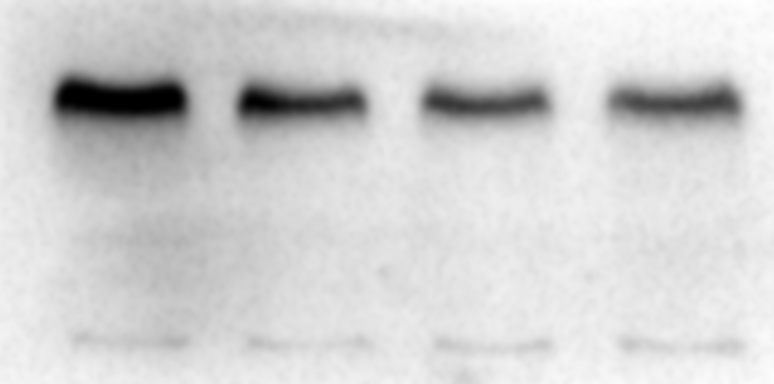

Supplement: Supplementary file 8 — Source data Fig. 6 [file 44318_2024_169_MOESM8_ESM.zip › SD_Figure_6.zip/Figure 6/Fig 6G/MUS81 S61A.tif]

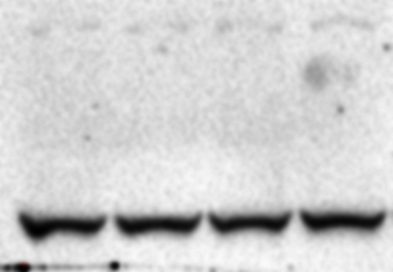

Supplement: Supplementary file 8 — Source data Fig. 6 [file 44318_2024_169_MOESM8_ESM.zip › SD_Figure_6.zip/Figure 6/Fig 6G/MUS81 WT input.tif]

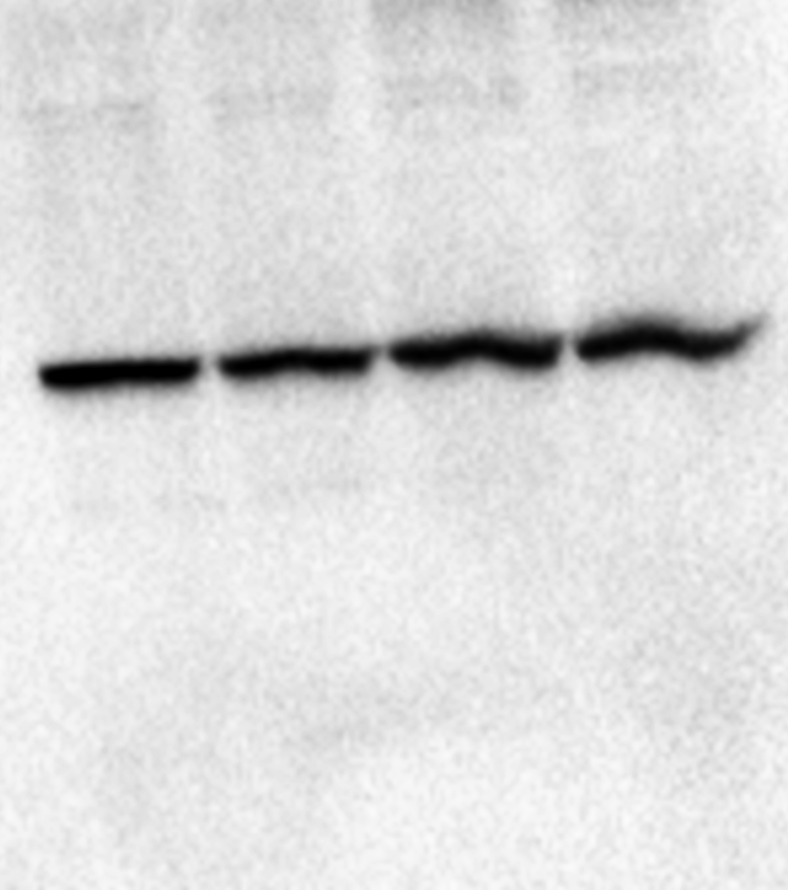

Supplement: Supplementary file 8 — Source data Fig. 6 [file 44318_2024_169_MOESM8_ESM.zip › SD_Figure_6.zip/Figure 6/Fig 6G/MUS81 WT.tif]

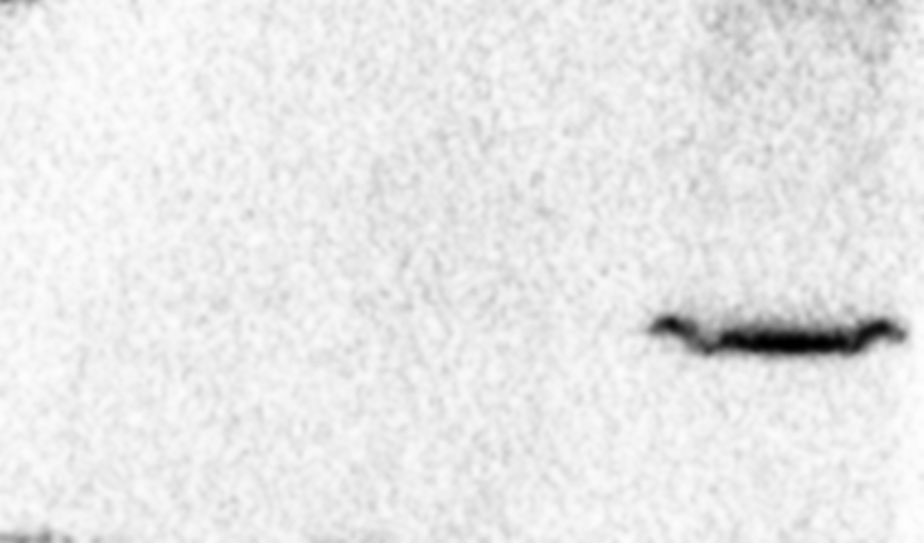

Supplement: Supplementary file 8 — Source data Fig. 6 [file 44318_2024_169_MOESM8_ESM.zip › SD_Figure_6.zip/Figure 6/Fig 6G/pHH3 S61A.tif]

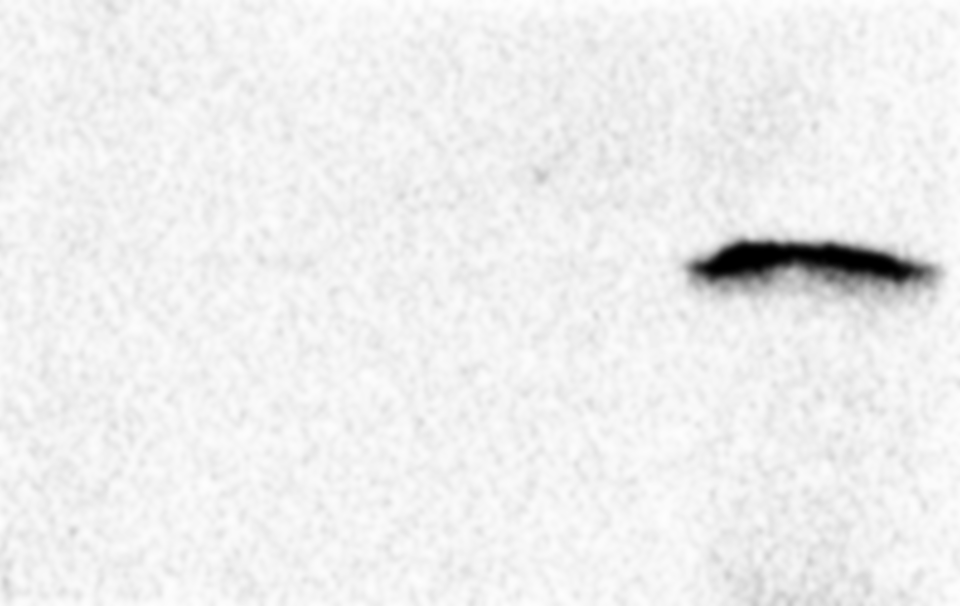

Supplement: Supplementary file 8 — Source data Fig. 6 [file 44318_2024_169_MOESM8_ESM.zip › SD_Figure_6.zip/Figure 6/Fig 6G/pHH3 WT.tif]

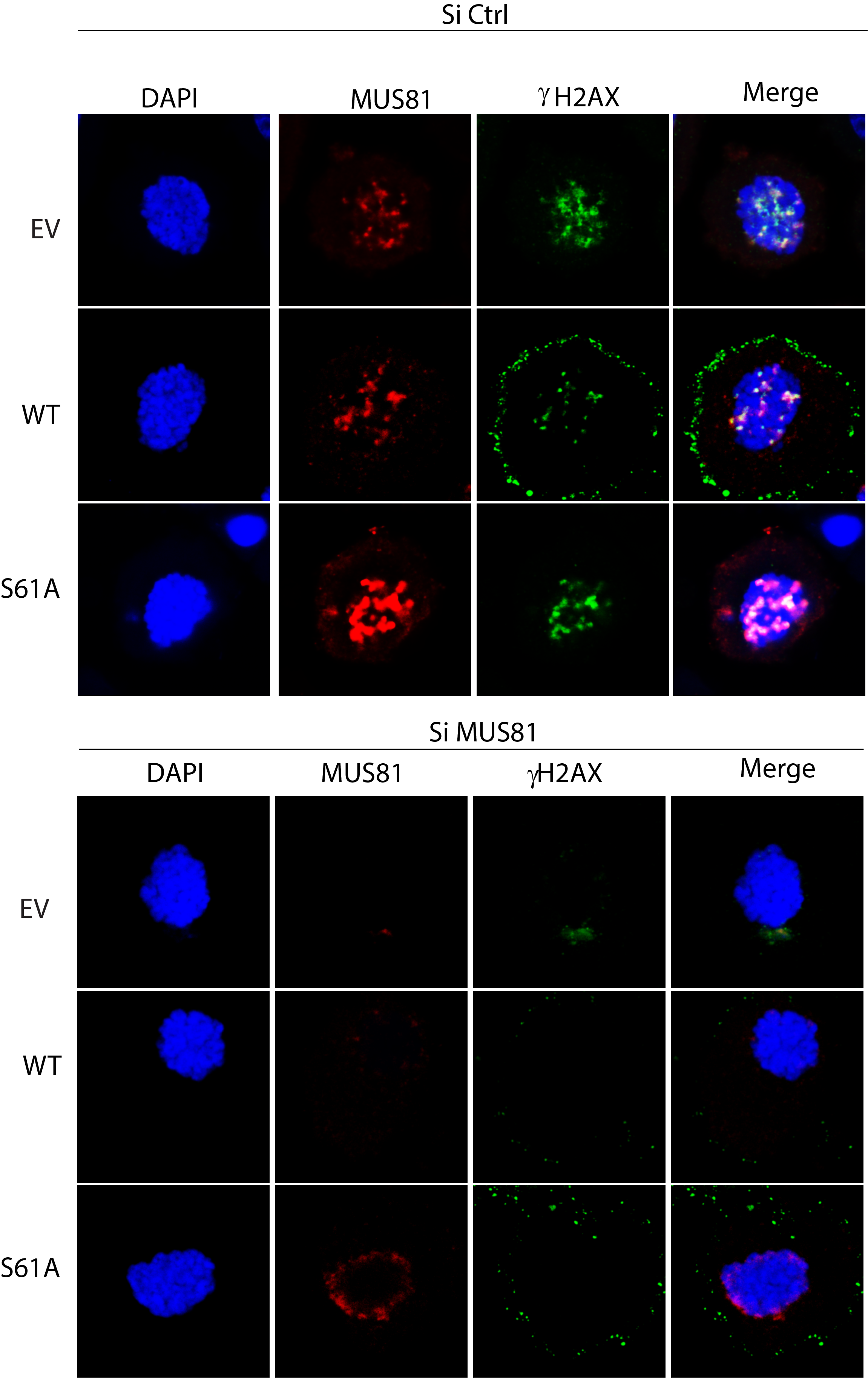

Supplement: Supplementary file 8 — Source data Fig. 6 [file 44318_2024_169_MOESM8_ESM.zip › SD_Figure_6.zip/Figure 6/Fig 6I/Fig 6I.tif]

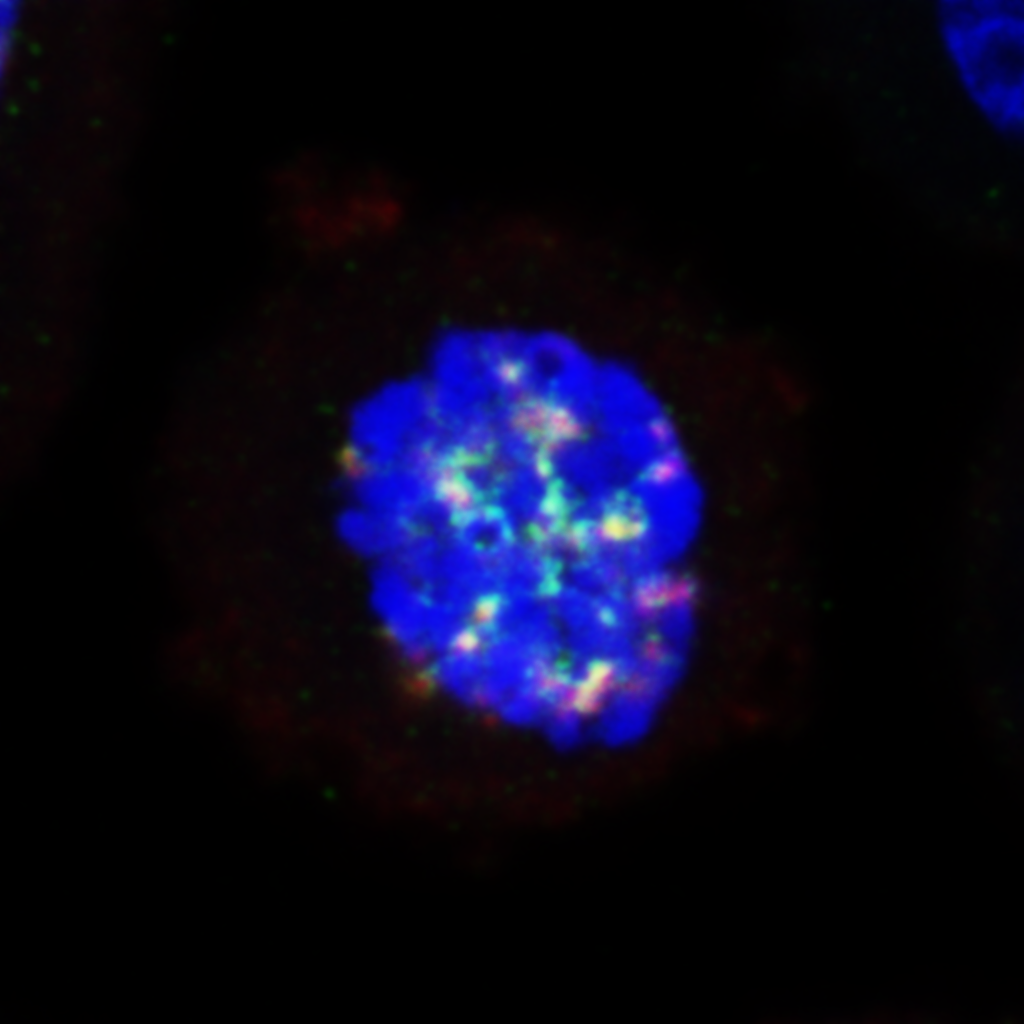

Supplement: Supplementary file 8 — Source data Fig. 6 [file 44318_2024_169_MOESM8_ESM.zip › SD_Figure_6.zip/Figure 6/Fig 6I/Si Ctr/EV.tif]

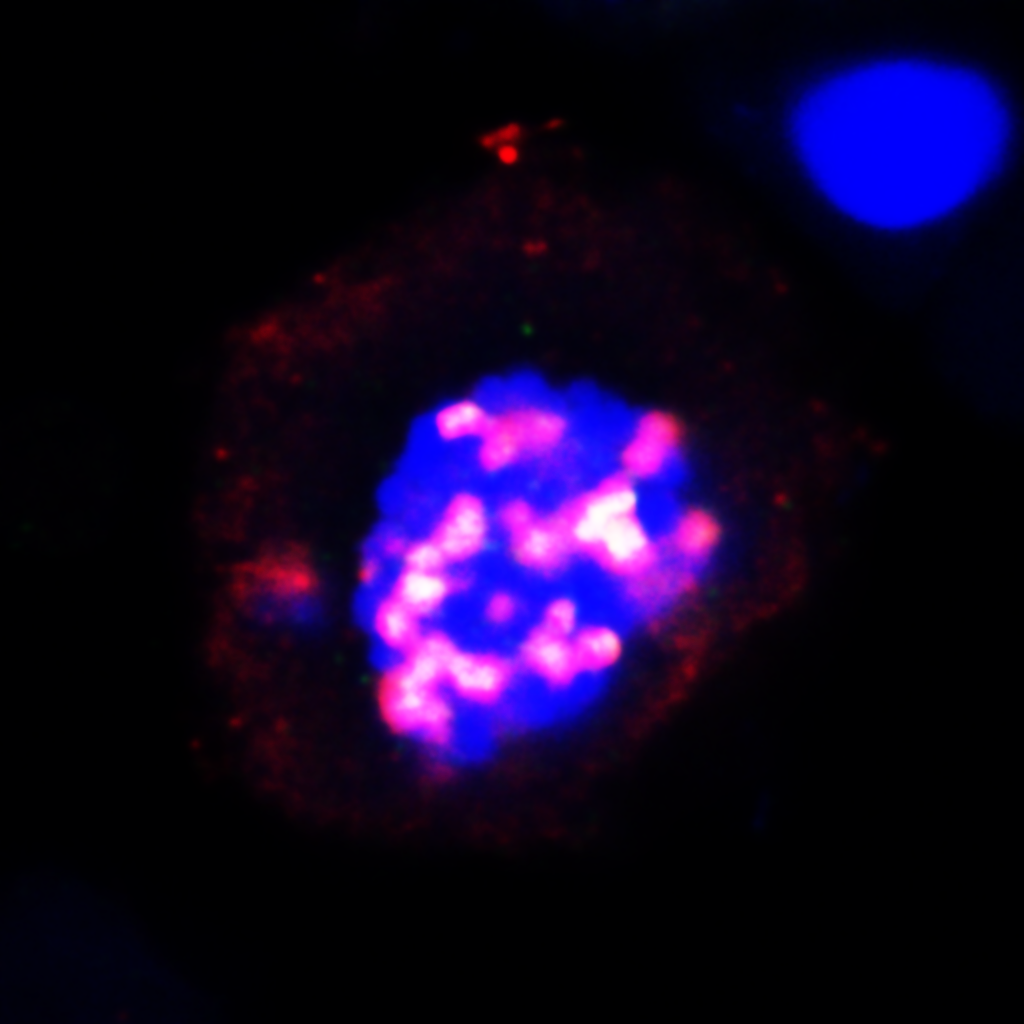

Supplement: Supplementary file 8 — Source data Fig. 6 [file 44318_2024_169_MOESM8_ESM.zip › SD_Figure_6.zip/Figure 6/Fig 6I/Si Ctr/S61A.tif]

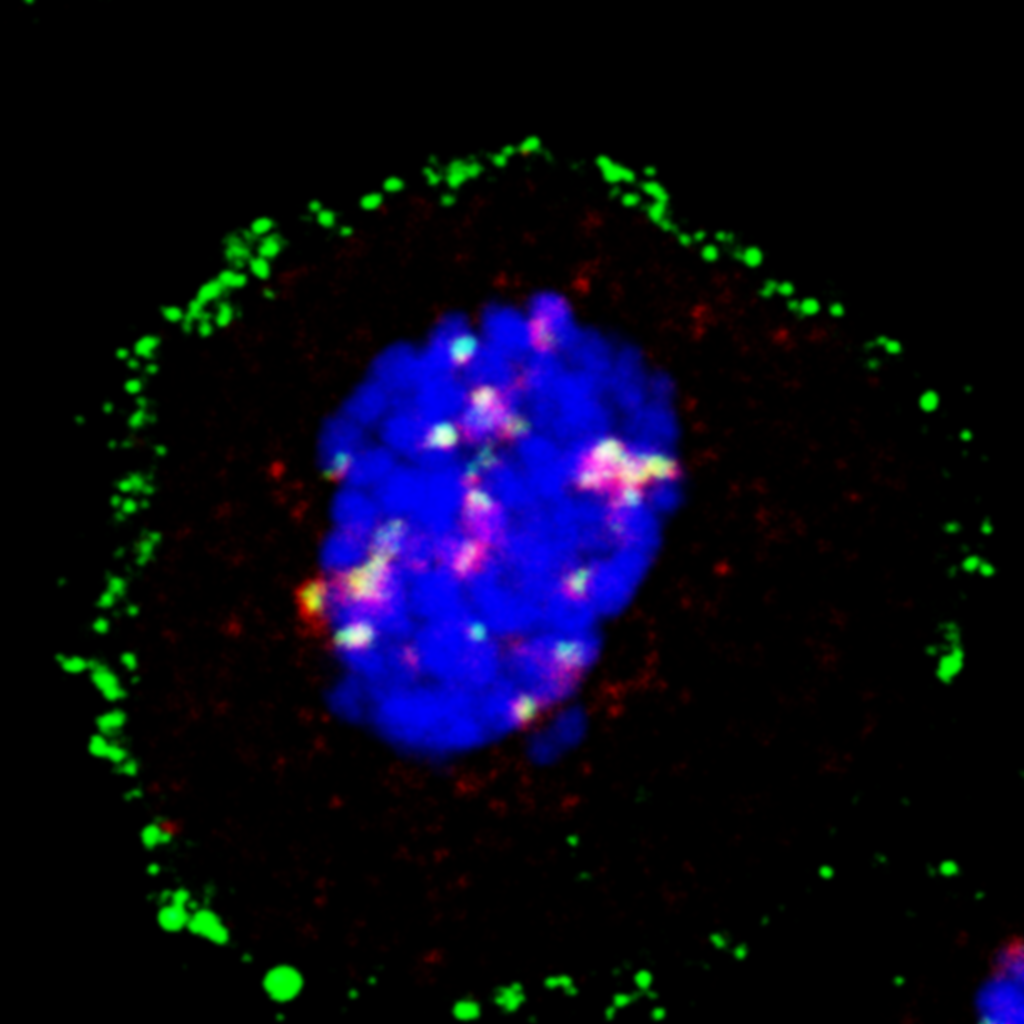

Supplement: Supplementary file 8 — Source data Fig. 6 [file 44318_2024_169_MOESM8_ESM.zip › SD_Figure_6.zip/Figure 6/Fig 6I/Si Ctr/W.tif]

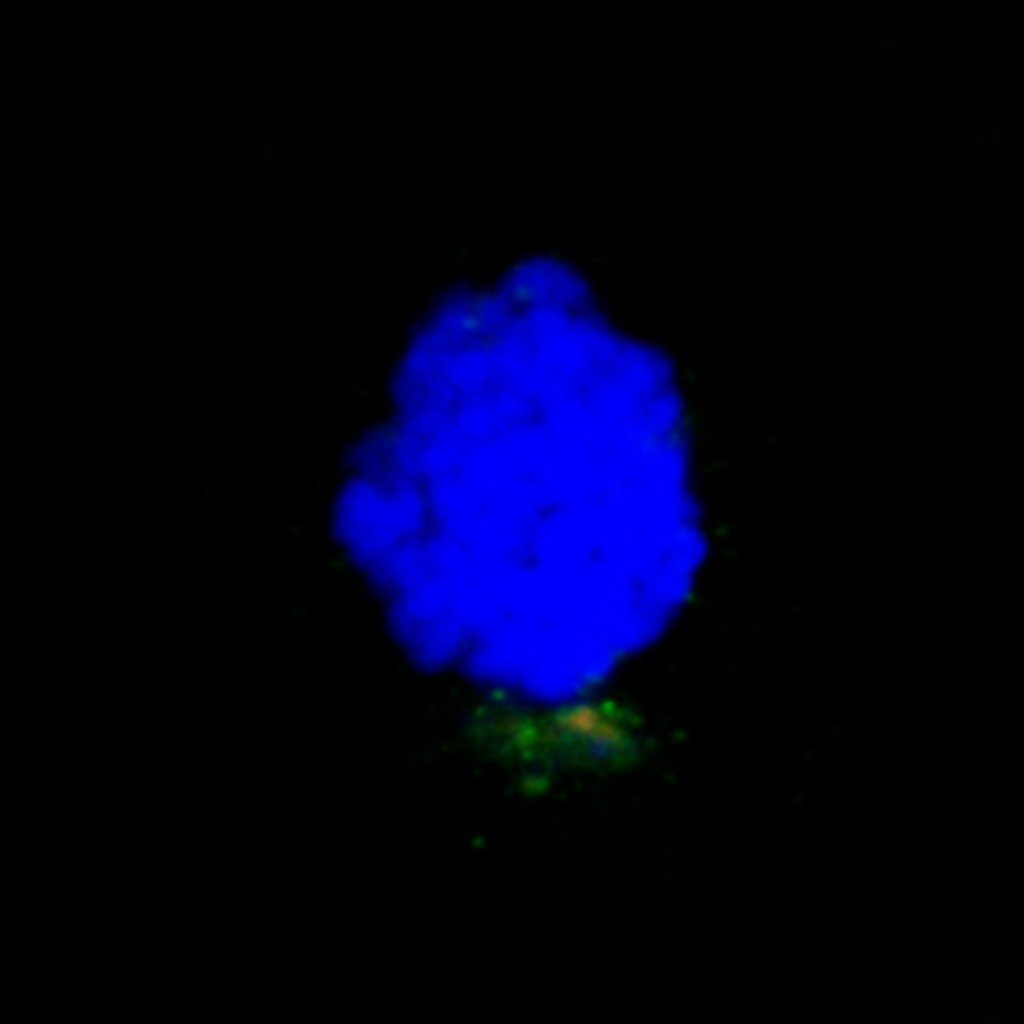

Supplement: Supplementary file 8 — Source data Fig. 6 [file 44318_2024_169_MOESM8_ESM.zip › SD_Figure_6.zip/Figure 6/Fig 6I/Si MUS81/EV.tif]

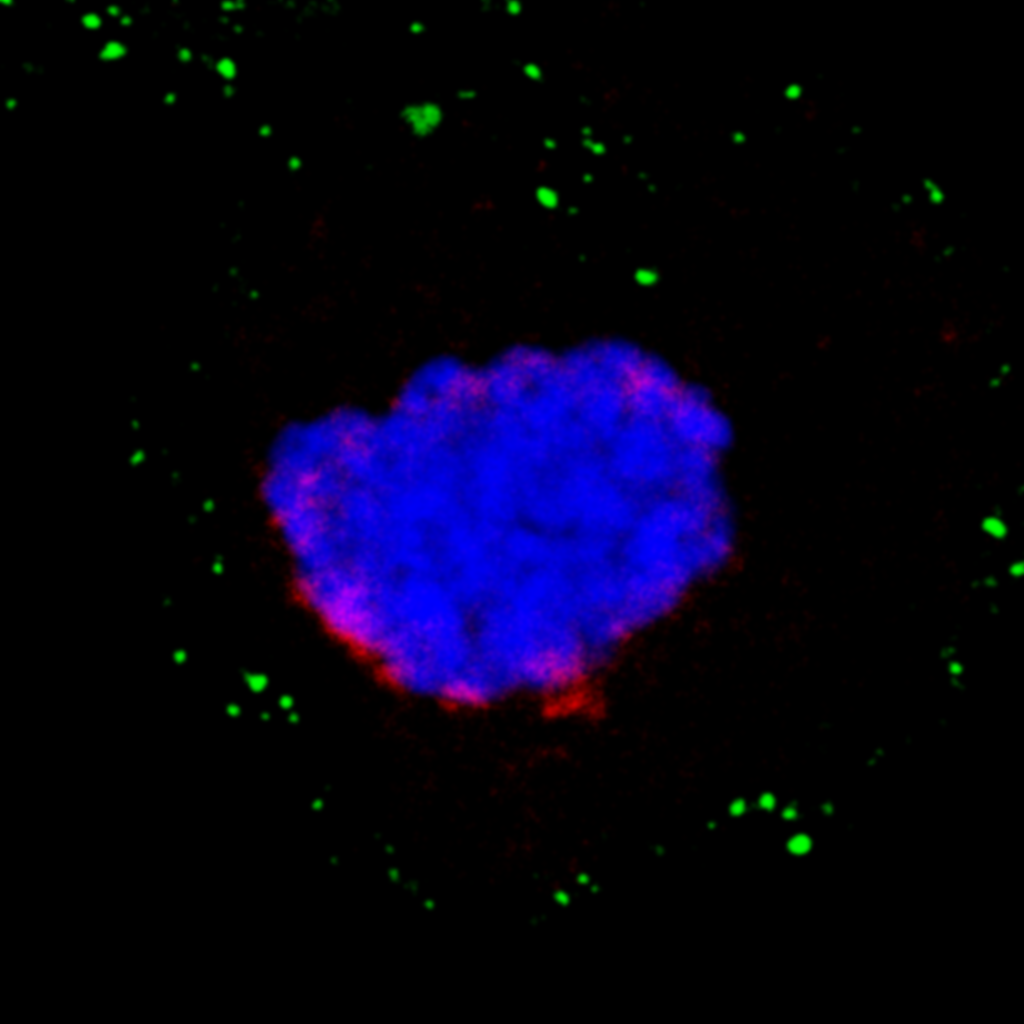

Supplement: Supplementary file 8 — Source data Fig. 6 [file 44318_2024_169_MOESM8_ESM.zip › SD_Figure_6.zip/Figure 6/Fig 6I/Si MUS81/S61A.tif]

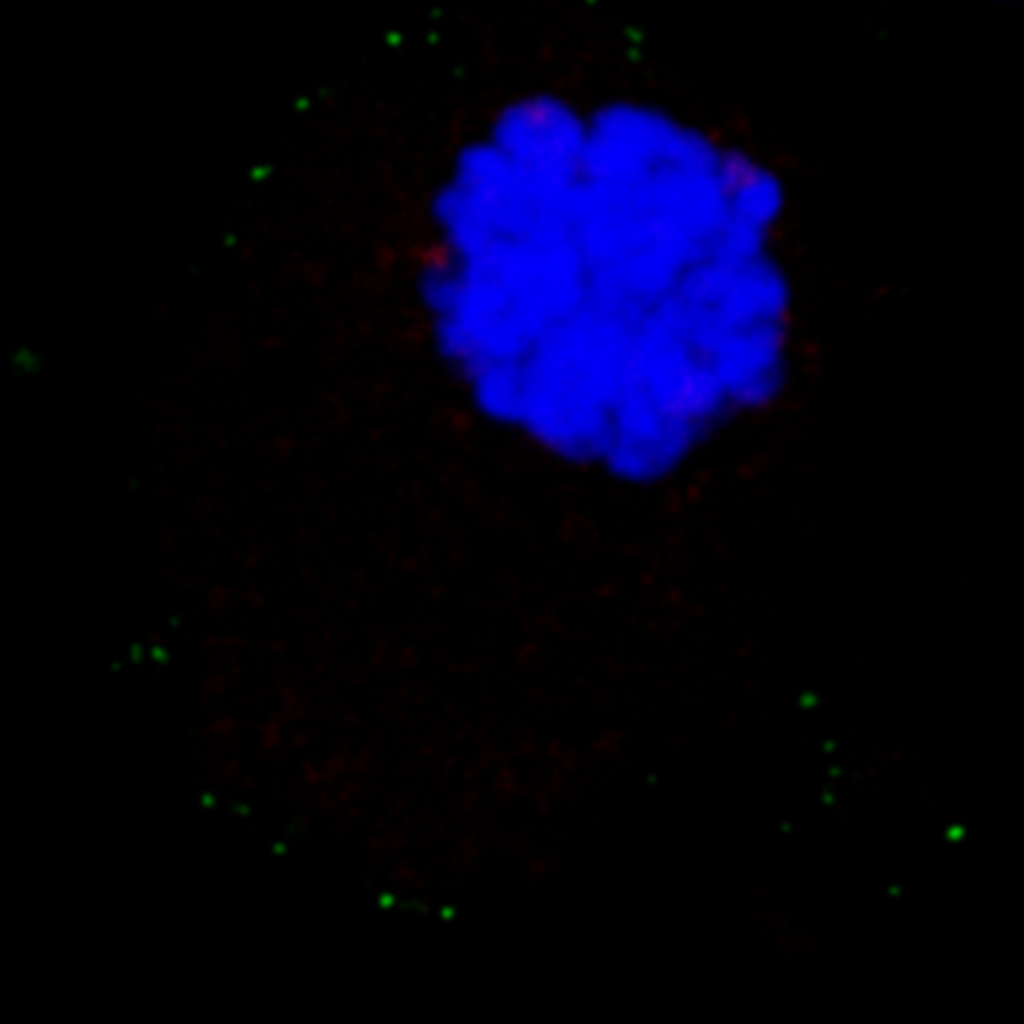

Supplement: Supplementary file 8 — Source data Fig. 6 [file 44318_2024_169_MOESM8_ESM.zip › SD_Figure_6.zip/Figure 6/Fig 6I/Si MUS81/WT.tif]

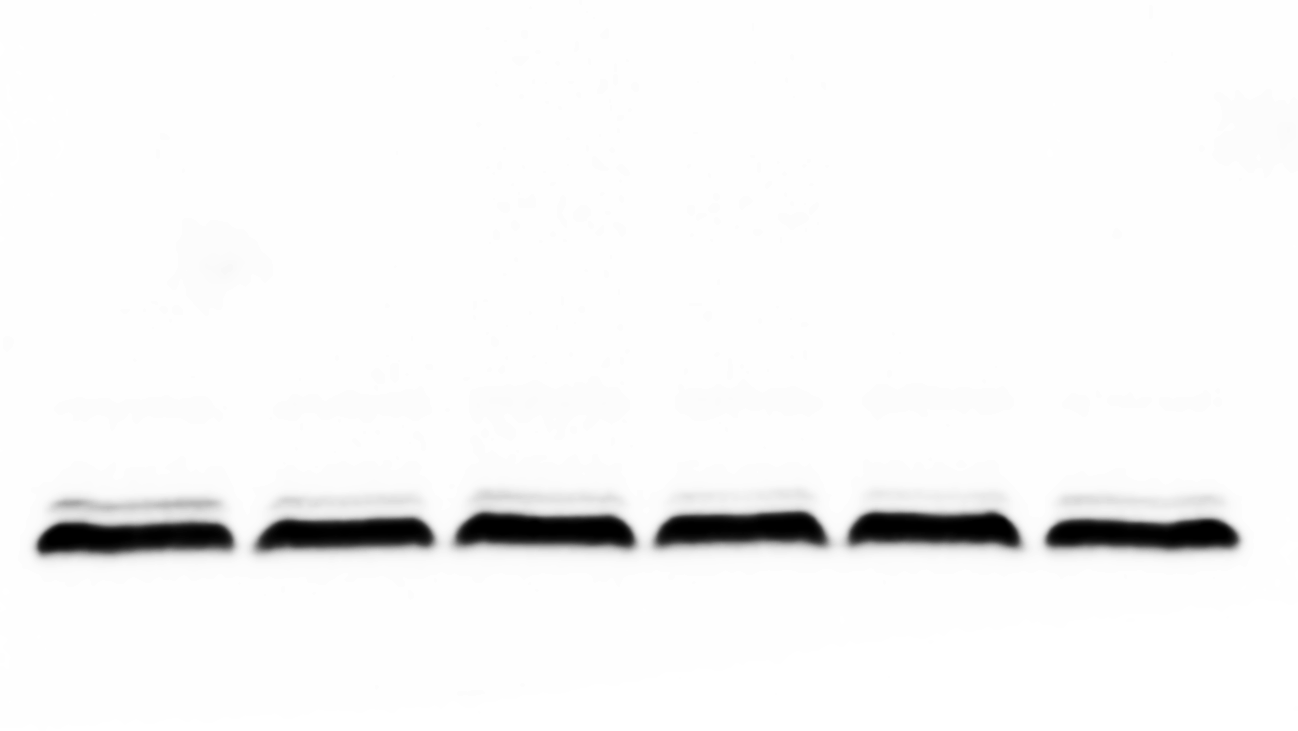

Supplement: Supplementary file 8 — Source data Fig. 6 [file 44318_2024_169_MOESM8_ESM.zip › SD_Figure_6.zip/Figure 6/Fig 6J/actin.tif]

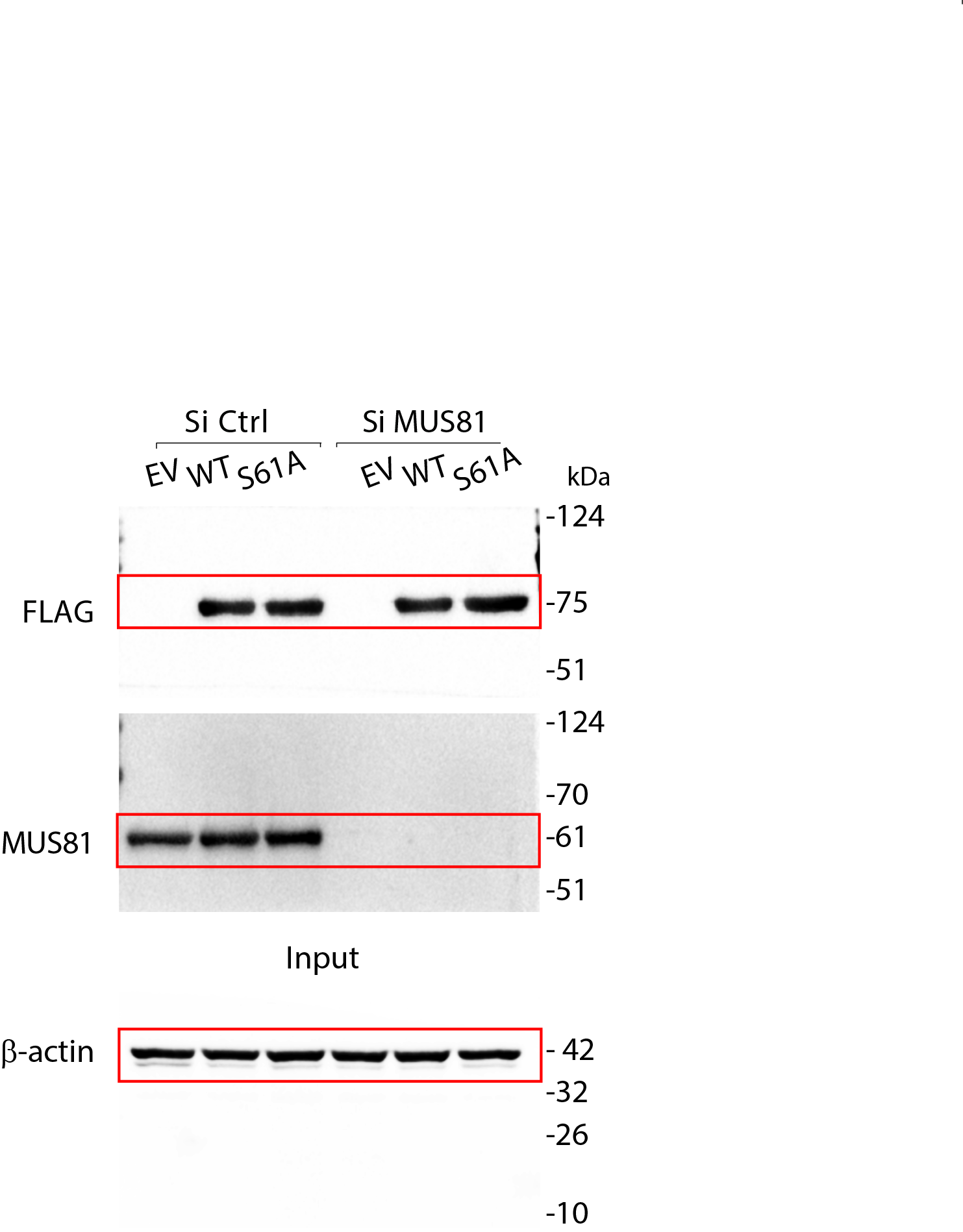

Supplement: Supplementary file 8 — Source data Fig. 6 [file 44318_2024_169_MOESM8_ESM.zip › SD_Figure_6.zip/Figure 6/Fig 6J/Fig 6J.tif]

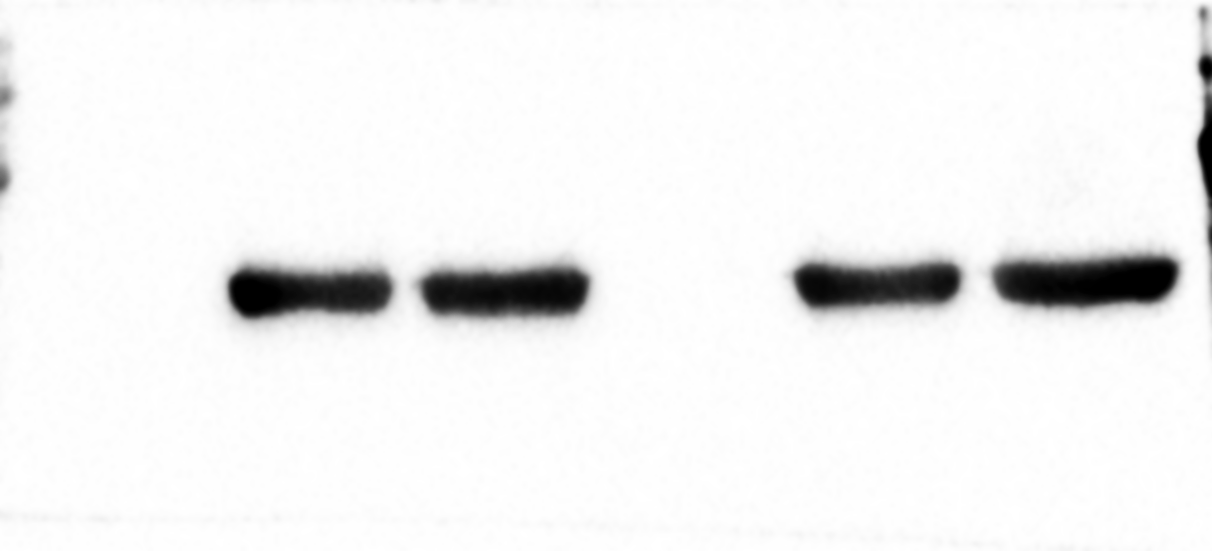

Supplement: Supplementary file 8 — Source data Fig. 6 [file 44318_2024_169_MOESM8_ESM.zip › SD_Figure_6.zip/Figure 6/Fig 6J/FLAG.tif]

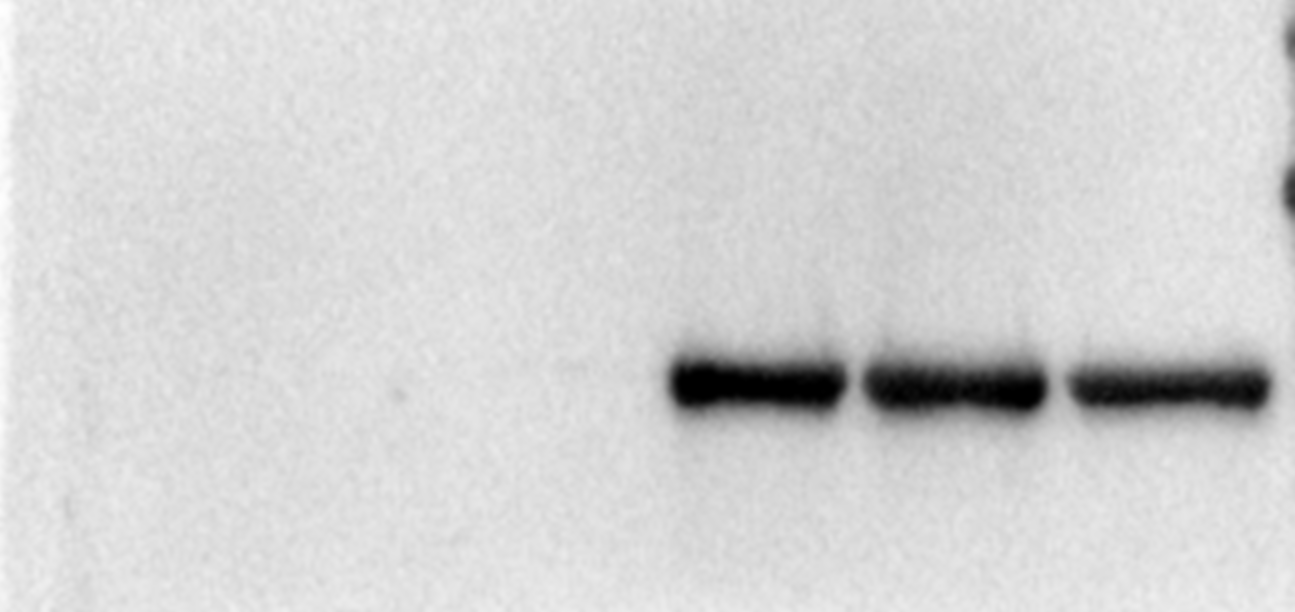

Supplement: Supplementary file 8 — Source data Fig. 6 [file 44318_2024_169_MOESM8_ESM.zip › SD_Figure_6.zip/Figure 6/Fig 6J/MUS81.tif]

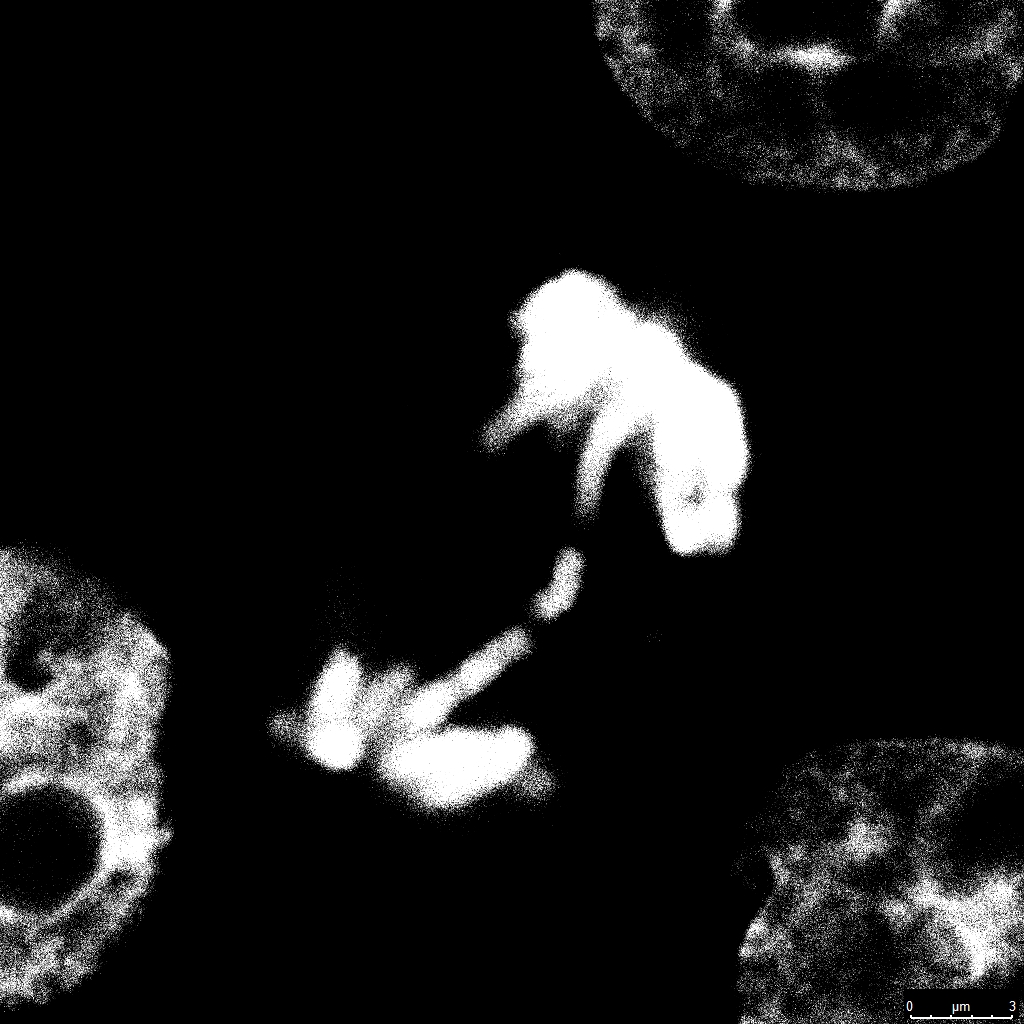

Supplement: Supplementary file 9 — Source data Fig. 7 [file 44318_2024_169_MOESM9_ESM.zip › SD_Figure_7.zip/Figure 7/Fig 7A/AB.jpg]

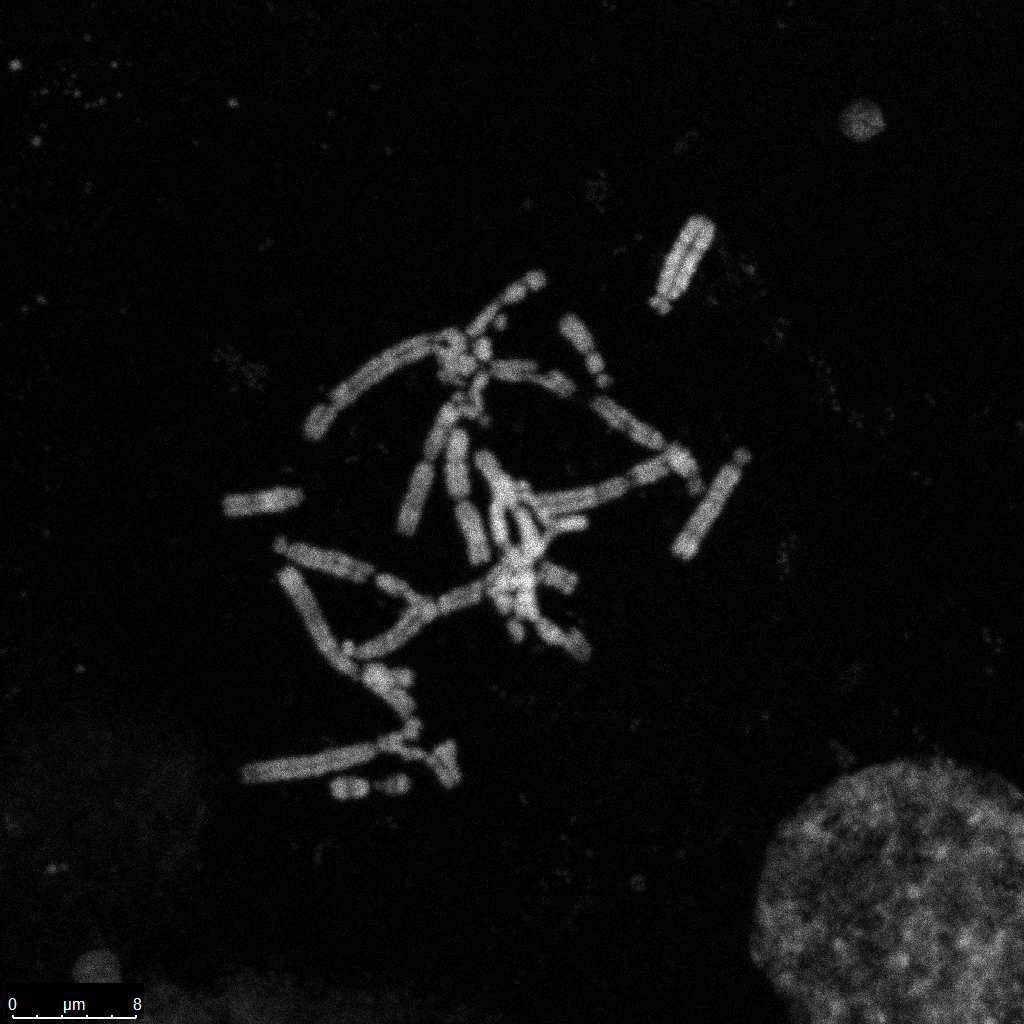

Supplement: Supplementary file 9 — Source data Fig. 7 [file 44318_2024_169_MOESM9_ESM.zip › SD_Figure_7.zip/Figure 7/Fig 7A/CB.tif]

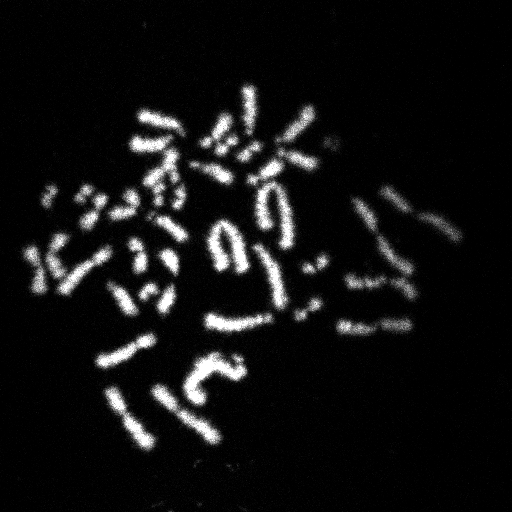

Supplement: Supplementary file 9 — Source data Fig. 7 [file 44318_2024_169_MOESM9_ESM.zip › SD_Figure_7.zip/Figure 7/Fig 7A/CD.tif]

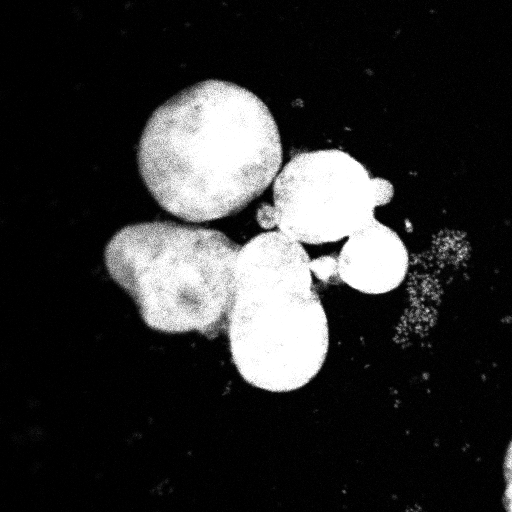

Supplement: Supplementary file 9 — Source data Fig. 7 [file 44318_2024_169_MOESM9_ESM.zip › SD_Figure_7.zip/Figure 7/Fig 7A/MN.tif]

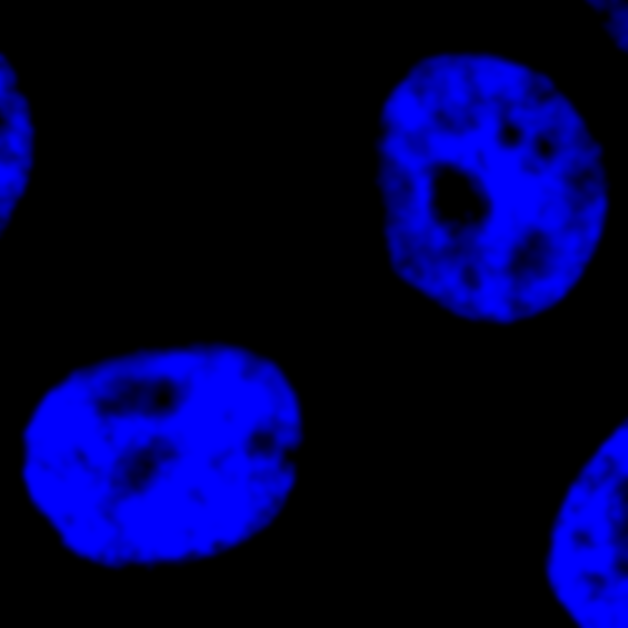

Supplement: Supplementary file 9 — Source data Fig. 7 [file 44318_2024_169_MOESM9_ESM.zip › SD_Figure_7.zip/Figure 7/Fig 7F/7F1.tif]

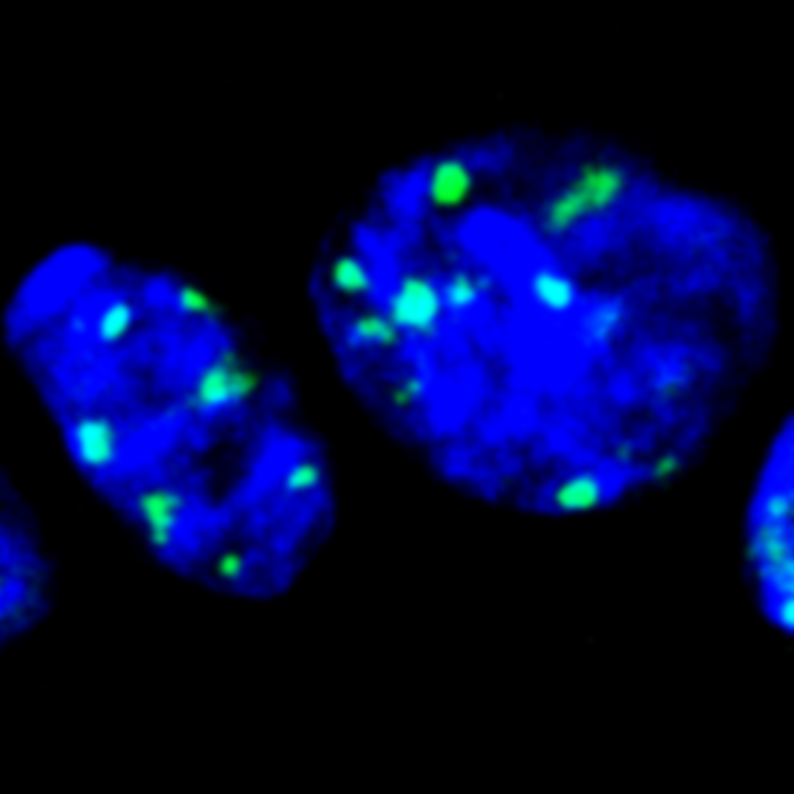

Supplement: Supplementary file 9 — Source data Fig. 7 [file 44318_2024_169_MOESM9_ESM.zip › SD_Figure_7.zip/Figure 7/Fig 7F/7F2.tif]

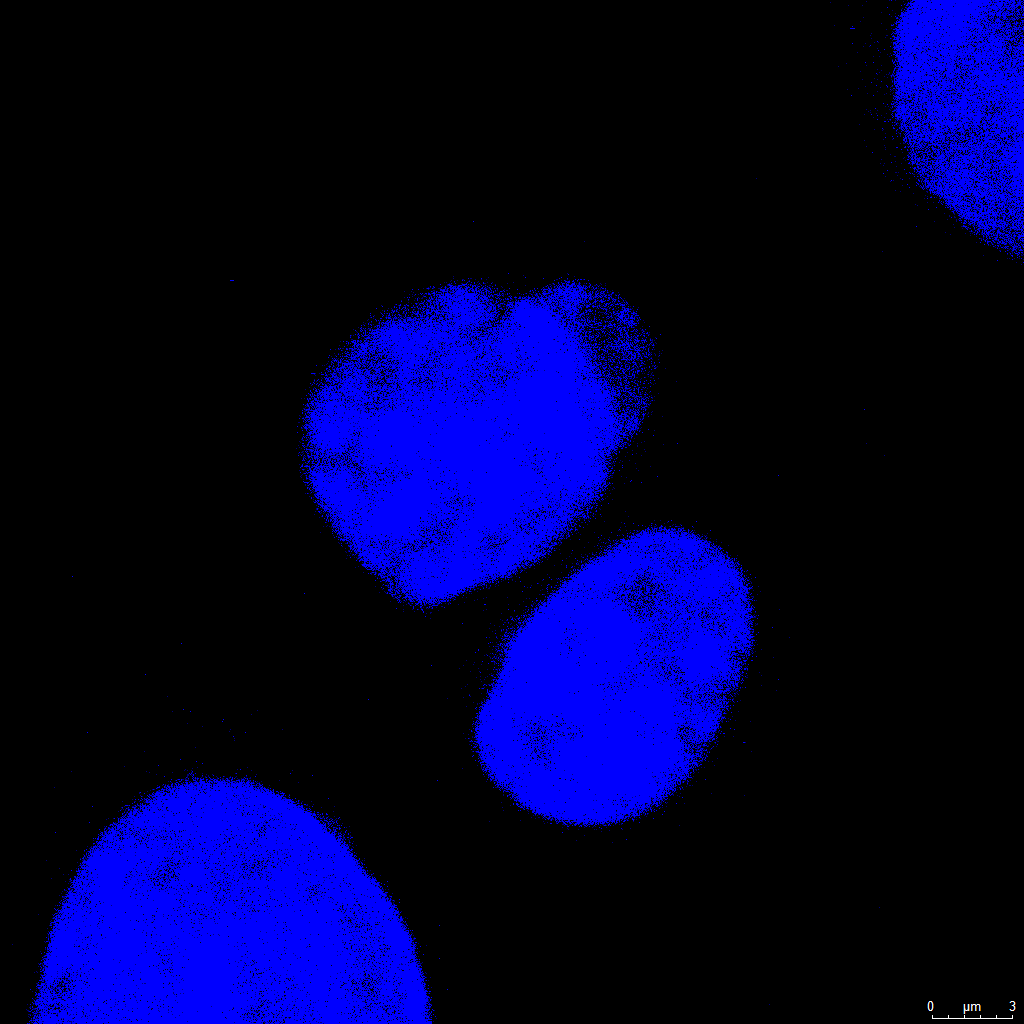

Supplement: Supplementary file 9 — Source data Fig. 7 [file 44318_2024_169_MOESM9_ESM.zip › SD_Figure_7.zip/Figure 7/Fig 7F/7F3.tif]

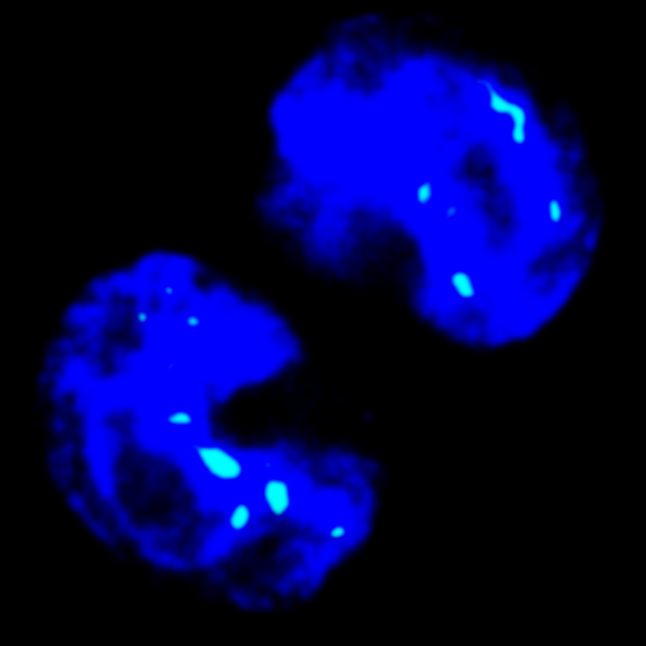

Supplement: Supplementary file 9 — Source data Fig. 7 [file 44318_2024_169_MOESM9_ESM.zip › SD_Figure_7.zip/Figure 7/Fig 7F/7F4.tif]

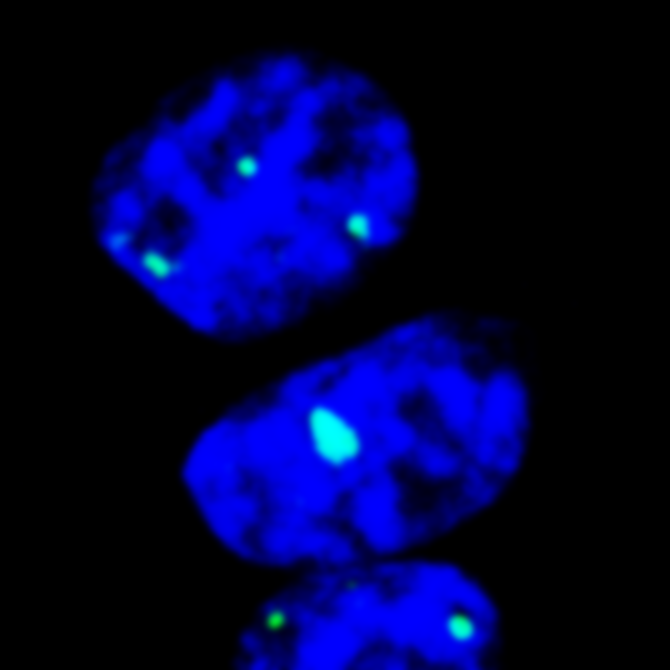

Supplement: Supplementary file 9 — Source data Fig. 7 [file 44318_2024_169_MOESM9_ESM.zip › SD_Figure_7.zip/Figure 7/Fig 7F/7F5.tif]

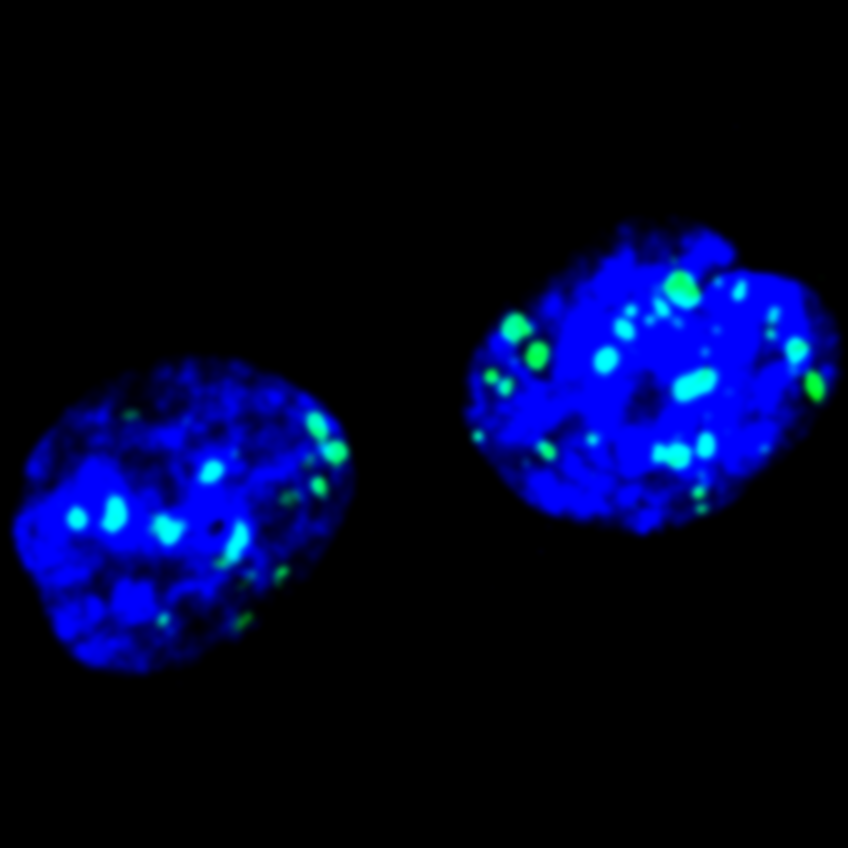

Supplement: Supplementary file 9 — Source data Fig. 7 [file 44318_2024_169_MOESM9_ESM.zip › SD_Figure_7.zip/Figure 7/Fig 7F/7F6.tif]

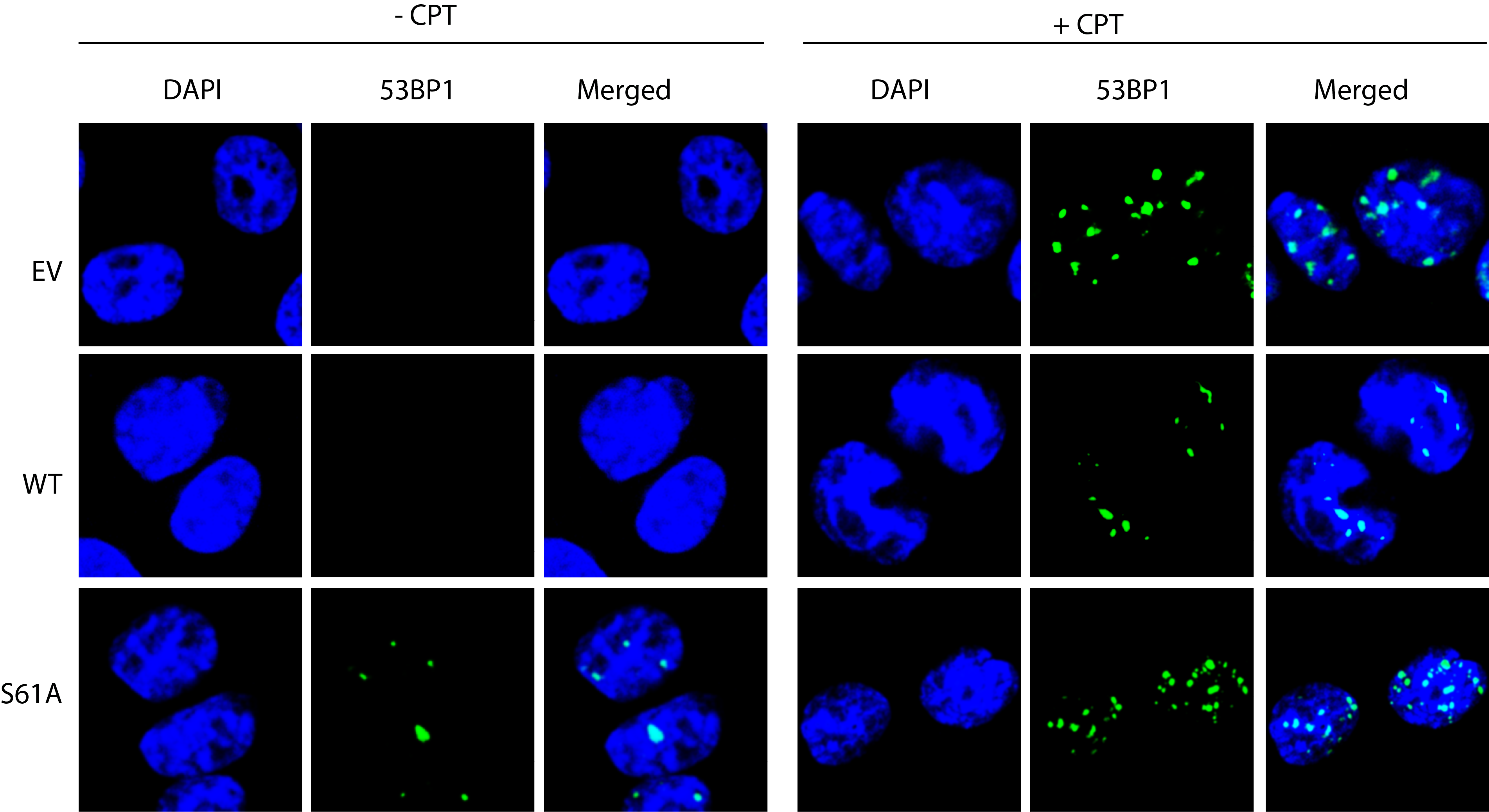

Supplement: Supplementary file 9 — Source data Fig. 7 [file 44318_2024_169_MOESM9_ESM.zip › SD_Figure_7.zip/Figure 7/Fig 7F/Fig 7F.tif]

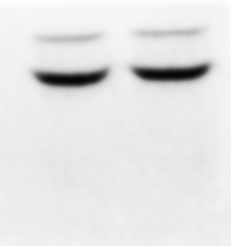

Supplement: Supplementary file 9 — Source data Fig. 7 [file 44318_2024_169_MOESM9_ESM.zip › SD_Figure_7.zip/Figure 7/Fig 7I/Actin.tif]

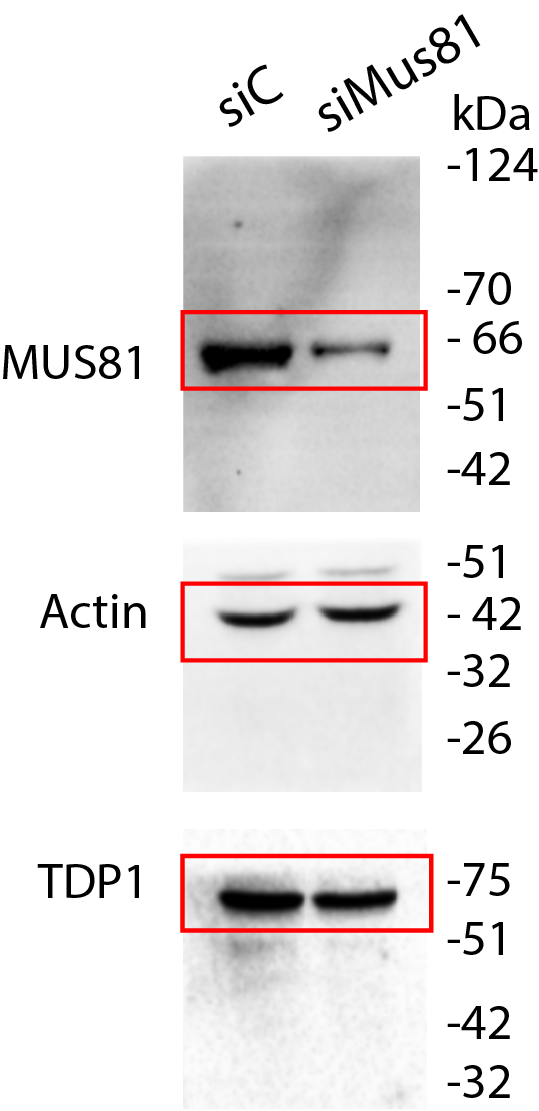

Supplement: Supplementary file 9 — Source data Fig. 7 [file 44318_2024_169_MOESM9_ESM.zip › SD_Figure_7.zip/Figure 7/Fig 7I/Fig 7I.tif]

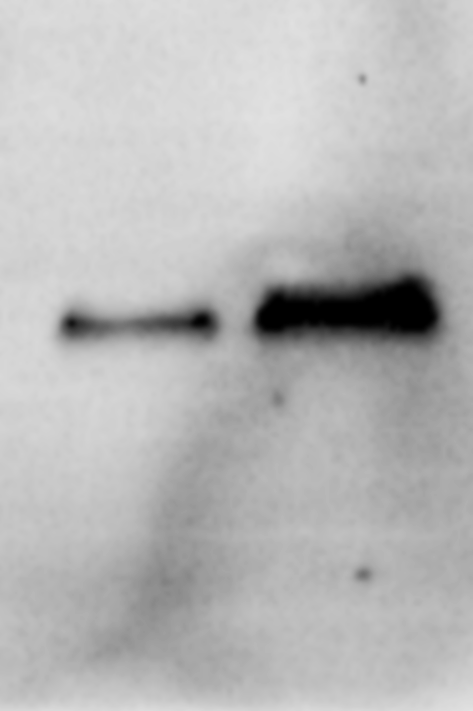

Supplement: Supplementary file 9 — Source data Fig. 7 [file 44318_2024_169_MOESM9_ESM.zip › SD_Figure_7.zip/Figure 7/Fig 7I/MUS81.tif]
