# Supplementary figures and images for: TDP1 phosphorylation by CDK1 in mitosis promotes MUS81-dependent repair of trapped Top1-DNA covalent complexes (part 3 of 3)
Source: EMBO J. 2024 Jul 16;43(17):3710–32. doi: 10.1038/s44318-024-00169-3 (PMC11377750; doi:10.1038/s44318-024-00169-3)

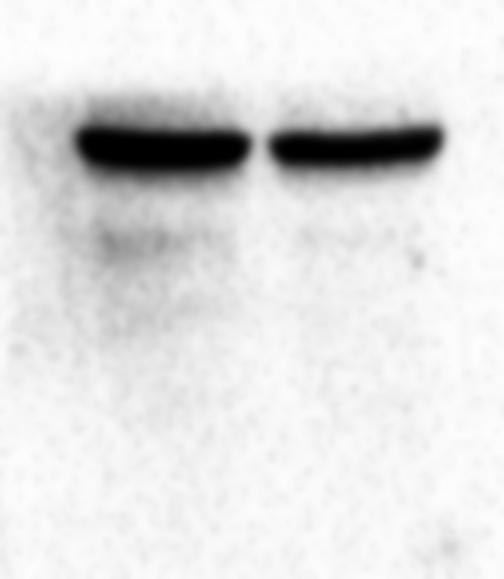

Supplement: Supplementary file 9 — Source data Fig. 7 [file 44318_2024_169_MOESM9_ESM.zip › SD_Figure_7.zip/Figure 7/Fig 7I/TDP1.tif]
